# Discovery of VEGFR‐2 Inhibitors From *Leonurus japonicus* via Integrated Genetic Authentication, Molecular Networking, and In Silico Analysis

**DOI:** 10.1002/ardp.70249

**Published:** 2026-08-13

**Authors:** Thiyagarajan Raviraj, Chen‐Lin Yu, Aekkhaluck Intharuksa, Shang‐Chih Lai, Vidya Febrasca Tenderly, Yen Chi Loo, Pin‐Wei Chen, Shih‐Wei Wang, Kartiko Arif Purnomo, Soundar Rajan Kulandhaivel, Sedin Renadi, Stephen Lirio, Yuan‐Bin Cheng, Yu‐Liang Yang, Tsong‐Long Hwang, Fang‐Rong Chang, Michal Korinek

**Affiliations:** ^1^ Graduate Institute of Natural Products, College of Pharmacy Kaohsiung Medical University Kaohsiung Taiwan; ^2^ Department of Pharmaceutical Sciences, Faculty of Pharmacy Chiang Mai University Chiang Mai Thailand; ^3^ Institute of Biomedical Sciences MacKay Medical University Taiwan; ^4^ Department of Medical Research Hsinchu MacKay Memorial Hospital Hsinchu City Taiwan; ^5^ School of Post‐Baccalaureate Chinese Medicine Tzu Chi University Hualien City Hualien County Taiwan; ^6^ Department of Medicine MacKay Medical University New Taipei City Taiwan; ^7^ Department of Plant Pathology and Microbiology, College of Bio‐Resources and Agriculture National Taiwan University Taipei Taiwan; ^8^ Center for Drug Research and Development and Graduate Institute of Health Industry Technology, College of Human Ecology Chang Gung University of Science and Technology Taoyuan Taiwan; ^9^ Department of Bioinformatics Alagappa University Karaikudi Tamil Nadu India; ^10^ Department of Chemistry Chung Yuan Christian University Taoyuan City Taiwan; ^11^ Department of Marine Biotechnology and Resources National Sun Yat‐sen University Kaohsiung Taiwan; ^12^ Agricultural Biotechnology Research Center, Academia Sinica Chang Gung University of Science and Technology Taipei Taiwan; ^13^ Department of Medical Research, Kaohsiung Medical University Hospital Kaohsiung Medical University Kaohsiung Taiwan; ^14^ Drug Development and Value Creation Research Center Kaohsiung Medical University Kaohsiung Taiwan

**Keywords:** anti‐angiogenesis, DNA barcoding, in silico analysis, *Leonurus japonicus*, molecular networking

## Abstract

Angiogenesis, regulated by vascular endothelial growth factor (VEGF), is crucial in tumor growth, metastasis, and inflammation. *Leonurus japonicus* Houtt., a traditional Chinese herb, was investigated for its anti‐angiogenic potential. DNA sequencing confirmed its identity, distinguishing it from the common morphological misidentification with *Leonurus sibiricus* by the public. Using UHPLC‐MS/MS and GNPS (Global Natural Products Social) molecular networking on the active methanol partition, over 6000 nodes were grouped into 273 clusters. Diterpenoids and flavonoids were targeted. Six compounds were successfully isolated from *L. japonicus*, including four labdane‐type diterpenoids (**1**–**4**) and two flavonoids (**5**, **6**) using column chromatography. Compound **1**, (–)‐8*S*‐acetoxy‐15,16‐epoxy‐8,9‐seco‐13(16),14‐labdadiene, was a new stereoisomer, while Compound **6**, nevadensin, was reported for the first time from this species. Compounds were characterized by NMR, ESI‐MS, and ECD. At 50 μM, Compounds **1**, **4**, and **5** exhibited inhibitory effects in endothelial progenitor cell (EPC) tube formation. Molecular docking and dynamics simulations supported this anti‐angiogenic activity. Compound **1** showed the highest VEGFR‐2 binding affinity (−9.24 kcal/mol), comparable to sunitinib (−9.52 kcal/mol), further verified by an immunoblotting assay. This study demonstrated the successful integration of GNPS and bioactivity‐guided isolation for identifying natural VEGFR‐2 inhibitors. Our findings highlight Compounds **1** and **4** as promising lead candidates for anti‐angiogenic therapy.

## Graphical abstract

Genetic authentication of *Leonurus japonicus* was confirmed using ITS sequencing. UHPLC‐MS/MS‐GNPS analysis and isolation led to a new labdane diterpenoid (**1**) and known compounds (**2**–**6**), with anti‐angiogenic activity validated by EPC assays and VEGFR‐2 docking studies.

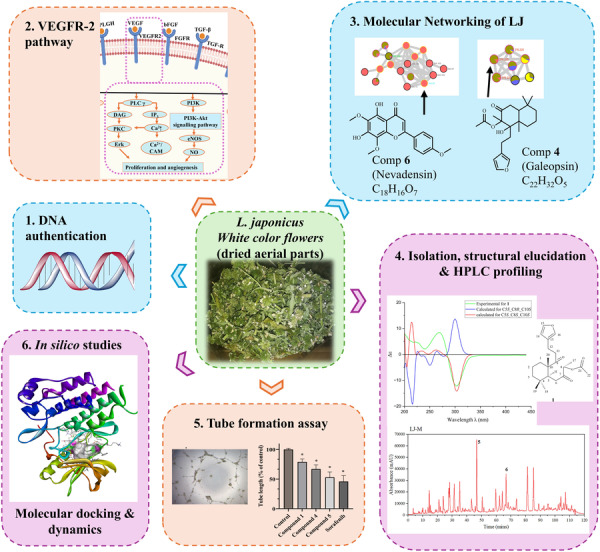

## 1 Introduction

*Leonurus japonicus* Houttuyn (LJ), commonly referred to as Chinese motherwort (益母草Yìmǔcǎo) [1], is an herbaceous flowering plant from the Lamiaceae family, native to Asia. It is widely distributed across Africa, Europe, North America, South America, and Asia, particularly Japan, South Korea, Laos, Malaysia, Myanmar, Taiwan, Thailand, Cambodia, China, and Vietnam. It is traditionally used for gynecological health and circulatory disorders due to its renowned medicinal properties [2].

*L. japonicus* contains diverse bioactive compounds, including diterpenes, alkaloids, essential oils, flavones, glycosides, and terpenoids [2]. These secondary metabolites contribute to its extensive pharmacological activities, which encompass anti‐oxidant [3, 4, 5], anti‐inflammatory, cardioprotective, dysmenorrhea, diuretic, promote blood circulation [4, 5], anti‐melanogenesis [6], analgesic, antimicrobial, promote wound healing (wound contraction, collagen synthesis) [7], anti‐tumor activities, anti‐platelet aggregation, immunomodulation, neuroprotection [8], hepatoprotective, and vasorelaxant [9].

In recent years, deoxyribonucleic acid (DNA) barcoding has been recognized as a highly effective tool for identifying and authenticating herbs, including cosmetic, medicinal, nutraceutical, and pharmaceutical products [10]. This method is widely employed to ensure safety monitoring and quality control, providing a reliable approach to verify the authenticity and purity of products, thereby safeguarding health and industry standards for consumers [11]. In Austria, *L. japonicus* and *L. sibiricus* have been regarded as synonyms, leading to confusion and misidentification of plant materials [12]. In previous reports, red‐colored flowers [2, 13, 14], or white‐colored flowers [15, 16], were also misidentified and described as *L. japonicus*. The challenge in accurate identification arises from the subtle morphological differences between these species [12]. In Taiwan, traditional knowledge has distinguished these plants based on flower color, categorizing purple (red) flowers as *L. japonicus* and white flowers as *L. sibiricus*. However, according to the Flora of China, both species exhibit a range of corolla colors from white to reddish or purplish red [1].

**Figure 1:**
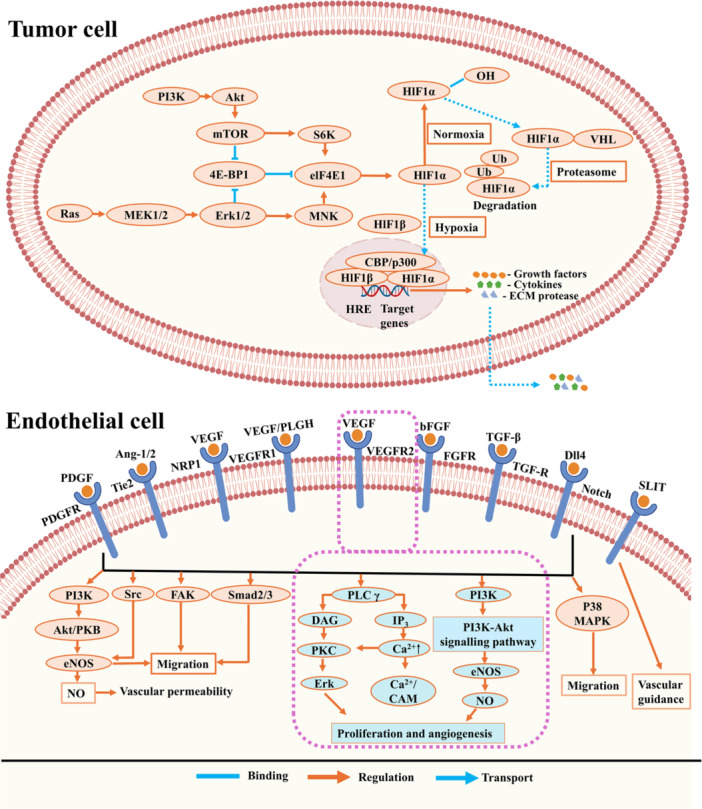
Schematic representation of the VEGFR‐2‐mediated angiogenesis signaling pathway.

Angiogenesis, the formation of new blood vessels, is regulated by several factors, with vascular endothelial growth factor (VEGF) being the most prominent pro‐angiogenic signal factor. VEGFs interact with three receptors (VEGFRs): vascular endothelial growth factor receptor‐1 (VEGFR‐1), VEGFR‐2, and VEGFR‐3 [17], initiating an intracellular signaling cascade upon binding. VEGF binds to tyrosine kinase receptors, leading to the activation of intracellular signaling pathways. VEGFs play a crucial role in various pathological conditions, including inflammatory diseases (e.g., arthritis), macular degeneration, tumor growth, and metastasis. The VEGF family in humans includes VEGF‐A, VEGF‐B, VEGF‐C, VEGF‐D, VEGF‐E, VEGF‐F, endocrine gland‐derived vascular endothelial growth factor (EG‐VEGF), and the placenta growth factor (PlGF). Each possesses valuable biological functions that exert pro‐angiogenic functions in normal and tumor cells [18]. Tumor progression is strongly associated with endothelial cell activation. When VEGFs bind to VEGFR‐2, they stimulate endothelial cells to secrete von Willebrand factor (vWF) [19]. VEGFR‐2 is the primary receptor in vascular endothelial cell biology, governing normal and pathological angiogenesis. VEGF‐A and VEGF‐E show higher binding affinity to VEGFR‐2. In contrast, VEGF‐C and VEGF‐D have a higher affinity for VEGFR‐3 [20]. The binding of VEGF to VEGFR‐2 triggers key signaling pathways involving protein kinase C (PKC), mitogen‐activated protein kinase (MAPK), as well as autophosphorylation of tyrosine residues that lead to functions related to angiogenesis, anti‐apoptosis, differentiation, endothelial cell survival, migration, proliferation, and vascular permeability [18]. The role of VEGFR‐2 in VEGF stimulation is well established. VEGF and/or VEGFR‐2 signaling inhibition has become a promising target for cancer therapy [21]. A diagrammatic representation is shown in Figure 1. Anti‐angiogenic effects involve suppressing the proliferation, migration, and invasion of endothelial cells [22, 23].

This study began with the identification of *L. japonicus* species, and six compounds were isolated. The planar structure of **1** had been reported with unresolved structural ambiguity [24]. However, Compound **1** was obtained as a new isolated stereoisomer after purification, which was clarified through comprehensive spectroscopic analysis. The known compounds were structurally elucidated by comparing their spectroscopic data with literature, and all compounds were evaluated for in vitro anti‐angiogenic activity. In addition, in silico studies assessed their interactions with VEGFR‐2, supporting their potential as angiogenic inhibitors.

## 2 Results and Discussion

### 2.1 DNA Authentication

DNA authentication was used to ensure species identification prior to phytochemical investigation. Genomic DNA was successfully extracted, and four target gene regions, namely ITS (internal transcribed spacer), *mat*K (maturase K gene), *rbc*L (ribulose‐bisphosphate carboxylase gene), and *trn*H‐*psb*A (transfer ribonucleic acid for histidine‐photosystem II protein D1), were amplified using polymerase chain reaction (PCR) and followed by sequencing analysis (Sequence S1.1.1). The nucleotide sequences obtained from these four regions exhibited high similarity to the reference sequences in the DDBJ/EMBL/GenBank database. The results of BLAST similarity‐based identification are summarized in Table 1. The white flower plant material was identified as *L. japonicus* based on the highest maximum score and percentage identity. *L. sibiricus* exhibited nucleotide sequence similarity to the plant material, as indicated by the percentage identity at the *trn*H‐*psb*A and rbcL loci, which were closely related to *L. japonicus*. However, at the ITS locus, the plant sample showed a percent identity of 99.86% with *L. japonicus*, whereas *L. sibiricus* exhibited a lower percent identity of 95.90%. Nucleotide differences within the ITS region were analyzed by comparing selected positions among the collected plant material, *L. japonicus* (MG772941) and *L. sibiricus* (EF395805). A total of 29 variable sites were identified, consisting of 20 single‐nucleotide polymorphisms (SNPs) and 9 insertion–deletion (indel) sites. Details are presented in Table 2. Moreover, the analysis of the Kimura two‐parameter model (K2P) pairwise distance (%) revealed that the distance between the plant material and *L. japonicus* was 0.15% (Sequence S1.1.2). The distance between the plant material and *L. sibiricus* was 2.94% (Sequence S1.1.3). The distance between *L. japonicus* and *L. sibiricus* was 3.10%.

**Table 1:** Sequence similarity of collected plant material to *L. japonicus* and *L. sibiricus* based on four DNA barcoding regions.

DNA barcoding regions	Species of BLAST results	Accession number	Max score	*E* values	Percent identity (%)
ITS	*Leonurus japonicus*	MG772941	1284	0.0	99.86
*mat*K	*Leonurus japonicus*	EF395815	1585	0.0	100.00
*rbc*L	*Leonurus japonicus*	OQ417578	1317	0.0	99.86
*trn*H‐*psb*A	*Leonurus japonicus*	OQ417578	1205	0.0	100.00
ITS	*Leonurus sibiricus*	EF395806	1097	0.0	95.90
*mat*K	*Leonurus sibiricus*	EF395814	1568	0.0	99.88
*rbc*L	*Leonurus sibiricus*	NC_067787	1287	0.0	99.86
*trn*H‐*psb*A	*Leonurus sibiricus*	NC_067787	1177	0.0	100.00

**Table 2:** Nucleotide variation in the ITS regions of collected plant material compared to *L. japonicus* (MG772941) and *L. sibiricus* (EF395805).

	Nucleotide number																			
	6	38	44	67	107	112	126–127	174	223	227	243	373	451	460–462	466–467	531	555	573–574	584–585	604–607
*L. japonicus* (MG772941)	A	A	C	G	C	T	TG	A	C	G	C	T	C	ACC	—	T	C	AA	TG	—
*L. sibiricus* (EF395805)	C	C	G	A	T	C	CA	G	G	A	A	C	T	—	TC	C	T	CC	CA	GCAC
LJ sample	*	*	*	*	*	C	**	*	*	*	*	*	*	***	—	*	*	**	**	—

*Note:* The asterisk (*) indicates the same nucleotide as the top sequence; the em dash (—) indicates nucleotide deletion.

The findings confirm that the plant material used in this study was *L. japonicus*, demonstrating high sequence similarity at multiple loci. However, the close relationship between *L. japonicus* and *L. sibiricus* highlights the critical need for careful marker selection to avoid misidentification. The ITS region served as the most effective marker to distinguish these two species, as it exhibited the highest percent identity with *L. japonicus* and revealed distinct SNP (single‐nucleotide polymorphism) and indel (insertion/deletion) variations.

These data align with the previous suggestion to utilize ITS sequences for comparative analysis within the *Leonurus* genus [25]. Additionally, most cultivated white‐flowered specimens in Taiwan had been misidentified as *L. sibiricus* prior to this study. According to our DNA authentication results, the *Taiwan Herbal Pharmacopeia* currently considers both color variants grown in Taiwan as the same species, which can be used as crude drug materials of *L. japonicus*.

### 2.2 Molecular Network Construction

#### 2.2.1 Global Natural Products Social Molecular Networking (GNPS) – Feature‐Based Molecular Networking (FBMN)

The GNPS‐FBMN analysis of the LJ‐M partition fraction revealed a total of 6632 nodes (each node representing one molecule) and 273 molecular families (clusters comprising at least two nodes). These clusters included diverse chemical classes such as alkaloids, amino acids and peptides, fatty acids, flavonoids, phenylpropanoids, polyketides, steroids, and terpenoids (mono‐, sesqui‐, di‐, and tri‐terpenoids). Among these, terpenoids, particularly labdane‐type diterpenoids, account for 564 nodes. Additionally, 95 flavonoid‐related nodes were also identified. Several nodes remained unannotated, suggesting the presence of potential novel or less‐characterized compounds.

To validate annotations and identify unknown compounds within the GNPS clusters, cross‐referencing was performed using the PubChem database, Reaxys database, and published literature. During this process, 228 nodes related to silica artifacts or lacking SMILES information were excluded, and 236 nodes were retained for further analysis.

Among the identified clusters, three nodes were confidently annotated as previously reported diterpenoids from *L. japonicus*: leoheterin, (+)‐copalol, and galeopsin.

The remaining 233 nodes included a variety of related compounds such as 15‐methoxyleoheteronin B, (24*R*)‐cycloartane‐24,25‐triol‐3*β*‐tetradecanoate, anticopalic acid, cyathisterone, forskolin, leoheteronins A, B, and C, leojapone C, leojaponins A, B, C, and J, leonuketal, methyl myristate, rhodojaponin I and II, schizostatin, sesamin, solidagenone, tenuifolin D, and virescenoside B.

In the GNPS–FBMN analysis, it represented a broad range of diterpenoid subtypes, including acyclic, bicyclic (e.g., labdane‐type), tricyclic, tetracyclic, polycyclic, and spiro‐oxirane diterpenoids. Acetate and diacetate diterpenoids (bicyclic, tricyclic, tetracyclic, pentacyclic, and polycyclic) were identified. Other related classes included diterpenoid alcohols, diterpenoid esters, diterpenoid glycosides, diterpenoid‐quinones, and methylated diterpenoids. Sesterterpenoids, along with their esters and acetates, were also identified. Non‐terpenoid constituents were identified as adamantane derivatives, benzoquinones, fatty acids (including eicosanoid derivatives), phenolic derivatives, and steroidal derivatives.

Flavonoid annotation followed a similar validation process. After removing 28 nodes related to silica or lacking SMILES data, 67 nodes remained. Among these, 5 were confidently matched to previously isolated flavonoids from *L. japonicus*: quercitin, kaempferol‐3‐*O*‐*β*‐d‐glucopyranoside, 1,6‐di‐*O*‐syringoyl‐*β*‐d‐glucopyranose, 2″‐syringylrutin, and apigenin‐7‐*O*‐*β*‐d‐glucopyranoside. The remaining 62 nodes included flavonoids and other related compounds such as 4′‐hydroxy‐2,3‐dihydrocinnamic acid tetracosyl ester, 5,7,3′,4′,5′‐pentamethoxy flavone, apigenin‐7‐*O*‐glucoside, cosmosiin, genkwanin, isoquercitrin, linarin, luteolin, myricetin, nevadensin, nodakenin, quervitin, rutin, and wogonin.

These flavonoid compounds were classified into flavonoid glycosides, flavonoid glycoside esters, and other subclasses. Non‐flavonoid compounds such as polyphenols and xanthone derivatives were also identified.

Many of the above‐labeled compounds were derivatives of known compounds. Their identification was performed based on similarities in core molecular structures and differences in functional groups. These structural similarities were identified through a comparison of SMILES codes with those reported in the literature for the corresponding compounds. Detailed information on identified annotations is provided in Supporting Information S1: Section S1.2. Discussion of compound duplication and limitations of GNPS‐FBMN are addressed in Supporting Information S1: Section 2.2.2.

In conclusion, this comprehensive GNPS‐FBMN analysis predicted chemically diverse profiles with a particular focus on diterpenoids and flavonoids. Notably, nevadensin, a flavonoid isolated for the first time from *L. japonicus* in this study, was also detected. These results highlight the predicted presence of novel or less‐characterized compounds within the dataset.

The abundant compound categories observed in GNPS‐FBMN aligned with a previous review summarizing 284 known constituents from *L. japonicus*, which reported 53.2% terpenoids (151/284), 12.3% flavonoids (35/284), 12.0% alkaloids, amino acids/cyclopeptides (34/284), 8.1% phenylpropanoids (23/284), 7.7% miscellaneous compounds (22/284), and 6.7% steroids (19/284) [26]. To our knowledge, this study represents the first application of GNPS‐FBMN to *L. japonicus*.

Although previous studies reported alkaloids such as leonurine and stachydrine from *L. japonicus* for their pharmacological effects, including angiogenesis [26], these compounds were not detected in this network, potentially due to differences in extraction methodology [27].

#### 2.2.2 GNPS‐FBMN and Its Limitations

##### 2.2.2.1 Challenges and Nuances in Molecular Networking Data Interpretation

Although molecular networking is a powerful untargeted metabolomic tool, interpreting its results, especially across diverse datasets, poses analytical challenges [28]. In this study, data integration of the LJ‐M partition fraction with isolated Compounds **1**–**6** revealed specific limitations in compound confirmation. Despite the inclusion of standard data, only a few compounds successfully matched those previously isolated from *L. japonicus*. This result highlights a key limitation of GNPS analysis, and researchers should exercise greater caution when using the database.

##### 2.2.2.2 Addressing Node Redundancy

Redundant nodes were observed in the molecular network. This phenomenon may arise due to the inherent nature of mass spectrometry data and the networking algorithm. Specifically, the same chemical entity can generate multiple nodes due to (1) its genuine presence across different input samples or (2) the formation of various ionization adducts or ion‐source fragments (e.g., [M + H]^+^, [M + Na]^+^, and [M − H]^+^). Despite originating from a single molecule, these can be resolved as distinct nodes due to specific retention time (RT) differences or subtle variations in fragmentation patterns under the applied networking parameters (e.g., a specific cosine score) [29, 30]. Despite the GNPS algorithm's aim to cluster similar ions, conservative (strict) settings may preserve closely related signals separately to avoid false positives or incorrect grouping. Furthermore, the presence of isomers or very similar isobars, which can yield distinct MS/MS spectra or RTs, could also contribute to multiple nodes representing structurally analogous but not identical molecules [31, 32].

##### 2.2.2.3 Challenges in Identifying Pure Compounds

Although ultra‐high‐performance liquid chromatography coupled to an Orbitrap Fusion Lumos mass spectrometer (UHPLC‐MS/MS) profiles of isolated pure Compounds **1**–**6** were included as internal references, Compounds **1**, **2**, **3**, and **5** were not definitely identified in the final network. Nevertheless, their derivatives or structurally related analogs were detected. This limitation may result from platform‐dependent MS/MS fragmentation variability, suboptimal spectral quality, low compound abundance at injection concentrations in LC‐MS/MS instruments, or mismatches with existing databases [33, 34]. This underscores that while pure standards are invaluable for validation, their successful integration into a network depends on robust spectral quality and compatibility with database‐matching algorithms.

##### 2.2.2.4 Need for Integrated Approaches

These challenges highlight the importance of an integrated, multi‐tiered annotation strategy. While GNPS‐FBMN provides a discovery platform, accurate identification requires combining spectral libraries, manual curation, and structure validation using chemical databases. In this study, SMILES‐based verification and literature matching were employed for the annotation of diterpenoids and flavonoids [35].

#### 2.2.3 Annotation of Isolated Compounds

**Figure 2:**
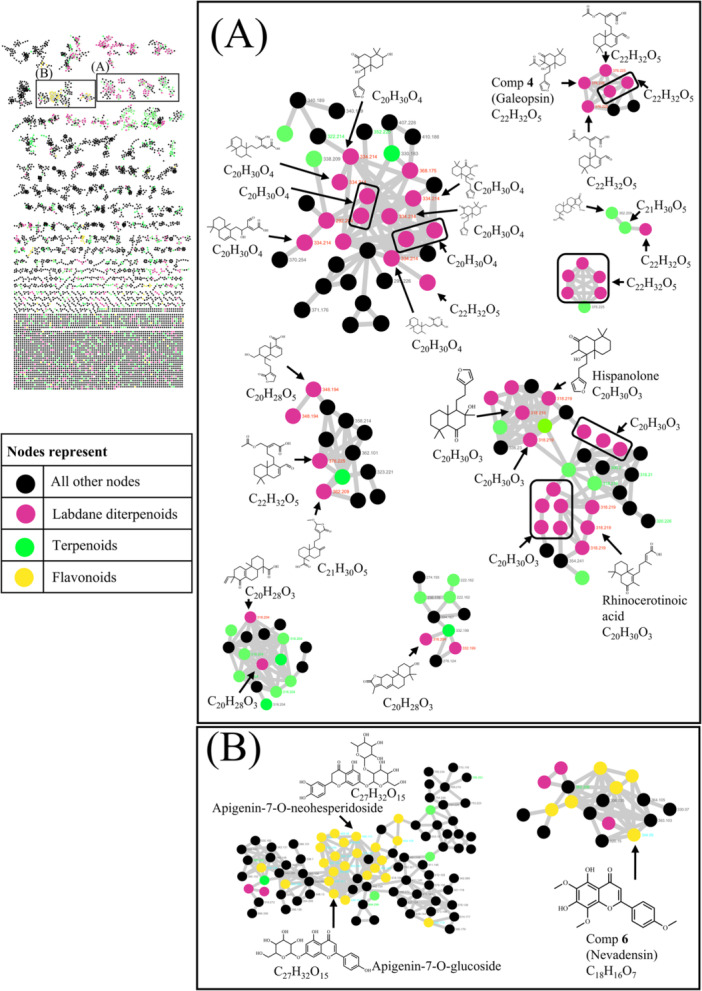
Molecular network of LJ‐M partition fraction (positive mode) and isolated compounds. A comprehensive molecular network was generated from the LJ‐M partition fraction (positive ionization mode), integrated with UHPLC‐MS/MS data of the fraction and isolated Compounds **1**–**6**. Highlighted regions (A) and (B) represent key clusters corresponding to terpenoids (specifically labdane diterpenoids) and flavonoids, respectively. Node fill colors indicate the source of the mass spectral data: green for terpenoids, pink for labdane diterpenoids, yellow for flavonoids, and black for all other nodes. The edge width is proportional to the cosine similarity score, with a minimum threshold of 0.7 between connected nodes.

Following the isolation of Compounds **1**–**6**, UHPLC‐MS/MS data were acquired and integrated with the molecular network of the LJ‐M partition fraction. To enhance visualization and highlight nodes corresponding to the isolated diterpenoids and flavonoids (*m/z* 200–410), relevant clusters were reorganized and color‐coded. This facilitates compound analysis, chemical classification, and annotation Figure 2.

Clusters labeled (A) and (B) were identified as corresponding to labdane diterpenoids and flavonoids, respectively. These clusters contained both annotated isolated compounds and several unannotated nodes, all of which were structurally related to the isolated compounds. Spectral library matching and structure prediction using SIRIUS facilitated the annotation of various terpenoids and flavonoids within these clusters. Importantly, Compounds **4**, **6**, and their derivatives, as well as those of **1**, **2**, **3**, and **5** were successfully mapped within the molecular network. Unannotated nodes with molecular masses similar to the isolated compounds were observed. These nodes may correspond to the isolated pure compounds themselves or their close analogs. These findings highlight the potential for integrating untargeted metabolomics approaches with targeted compound isolation strategies.

Although the anti‐angiogenic potential of diterpenoids remains relatively under‐explored, Kumar et al. [36] reported that andrographolide, a diterpenoid, exerted anti‐tumor activity in an in vivo breast cancer model via inhibition of VEGF pathway. Chao et al. [37] demonstrated that flavonoid apigenin suppressed VEGF expression in human uveal melanoma cell lines by mitigating phosphorylation of Akt and ERK1. Qi et al. [38] investigated the anti‐angiogenic activity of various terpenoids using a rat aortic ring assay. Their finding showed that the diterpenoid eurifoloid Q suppressed both VEGFR expression and Akt phosphorylation, while the triterpenoid 23(*E*)‐eupha‐8,23‐diene‐3*β*,25‐diol‐7‐one specifically inhibited Akt phosphorylation. Wang et al. [39] reported the anti‐angiogenesis activity of caseaveolen H (clerodane diterpenoid) using zebrafish xenograft models. In silico modeling, it also showed high affinity toward VEGFR‐2, a key regulator of angiogenesis. Flavonoids such as apigenin, quercetin, taxifolin, hesperidin, naringenin, epicatechin, kaempferol, and several other flavonoids were reported to possess anti‐cancer activity by VEGRF inhibition [40, 41, 42]. These studies collectively support the observed anti‐angiogenic potential of LJ‐M and its key compounds.

### 2.3 Structure Elucidation

**Figure 3:**
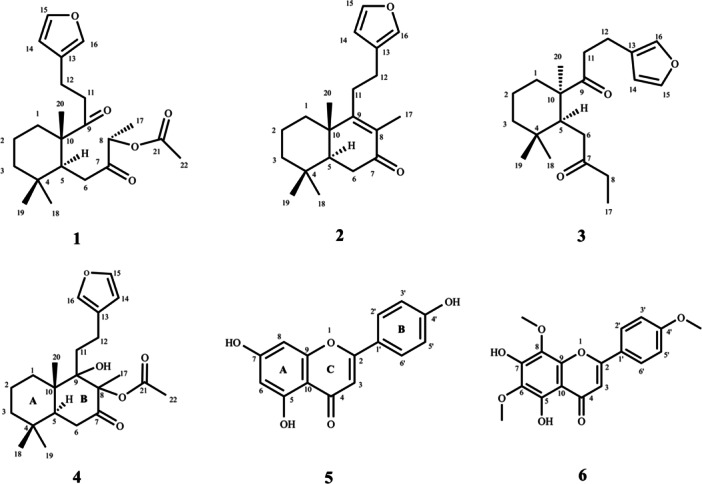
Isolated Compounds **1**–**6** and their structures.

**Figure 4:**
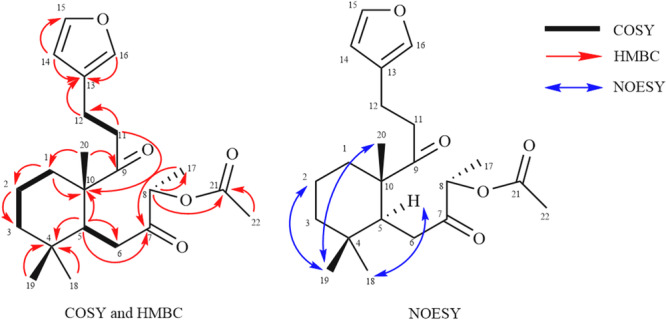
Selected COSY, HMBC, and NOESY interactions of Compound **1**.

Compound **1** was obtained as a white amorphous powder, and its molecular formula was determined to be C_22_H_32_O_5_ by HRESIMS *m/z* 399.21390 [M + Na]^+^ (calculated for C_22_H_32_O_5_Na, 399.21420), deviation 0.0003 *m*/*z*, Δ = −0.75 ppm together with ^1^H and ^13^C NMR. The structure was elucidated based on 1D and 2D NMR (Table 3). The ^1^H NMR spectrum of **1** exhibited resonances of five methyls (including one methyl at *δ*_H_ 1.36 [H‐17] and one methyl attached to the acetoxy group at *δ*_H_ 2.11 [H‐22]), six methylene protons (including two methylene protons of ethane chain at *δ*_H_ 2.53 [1H, dd, *J* = 18.9, 4.8 Hz], 2.21 [1H, dd, *J* = 18.9, 4.8 Hz] for [H‐11] and *δ*_H_ 2.65 [1H, m], 2.45 [1H, m] for [H‐12]), and five methine protons (including three olefinic protons [furan moiety], showed peaks at ppm *δ*_H_ 6.27 [H‐14], 7.32 [H‐15], and 7.21 [H‐16]). ^13^C NMR spectra analysis showed four non‐protonated carbons (including two carbonyl carbons at *δ*_C_ 215.6 [C‐9] and 210.7 [C‐7]), four methyl carbons (including one acetoxy group carbon at *δ*_C_ 170.5 [C‐21] and 20.8 [C‐22]), six methylene carbons, and five methine carbons (including three olefinic carbons at *δ*_C_ 124.4 [C‐14], 111.2 [C‐15], and 139.1 [C‐16]). By comparison of the NMR data, the structure is similar to the known Compound **4** (Figure 3). After careful analysis of HSQC, HMBC, COSY, and NOESY spectral data for spatial arrangements of the substituents (Figure 4), an acetoxy (C‐21, *δ*_C_ 170.5; C‐22, *δ*_H_ 2.11 [3H, s]/*δ*_C_ 20.8), an adjacent methyl (C‐17, *δ*_H_ 1.36 [3H, d, *J* = 6.8 Hz]/*δ*_C_ 16.4), and a ketone (C‐7, *δ*_C_ 206.4) were confirmed to be connected to a methine (C‐8, *δ*_H_ 5.04, q [7.0 Hz]/*δ*_C_ 74.5). A hydroxyl group at C‐9 was oxidized to a ketone. Thus, the planar structure was identified.

**Table 3:** ^1^H and ^13^C NMR data (400 and 100 MHz; *δ* in ppm, *J* in Hz) of Compound **1** in CDCl_3_.

	1	
No.	*δ*_H_ (multi; *J* in Hz)	*δ*_C_, type
1	1.75, m 1.42, m	35.0 CH_2_
2	1.57, m	18.4 CH_2_
3	1.48, m 1.30, m	40.9 CH_2_
4		34.1 C
5	2.45, m^a^	41.4 CH
6	2.85, m 2.65, m^a^	37.7 CH_2_
7		206.4 C
8	5.04, q (7.0)	74.6 CH
9		215.2 C
10		52.3 C
11	2.53, dd (18.9, 4.8) 2.21, dd (18.9, 4.8)	37.0 CH_2_
12	2.65, m^a^ 2.45, m^a^	19.4 CH_2_
13		124.4 C
14	6.27, dd (1.80, 0.9)	111.2 CH
15	7.32, t (1.7)	142.8 CH
16	7.21, dt (1.8, 0.9)	139.2 CH
17	1.36, d (7.0)	16.5 CH_3_
18	0.80, s	32.6 CH_3_
19	0.88, s	22.9 CH_3_
20	1.06, s	17.5 CH_3_
21 OAc		170.5 C
22 OAc	2.11, s	20.9 CH_3_

^a^ Overlapped signals.

Compound **1** has three stereocenters: C‐5, C‐8, and C‐10. According to the NOESY data, relative conformations of C‐5S* and C‐10S* were assigned. After the calculation of ECD spectra, the absolute configuration of C‐5, C‐8, and C‐10 was determined as *S* (Figure 5). Consequently, Compound **1** was identified as (−)‐8*S*‐acetoxy‐15,16‐epoxy‐8,9‐*seco*‐13(16),14‐labdadiene‐7,9‐dione. The stereochemistry of Compound **1** was revealed. Since previous reports lack clear assignments of NMR data for the well‐known Compounds **4** and **6**, we provided their complete 2D NMR spectra in this study (Supporting Information S1: Figures 38–46 for **4** and Supporting Information S1: Figures 52–60 for **6**).

**Figure 5:**
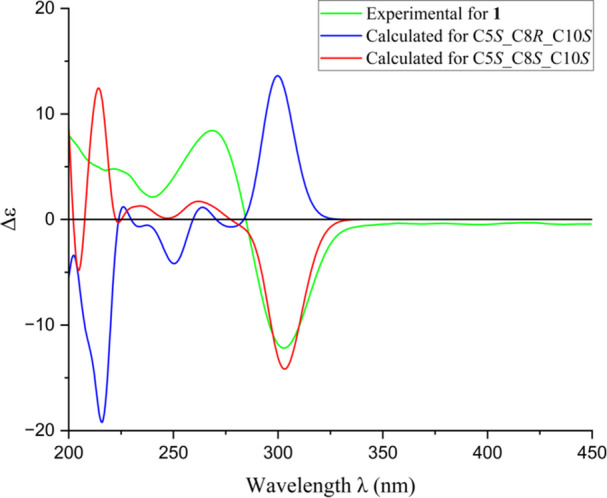
ECD spectra of Compound **1**.

Based on instrumental analyses and comparison of NMR and mass spectral data, Compounds **2**–**6** (Figure 3) were identified as hispanone (**2**) [43], secohispanolone (**3**) [24, 44], galeopsin (**4**) [24, 45], apigenin (**5**) [46], and nevadensin (**6**) [47]. The ^1^H and ^13^C NMR data are presented in Supporting Information S1: Tables 1 and 2.

### 2.4 High‐Performance Liquid Chromatography Isolation and Profiling

The isomer of Compound **1** was separated using chiral high‐performance liquid chromatography (HPLC) with an RT of 9.26 min. The chromatogram is shown in Supporting Information S1: Figure 1.1.

**Figure 6:**
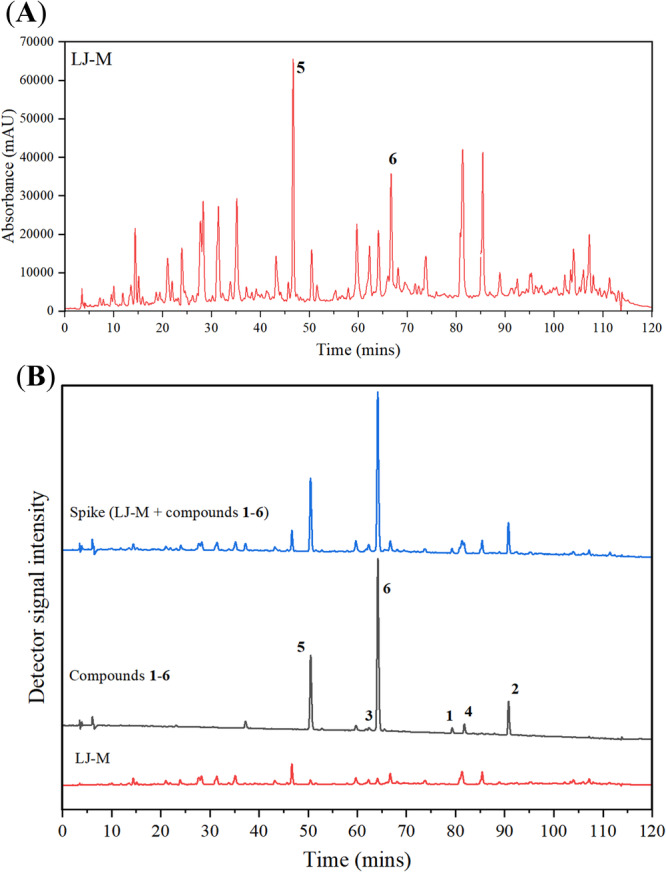
HPLC profiling and spiking chromatograms of LJ‐M and isolated compounds. (A) HPLC chromatogram of LJ‐M, observed at 230 nm wavelength. (B) HPLC spike profiling. The blue trace represents the LJM fraction spiked with the mixture of isolated Compounds **1**–**6**; the black trace represents the isolated Compounds **1**–**6** mixture alone; and the red trace represents the LJ‐M fraction.

HPLC profiling of the bioactive LJ‐M partition fraction from *L. japonicus* was performed to obtain its chromatographic fingerprint at 230 nm (Figure 6A) and 210 nm (Supporting Information S1: Figure 1.2). The calculated purity of isolated compounds is shown in Supporting Information S1: Figure 1.3. The isolated Compounds **1**–**6** were pooled as standards and analyzed under identical conditions to determine their RT and confirm their presence within the LJ‐M fraction.

As shown in Figure 6B, Compounds **1**–**6** were successfully separated with RTs as follows: apigenin (**5**) at 50.491 min, secohispanolone (**3**) at 62.377 min, nevadensin (**6**) at 64.179 min, galeopsin (**4**) at 79.309 min, (−)‐8*S*‐acetoxy‐15,16‐epoxy‐8,9‐seco‐13(16),14‐labdadiene (**1**) at 81.785 min, and hispanone (**2**) at 90.778 min. A spiking experiment was performed by adding the isolated standards to the LJ‐M fraction, confirming the specific chromatographic peaks.

Due to the limited amounts of Compounds **1**–**4** in the LJ‐M fraction and the presence of overlapping peaks within the mixture, only Compounds **5** and **6** were suitable for relative quantitative analysis. Following the spiking experiment, the yields of Compounds **5** and **6** were calculated to be approximately 29.59 and 42.69 mg/kg of LJ‐M fraction, respectively. Compounds **5** and **6** may serve as valuable chemical markers in the active fraction of this plant.

### 2.5 Anti‐Angiogenesis Activity

The partition fractions LJ‐B and LJ‐W exhibited a slight reduction in endothelial progenitor cell (EPC) growth. In contrast, LJ‐H and LJ‐M significantly decreased cell viability to 74% and 23%, respectively, after 48 h of treatment (Supporting Information S1: Table 3.1).

The isolated compounds were evaluated for their effects on the viability of EPC cells. Compounds **1**, **2**, **4**, and **5** inhibited EPC growth by over 50% at a concentration of 100 μM, with IC_50_ values of 68 ± 2 μM, 91 ± 1 μM, 61 ± 2 μM, and 35 ± 1 μM, respectively (Table 4), and the viability of EPC cells at 30 and 100 μM is shown in Supporting Information S1: Table 3.2.

**Table 4:** Anti‐angiogenic effects of compounds on human EPCs.

Compound	IC_50_ (μM)
**1**	68 ± 2
**2**	91 ± 1
**3**	> 100
**4**	61 ± 2
**5**	35 ± 1
**6**	> 100

**Figure 7:**
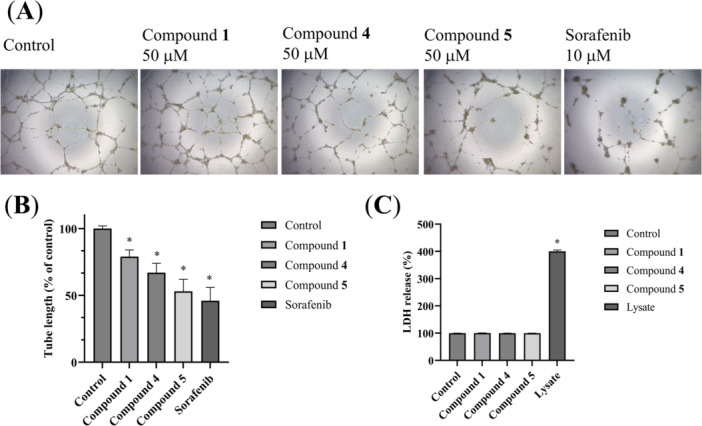
Effects of Compounds **1**, **4**, and **5** on tube formation and LDH release (cytotoxicity) of human EPCs. (A) EPCs were treated with Compounds **1**, **4**, and **5** (50 μM) and sorafenib (10 μM) for 24 h, and the formation of capillary‐like structures was assessed using the tube formation assay (*n* = 6). (B) Tube formation length was quantified by using ImageJ. (C) EPCs were treated with the specified compounds (50 μM) for 24 h, followed by cytotoxicity assessment using the LDH assay (*n* = 3). Results are expressed as the mean ± S.E.M. from at least three independent experiments. *p < 0.05 compared to the control group.

Further investigations included a tube formation assay and cytotoxicity assessment by lactate dehydrogenase (LDH) release in EPCs. In tube formation assay, at a 50 μM concentration, Compounds **1**, **4**, and **5**, along with positive control sorafenib, resulted in EPC tube lengths of 79 ± 5%, 67 ± 7%, 53 ± 19%, and 46 ± 10%, respectively, when compared to the negative control (100% tube length) (Figure 7 and Supporting Information S1: Table 4). It corresponded to tube length inhibition percentages of 21 ± 5%, 33 ± 7%, 47 ± 19%, and 54 ± 10%. These findings demonstrate that Compounds **1**, **4**, and **5** exhibit significant anti‐angiogenic inhibition, ranging from moderate to comparable with that of sorafenib.

The LDH assay confirmed no significant cytotoxicity, as evidenced by the lack of significant LDH release in EPCs treated with Compounds **1**, **4**, and **5**. These results collectively confirm that at a 50 µM concentration, these compounds are non‐toxic and safe, and their anti‐angiogenic effects are not attributable to cellular toxicity.

### 2.6 Anti‐Inflammatory Activity

The partition fractions LJ‐M and LJ‐B at 10 μg/mL inhibited superoxide anion generation by 95.14% and 81.72%, respectively, and elastase release by 100% and 50.16% inhibition of elastase release by human neutrophils in response to fMLF/CB (Table 5). The results indicated that LJ‐M suppressed the inflammatory response.

**Table 5:** Effects of crude fractions on superoxide anion generation and elastase release in fMLF/CB‐induced human neutrophils.

Sample	Superoxide anion inhibition %	Elastase release inhibition %
LJ‐H	57.01 ± 6.11***	95.36 ± 6.54***
LJ‐M	95.14 ± 1.91***	111.55 ± 4.25***
LJ‐B	81.72 ± 2.07***	50.16 ± 5.81***
LJ‐W	19.86 ± 2.71**	4.28 ± 4.52

*Note:* Percentage of inhibition (Inh %) at 10 μg/mL. Results are presented as mean ± S.E.M. (*n* = 3–4). *p < 0.05

** *p* < 0.01

*** *p* < 0.001 compared with the control (DMSO).

### 2.7 Molecular Docking

#### 2.7.1 Structure Validation of Target Receptor

Molecular docking was performed against receptor 4ASE [48], chosen for its high‐quality crystal structure resolution below 2.00 Å [49]. The structure was validated by Ramachandran plot and ERRAT (evaluation of Ramachandran plot and atomic density) analysis. The Ramachandran plot and ERRAT scores confirmed protein stability and accuracy. Validation results are provided in Supporting Information S1: Table 5 and Supporting Information S1: Figures 2 and 3. Detailed validation results are described in Supporting Information S1: Section 1.3.

The docking procedure was validated by redocking the native ligand AV‐951 (tivozanib). The root mean square deviation (RMSD) value of 1.31 Å (< 2 Å) ensures docking reliability [50]. A stacked view of crystallographic and redocked conformations (yellow vs. violet) revealed no significant structural changes (Supporting Information S1: Figure 4). Docking parameters are available in Supporting Information S1: Table 6.

#### 2.7.2 Molecular Docking

Following validation, the established parameters were applied to perform molecular docking of the isolated compounds and standard anti‐angiogenesis drugs to evaluate their binding affinities and identify potential VEGFR‐2 inhibitors. Additional details on thermodynamic parameters (Gibbs free energy [Δ*G*], inhibition constant [*K*_I_]) and interaction types are provided in Supporting Information S1: Section 1.4. All isolated compounds and selected standard drugs were analyzed in the in silico study.

To assess the impact of stereochemistry, two isomers of Compound **1** were evaluated: (−)‐8*S*‐acetoxy‐15,16‐epoxy‐8,9‐*seco*‐13(16),14‐labdadiene (**1a**, *S*‐isomer, isolated) and (−)‐8*R*‐acetoxy‐15,16‐epoxy‐8,9‐*seco*‐13(16),14‐labdadiene (**1b**, *R*‐isomer, modeled), and the only structural difference between the two isomers was at the C‐8 position (Supporting Information S1: Figure 5).

The docking results (Supporting Information S1: Table 7) showed the binding energies of the isolated compounds: **1b** (−9.58 kcal/mol), **3** (−9.35 kcal/mol), **1a** (−9.24 kcal/mol), **2** (−9.13 kcal/mol), **6** (−8.60 kcal/mol), **5** (−7.94 kcal/mol), and **4** (−7.20 kcal/mol). The binding energies of the standard drugs were as follows: axitinib (−11.52 kcal/mol), cabozantinib (−11.29 kcal/mol), lenvatinib (−11.60 kcal/mol), sunitinib (−9.52 kcal/mol), and sorafenib (−11.92 kcal/mol). The binding energy of native ligand‐tivozanib (−12.13 kcal/mol). Compounds **1b**, **3**, **1a**, and **2** displayed binding energies comparable to sunitinib. In contrast, Compounds **4** and **5** exhibited lower docking scores despite showing in vitro activity.

**Figure 8:**
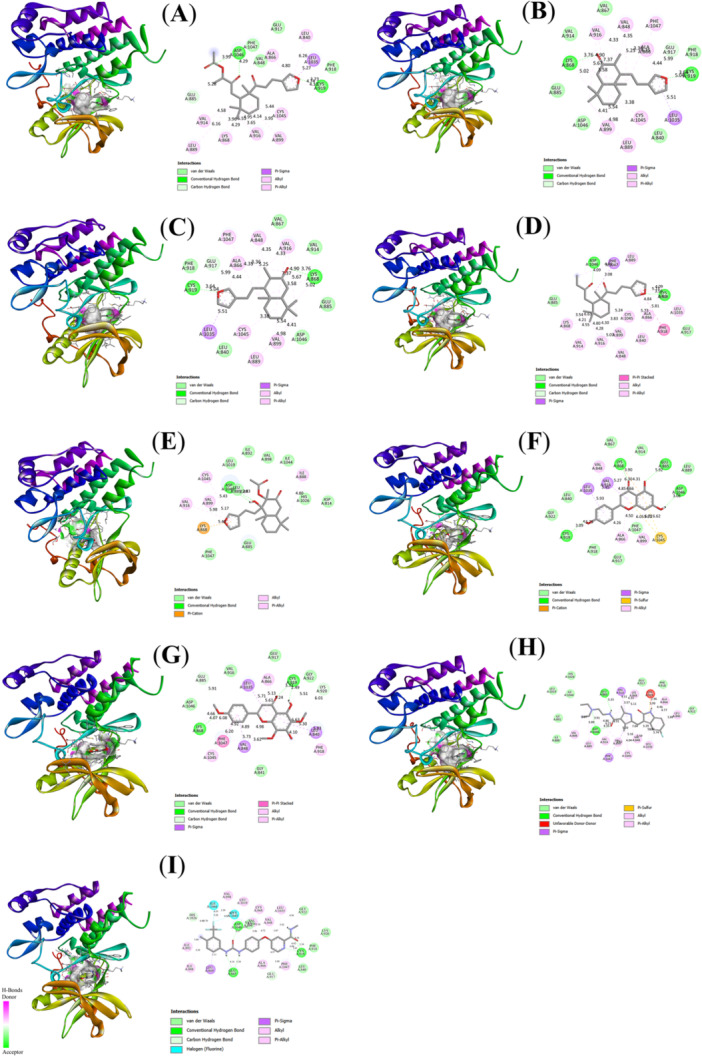
Molecular docking interactions of isolated compounds and sunitinib with VEGFR‐2. This figure presents the 2D and 3D molecular docking interactions of isolated Compounds (**1**–**6**) and the reference drug sunitinib with VEGFR‐2 (PDB ID: 4ASE). Panels (A–I) show the binding interactions of Compounds **1a**, **1b**, **2**, **3**, **4**, **5**, and **6**, respectively, within the VEGFR‐2 active site, while panels (H) and (I) display the binding interactions of sunitinib and sorafenib. Amino acid residues are indicated by their three‐letter abbreviations and numbers. An interaction key highlights the specific types of bonds and accessibility: dashed dark green lines represent classical hydrogen bonds; light green lines indicate carbon–hydrogen bonds; different shades of purple and pink denote pi‐interactions and other weak non‐covalent interactions; and a blue cloud illustrates solvent accessibility.

The USFDA‐approved drug sunitinib targets VEGFR, PDGFR, and c‐Kit; whereas, sorafenib targets VEGFR‐1, 2, and 3, PDGFR‐β, Flt‐3, c‐Kit, and RET. Sunitinib is a Type I ATP‐competitive VEGFR‐2 inhibitor that binds the kinase in its active DFG‐in (Asp‐Phe‐Gly active) conformation. It forms hydrogen‐bond interactions with the hinge‐region residues Glu917 and Cys919. In contrast, sorafenib is a Type II inhibitor that binds to both the ATP‐binding site and an adjacent hydrophobic site in its inactive DFG‐out (inactive) conformation, leading to improved selectivity and potency. Sorafenib forms hydrogen bonds with Asp1046, Cys919, and Glu885 [51, 52, 53]. The 2D and 3D docking interactions of the isolated compounds, sunitinib and sorafenib, are shown in Figure 8. Docking results of standard drugs axitinib, cabozantinib, lenvatinib, and tivozanib (native ligand) are provided in Supporting Information S1: Figure 6.

Hydrogen bond interactions were critical for stable binding within the VEGFR‐2 receptor. All tested compounds formed at least one hydrogen bond, contributing to their predicted binding affinity. Notably, Compound **5** showed hydrogen bond interactions similar to those of sorafenib, whereas the other compounds' interactions were similar to Type I inhibitor sunitinib. However, only Compounds **1a** and **5** showed interactions with both key residues. Based on the overall binding energy and interaction profiles, sunitinib and sorafenib were selected as a reference drug for further in silico analysis.

Flavonoids such as apigenin, quercetin, taxifolin, hesperidin, naringenin, epicatechin, kaempferol, and several other flavonoids were reported to possess anti‐cancer activity by VEGFR inhibition [40, 41, 42]. A previous study also predicted that polyphenols interact with the VEGF region responsible for VEGFR‐2 binding, indicating that inhibitory activities were strongly correlated with their binding affinities [42]. Collectively, these studies support the observed anti‐angiogenic potential of LJ‐M and isolated compounds.

#### 2.7.3 The VEGFR‐2 Inhibitory Effects Using Immunoblot Analysis

**Figure 9:**
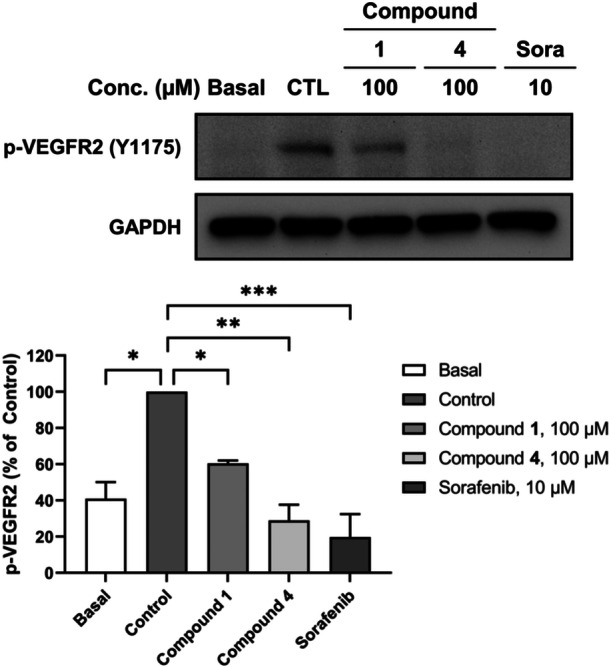
The p‐VEGFR‐2 inhibitory effects of Compounds **1** and **4**. Quiescent EPCs were treated with or without FBS in the absence (control) or presence of indicated compounds for 5–10 min. The phosphorylation of VEGFR‐2 at Tyr1775 was evaluated by immunoblotting. The relative levels of phosphorylated VEGFR‐2 were quantified using ImageJ software. Statistical significance compared to the control group (CTL) is indicated by asterisks: *p < 0.05, **p < 0.01, and ***p < 0.001.

Additional investigations were conducted to evaluate the effects of Compounds **1** and **4** on VEGFR‐2 phosphorylation using immunoblot analysis in EPCs. VEGFR‐2 kinase inhibition was assessed by evaluating phosphorylation of VEGFR‐2 at Tyr1175 in lysates. As shown in Figure 9, Compound **1** displayed a moderate inhibition on the phosphorylation of VEGFR‐2, while Compound **4** had a similar inhibitory effect on VEGFR‐2 phosphorylation to the positive control (sorafenib). These results revealed a promising effect of the labdane diterpenoids **1** and **4** on VEGFR‐2. Previous studies demonstrated Compound **5** inhibitory activity of angiogenesis in both cellular and animal models [54, 55, 56] suggesting that it is a well‐known angiogenesis inhibitor.

#### 2.7.4 Induction of Apoptosis in EPCs

**Figure 10:**
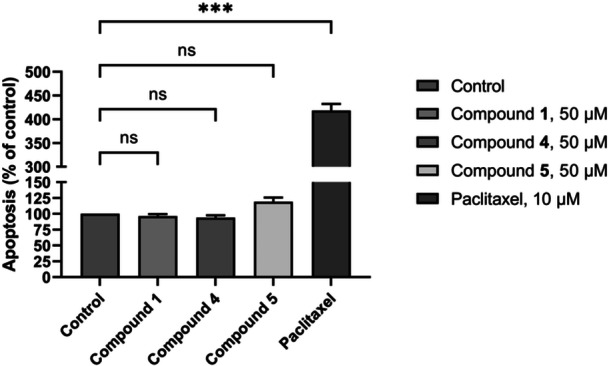
The effects of Compounds **1**, **4**, and **5** on apoptosis. EPCs were treated with the indicated compounds for 24 h. The total lysates were harvested, and the induction of apoptosis was determined by detecting the oligonucleosomal DNA fragmentation. Statistical significance compared to the control group (CTL) is indicated by asterisks: *p < 0.05, **p < 0.01, and ***p < 0.001.

To further examine whether the observed anti‐angiogenic activity was associated with apoptosis, EPCs were treated with Compounds **1**, **4**, and **5** and analyzed using the Cell Death Detection ELISA^PLUS^ assay. As shown in Figure 10, none of the tested compounds induced significant apoptotic cell death in EPCs. These results indicate that the anti‐angiogenic effects of Compounds **1**, **4**, and **5** are not mediated by apoptosis induction.

The experimental findings demonstrated consistency with the in silico predictions, confirming the potential of isolated compounds as VEGFR‐2 inhibitors.

### 2.8 Molecular Dynamics

Molecular dynamics (MD) simulation estimates atomic‐level interactions and the stability of biomolecular systems over time [57]. Hydrophobic regions of ligands tend to form stable interactions with proteins, enhancing binding stability. In contrast, hydrophilic regions exhibit weaker or nonspecific interactions, resulting in greater flexibility [58]. Cofactors, compound protonation, ions, ligand conformations, solvent entropy, and water molecules shape MD [59].

In this study, simulations were performed for 100 nanoseconds (ns) for isolated compounds and sunitinib. The results were validated using Ramachandran plots (Supporting Information S1: Table 8), which showed that more than 99.5% of residues were within allowed regions, ensuring structural integrity (no changes in conformational and dihedral angles). The diagrammatic representation of MD simulation results is shown in Supporting Information S1: Figure 7. Additionally, root mean square fluctuation (RMSF) and residue contact data are presented in Supporting Information S1: Figure 8. Intramolecular hydrogen bonds (IntraHB), radius of gyration (rGyr), molecular surface area (MoISA), solvent‐accessible surface area (SASA), and polar surface area (PSA) data are presented in Supporting Information S1: Figure 9.

#### 2.8.1 Protein‐Ligand Root Mean Square Deviation

**Figure 11:**
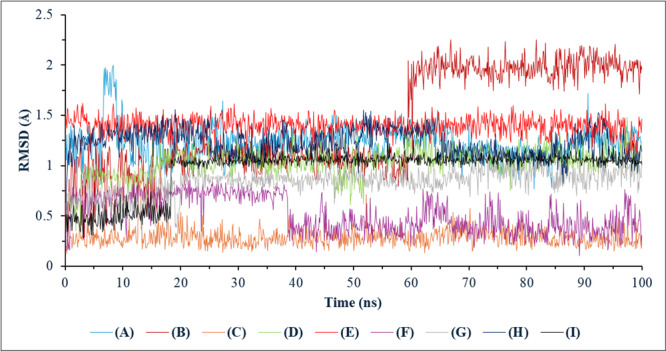
PL‐RMSD trajectories of isolated Compounds **1**–**6**, sunitinib, and sorafenib complexes with VEGFR‐2. This figure displays the PL‐RMSD trajectories for the VEGFR‐2 (PDB ID: 4ASE) complexes with isolated compounds (**1**–**6**) and the reference drug sunitinib. Each colored line represents a distinct complex trajectory: (A) light blue for Compound **1a**, (B) dark red for Compound **1b**, (C) orange for Compound **2**, (D) light green for Compound **3**, (E) red for Compound **4**, (F) purple for Compound **5**, (G) light gray for Compound **6**, (H) dark blue for sunitinib, and (I) black for sorafenib. The *X*‐axis represents time (ns), and the *Y*‐axis represents RMSD (Å).

The protein‐ligand root mean square deviation (PL‐RMSD) trajectory was used to assess the similarity and stability of protein‐ligand complexes. An RMSD value of < 3 Å indicates a stable protein backbone, with lower values reflecting greater conformational stability and reliability of the simulation [60, 61]. The average RMSD values of tested compounds are shown in Figure 11 and Supporting Information S1: Table 9: (A) **1a**‐4ASE (1.227 Å), (B) **1b**‐4ASE (1.397 Å), (C) **2**‐4ASE (0.285 Å), (D) **3**‐4ASE (1.029 Å), (E) **4**‐4ASE (1.394 Å), (F) **5**‐4ASE (0.518 Å), (G) **6**‐4ASE (0.842 Å), (H) sunitinib‐4ASE (1.221 Å), and (I) sorafenib‐4ASE (0.954 Å). All complexes were considered stable, with RMSD values < 2 Å. Compound **1b** showed fluctuations from 1 to 20 ns and 55 to 60 ns followed by stabilization, whereas other compounds exhibited no significant changes in their trajectory.

#### 2.8.2 RMSF

**Figure 12:**
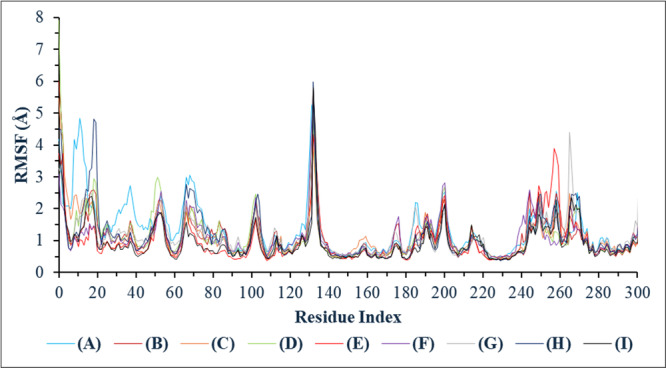
RMSF trajectories of isolated Compounds **1**–**6**, sunitinib, and sorafenib complexes with VEGFR‐2. This figure displays the RMSF trajectories for the VEGFR‐2 (PDB ID: 4ASE) complexes with isolated compounds (**1**–**6**) and the reference drug sunitinib. Each colored line represents a distinct complex trajectory: (A) light blue for Compound **1a**, (B) dark red for Compound **1b**, (C) orange for Compound **2**, (D) light green for Compound **3**, (E) red for Compound **4**, (F) purple for Compound **5**, (G) light gray for Compound **6**, (H) dark blue for sunitinib, and (I) black for sorafenib. The *X*‐axis represents residue index, and the *Y*‐axis represents RMSD (Å).

The RMSF trajectory was used to evaluate local flexibility, stability, and rigid regions of the protein structure. Higher RMSF values indicate flexible regions, while lower values reflect stable regions [62]. The average RMSF values of the tested compounds are presented in Figure 12 and Supporting Information S1: Table 10: (A) **1a**‐4ASE (1.392 Å), (B) **1b**‐4ASE (1.128 Å), (C) **2**‐4ASE (1.114 Å), (D) **3**‐4ASE (1.141 Å), (E) **4**‐4ASE (1.040 Å), (F) **5**‐4ASE (1.137 Å), (G) **6**‐4ASE (1.221 Å), (H) sunitinib‐4ASE (1.179 Å), and (I) sorafenib‐4ASE (0.958 Å). Significant fluctuations in the trajectory were observed in the loop regions residues at 12–20, 133–137, 189–196, 200–201, and 260–268 across all complexes. No substantial changes were detected in the C‐alpha and backbone regions of the protein complex (Supporting Information S1: Figure 8). These results indicate that the tested compounds did not significantly alter the overall protein structure.

#### 2.8.3 Protein‐Ligand Contact Analysis

**Figure 13:**
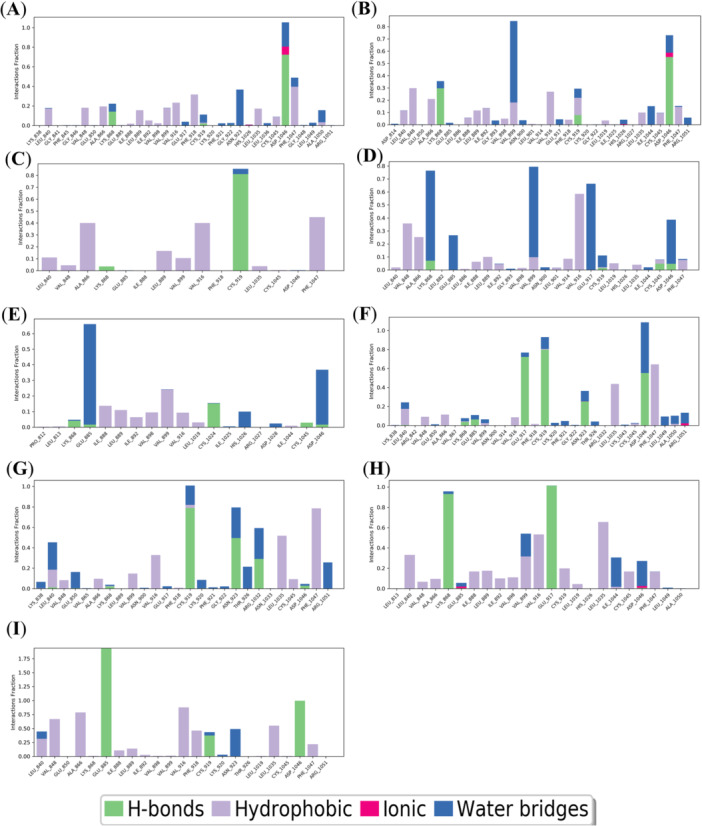
Protein‐ligand interaction fraction of isolated Compounds **1**–**6**, sunitinib, and sorafenib complexes with VEGFR‐2. This figure displays the protein and ligand interaction fraction for VEGFR‐2 (PDB ID: 4ASE) complexes with isolated compounds (**1**–**6**) and the reference drug sunitinib over a 100 ns MD simulation. Each panel represents a distinct complex: (A) Compound **1a**, (B) Compound **1b**, (C) Compound **2**, (D) Compound **3**, (E) Compound **4**, (F) Compound **5**, (G) Compound **6**, (H) sunitinib, and (I) sorafenib. The *X*‐axis represents the residue index, and the *Y*‐axis represents the interaction fraction.

Protein‐ligand contact analysis was performed to examine the interaction between the protein and ligand throughout the 100 ns MD simulations [63]. Figure 13 presents the histogram of these interactions, revealing that the compounds interacted with 15–33 residues of the 4ASE protein. This analysis included hydrogen bonds, hydrophobic interactions, ionic bonds, and water bridges. A taller graph in the histogram indicates more frequent and stable interactions. Notably, hydrogen bond interactions were consistently observed with the Cys919 and/or Asp1046 regions. Further details on ligand interaction properties are summarized in Supporting Information S1: Figure 9. This figure provides valuable insights into binding patterns and overall stability of the tested compounds within the 4ASE receptor binding site.

#### 2.8.4 The Trajectory Conformations

The RMSD and RMSF analyses collectively confirmed the stability of the protein structure throughout the simulation. To visualize atomic interactions between the compounds and the protein over time, 3D snapshots from the MD simulation were utilized [64]. All compounds demonstrated stable interactions, with average trajectory fluctuations < 2 Å. Compound **1b** stabilized after 60 ns. The positions of the compounds within the 4ASE at 20, 40, 60, 80, and 100 ns are shown in Supporting Information S1: Figure 10.

### 2.9 Lipinski's Rule of Five and Absorption, Distribution, Metabolism, Excretion, and Toxicity Parameters

To assess the drug‐like properties of isolated Compounds **1**–**6**, Lipinski's rule of five was applied, a widely recognized guideline for predicting oral bioavailability. The compounds were evaluated against the rule's criteria: molecular mass (≤ 500 Da), hydrogen bond donor (≤ 5), hydrogen bond acceptor (≤ 10), lipophilicity (≤ 5), and molar refractivity (130–40). As shown in Supporting Information S1: Table 11, all isolated compounds met these criteria, indicating good oral bioavailability.

Further absorption, distribution, metabolism, excretion, and toxicity (ADMET) analysis results (Supporting Information S1: Table 12) indicated that Compounds **1**–**6** possessed non‐toxic and safe profiles. Specifically, Compounds **1** and **4** were not predicted as P‐glycoprotein substrates, unlike Compound **5**. Compounds **4** and **5** were not predicted as CYP3A4 substrates, unlike Compound **1**. The intestinal absorption rates for Compounds **1** and **4** were 97.5% and 97.1%, respectively, while Compound **5** showed an absorption rate of 91.6%. Furthermore, Compounds **1**, **4**, and **5** exhibited good general toxicity profiles without predicted AMES mutagenicity, hERG inhibition, hepatotoxicity, and skin sensitization. Their predicted oral rat acute toxicity (LD_50_) values were 2.12, 2.98, and 2.04 mol/kg for Compounds **1**, **4**, and **5**, respectively.

## 3 Conclusion

This study successfully integrated DNA authentication, molecular networking, and bioactivity‐guided isolation to identify potential VEGFR‐2 inhibitors from *L. japonicus*. ITS region sequencing confirmed the plant's identity and distinguished it from *L. sibiricus*. Molecular networking facilitated the prioritization of compounds, resulting in the isolation of six phytochemicals, including an isolated stereoisomer of a labdane diterpenoid (Compound **1**) and the rare flavonoid nevadensin (Compound **6**). Compounds **1**, **4**, and **5** exhibited in vitro anti‐angiogenic activity. Compounds **1** and **4**, identified as novel anti‐angiogenic agents, showed tube length inhibition of 21% and 33%, respectively. Although their effects were less pronounced than those of the previously studied Compound **5** (47% inhibition) or the standard drug (54% inhibition), Compounds **1** and **4** represent promising leads for further investigation and structural optimization to enhance potency.

In silico analyses revealed that Compound **1** had strong binding affinities and interactions with VEGFR‐2, comparable to those of sunitinib, as supported by molecular docking and MD simulation. Additionally, Compounds **1** and **4** satisfied Lipinski's Rule of Five and showed favorable ADMET properties. These findings collectively support Compounds **1** and **4** as promising lead candidates for anti‐angiogenic therapy. Furthermore, in vivo studies are needed to validate its efficacy and pharmacokinetic profiles.

## 4 Experimental

### 4.1 General Experimental Procedures

Solvents were procured from Duksan Pure Chemicals (Gyeonggi, South Korea), HY Biocare Chemical Enterprise (New York, USA), and Merck (Merck KGaA, Darmstadt, Germany) and used for extraction and isolation by column chromatography, medium‐pressure liquid chromatography (MPLC), and high‐performance liquid chromatography with diode‐array detector (HPLC‐DAD). Thin‐layer chromatography (TLC) spots were visualized using 20% aqueous H_2_SO_4_ and heating on a hot plate. Sephadex LH‐20 gel (GE Healthcare, Sweden), Geduran Si 60 silica gel (63–200 and 40–63 µm), and C18 silica gel (25–40 µm) were obtained from Merck (Merck KGaA, Darmstadt, Germany). NMR spectra were recorded on a Varian‐Mercury‐plus 600 MHz FT‐NMR (Varian Corporation, CA, USA) and a JOEL JNM‐ECS 400 MHz NMR spectrometer (manufactured by JOEL Ltd., Tokyo, Japan). Electrospray‐ionization mass spectrometry (ESIMS) data were acquired using a Waters Micromass ZQ mass spectrometer (Waters Corporation, Milford, MA, USA). UV absorption spectra were recorded on a Jasco V‐770 spectrophotometer (JASCO Inc., Tokyo, Japan). Optical rotations were determined using a JASCO P‐2000 digital polarimeter (JASCO Corporation, Tokyo, Japan). ECD spectra were obtained using a Jasco J‐815 CD spectrometer (JASCO Inc., Tokyo, Japan). IR spectra were obtained using a Bruker Alpha spectrometer (Bruker, Germany). LC‐MS/MS analyses were performed using a Thermo Scientific Orbitrap Fusion Lumos equipped with an ESI source. Details on the instruments used for total DNA extraction, PCR amplification, and sequencing are outlined in Section 4.3. The LC‐MS/MS method is described in Section 4.5.

### 4.2 Plant Material

*L. japonicus* with white flowers (LJ) was provided by Tzu Chi University, Hualien, Taiwan, and was identified by Professor Shang‐Chih Lai. Dried aerial parts weighing 500 g were cut into pieces approximately 3 cm in length prior to extraction. The voucher specimen no. KMU‐LJ01 was stored in the herbarium of the Graduate Institute of Natural Products, Kaohsiung Medical University, Taiwan.

### 4.3 Isolation of Total DNA, PCR Amplification, and Sequencing

To confirm the genetic identity, total genomic DNA was extracted from dried aerial parts of *L. japonicus* using the DNeasy Plant Mini Kit (Qiagen, Germany), following the manufacturer's protocol with slight modifications as previously described [65]. The extracted DNA was then subjected to PCR amplification targeting specific genetic regions to ensure accurate identification. These regions included the *mat*K, *rbc*L, and the *trn*H‐*psb*A intergenic spacer from the chloroplast genome, as well as the ITS region of the nuclear genome. The primers used for PCR amplification were ITS1 (CCTTATCATTTAGAGGAAGGAG) [66] and ITS2 (TCCTCCGCTTATTGATATGC) [67] for the ITS region, matK‐1RKIM‐f (ACCCAGTCCATCTGGAAATCTTGGTTC) and matK‐3RKIM‐r (CGTACAGTACTTTTGTGTTTACGAG) for the matK region [68], rbcL1F (ATGTCACCACAAACAGAGACTAAAGC) and rbcL724R (GTAAAATCAAGTCCACCGCG) for the rbcL region [69], and trnH (GUG) (CGTAACAAGGTTTCCGTAGGTGAA) and psbA (GTTATGCATGAACGTAATGCTC) for the trnH‐psbA spacer [70].

The sequence with the highest percentage identity in the database was recorded to confirm the genetic identity of the *L. japonicus* samples. Due to the ongoing confusion between *L. japonicus* and *L. sibiricus* Linn. (細葉益母草Xì yè yìmǔcǎo) [1], the genetic divergence method utilizing Kimura 2‐parameter (K2P) distance matrices with 10,000 bootstrap replications was employed to analyze and differentiate these two species.

Detailed experimental procedures for DNA extraction quality assessment, PCR thermal cycling conditions, gel electrophoresis procedures, sequencing workflow, sequence editing and alignment, and BLAST analysis are provided in Supporting Information S2: Section 2.1.

### 4.4 Extraction and Partition

A total of 500 g of dried plant material was subjected to maceration using 99.8% methanol (20 L × 3) at room temperature overnight. The *L. japonicus* extract was filtered through Whatman Filter No. 1 and concentrated using a rotary evaporator, yielding 82.96 g (16.13% yield) of crude extract. The crude extract was suspended in deionized water (DI) (1 L) and partitioned three times with ethyl acetate (1 L each), resulting in two layers: LJ‐E (23.71 g) and LJ‐TW (55.86 g). The LJ‐E extract was further partitioned using 75% aqueous methanol (1 L) and *n*‐hexane (1 L) three times, yielding LJ‐H (12.13 g) and LJ‐M (11.58 g). Similarly, the LJ‐TW layer was dissolved in DI water (1 L) and partitioned with butanol (1 L) three times, to obtain LJ‐B (8.93 g) and LJ‐W (46.93 g). The partitioning scheme is shown in Supporting Information S1: Figure 1.

### 4.5 LC‐MS/MS Analysis

A UHPLC system (UltiMate 3000; Thermo Fisher Scientific, San Jose, USA) equipped with a binary pump (Model HPG‐3400RS), vacuum degasser (Model SRD‐3400), column oven (Model TCC‐3000RS), and autosampler (Model WPS‐3000TRS) coupled to an Orbitrap mass spectrometer (Thermo Scientific Orbitrap Fusion Lumos, USA) was used for LC‐MS/MS analysis. The chromatographic separation was achieved using a Water ACQUITY UPLC BEH C18 column (130 Å, 1.7 μm, 2.1 × 100 mm) at 40°C. The sample injection volume was 10 μL at a concentration of 500 μg/mL. The gradient elution consisted of water (A)/acetonitrile (B) (both with 0.1% formic acid [FA]) as follows: 0–1 min 5% B; 1–11 min 5% to 100% B; 11–14 min 100% B; 14.00–14.20 min 5% B, 14.20–17.00 min 5% B at a flow rate of 0.4 mL/min [71]. In positive ion mode, the electrospray ionization (ESI) mass spectrometer parameters were set as follows: capillary voltage at 3500 V; sheath gas arbitrary unit setting (Arb) at 35; sweep gas (Arb) at 1; ion transfer tube temperature at 300°C; vaporizer temperature at 300°C. LC‐MS/MS data acquisition was performed using Thermos Xcalibur software in Orbitrap Fusion Lumos. Centroid mode was used in MS with a resolution of 60,000 and spectra with mass range (*m/z*) from 100 to 1800.

### 4.6 Molecular Networking Construction

#### 4.6.1 LC‐MS/MS Data Pre‐Processing

Data pre‐processing was carried out following the approach described by Su et al. [72], with some modifications in the LC‐MS chromatogram construction parameter. Data pre‐processing was carried out following the approach described by Su et al. [72], with some modifications to the LC‐MS chromatogram construction parameters. After data processing, results were exported using the built‐in “Export/Submit to GNPS‐FBMN” (feature‐based molecular networking) function as.csv and.mgf files. Detailed parameter conditions were provided in Supporting Information S1: Section 2.2.

#### 4.6.2 GNPS Molecular Network

The molecular network was constructed using the GNPS‐FBMN web platform based on the pre‐processed LC‐MS/MS data (.mgf file). Parameter settings for network generation and visualization were carried out as described by Su et al. [72].

#### 4.6.3 Compound Annotation

The acquired mass spectra were compared against public mass spectra libraries on the GNPS website. Additionally, in silico tools, including SIRIUS 4, Network Annotation Propagation (NAP), and MS2LDA, were utilized for structure prediction and annotation of unidentified compounds as previously described.

### 4.7 Isolation

The LJ‐M fraction was selected for further isolation due to the presence of terpene signals in ^1^H NMR and terpene‐rich cluster identification in FBMN analysis using GNPS. The isolation procedure is shown in Supporting Information S1: Section 2.3. The mass and spectral data are shown as follows.

(−)‐8*S*‐Acetoxy‐15,16‐epoxy‐8,9‐*seco*‐13(16),14‐labdadiene‐7,9‐dione (**1**, 7 mg): White amorphous powder; $[{\alpha ]}_{D}^{27}$ −34.8 (c 0.11, CH_2_Cl_2_); UV (CH_2_Cl_2_) *λ*_max_ (log *ε*) 228.5 (0.62) nm; IR (CH_2_Cl_2_) *ν*_max_ 2927, 1731 (ester), 1695 (ketone), 1501 (furan), 1458, 1372 (alkyl bend), 1244, 1164, 1081, 1066, 1022 (C–O stretch) cm⁻¹; HRESIMS *m/z* 399.21390 [M + Na]^+^ (calculated for C_22_H_32_O_5_Na, 399.21420, Δ = −0.75 ppm). ^1^H (400 MHz, CDCl_3_) and ^13^C NMR (100 MHz, CDCl_3_) see Table 3; 1D and 2D NMR spectra see Supporting Information S1: Figures 11–19.

Hispanone (**2**, 2.7 mg): Colorless oil; $[{\alpha ]}_{D}^{25}$ +29.7 (c 0.60, CHCl_3_); UV (CH_2_Cl_2_) *λ*_max_ (log *ε*) 248 (0.83) nm; ESI‐MS *m/z* 323 [M + Na]^+^; molecular formula C_20_H_28_O_2_; ^1^H (600 MHz, CDCl_3_) and ^13^C NMR (150 MHz, CDCl_3_) see Supporting Information S1: Table 1; 1D and 2D NMR spectra see Supporting Information S1: Figures 20–28.

Secohispanolone (**3**, 1.2 mg): White amorphous powder; $[{\alpha ]}_{D}^{27}$ −7.4 (c 0.06, CHCl_3_); UV (CH_2_Cl_2_) *λ*_max_ (log *ε*) 229 (0.77), 285 (0.06) nm; ESI‐MS *m/z* 341 [M + Na]^+^; molecular formula C_20_H_30_O_3_; ^1^H (600 MHz, CDCl_3_) and ^13^C NMR (150 MHz, CDCl_3_) see Supporting Information S1: Table 1; 1D and 2D NMR spectra see Supporting Information S1: Figures 29–37.

Galeopsin (**4**, 5.0 mg): Colorless powder; $[{\alpha ]}_{D}^{27}$ +25.2 (c 0.25, CHCl_3_); UV (CH_2_Cl_2_) *λ*_max_ (log *ε*) 228.5 (0.56), 282.5 (0.09) nm; ESI‐MS *m/z* 399 [M + Na]^+^; molecular formula C_22_H_32_O_5_; ^1^H (400 MHz, CDCl_3_) and ^13^C NMR (100 MHz, CDCl_3_) see Supporting Information S1: Table 1; 1D and 2D NMR spectra see Supporting Information S1: Figures 38–46.

Apigenin (**5**, 6.5 mg): Yellow powder; UV (MeOH) *λ*_max_ (log *ε*) 218 (3.92), 343.5 (3.64), 268 (3.35) nm; ESI‐MS *m/z* 271 [M + H]^+^; molecular formula C_15_H_10_O_5_; ^1^H (400 MHz, C_5_D_5_N) and ^13^C NMR (100 MHz, C_5_D_5_N) see Supporting Information S1: Table 2; 1D and 2D NMR spectra see Supporting Information S1: Figures 47–51.

Nevadensin (**6**, 5.6 mg): Yellow powder; UV (MeOH) *λ*_max_ (log *ε*) 285.5 (0.99), 327.5 (0.99), 230 (0.88) nm; ESI‐MS *m/z* 345 [M + H]^+^; molecular formula C_18_H_16_O_7_; ^1^H (600 MHz, CDCl_3_) and ^13^C NMR (150 MHz, CDCl_3_) see Supporting Information S1: Table 2; 1D and 2D NMR spectra see Supporting Information S1: Figures 52–60.

### 4.8 HPLC Isolation and Profiling of Isolated Compounds

#### 4.8.1 HPLC Isolation of Chiral Compound **1**

HPLC isolation was carried out by using a Shimadzu Prominence LC (LC‐20A) (Shimadzu Corporation, Kyoto, Japan) equipped with pumps and a Shimadzu SPD‐M10Avp with a DAD. The chiral column (Phenomenex Lux 5μm, cellulose‐2, 4.6 × 250 mm) was used for isolation. Isolation was performed under the following conditions: Mobile phase A consisted of *n*‐hexane, while mobile phase B was isopropyl alcohol (IPA). The flow rate was maintained at 0.8 mL/min. The injection volume was 10 μL, and the UV (ultraviolet) detection wavelength at 254 nm. Initially, an isocratic condition of 10% B was maintained for 5 min, followed by a gradient increase to 100% over the next 55 min. A subsequent isocratic elution at 100% B was continued until 70 min, after which the system returned to 10% B from 70 to 80 min.

#### 4.8.2 HPLC Profiling

HPLC analysis was performed using a Shimadzu Prominence LC‐2030C 3D plus system (Shimadzu Inc., Kyoto, Japan) equipped with pumps and a PDA‐UV detector. A Shimadzu Shim‐pack GIST C18 column (5 μm, 4.6 × 250 mm) was used for the analysis. Separation was performed under the following conditions: Mobile Phase A consisted of DI H_2_O with 0.1% FA, and mobile phase B was methanol (MeOH) with 0.1% FA. The flow rate was set at 0.8 mL/min. The gradient condition began at 25% B, increasing to 100% over 110 min. Afterwards, an isocratic elution at 100% B for 10 min (110–120 min). The system then returned to 25% B from 120 to 130 min. The isolated pure compounds from the LJ‐M partition layer were used as standards. The sample injection volume was 10 μL, and detection was carried out at 230 and 210 nm using a UV detector. The isolated Compounds **1**–**6** were analyzed at a concentration of 0.5 mg/mL, and LJ‐M, the mother fraction from which Compounds **1**–**6** were isolated, was studied at a concentration of 2.5 mg/mL.

### 4.9 Anti‐Angiogenesis Activity

The effects of LJ partition fractions (LJ‐H, LJ‐M, LJ‐B, and LJ‐W) on the cell viability and anti‐angiogenic function of human CD34‐positive EPCs were assessed using the sulforhodamine B (SRB) assay and capillary tube formation assay, respectively, as previously described [73, 74]. Detailed protocols for EPCs isolation, culture, and assay conditions are provided in Supporting Information S1: Section 2.4.

### 4.10 Anti‐Inflammatory Activity

The anti‐inflammatory potential of LJ partition fractions was assessed by measuring the mitigation of reactive oxygen species (ROS) and elastase release in human neutrophils. Methodologies for human neutrophil preparation, ROS release measurement, elastase release, and LDH release assays were performed as previously described by Hwang et al. [75].

### 4.11 Molecular Docking and Dynamics

Molecular docking was performed with AutoDockTools (version 1.5.7) to evaluate the binding affinity of isolated compounds. The structure of VEGFR‐2 (PDB ID: 4ASE, resolution: 1.83 Å) was retrieved from the Protein Data Bank (PDB, https://www.rcsb.org/, accessed on October 16, 2024).

The stability of the protein‐ligand complexes was analyzed using MD simulations using Maestro (version 12.7, 2021) from Schrodinger. MD simulations were conducted for 100 ns using the transferable intermolecular potential 3‐point (TIP3P) water model. The system was maintained at a temperature of 300 K and pressure of 1.01325 bar using the Nose–Hoover and Martyna–Tobias–Klein algorithms in an isothermal–isobaric ensemble (NPT). Post‐simulation analysis was performed using RMSD, RMSF, principal component analysis (PCA), protein‐ligand contact analysis, and the simulation interaction diagram (SID) [50].

Detailed protocols for tools and materials, protein/receptor preparation, ligand preparation, molecular docking, interaction analysis, and MD simulation are provided in Supporting Information S1: Section 2.5.

### 4.12 Western Blot

Western blot was performed as previously described [76] with some modifications. Total proteins were isolated from EPCs subjected to the indicated conditions using RIPA buffer supplemented with a protease and phosphatase cocktail (Roche, Mannheim, Germany). The BCA assay (Thermo Fisher Scientific, MA, USA) was employed to quantify the protein concentration of each sample. Equal amounts of proteins (20 µg) were separated by SDS‐PAGE (sodium dodecyl sulfate‐polyacrylamide gel electrophoresis) and transferred onto PVDF (polyvinylidene difluoride) (Bio‐Rad, CA, USA) membranes. After blocking non‐specific binding sites on PVDF via a 1 h 5% BSA (bovine serum albumin) incubation at room temperature, PVDF membranes were then incubated overnight with primary antibodies against phospho‐VEGFR‐2 (Y1175) (Cat#2478, 1:1000) and GAPDH (Cat#2118, 1:3000) at 4°C. After washing the membrane with TBST (Tris‐buffered saline with 0.1% Tween 20), secondary HRP (horseradish peroxidase)–conjugated anti‐rabbit antibodies (#7074, 1:5000) and ECL (enhanced chemiluminescence) reagents (Thermo Fisher Scientific, MA, USA) were employed to detect and visualize the change in phosphorylation levels of VEGFR‐2 under a UVP imaging system (Analytik Jena, Jena, Germany). All antibodies were purchased from Cell Signaling Technology (MA, USA).

### 4.13 Apoptosis Detection

In total, 5 × 10^3^/well of EPCs were seeded in 96‐well plates. Following an overnight incubation, EPCs were subjected to the indicated conditions for 24 h. The Cell Death ELISA^PLUS^ kit was then used to measure the formation of histone‐associated DNA fragments, a sign of apoptosis‐associated DNA degradation, according to the manufacturer's instructions [76].

### 4.14 Statistical Analysis

Experiments were routinely conducted as three independent biological replications (*n* = 3). Data are presented as mean ± SEM. One‐way ANOVA (with Dunnett's test) was performed to evaluate whether the differences are statistically significant using GraphPad Prism (v. 9.5.1) for anti‐angiogenic data or Student's *t*‐test (Excel) for anti‐inflammatory data. Statistical significance was defined as *p*‐value *p < 0.05, **p < 0.01, and ***p < 0.001.

### 4.15 Drug‐Likeness and ADMET Predictions

The SMILES representation of each isolated compound was entered into prediction tools, and drug‐likeness was assessed based on the Lipinski Rule of Five [77, 78]. SwissADME [79] and pkCSM [80] were employed to evaluate the pharmacokinetic properties and ADMET properties of the compounds.

## Conflicts of Interest

The authors declare no conflicts of interest.

## Supporting information

Supporting File

## Data Availability

The data that support the findings of this study are available in the supporting material of this article.
