# Supplementary material for: Discovery of VEGFR‐2 Inhibitors From Leonurus japonicus via Integrated Genetic Authentication, Molecular Networking, and In Silico Analysis
Source: Arch Pharm (Weinheim). 2026 Aug 13;359(8):e70249. doi: 10.1002/ardp.70249 (PMC13470473; doi:10.1002/ardp.70249)
Supplement: Supplementary file 1 [file ARDP-359-e70249-s001.pdf]

# Supplementary Data

## Discovery of VEGFR-2 Inhibitors from *Leonurus japonicus* via Integrated Genetic Authentication, Molecular Networking, and *In Silico* Analysis

Thiyagarajan Raviraj[1,2], Chen-Lin Yu[3,4], Aekkhaluck Intharuksa[2], Shang-Chih Lai[5], Vidya Febrasca Tenderly[1], Yen Chi Loo[1], Pin-Wei Chen[1], Shih-Wei Wang[1,3,6], Kartiko Arif Purnomo[1,7,8], Soundar Rajan Kulandhaivel[1,9], Sedin Renadi[1], Stephen Lirio[10], Yuan-Bin Cheng[1,11], Yu-Liang Yang[12], Tsong-Long Hwang[8], Fang-Rong Chang[1,11,13,14,*], Michal Korinek[1,13,14,*]

[1]Graduate Institute of Natural Products, College of Pharmacy, Kaohsiung Medical University, Kaohsiung 80708, Taiwan. [2]Department of Pharmaceutical Sciences, Faculty of Pharmacy, Chiang Mai University, Chiang Mai, Thailand. [3]Institute of Biomedical Sciences, MacKay Medical University, New Taipei City 252005, Taiwan. [4]Department of Medical Research, Hsinchu MacKay Memorial Hospital, Hsinchu City 300044, Taiwan. [5]School of Post-Baccalaureate Chinese Medicine, Tzu Chi University, Hualien City, Hualien County, 970, Taiwan. [6]Department of Medicine, MacKay Medical University, New Taipei City, Taiwan. [7]Department of Plant Pathology and Microbiology, College of Bio-Resources and Agriculture, National Taiwan University, Taipei 106319, Taiwan. [8]Center for Drug Research and Development and Graduate Institute of Health Industry Technology, College of Human Ecology, Chang Gung University of Science and Technology, Taoyuan, 333324, Taiwan. [9]Department of Bioinformatics, Alagappa University, Karaikudi, Tamil Nadu, India - 630003. [10]Department of Chemistry, Chung Yuan Christian University, Taoyuan City 320, Taiwan. [11]Department of Marine Biotechnology and Resources, National Sun Yat-sen University, Kaohsiung 804201, Taiwan. [12]Agricultural Biotechnology Research Center, Academia Sinica, Taipei 115201, Taiwan. [13]Department of Medical Research, Kaohsiung Medical University Hospital, Kaohsiung Medical University, Kaohsiung 80708, Taiwan. [14]Drug Development and Value Creation Research Center, Kaohsiung Medical University, Kaohsiung 80708, Taiwan.

* Corresponding authors: Michal Korinek, Fang-Rong Chang, Graduate Institute of Natural Products, College of Pharmacy, Kaohsiung Medical University, Kaohsiung 80708, Taiwan.

E-mail addresses: michalk@kmu.edu.tw, tel: +886-7-3121101 ext. 2197 (Michal Korinek).
E-mail addresses: aaronfrc@kmu.edu.tw, tel: +886-7-3121101 ext. 2162 (Fang-Rong Chang).

## Table of Contents

S1. Supplemental results ........ 8

S1.1 Sequencing analysis ........ 8

Sequence S1.1.1 The nucleotide sequences of the four gene regions of the plant sample used in this study. ........ 8

Sequence S1.1.2 Comparison of ITS region between plant material and *Leonurus japonicus* ........ 10

Sequence S1.1.3. Comparison of ITS region between plant material and *Leonurus sibiricus* 11

S1.2. GNPS-FBMN ........ 12

Detailed annotation and classification of Labdane diterpenoids identified from LJ-M and compounds 1-6 Molecular Network. ........ 12

S1.3. Structure validation of target protein ........ 127

S1.4. Molecular Docking ........ 127

S2. Supplemental Methods ........ 128

S2.1. Isolation of Total DNA, PCR amplification and Sequencing ........ 128

S2.2. LC-MS/MS Data pre-processing parameters ........ 129

S2.3. Isolation method ........ 129

S2.4. Anti-angiogenesis activity ........ 130

S2.5. Molecular docking and dynamics ........ 131

S2.5.1. Tools and materials ........ 131

S2.5.2. Protein/ receptor preparation ........ 132

S2.5.3. Ligand preparation ........ 132

S2.5.4. Molecular docking and interaction analysis ........................................................ 133

S2.5.5. Molecular dynamic simulation ................................................................................ 133

Table S1. $^{1}H$ and $^{13}C$ NMR data of compounds 2 and 3 (600 and 150 MHz; $\delta$ in ppm, $J$ in Hz), compound 4 (400 and 100 MHz; $\delta$ in ppm, $J$ in Hz) in $CDCl_3$. ................................... 134

Table S2. $^{1}H$ and $^{13}C$ NMR data of compound 5 (400 and 100 MHz; $\delta$ in ppm, $J$ in Hz) in $C_5D_5N$ and compound 6 (600 and 150 MHz; $\delta$ in ppm, $J$ in Hz) in $CDCl_3$. ......................... 135

Table S3 Anti-angiogenesis activity ........................................................................................ 136

Table S3.1 Cytotoxicity effect of crude fractions on EPCs that may indicate anti-angiogenesis. ............................................................................................................. 136

Table S3.2 Cytotoxicity effect of isolated compounds on EPCs that may indicate anti-angiogenesis. ............................................................................................................. 136

Table S4. Effect of isolated compounds on tube formation and LDH release in EPCs after a 48-hour treatment period ................................................................................................ 137

Table S5. Receptor resolution. ................................................................................................ 137

Table S6. Molecular docking validation results. ................................................................... 137

Table S7. Molecular docking results of isolated compounds (1-6), standard drugs, and a native ligand with PDB ID: 4ASE. ........................................................................................ 137

Table S8. Amount and percentage of residue in favored, allowed, and disallowed regions of isolated compounds (1-6)-4ASE complex and sunitinib-4ASE complex ............................ 141

Table S9. Average, minimum, and maximum RMSD of isolated compounds (1-6) and sunitinib complexes with VEGFR2 (PDB ID: 4ASE). ......................................................... 141

Table S10. Average, minimum, and maximum RMSF of isolated compounds (1-6) and sunitinib complexes with VEGFR2 (PDB ID: 4ASE). ......................................................... 142

Table S11. Lipinski results of isolated compounds (1-6). ...................................................... 143

Table S12. ADMET parameters of isolated compounds (1-6). ............................................. 143

Figure S1. Extraction and partition scheme. ........ 146

Figure S1.1. HPLC isolation and chromatogram of chiral compound 1 ........ 146

Figure S1.2. LJ-M HPLC profiling chromatogram at 210 nm wavelength ........ 147

Figure S1.3. HPLC profiling and purity of isolated compounds ........ 147

Figure S1.3.1. HPLC profile of compound 1 ........ 148

Figure S1.3.2. HPLC profile compound 2 ........ 148

Figure S1.3.3. HPLC profile compound 3 ........ 149

Figure S1.3.4. HPLC profile compound 4 ........ 149

Figure S1.3.5. HPLC profile compound 5 ........ 150

Figure S1.3.6. HPLC profile compound 6 ........ 150

Figure S2. The Ramachandran plot of PDB ID: 4ASE. ........ 151

Figure S3. ERRAT result value of PDB ID: 4ASE. ........ 151

Figure S4. Stack view of the native ligand crystallographic structure and its re-docking structure. ........ 152

Figure S5. Showing key difference between compound 1a (generated) and 1b (isolated). ........ 152

Figure S6. Molecular docking interactions of standard drugs with VEGFR2. ........ 153

Figure S7. Ramachandran plot results of isolated compound, sunitinib and sorafenib complexes with VEGFR2. ........ 154

Figure S8. RMSF and residue contact analysis of isolated compound, sunitinib and sorafenib complexes with VEGFR2. ........ 155

Figure S9. Ligand interaction properties of isolated compounds sunitinib and sorafenib complexes with VEGFR2 during MD simulation. ........ 156

Figure S10. Conformational changes of isolated compounds and sunitinib complexes during molecular dynamics simulation. ....157

Figure S11. $^{1}$H NMR spectrum of compound 1....158

Figure S12. $^{13}$C NMR spectrum of compound 1....158

Figure S13. DEPT NMR spectrum of compound 1....159

Figure S14. COSY spectrum of compound 1....159

Figure S15. HSQC spectrum of compound 1....160

Figure S16. HMBC spectrum of compound 1....160

Figure S17. NOESY spectrum of compound 1....161

Figure S18. HRESI-MS spectrum of compound 1....161

Figure S19. UV and IR spectrum of compound 1....162

Figure S20. $^{1}$H NMR spectrum of compound 2....163

Figure S21. $^{13}$C NMR spectrum of compound 2....163

Figure S22. DEPT NMR spectrum of compound 2....164

Figure S23. COSY spectrum of compound 2....164

Figure S24. HMQC spectrum of compound 2....165

Figure S25. HMBC spectrum of compound 2....165

Figure S26. NOESY spectrum of compound 2....166

Figure S27. ESI-MS spectrum of compound 2....166

Figure S28. UV and IR spectrum of compound 2....167

Figure S29. $^{1}$H NMR spectrum of compound 3....168

Figure S30. $^{13}C$ NMR spectrum of compound 3 .......... 168
Figure S31. DEPT NMR spectrum of compound 3 .......... 169
Figure S32. COSY spectrum of compound 3 .......... 169
Figure S33. HSQC spectrum of compound 3 .......... 170
Figure S34. HMBC spectrum of compound 3 .......... 170
Figure S35. NOESY spectrum of compound 3 .......... 171
Figure S36. ESI-MS spectrum of compound 3 .......... 171
Figure S37. UV and IR spectrum of compound 3 .......... 172
Figure S38. $^{1}H$ NMR spectrum of compound 4 .......... 173
Figure S39. $^{13}C$ NMR spectrum of compound 4 .......... 173
Figure S40. DEPT NMR spectrum of compound 4 .......... 174
Figure S41. COSY spectrum of compound 4 .......... 174
Figure S42. HSQC spectrum of compound 4 .......... 175
Figure S43. HMBC spectrum of compound 4 .......... 175
Figure S44. ROESY spectrum of compound 4 .......... 176
Figure S45. ESI-MS spectrum of compound 4 .......... 176
Figure S46. UV and IR spectrum of compound 4 .......... 177
Figure S47. $^{1}H$ NMR spectrum of compound 5 .......... 178
Figure S48. $^{13}C$ NMR spectrum of compound 5 .......... 178
Figure S49. DEPT NMR spectrum of compound 5 .......... 179

Figure S50. ESI-MS spectrum of compound 5 ........ 179

Figure S51. UV and IR spectrum of compound 5 ........ 180

Figure S52. $^{1}$H NMR spectrum of compound 6 ........ 181

Figure S53. $^{13}$C NMR spectrum of compound 6 ........ 181

Figure S54. DEPT NMR spectrum of compound 6 ........ 182

Figure S55. COSY spectrum of compound 6 ........ 182

Figure S56. HSQC spectrum of compound 6 ........ 183

Figure S57. HMBC spectrum of compound 6 ........ 183

Figure S58. NOESY spectrum of compound 6 ........ 184

Figure S59. ESI-MS spectrum of compound 6 ........ 184

Figure S60. UV and IR spectrum of compound 6 ........ 185

References ........ 186

## Supplementary Data

### S1. Supplemental results

### S1.1 Sequencing analysis

**Sequence S1.1.1 The nucleotide sequences of the four gene regions of the plant sample used in this study.**

**ITS**

```
CTTATGATATGCTTAAACTCAGCGGGTGATCCCGCCTGACCTGGGGTCGCGGTCGTGGGCAC
GATGCTCGCGCCGTTGGGTTTCGCAATCGTTTCCTTGCCGACGGCGACGCGGGGCGCGACAG
CACGCGAGTTGAATCATCAACCACCACTGGTCGCGACGCGCGCCGACGGCGGATTCGCATTT
GGGCCGGCCGCGCGCGCTCGGGTCGCACGGGGGGCCAATCTCCGCCCCACCCCGCGGGGGAG
GGGGGCGACGCGATGCGTGACGCCCAGGCAGACGTGCCCTCGGCCTAATGGCTTCGGGCGCA
ACTTGCGTTCAAAGACTCGATGGTTCACGGGATTCTGCAATTCACACCAAGTATCGCATTTC
GCTACGTTCTTCATCGATGCGAGAGCCGAGATATCCGTTGCCGAGAGTCGTTTGGACATTCG
ATAGACGCCCCGGGCTCCCCCGCCGCCCCGCGGACGGGGCGTGGAGGGGGAGGGGAGCGCTA
CGTTGTGTATTCCTTGGCGCATTCCGCGCCCGAGTTTGTTAGCCCCATCGACGCGGCGCGGG
CGCGCGCGGCGACGGGGTTGGGGCCGCGGCGGGAGGTTGCCCGCCCCCCGCTGCCCCGGGGT
TGTTGGGAACTGGTTCGCGGTCTGCTTTGCAGGTTTCGACAATGATCCTTCCGCAGGTTCAC
CTACGGAAACCTTGTTACGACTTCTCCTTCCT
```

***mat*K**

```
TGAATCTTGGTTCAAATCCTTCGCTATTGGGTAAAAGATGCTTCCTCCTTGCATTTATTACG
AGTCTTTCTCAACGAATATTGTAATTGGAATAGTCTTCTTTTTCCAAAGAAAGTAAGTTCCA
CTTCTTCAAAAAAAAATAAAAGATTATTCTTATTCTTATATAATTCTCATGTATGTGAATAT
GAATCCATTTTCGTCTTTCTACGTAACCAATCTTTTCATTTACGATCAACATCTTCTGGAGT
TTTTCTTGAACGAATCTATTTCTATAGAAAAATAGAACGTCTTGTGAACGTCTTTGTTAAGA
TTAAAGATTTTGGGGCAAACCTGCGGTTGGTCAAGGAACCTTTCATGCATTATATTAGGTAT
CAAAAAAGATCCATTCTGGCTTCAAAAGGGACATTTCTTTTCATGAAGAAATGGAAATTTTA
CCTTGTCACTTTTTTGGCAATGGCATTTTTCGGTGTGGTTTCATCCAAGAAGCATTTATATAA
ACCAATTATCCAAGCATTCCCTTGAGTTTTTGGGCTATCTTTCAAGTGTGCGAATCAACCCT
TCCCTAGTACGCAGTCAAATTCTAGAAAATTCATTTCTAATCCATAATGCTATTAAGAAGTT
TGAGACTCTTGTTCCAATTATTCCTCTGATTGCGTCATTGGCTAAAGACAAATTTTGTAACG
TATTGGGGCATCCCGTTAGTAAGCCGATTCGGACTGATTTATCAGATTCTAATATTATTGAC
CGATTTACGCGTATATGCAGAAATATTTCTCGTTATCATAGTGGATCTTCCAAAAAAAAGAG
TTTGTATCGAATAAAGTATATACTTCGACTTTCTTGCGCTAGAACTTTGGCTCGTAACA
```

***rbc*L**

```
CAGAAACTAAAGCAAGTGTTGGATTCAAAGCGGGTGTTAAAGAGTACAAATTGACTTATTAT
ACCCCTGAATACGAAACCAAAGATACTGATATCTTGGCAGCATTCCGAGTAACTCCTCAACC
```

TGGAGTTCCACCCGAAGAAGCAGGGGCCGCGGTAGCTGCCGAATCTTCGACTGGTACATGGA
CAACTGTGTGGACCGATGGACTTACCAGCCTTGATCGTTACAAAGGGCGATGCTACCACATC
GAGCCTGTTCTTGGAGAAAAAGATCAATATATCTGTTATGTAGCTTACCCTTTAGACCTTTT
TGAAGAAGGTTCTGTTACTAACATGTTTACTTCCATTGTAGGAAATGTATTTGGATTCAAAG
CCCTACGTGCTCTACGTCTGGAAGATCTGCGAATCCCTACTGCTTATATTAAAACTTTCCAA
GGCCCACCTCATGGGATCCAAGTTGAGAGAGATAAATTGAACAAGTATGGTCGTCCTCTGTT
GGGATGTACTATTAAACCGAAATTGGGGTTATCTGCTAAAAACTATGGTAGAGCAGTTTATG
AATGTCTTCGCGGTGGACTTGATTTTACCAAAGATGATGAAAACGTGAACTCCCAGCCATTT
ATGCGTTGGAGAGATCGCTTCTTATTTTGTGCCGAAGCAATTTATAAAGCACAGGCTGAAAC
AGGTGAAATCAAAGGGCATTATTTGAATGCTACT

***trn*H-*psb*A**

CTAGACCTAGCTTCTATCGAAGCTCCAACAAATGGATAACACTTGGTCTTAGTGTATAGGAG
TTTTTGAAAATAGAATAGATAAATATAAGGAGCAATAACCCCTCTTGATAAAACAAGAAAGA
GTTAATTGCTCCTTAATTTTCTTTTCAATTACTTTTTTTCTTTCCATTACAGGATTCAGAAA
AAGAAAGAAGAAAAAAAAAATGATTAAAATTCAATTTATTTGGAATTTAAAAGTAAATTTATT
TTCATTATACTAATAATAGAGGGGCGGATGTAGCCAAGTGGATCAAGGCAGTGGATTGTGAA
TCCACCATGCGCGGGTTCAATTCCCGTCGTTCGCCCATGATTGGCTACAAAAGGATTTTTTT
TTAGTGAACGTATCACAGCTTACTCCTATTTTTTTTTAAAGACGAAGAAAGAAATTCTATTT
TCTCGCCTATTTACTACGGCGACGAACAATCAAATTTTGACTATATTTATTCCTTTTTCTAC
TTCTTCTTCCAAGTGCAGGATAACCCCAAGGGGTTGTGGGTTTTTTTCTACCAATTGGGGCT
CGCCCTTCACCACCCCCATGGGGATGGTCTACAGGGTTCATAACGACTCCTCTTACTACAGG
ACGCTTACCTAGCCAACGCTTGGATCCGGCTCTACCCAAACTTTTCTGGTTCGCCCCAACAT
TCCCCACTTGTCCGACTGTTGCTGAGCAGTTTTTGGATATCAAACGGACCTCCCCAGAAGGT
AATTTTAATGTGGCCGATTTCCCCTCTTTTGCAATCAGTTTCGCTACAGCACCCGCTGCTCT
AACTAATTGTCCACCCTTTCCAAGTGTGATTTCTATGTTATGTATGGCCGTGCCTAAGGGCA
TATCGGTTGAAGTAGATTCCTCTTTTTGATCAATCAAAACCCCTTCCCAAACTGTACAAGCT
TCTTCCAAAGCATACGGCTTTCTGGATGTAGATGATGATATCTATACAGACGGATCTTATAT
ATATGG

**Sequence S1.1.2 Comparison of ITS region between plant material and *Leonurus japonicus***

**Range 1: 3 to 700** Graphics ▼ Next Match ▲ P

| Score | Expect | Identities | Gaps | Strand |
|---|---|---|---|---|
| 1284 bits(695) | 0.0 | 697/698(99%) | 0/698(0%) | Plus/Minus |

```
Query  17   ACTCAGCGGGTGATCCCGCCTGACCTGGGGTCGCGGTCGTGGGCACGATGCTCGCGCCGT  76
            ||||||||||||||||||||||||||||||||||||||||||||||||||||||||||||
Sbjct  700  ACTCAGCGGGTGATCCCGCCTGACCTGGGGTCGCGGTCGTGGGCACGATGCTCGCGCCGT  641

Query  77   TGGGTTTCGCAATCGTTTCCTTGCCGACGGCGACGCGGGGCGCGACAGCACGCGAGTTGA  136
            ||||||||||||||||||||||||||||||||||||||||||||||||||||||||||||
Sbjct  640  TGGGTTTCGCAATCGTTTCCTTGCCGACGGCGACGCGGGGCGCGACAGCACGCGAGTTGA  581

Query  137  ATCATCAACCACCACTGGTCGCGACGCGCGCCGACGGCGGATTCGCATTTGGGCCGGCCG  196
            ||||||||||||||||||||||||||||||||||||||||||||||||||||||||||||
Sbjct  580  ATCATCAACCACCACTGGTCGCGACGCGCGCCGACGGCGGATTCGCATTTGGGCCGGCCG  521

Query  197  CGCGCGCTCGGGTCGCACGGGGGGCCAATCTCCGCCCCACCCCGCgggggagggggggCGA  256
            ||||||||||||||||||||||||||||||||||||||||||||||||||||||||||||
Sbjct  520  CGCGCGCTCGGGTCGCACGGGGGGCCAATCTCCGCCCCACCCCGCGGGGGAGGGGGGCGA  461

Query  257  CGCGATGCGTGACGCCCAGGCAGACGTGCCCTCGGCCTAATGGCTTCGGGCGCAACTTGC  316
            ||||||||||||||||||||||||||||||||||||||||||||||||||||||||||||
Sbjct  460  CGCGATGCGTGACGCCCAGGCAGACGTGCCCTCGGCCTAATGGCTTCGGGCGCAACTTGC  401

Query  317  GTTCAAAGACTCGATGGTTCACGGGATTCTGCAATTCACACCAAGTATCGCATTTCGCTA  376
            ||||||||||||||||||||||||||||||||||||||||||||||||||||||||||||
Sbjct  400  GTTCAAAGACTCGATGGTTCACGGGATTCTGCAATTCACACCAAGTATCGCATTTCGCTA  341

Query  377  CGTTCTTCATCGATGCGAGAGCCGAGATATCCGTTGCCGAGAGTCGTTTGGACATTCGAT  436
            ||||||||||||||||||||||||||||||||||||||||||||||||||||||||||||
Sbjct  340  CGTTCTTCATCGATGCGAGAGCCGAGATATCCGTTGCCGAGAGTCGTTTGGACATTCGAT  281

Query  437  AGACGCCCCGGGCTCCCCCGCCGCCCCGCGGACGGGGCGTGGAGGGGGAGGGGAGCGCTA  496
            ||||||||||||||||||||||||||||||||||||||||||||||||||||||||||||
Sbjct  280  AGACGCCCCGGGCTCCCCCGCCGCCCCGCGGACGGGGCGTGGAGGGGGAGGGGAGCGCTA  221

Query  497  CGTTGTGTATTCCTTGGCGCATTCCGCGCCCGAGTTTGTTAGCCCCATCGAcgcggcgcg  556
            ||||||||||||||||||||||||||||||||||||||||||||||||||||||||||||
Sbjct  220  CGTTGTGTATTCCTTGGCGCATTCCGCGCCCGAGTTTGTTAGCCCCATCGACGCGGCGCG  161

Query  557  ggcgcgcgcggcgacggggttggggccgcggcgggAGGTTGCCCGCCCCCCGCTGCCCCG  616
             |||||||||||||||||||||||||||||||||||||||||||||||||||||||||||
Sbjct  160  AGCGCGCGCGGCGACGGGGTTGGGGCCGCGGCGGGAGGTTGCCCGCCCCCCGCTGCCCCG  101

Query  617  GGGTTGTTGGGAACTGGTTCGCGGTCTGCTTTGCAGGTTTCGACAATGATCCTTCCGCAG  676
            ||||||||||||||||||||||||||||||||||||||||||||||||||||||||||||
Sbjct  100  GGGTTGTTGGGAACTGGTTCGCGGTCTGCTTTGCAGGTTTCGACAATGATCCTTCCGCAG  41

Query  677  GTTCACCTACGGAAACCTTGTTACGACTTCTCCTTCCT  714
            ||||||||||||||||||||||||||||||||||||||
Sbjct  40   GTTCACCTACGGAAACCTTGTTACGACTTCTCCTTCCT  3
```

**Sequence S1.1.3. Comparison of ITS region between plant material and *Leonurus sibiricus***

Sequence ID: **Query_5256797** Length: **683** Number of Matches: **1**

**Range 1: 1 to 682** Graphics ▼ Next Match ▲

| Score | Expect | Identities | Gaps | Strand |
|---|---|---|---|---|
| 1099 bits(595) | 0.0 | 655/683(96%) | 7/683(1%) | Plus/Minus |

```
Query  1     CTTA-TGATATGCTTAAACTCAGCGGGTGATCCCGCCTGACCTGGGGTCGCGGTCGTGGG  59
             |||| |||||||||||||||||||||||||||||||||||||||||||||||||||||||
Sbjct  682   CTTATTGATATGCTTAAACTCAGCGGGTGATCCCGCCTGACCTGGGGTCGCGGTCGTGGG  623

Query  60    CACGATGC----TCGCGCCGTTGGGTTTCGCAATCGTTTCCTTGCCGACGGCGACGCGGG  115
             ||||||||    ||||||||||||||||||  |||||||||  |||||||||||||||||
Sbjct  622   CACGATGCGTGCTCGCGCCGTTGGGTTTCGTGATCGTTTCCGGGCCGACGGCGACGCGGG  563

Query  116   GCGCGACAGCACGCGAGTTGAATCATCAACCACCACTGGTCGCGACGCGCGCCGACGGCG  175
              ||||||||||||||||||||||| |||||||||||||||||||||||||||||||||||
Sbjct  562   ACGCGACAGCACGCGAGTTGAATCGTCAACCACCACTGGTCGCGACGCGCGCCGACGGCG  503

Query  176   GATTCGCATTTGGGCCGGCCGCGCGCGCTCGGGTCGCACGGGGGGCCAATCTCCGCCCCA  235
             ||||||||||||||||||||||||||||    | |||||||| |||||||||||||||||
Sbjct  502   GATTCGCATTTGGGCCGGCCGCGCGCGCGATCG-CGCACGGGAGGCCAATCTCCGCCCCA  444

Query  236   CCCCGCgggggagggggCGACGCGATGCGTGACGCCCAGGCAGACGTGCCCTCGGCCTA  295
             ||||||||||||||||||||||||||||||||||||||||||||||||||||||||||||
Sbjct  443   CCCCGCGGGGGAGGGGGGCGACGCGATGCGTGACGCCCAGGCAGACGTGCCCTCGGCCTA  384

Query  296   ATGGCTTCGGGCGCAACTTGCGTTCAAAGACTCGATGGTTCACGGGATTCTGCAATTCAC  355
              |||||||||||||||||||||||||||||||||||||||||||||||||||||||||||
Sbjct  383   GTGGCTTCGGGCGCAACTTGCGTTCAAAGACTCGATGGTTCACGGGATTCTGCAATTCAC  324

Query  356   ACCAAGTATCGCATTTCGCTACGTTCTTCATCGATGCGAGAGCCGAGATATCCGTTGCCG  415
             ||||||||||||||||||||||||||||||||||||||||||||||||||||||||||||
Sbjct  323   ACCAAGTATCGCATTTCGCTACGTTCTTCATCGATGCGAGAGCCGAGATATCCGTTGCCG  264

Query  416   AGAGTCGTTTGGACATTCGATAGACGCCCCGGGCTCCCCCGCCGCCCCGCGGACGGGGCG  475
             |||||||||| ||||||||||||||| ||| |||||||||||||||||||||||||||||
Sbjct  263   AGAGTCGTTTTGACATTCGATAGACGTCCCCGGCTCCCCCGCCGCCCCGCGGACGGGGCG  204

Query  476   TGGAGGGGGAGGGGAGCGCTACGTTGTGTATTCCTTGGCGCATTCCGCGCCCGAGTTTGT  535
             ||||||||||||||||||| ||||||||||||||||||||||||||||||||||||||||
Sbjct  203   TGGAGGGGGAGGGGAGCGCCACGTTGTGTATTCCTTGGCGCATTCCGCGCCCGAGTTTGT  144

Query  536   TAGCCCCATCGAcgcggcgcgggcgcgcgcggcgacgggg-ttggggccgcggcgggAGG  594
             ||||||  |||||||||||||||||| ||||||||||||| |||||||||||||||||||
Sbjct  143   TAGCCCTGTCGACGCGGCGCGGGCGCACGCGGCGACGGGGGTTGGGGCCGCGGCGGGAGG  84

Query  595   TTGCCCGCCCCCCGCTGCCCCGGGGTTGTTGGGAACTGGTTCGCGGTCTGCTTTGCAGGT  654
             |||||||||||||||||||||||||||||| ||||| |||||||||||||||||||||||
Sbjct  83    TTGCCCGCCCCCCGCTGCCCCGGGGTTGTTCGGAACGGGTTCGCGGTCTGCTTTGCAGGT  24

Query  655   TTCGACAATGATCCTTCCGCAGG  677
             |||||||||||||||||||||||
Sbjct  23    TTCGACAATGATCCTTCCGCAGG  1
```

## S1.2. GNPS-FBMN

**Detailed annotation and classification of Labdane diterpenoids identified from LJ-M and compounds 1-6 Molecular Network.**

| **Labdane diterpenoids** | | | | | | | | | |
|---|---|---|---|---|---|---|---|---|---|
| S.NO | Cluster Index | parent mass | Retention Time in Minutes | SMILES | Compound IUPAC Name | Final classification | Confidence Score Exact | Sirius Score Normalized | Reference of derivative |
| 1 | 8822 | 444.2593 | 6.902 | CC(=O)OCC1(C2CCC3C(C2)(C1O)C4(C(C5C3(CO4)C(CCC5(C)C)O)O)O)O | (6,7,9,10,15-pentahydroxy-12,12-dimethyl-17-oxapentacyclo[7.6.2.15,8.01,11.02,8]octadecan-6-yl)methyl acetate | **Diterpenoid Diacetate** (Complex Polycyclic | 0.094 | 0.333 | [31] compound 71 |
| 2 | 20164 | 435.2733 | 10.485 | CC(=O)OCC12CCC3C(CCCC3(C1C(CC4(C2CC(=O)O4)C)OC(=O)C)C)(C)C | (10-acetyloxy-6,6,9a,11a-tetramethyl-2-oxo-3a,4,5,5a,7,8,9,9b,10,11-decahydro-3H-naphtho[2,1-e][1]benzofuran-3b-yl)methyl acetate | **Diterpenoid Diacetate** (Complex Polycyclic) | 0.257 | 0.5 | [31] compound 71 |

| | | | | | | | | | |
|---|---|---|---|---|---|---|---|---|---|
| 3 | 12049 | 354.2274 | 7.971 | C1CCOC(C1)OCC#CCC(CC#CCOC2CCCCO2)O | (12-Acetyloxy-5,8-dihydroxy-3,7,11-trimethyldodeca-2,6,10-trienyl) acetate | **Diterpenoid Diacetate** (Acyclic) | 0.026 | 0.017 | |
| 4 | 4177 | 471.2588 | 5.126 | CC(=O)OC1CC23CC(C(C2OC(=O)C)CCC3C(C4C1(C(C(C4O)O)(C)C)O)(C)O)(C)O | (16-Acetyloxy-4,6,7,9,14-pentahydroxy-5,5,9,14-tetramethyl-3-tetracyclo[11.2.1.01,10.04,8]hexadecanyl) acetate | **Diterpenoid Diacetate** (Tetracyclic, e.g., Grayanane-type) | 0.234 | 0.5 | |
| 5 | 10183 | 392.2431 | 7.342 | CC(C)C1=C(C2=C(C(=O)C1=O)C3(CCCC(C3CC2OC(=O)C)(C)C)C)O | (16-Acetyloxy-4,6,9,14-tetrahydroxy-5,5,9,14-tetramethyl-3-tetracyclo[11.2.1.01,10.04,8]hexadecanyl) acetate | **Diterpenoid Diacetate** (Tetracyclic, e.g., Grayanane-type) | 0.293 | 0.333 | |

| | | | | | | | | | |
|---|---|---|---|---|---|---|---|---|---|
| 6 | 15701 | 455.2638 | 9.215 | CC(=O)OC1CC23CC(C(C2OC(=O)C)CCC3C(C4C1(C(C(C4)O)(C)C)O)(C)O)(C)O | (16-Acetyloxy-4,6,9,14-tetrahydroxy-5,5,9,14-tetramethyl-3-tetracyclo[11.2.1.01,10.04,8]hexadecanyl) acetate | **Diterpenoid Diacetate** (Tetracyclic, e.g., Grayanane-type) | 0.262 | 0.149 | |
| 7 | 15937 | 455.2639 | 9.313 | CC(=O)OC1CC23CC(C(C2OC(=O)C)CCC3C(C4C1(C(C(C4)O)(C)C)O)(C)O)(C)O | (16-Acetyloxy-4,6,9,14-tetrahydroxy-5,5,9,14-tetramethyl-3-tetracyclo[11.2.1.01,10.04,8]hexadecanyl) acetate | **Diterpenoid Diacetate** (Tetracyclic, e.g., Grayanane-type) | 0.056 | 0.5 | |
| 8 | 11081 | 323.2217 | 7.701 | CC(=C)CCC(=CCCC(=CCCC(=CCO)C)CO)C(=O)O | (17-acetyl-4-hydroxy-10,13-dimethyl-3-oxo-2,6,7,8,9,11,12,14,15,16-decahydro-1H- | **Steroid Acetate** (derived from cholesterol | 0.043 | 0.5 | [31] compound 161 |

| | | | | | | | | | |
|---|---|---|---|---|---|---|---|---|---|
| | | | | | cyclopenta[a]phenanthren-17-yl) acetate | pathway, not isoprene units directly. C17-acetyl is characteristic of corticosteroids or progestogens. This is not a terpenoid.) | | | |
| 9 | 12042 | 458.2747 | 7.971 | CC1C=CC2=CC(CC(C2C1CCC(CC(CC(=O)O)O)O)OC(=O)C(C)C(C)O)O | (17-Acetyloxy-4,10,15-trihydroxy-5,5,10,15-tetramethyl-7-oxapentacyclo[12.2.1.01,11.04,9.06,8]heptadecan-3-yl) acetate | **Diterpenoid Diacetate** (Pentacyclic, often a modified | 0.102 | 0.328 | |

| | | | | | | | | | |
|---|---|---|---|---|---|---|---|---|---|
| | | | | | | kaurane or similar) | | | |
| 10 | 9204 | 393.2271 | 6.988 | CC(=O)OC1C(C2C(C3(C1C4(CCCC(C4CC3O)(C)C)C)C(=O)C2=C)O)O | (2,12,16-Trihydroxy-5,5,9-trimethyl-14-methylidene-15-oxo-11-tetracyclo[11.2.1.01,10.04,9]hexadecanyl) acetate | **Diterpenoid Acetate** (Tetracyclic, e.g., Grayanane-type) | 0.102 | 0.5 | |
| 11 | 8877 | 409.2218 | 6.91 | CC(=O)OC1CC(C2(C3CC(C4C(C3(C(CC2C1(C)C)O)C(=O)C4=C)O)O)C)O | (2,8,12,16-Tetrahydroxy-5,5,9-trimethyl-14-methylidene-15-oxo-6-tetracyclo[11.2.1.01,10.04,9]hexadecanyl) acetate | **Diterpenoid Acetate** (Tetracyclic, e.g., Grayanane-type) | 0.095 | 0.5 | |
| 12 | 10540 | 464.2643 | 7.496 | CC(=O)OCC(=O)C1(CCC2C1(CC(C3C2CCC4=CC(=O)CCC34C)OC(=O)C)C)O | (2,8,12,16-Tetrahydroxy-5,5,9-trimethyl-14-methylidene-15-oxo-6- | **Diterpenoid Acetate** (Tetracyclic, | 0.036 | 0.333 | |

| | | | | | | | | | |
|---|---|---|---|---|---|---|---|---|---|
| | | | | | tetracyclo[11.2.1.01,10.04,9]hexadecanyl) acetate | e.g., Grayanane-type) | | | |
| 13 | 10931 | 351.2166 | 7.617 | CC(CCCC(C=CC1C=CC(=O)C1CC=CCCCC(=O)O)O)O | (2,8,12,16-Tetrahydroxy-5,5,9-trimethyl-14-methylidene-15-oxo-6-tetracyclo[11.2.1.01,10.04,9]hexadecanyl) acetate | **Diterpenoid Acetate** (Tetracyclic, e.g., Grayanane-type) | 0.066 | 0.5 | |
| 14 | 17985 | 426.2486 | 9.917 | CC(=O)OC1CC(C2(C3CC(C4C(C3(C(CC2C1(C)C)O)C(=O)C4=C)O)O)C)O | (2,8,12,16-Tetrahydroxy-5,5,9-trimethyl-14-methylidene-15-oxo-6-tetracyclo[11.2.1.01,10.04,9]hexadecanyl) acetate | **Diterpenoid Acetate** (Tetracyclic, e.g., Grayanane-type) | 0.18 | 0.333 | |

| | | | | | | | | | |
|---|---|---|---|---|---|---|---|---|---|
| 15 | 19671 | 409.222 | 10.317 | CC(=O)OC1CC(C2(C3CC(C4C(C3(C(CC2C1(C)C)O)C(=O)C4=C)O)O)C)O | (2,8,12,16-Tetrahydroxy-5,5,9-trimethyl-14-methylidene-15-oxo-6-tetracyclo[11.2.1.01,10.04,9]hexadecanyl) acetate | **Diterpenoid Acetate** (Tetracyclic, e.g., Grayanane-type) | 0.152 | 0.5 | |
| 16 | 13766 | 495.2571 | 8.653 | CC(=O)OC1CC2CC3(C1C4(C(CC(C(C4C(C3OC(=O)C)O)(C)C)O)OC(=O)C)C)C(C2=C)O | (2,8-Diacetyloxy-3,6,15-trihydroxy-5,5,9-trimethyl-14-methylidene-11-tetracyclo[11.2.1.01,10.04,9]hexadecanyl) acetate | **Diterpenoid Triacetate** (Tetracyclic, e.g., Grayanane-type) | 0.159 | 0.424 | |
| 17 | 14093 | 512.2852 | 8.708 | CC(=O)OC1CC2CC3(C1C4(C(CC(C(C4C(C3OC(=O)C)O)(C)C)O)OC(=O)C)C)C(C2=C)O | (2,8-Diacetyloxy-3,6,15-trihydroxy-5,5,9-trimethyl-14-methylidene-11-tetracyclo[11.2.1.01,10.04,9]hexadecanyl) acetate | **Diterpenoid Triacetate** (Tetracyclic, e.g., | 0.253 | 0.33 | |

| | | | | | | | | | |
|---|---|---|---|---|---|---|---|---|---|
| | | | | | | Grayanane-type) | | | |
| 18 | 14094 | 517.2405 | 8.699 | CC(=O)OC1CC2CC3(C1C4(C(CC(C(C4C(C3OC(=O)C)O)(C)C)O)OC(=O)C)C)C(C2=C)O | (2,8-Diacetyloxy-3,6,15-trihydroxy-5,5,9-trimethyl-14-methylidene-11-tetracyclo[11.2.1.01,10.04,9]hexadecanyl) acetate | **Diterpenoid Triacetate** (Tetracyclic, e.g., Grayanane-type) | 0.143 | 0 | |
| 19 | 14164 | 495.2567 | 8.751 | CC(=O)OC1CC2CC3(C1C4(C(CC(C(C4C(C3OC(=O)C)O)(C)C)O)OC(=O)C)C)C(C2=C)O | (2,8-Diacetyloxy-3,6,15-trihydroxy-5,5,9-trimethyl-14-methylidene-11-tetracyclo[11.2.1.01,10.04,9]hexadecanyl) acetate | **Diterpenoid Triacetate** (Tetracyclic, e.g., Grayanane-type) | 0.118 | 0.497 | |
| 20 | 15173 | 512.285 | 9.056 | CC(=O)OC1CC2CC3(C1C4(C(CC(C(C4C(C3OC(=O)C)O)(C)C)O)OC(=O)C)C)C(C2=C)O | (2,8-Diacetyloxy-3,6,15-trihydroxy-5,5,9-trimethyl-14-methylidene-11- | **Diterpenoid Triacetate** (Tetracyclic, | 0.151 | 0.188 | |

| | | | | | | | | | |
|---|---|---|---|---|---|---|---|---|---|
| | | | | | tetracyclo[11.2.1.01,10.04,9]hexadecanyl) acetate | e.g., Grayanane-type) | | | |
| 21 | 14291 | 417.2265 | 8.772 | CC(=O)OC1C2C(C(CCC2(C3C(CC4CC3(C1OC(=O)C)C(=O)C4=C)O)C)O)(C)C | (2-Acetyloxy-6,11-dihydroxy-5,5,9-trimethyl-14-methylidene-15-oxo-3-tetracyclo[11.2.1.01,10.04,9]hexadecanyl) acetate | **Diterpenoid Diacetate** (Tetracyclic, e.g., Grayanane-type) | 0.082 | 0.5 | |
| 22 | 12136 | 456.2597 | 8.01 | | (2-Acetyloxy-6,8,15-trihydroxy-5,5,9-trimethyl-14-methylidene-11-tetracyclo[11.2.1.01,10.04,9]hexadecanyl) acetate | **Diterpenoid Diacetate** (Tetracyclic, e.g., Grayanane-type) | | | |

| | | | | | | | | | |
|---|---|---|---|---|---|---|---|---|---|
| 23 | 15340 | 437.2531 | 9.112 | CC(=O)OC1CC2CC3(C1C4(C(CC3OC(=O)C)C(C(CC4O)O)(C)C)C)C(C2=C)O | (2-Acetyloxy-6,8,15-trihydroxy-5,5,9-trimethyl-14-methylidene-11-tetracyclo[11.2.1.01,10.04,9]hexadecanyl) acetate | **Diterpenoid Diacetate** (Tetracyclic, e.g., Grayanane-type) | 0.128 | 0.5 | |
| 24 | 17973 | 454.2798 | 9.922 | CC(=O)OC1CC2CC3(C1C4(C(CC3OC(=O)C)C(C(CC4O)O)(C)C)C)C(C2=C)O | (2-Acetyloxy-6,8,15-trihydroxy-5,5,9-trimethyl-14-methylidene-11-tetracyclo[11.2.1.01,10.04,9]hexadecanyl) acetate | **Diterpenoid Diacetate** (Tetracyclic, e.g., Grayanane-type) | 0.084 | 0.333 | |
| 25 | 13821 | 442.2799 | 8.616 | CCC(=O)OC1C2CCC3C1(CC(C4(C(C3(C)O)C5C(C4(C)C)O5)O)O)CC2(C)O | (3,4,10,15-Tetrahydroxy-5,5,10,15-tetramethyl-7-oxapentacyclo[12.2.1.01,11.04,9.06,8]heptadecan-17-yl) propanoate | **Diterpenoid Propanoate** (Pentacyclic, often modified | 0.214 | 0.333 | |

| | | | | | | | | | |
|---|---|---|---|---|---|---|---|---|---|
| | | | | | | kaurane or similar) | | | |
| 26 | 11515 | 391.2107 | 7.819 | CC(C)C1=C(C2=C(C(=O)C1=O)C3(CCCC(C3CC2OC(=O)C)(C)C)CO)O | (4,13-Dihydroxy-15-methoxy-2,6,14,17-tetramethyl-3,11-dioxo-10-oxatetracyclo[7.7.1.02,7.013,17]heptadecan-16-yl) acetate | **Diterpenoid Acetate** (Tetracyclic) | 0.37 | 0.5 | |
| 27 | 11251 | 317.2111 | 7.772 | CC1C(=O)CC2C(C(CCC2(C1(CCC3=COC=C3)O)C)O)(C)C | (4,7,9-Trihydroxy-12,12-dimethyl-6-methylidene-17-oxapentacyclo[7.6.2.15,8.01,11.02,8]octadecan-10-yl) acetate | **Diterpenoid Acetate** (Pentacyclic, often a rearranged type like Taxane-type or similar) | 0.335 | 0.5 | |

| | | | | | | | | | |
|---|---|---|---|---|---|---|---|---|---|
| 28 | 15946 | 423.2377 | 9.32 | CC1C2C(CC3(C1=CC(=O)O3)OC)C4(CCCC(C4(C(C2OC(=O)C)O)O)(C)C)C | (4a,5-dihydroxy-10a-methoxy-4,4,7,11b-tetramethyl-9-oxo-2,3,5,6,6a,7,11,11a-octahydro-1H-naphtho[2,1-f][1]benzofuran-6-yl) acetate | **Diterpenoid Acetate** (Polycyclic, Naphthobenzofuran derivative) | 0.177 | 0.5 | |
| 29 | 13556 | 349.2009 | 8.549 | CC(=CCCC(=CCCC(=C)C(=O)CCC(=CCO)C(=O)O)CO)CO | (5,9,9,13-Tetramethyl-22-oxo-19,23,24-trioxahexacyclo[16.5.1.01,20.04,17.05,14.08,13]tetracosan-2-yl) acetate | **Sesterterpenoid Acetate** (Hexacyclic, highly oxygenated) | 0.058 | 0.5 | |
| 30 | 8878 | 427.2329 | 6.906 | CC(=O)OCC1(C2CCC3C(C2)(C1O)C4(C(C5C3(CO4)C(CCC5(C)C)O)O)O)O | (6,7,9,10,15-Pentahydroxy-12,12-dimethyl-17-oxapentacyclo[7.6.2.15,8.01,11.02,8]octadecan-6-yl)methyl acetate | **Diterpenoid Acetate** (Pentacyclic, often rearranged | 0.06 | 0.5 | |

| | | | | | | | | | |
|---|---|---|---|---|---|---|---|---|---|
| | | | | | | like Taxane-type or similar) | | | |
| 31 | 10472 | 409.222 | 7.45 | CC(=O)OC1CC(C2(C3CC(C4C(C3(C(CC2C1(C)C)O)C(=O)C4=C)O)O)C)O | (6,7,9,10,15-Pentahydroxy-12,12-dimethyl-17-oxapentacyclo[7.6.2.15,8.01,11.02,8]octadecan-6-yl)methyl acetate | **Diterpenoid Acetate** (Pentacyclic, often rearranged like Taxane-type or similar) | 0.219 | 0.5 | |
| 32 | 9201 | 409.2219 | 7.006 | CC(=O)OCC1(C2CCC3C45CCCC(C4C(C(C3(C2O)C1O)(OC5)O)O)(C)C)O | (6,7,9,10,18-Pentahydroxy-12,12-dimethyl-17-oxapentacyclo[7.6.2.15,8.01,11.02,8]octadecan-6-yl)methyl acetate | **Diterpenoid Acetate** (Pentacyclic, often rearranged like Taxane- | 0.092 | 0.5 | |

| | | | | | | | | | |
|---|---|---|---|---|---|---|---|---|---|
| | | | | | | type or similar) | | | |
| 33 | 15452 | 459.2355 | 9.134 | CC(=O)OC1CC2C(C(CC(C2(C3C14CC(CC3O)C(=C)C4O)C)O)OC(=O)C)(C)C | (6-Acetyloxy-8,11,15-trihydroxy-5,5,9-trimethyl-14-methylidene-2-tetracyclo[11.2.1.01,10.04,9]hexadecanyl) acetate | **Diterpenoid Diacetate** (Tetracyclic, e.g., Grayanane-type) | 0.047 | 0.951 | |
| 34 | 10914 | 349.2009 | 7.619 | CC(=CCCC1=CCOC1=O)CCC=C(CCC=C(C)CO)C(=O)O | (8-Acetyloxy-6,11,15-trihydroxy-5,5,9-trimethyl-14-methylidene-2-tetracyclo[11.2.1.01,10.04,9]hexadecanyl) acetate | **Diterpenoid Diacetate** (Tetracyclic, e.g., Grayanane-type) | 0.053 | 0.5 | |
| 35 | 13657 | 313.1798 | 8.617 | CC1CCC(=C)C(=CC=CCC1(C)CCC2=CC(=O)OC2)C(=O)O | (8-Acetyloxy-6,11,15-trihydroxy-5,5,9-trimethyl-14-methylidene-2- | **Diterpenoid Diacetate** (Tetracyclic, | 0.076 | 0.5 | |

| | | | | | | | | | |
|---|---|---|---|---|---|---|---|---|---|
| | | | | | tetracyclo[11.2.1.01,10.04,9]hexadecanyl) acetate | e.g., Grayanane-type) | | | |
| 36 | 15207 | 454.28 | 9.058 | CC(=O)OC1CC2C(C(CC(C2(C3C14CC(CC3O)C(=C)C4O)C)OC(=O)C)O)(C)C | (8-Acetyloxy-6,11,15-trihydroxy-5,5,9-trimethyl-14-methylidene-2-tetracyclo[11.2.1.01,10.04,9]hexadecanyl) acetate | **Diterpenoid Diacetate** (Tetracyclic, e.g., Grayanane-type) | 0.137 | 0.333 | |
| 37 | 15256 | 459.2356 | 9.129 | CC(=O)OC1CC2C(C(CC(C2(C3C14CC(CC3O)C(=C)C4O)C)OC(=O)C)O)(C)C | (8-Acetyloxy-6,11,15-trihydroxy-5,5,9-trimethyl-14-methylidene-2-tetracyclo[11.2.1.01,10.04,9]hexadecanyl) acetate | **Diterpenoid Diacetate** (Tetracyclic, e.g., Grayanane-type) | 0.109 | 0.626 | |

| | | | | | | | | | |
|---|---|---|---|---|---|---|---|---|---|
| 40 | 15451 | 454.2799 | 9.135 | CC(=O)OC1CC2C(C(CC(C2(C3C14CC(CC3O)C(=C)C4O)C)OC(=O)C)O)(C)C | (8-Acetyloxy-6,11,15-trihydroxy-5,5,9-trimethyl-14-methylidene-2-tetracyclo[11.2.1.01,10.04,9]hexadecanyl) acetate | **Diterpenoid Diacetate** (Tetracyclic, e.g., Grayanane-type) | 0.074 | 0.333 | |
| 41 | 11687 | 299.2005 | 7.842 | CC1(CCC2=C(C1=O)CCC3C2(CCCC3(C)C(=O)O)C)C=C | (8-Hydroperoxy-5,13-dimethyl-9,16-dimethylidene-15-oxo-4,14-dioxatricyclo[11.3.2.03,5]octadecan-12-yl) acetate | **Diterpenoid Acetate** (Tricyclic, highly oxidized with hydroperoxy/epoxide) | 0.314 | 0.5 | |
| 42 | 13782 | 470.3111 | 8.596 | CC(=O)OC1C2CCC3C1(CC4C5(C(C3(C)O)CC(C5(C)C)O)OC(O4)(C)C)CC2(C)O | (9,12,17-Trihydroxy-5,5,8,8,12,17-hexamethyl-4,6- | **Sesterterpenoid Acetate** | 0.284 | 0.333 | |

| | | | | | | | | | |
|---|---|---|---|---|---|---|---|---|---|
| | | | | | dioxapentacyclo[14.2.1.01,13.03,7.07,11]nonadecan-19-yl) acetate | (Pentacyclic, highly oxygenated) | | | |
| 43 | 10572 | 392.2432 | 7.513 | CC(C)C1=C(C2=C(C(=O)C1=O)C3(CCCC(C3CC2OC(=O)C)(C)C)C)O | [2-(11-acetyloxy-17-hydroxy-10,13-dimethyl-3-oxo-2,6,7,8,9,11,12,14,15,16-decahydro-1H-cyclopenta[a]phenanthren-17-yl)-2-oxoethyl] acetate | **Steroid Diacetate** (Not a terpenoid) | 0.368 | 0.333 | [31] compound 164 |
| 44 | 20107 | 540.3893 | 10.493 | CCCCCCCCCCCCCCOCCCOC(=O)NCC(=O)OCC(C)(C)OCC(C)(C)O | [2-(2-Hydroxy-2-methylpropoxy)-2-methylpropyl] 2-(3-tetradecoxypropoxycarbonylamino)acetate | **Fatty Acid Derivative/ Complex Lipid** (Not a terpenoid) | 0.014 | 0.391 | |
| 45 | 11730 | 759.4295 | 7.893 | CCOCC(CC1C(C2CC(=O)CC3CCC4C(O3)C5C6C(O4)C7C(O6)CC(O7) | [3a-Acetyloxy-3-(4,4,7a-trimethyl-1,2,3,3a,5,6,7,7b-octahydrocyclopropa[a]naphthale | **Diterpenoid Diacetate** (Polycyclic, | 0.034 | 0 | |

| | | | | | | | | | |
|---|---|---|---|---|---|---|---|---|---|
| | | | | (O5)CCC8CC(=C)C(O8)CCC9CC(C(=C)C(O9)CC2O1)C)OC)O | n-1a-yl)-5-oxo-2,3,4,6a-tetrahydrofuro[2,3-b]furan-2-yl] acetate | Cyclopropanaphthalene derivative) | | | |
| 46 | 20779 | 468.3318 | 10.686 | CC(=O)OC1CC(C(C2(C1C(CCC2)(C)C)C)CCC(C)(C=C)OC(=O)C)(C)OC(=O)C | [3-acetyloxy-4-(3-acetyloxy-3-methylpent-4-enyl)-3,4a,8,8-tetramethyl-2,4,5,6,7,8a-hexahydro-1H-naphthalen-1-yl] acetate | **Diterpenoid Diacetate** (Bicyclic, e.g., Labdane-type derivative) | 0.556 | 0.333 | |
| 47 | 8450 | 394.2223 | 6.747 | CC(=CC=CC(=CCC(C(=COC(=O)C)C=COC(=O)C)OC(=O)C)C)C | [4-Acetyloxy-3-(acetyloxymethylidene)-7,11-dimethyldodeca-1,6,8,10-tetraenyl] acetate | **Diterpenoid Diacetate** (Acyclic) | 0.028 | 0.333 | |

| | | | | | | | | | |
|---|---|---|---|---|---|---|---|---|---|
| 48 | 11670 | 412.2692 | 7.852 | CCCCCCCCCCCC(CC1=C(C(=O)C=C(C1=O)OC)O)OC(=O)C | [4-Acetyloxy-5,5,8a-trimethyl-1-(3-oxobut-1-enyl)-1,4,4a,6,7,8-hexahydronaphthalen-2-yl]methyl acetate | **Diterpenoid Acetate** (Bicyclic, e.g., Labdane-type derivative) | 0.174 | 0.333 | [31] compound 110 |
| 49 | 11569 | 458.2746 | 7.833 | CC1C=CC2=CC(CC(C2C1CCC(CC(CC(=O)O)O)O)OC(=O)C(C)C(C)O)O | [5-(1-Acetyloxytridecyl)-5-hydroxy-4-oxocyclopent-2-en-1-yl] acetate | **Fatty Acid Derivative/ Cyclopentenone Lipid** (Not a terpenoid) | 0.111 | 0.333 | |
| 50 | 14285 | 466.2798 | 8.768 | CC1CC(=O)C2C3C1C2(C(CC(C3(C)C)OC(=O)CC=C(C)O)OC(=O)CC=C(C)O)C | [6-(4-Hydroxypent-3-enoyloxy)-3,3,7,9-tetramethyl-11-oxo-4-tricyclo[5.4.0.02,8]undecanyl] 4-hydroxypent-3-enoate | **Sesquiterpenoid Ester** (Tricyclic core, with | 0.309 | 0.333 | |

| | | | | | | | | | |
|---|---|---|---|---|---|---|---|---|---|
| | | | | | | C5 acyl side chains) | | | |
| 51 | 5143 | 456.2956 | 5.509 | CC(=CCCC(=CCCC(=CCCC(=CCO)CO)COC(=O)C)COC(=O)C)CO | [6-(Acetyloxymethyl)-12-hydroxy-10-(hydroxymethyl)-2-(5-hydroxy-4-methylpent-3-enyl)dodeca-2,6,10-trienyl] acetate | **Diterpenoid Diacetate** (Acyclic) | 0.046 | 0.333 | |
| 52 | 13423 | 440.2642 | 8.434 | CC(=O)OC1CC2CC3(C1C45CCCC(C4C(C3(OC5)O)O)(C)C)C(=O)C2COC | [6-(Acetyloxymethyl)-12-hydroxy-10-(hydroxymethyl)-2-(5-hydroxy-4-methylpent-3-enyl)dodeca-2,6,10-trienyl] acetate | **Diterpenoid Diacetate (Acyclic)** | 0.165 | 0.333 | |
| 53 | 13629 | 465.2483 | 8.539 | C1CCC(C1)OC(=O)C(C(=O)OC2CCCC2)(C(=O)OC3CCCC3)C(=O)OC4CCCC4 | [6,10a-Dihydroxy-4-(hydroxymethyl)-4,7,11b-trimethyl-9-oxo-1,2,3,4a,5,6,6a,7,11,11a- | Polycyclic Diterpenoid Acetate | 0.058 | 0.5 | [31] Compound 69 |

| | | | | | | | | | |
|---|---|---|---|---|---|---|---|---|---|
| | | | | | decahydronaphtho[2,1-f][1]benzofuran-5-yl] acetate | | | | |
| 54 | 17968 | 409.222 | 9.917 | CC1C2C(CC3(C1=CC(=O)O3)O)C4(CCCC(C4C(C2O)OC(=O)C)(C)CO)C | [6,10a-Dihydroxy-4-(hydroxymethyl)-4,7,11b-trimethyl-9-oxo-1,2,3,4a,5,6,6a,7,11,11a-decahydronaphtho[2,1-f][1]benzofuran-5-yl] acetate | **Iridoid Glycoside Derivative** (Not a terpenoid) | 0.123 | 0.5 | [31] Compound 69 |
| 55 | 13168 | 313.1798 | 8.365 | | [7-(Hydroxymethyl)-3,5-dimethoxy-1-(2-methylbutanoyloxy)-1,3,5,7a-tetrahydrocyclopenta[c]pyran-4-yl]methyl 2-methylbutanoate | **Iridoid Glycoside Derivative** (Not a terpenoid) | | | [31] compound 111 |
| 56 | 13405 | 335.2216 | 8.457 | CC(=CCCC(=CCCC(=CCCC(=CC(=O)O)C(=O)O)C)C)C | [7-(Hydroxymethyl)-3,5-dimethoxy-1-(2-methylbutanoyloxy)-1,3,5,7a- | **Iridoid Glycoside Derivative** | 0.055 | 0.5 | [31] compound 111 |

| | | | | | | | | | |
|---|---|---|---|---|---|---|---|---|---|
| | | | | | tetrahydrocyclopenta[c]pyran-4-yl]methyl 2-methylbutanoate | (Not a terpenoid) | | | |
| 57 | 12237 | 441.2482 | 8.055 | | [7-hydroxy-5-(hydroxymethyl)-1,2-dimethyl-1-[2-(5-oxo-2H-furan-3-yl)ethyl]-2,3,4,7,8,8a-hexahydronaphthalen-4a-yl]methyl acetate | **Diterpenoid Acetate** (Bicyclic, e.g., Labdane-type derivative with furanone) | | | [31] compound 73 |
| 58 | 11551 | 357.2172 | 7.823 | CC(CN=CC1=C(CC(CC1=O)(C)C)O)N=CC2=C(CC(CC2=O)(C)C)O | [8-Hydroxy-4a-(hydroxymethyl)-1,1-dimethyl-5,6-dioxo-7-propan-2-yl-2,3,4,9,10,10a-hexahydrophenanthren-9-yl] acetate | **Diterpenoid Acetate** (Tricyclic, Phenanthrene-based) | 0.064 | 0.333 | [31] compound 115 |

| | | | | | | | | | |
|---|---|---|---|---|---|---|---|---|---|
| 59 | 10543 | 392.2431 | 7.428 | CC(C)C1=C(C2=C(C(=O)C1=O)C3(CCCC(C3CC2OC(=O)C)(C)C)C)O | [9,10-Dihydroxy-6-(methoxymethyl)-12,12-dimethyl-7-oxo-17-oxapentacyclo[7.6.2.15,8.01,11.02,8]octadecan-3-yl] acetate | **Diterpenoid Acetate** (Pentacyclic, rearranged type, e.g., Taxane-type or similar) | 0.082 | 0.333 | |
| 60 | 11997 | 333.2059 | 7.962 | CC1CCC2(C(C1(C)CCC(C)CC(=O)O)CC(=O)C=C2C(=O)O)C | [9,10-Dihydroxy-6-(methoxymethyl)-12,12-dimethyl-7-oxo-17-oxapentacyclo[7.6.2.15,8.01,11.02,8]octadecan-3-yl] acetate | **Diterpenoid Acetate** (Pentacyclic, rearranged type, e.g., Taxane-type or similar) | 0.408 | 0.5 | |
| 61 | 13448 | 299.2005 | 8.562 | CC1=C2C=C3CCC4C(C(CCC4(C3CC2OC1=O)C)O)(C)C | [9,10-Dihydroxy-6-(methoxymethyl)-12,12-dimethyl-7-oxo-17- | **Diterpenoid Acetate** (Pentacyclic, | 0.327 | 0.5 | |

| | | | | | | | | | |
|---|---|---|---|---|---|---|---|---|---|
| | | | | | oxapentacyclo[7.6.2.15,8.01,11.02,8]octadecan-3-yl] acetate | rearranged type, e.g., Taxane-type or similar) | | | |
| 62 | 13866 | 445.2195 | 8.658 | CC(=O)OC1CC2CC3(C1C45CCCC(C4C(C3(OC5)O)O)(C)C)C(=O)C2COC | [9,10-Dihydroxy-6-(methoxymethyl)-12,12-dimethyl-7-oxo-17-oxapentacyclo[7.6.2.15,8.01,11.02,8]octadecan-3-yl] acetate | **Diterpenoid Acetate** (Pentacyclic, rearranged type, e.g., Taxane-type or similar) | 0.185 | 1 | |
| 63 | 13879 | 440.2642 | 8.648 | CC(=O)OC1CC2CC3(C1C45CCCC(C4C(C3(OC5)O)O)(C)C)C(=O)C2COC | [9,10-Dihydroxy-6-(methoxymethyl)-12,12-dimethyl-7-oxo-17-oxapentacyclo[7.6.2.15,8.01,11.02,8]octadecan-3-yl] acetate | **Diterpenoid Acetate** (Pentacyclic, rearranged type, e.g., | 0.28 | 0.333 | |

| | | | | | | | | | |
|---|---|---|---|---|---|---|---|---|---|
| | | | | | | Taxane-type or similar) | | | |
| 64 | 13897 | 423.2376 | 8.651 | CC(=O)OC1CC2CC3(C1C45CCCC(C4C(C3(OC5)O)O)(C)C)C(=O)C2COC | [9,10-Dihydroxy-6-(methoxymethyl)-12,12-dimethyl-7-oxo-17-oxapentacyclo[7.6.2.15,8.01,11.02,8]octadecan-3-yl] acetate | **Diterpenoid Acetate** (Pentacyclic, rearranged type, e.g., Taxane-type or similar) | 0.439 | 0.5 | |
| 65 | 14189 | 440.2642 | 8.731 | CC(=O)OC1CC2CC3(C1C45CCCC(C4C(C3(OC5)O)O)(C)C)C(=O)C2COC | [9,10-Dihydroxy-6-(methoxymethyl)-12,12-dimethyl-7-oxo-17-oxapentacyclo[7.6.2.15,8.01,11.02,8]octadecan-3-yl] acetate | **Diterpenoid Acetate** (Pentacyclic, rearranged type, e.g., Taxane-type or similar) | 0.279 | 0.333 | |

| | | | | | | | | | |
|---|---|---|---|---|---|---|---|---|---|
| 66 | 11672 | 395.2428 | 7.848 | CCCCCCCCCCCC(CC1=C(C(=O)C=C(C1=O)OC)O)OC(=O)C | [Diacetyloxy(hexadecan-4-yl)silyl] acetate | **Organosilane Ester** (Not a terpenoid) | 0.181 | 0.5 | |
| 67 | 11671 | 413.2726 | 7.852 | CCCCCCCCCCCCC(CCC)[Si](OC(=O)C)(OC(=O)C)OC(=O)C | 1-(2-Hydroxy-5-methoxy-3,6-dioxocyclohexa-1,4-dien-1-yl)tridecan-2-yl acetate | **Benzoquinone Derivative** (Not a terpenoid, | 0.004 | 0 | [31] compound 284 |
| 68 | 11683 | 447.1779 | 7.853 | CC(=O)OC1CCC(=C)C(CCC2(C(O2)CC3CCC1(OC(=O)C3=C)C)C)OO | 1-(2-Hydroxy-5-methoxy-3,6-dioxocyclohexa-1,4-dien-1-yl)tridecan-2-yl acetate | **Benzoquinone Derivative** (Not a terpenoid, | 0.076 | 0.999 | [31] compound 284 |
| 69 | 15644 | 395.2427 | 9.192 | CCCCCCCCCCCC(CC1=C(C(=O)C=C(C1=O)OC)O)OC(=O)C | 1-(2-Hydroxy-5-methoxy-3,6-dioxocyclohexa-1,4-dien-1-yl)tridecan-2-yl acetate | **Benzoquinone Derivative** | 0.064 | 0.5 | [31] compound 284 |

| | | | | | | | | | |
|---|---|---|---|---|---|---|---|---|---|
| | | | | | | (Not a terpenoid, | | | |
| 70 | 18374 | 395.2428 | 10.006 | CCCCCCCCCCCC(CC1=C(C(=O)C=C(C1=O)OC)O)OC(=O)C | 1-(2-Hydroxy-5-methoxy-3,6-dioxocyclohexa-1,4-dien-1-yl)tridecan-2-yl acetate | **Benzoquinone Derivative** (Not a terpenoid, | 0.145 | 0.5 | [31] compound 284 |
| 71 | 19403 | 395.2428 | 10.314 | CCCCCCCCCCCC(CC1=C(C(=O)C=C(C1=O)OC)O)OC(=O)C | 1-(2-Hydroxy-5-methoxy-3,6-dioxocyclohexa-1,4-dien-1-yl)tridecan-2-yl acetate | **Benzoquinone Derivative** (Not a terpenoid, | 0.105 | 0.5 | [31] compound 284 |
| 72 | 4231 | 333.206 | 5.168 | CC12CCCC(C1CCC(=C)C2CCC3=CCOC3=O)(C)C(=O)O | 1,4a-dimethyl-6-methylidene-5-[2-(5-oxo-2H-furan-4-yl)ethyl]-3,4,5,7,8,8a-hexahydro-2H-naphthalene-1-carboxylic acid | **Diterpenoid** (Bicyclic, e.g., Labdane-type | 0.229 | 0.5 | [31] compound 64 |

| | | | | | | | | | |
|---|---|---|---|---|---|---|---|---|---|
| | | | | | | derivative with furanone) | | | |
| 73 | 11888 | 368.2427 | 7.906 | CC(=CC(=O)O)C(CC1C(=C)CCC2C1(CCCC2(C)C(=O)O)C)O | 1,4a-dimethyl-6-methylidene-5-[2-(5-oxo-2H-furan-4-yl)ethyl]-3,4,5,7,8,8a-hexahydro-2H-naphthalene-1-carboxylic acid | **Diterpenoid** (Bicyclic, e.g., Labdane-type derivative with furanone) | 0.084 | 0.333 | [31] compound 64 |
| 74 | 14169 | 315.1954 | 8.706 | CC12CCCC(C1CCC(=C)C2CCC3=CCOC3=O)(C)C(=O)O | 1,4a-dimethyl-6-methylidene-5-[2-(5-oxo-2H-furan-4-yl)ethyl]-3,4,5,7,8,8a-hexahydro-2H-naphthalene-1-carboxylic acid | **Diterpenoid (Bicyclic, e.g., Labdane-type derivative** | 0.107 | 0.5 | [31] compound 64 |

| | | | | | | | | | |
|---|---|---|---|---|---|---|---|---|---|
| | | | | | | **with furanone)** | | | |
| 75 | 12050 | 355.2311 | 7.964 | CCCCCCCCCCCCSC1CC(=O)OCCCCOC1=O | 1,9-Bis[(oxan-2-yl)oxy]nona-2,7-diyn-5-ol | **Polyketide/ Non-terpenoid Polyether** (Not a terpenoid) | 0.015 | 0.5 | |
| 76 | 9011 | 386.2531 | 6.923 | CCCCCC(C=CC1C(CC(=O)C1CC(=O)CCCCC(=O)O)O)O | 11,15-Dihydroxy-6,9-dioxoprost-13-en-1-oic acid | **Eicosanoid** (Prostaglandin derivative) | 0.111 | 0.333 | |
| 77 | 11572 | 309.2059 | 7.801 | CCCCCCC1=C(C(=O)C(=C(C1=O)O)CCCCCC)O | 11,15-Dihydroxy-6,9-dioxoprost-13-en-1-oic acid | **Eicosanoid** (Prostaglandin derivative) | 0.099 | 0.5 | |

| | | | | | | | | | |
|---|---|---|---|---|---|---|---|---|---|
| 78 | 12083 | 382.2587 | 7.983 | CC12CCC(C(C1CCC(=C)C2CC(C3=CCOC3=O)OC)(C)CO)O | 11,17,21-Trihydroxy-3,20-dioxopregn-4-en-18-al | **Steroid** (e.g., Corticosteroid or related, not a terpenoid) | 0.472 | 0.333 | [31] compound 161 |
| 79 | 15500 | 275.2005 | 9.164 | CC=C(C)C(C(C)C=C(C)C=CCC(=CCC(=O)O)C)O | 11-Hydroxy-4,8,10,12-tetramethyltetradeca-3,6,8,12-tetraenoic acid | **Fatty Acid Derivative/Polyketide** (Not a terpenoid, l | 0.033 | 0.5 | |
| 80 | 10210 | 411.2378 | 7.341 | CC(=O)OC1CC23CC(C(C2O)CCC3C(C4C1(C(C5C4O5)(C)C)O)(C)O)(C)O | 12-Hydroxy-11,14-dioxoabieta-8,12-dien-7-yl acetate | **Diterpenoid Acetate** (Tricyclic, Abietane-type) | 0.178 | 0.5 | [31] compound 115 |

| | | | | | | | | | |
|---|---|---|---|---|---|---|---|---|---|
| 81 | 10445 | 333.206 | 7.407 | CC1(CCCC2(C1C(C=C(C2CCC(=CC(=O)O)CO)C=O)O)C)C | 12-Hydroxy-11,14-dioxoabieta-8,12-dien-7-yl acetate | **Diterpenoid Acetate** (Tricyclic, Abietane-type) | 0.207 | 0 | [31] compound 115 |
| 82 | 10586 | 345.206 | 7.563 | CC12CCC(C(C1CCC(=C)C2C=CC3=CC(OC3=O)OC)(C)CO)O | 12-Hydroxy-11,14-dioxoabieta-8,12-dien-7-yl acetate | **Diterpenoid Acetate** (Tricyclic, Abietane-type) | 0.095 | 0.5 | [31] compound 115 |
| 83 | 10796 | 455.2639 | 7.621 | C1C(O1)CCC(CC2CO2)C(C(CC3CO3)C(CCC4CO4)OCC5CO5)OCC6CO6 | 12-Hydroxy-11,14-dioxoabieta-8,12-dien-7-yl acetate | **Diterpenoid Acetate** (Tricyclic, Abietane-type) | 0.02 | 0.5 | [31] compound 115 |

| | | | | | | | | | |
|---|---|---|---|---|---|---|---|---|---|
| 84 | 13575 | 468.2955 | 8.637 | CC1=CC(=O)OC1=CC(C(C)(CCC2C3(CCCC(C3CCC2(C)O)(C)C(=O)O)C)O)O | 15-Hydroxy-2-(2-hydroxyethylidene)-10-(hydroxymethyl)-14-methyl-6-methylidene-5-oxopentadeca-9,13-dienoic acid | **Diterpenoid** (Acyclic) | 0.094 | 0.333 | [31] compound 63 |
| 85 | 18256 | 303.2318 | 10.004 | CC1=CCC2C(CCCC2(C1CCC(=CC(=O)O)CO)C)(C)C | 15-Hydroxy-2-(2-hydroxyethylidene)-10-(hydroxymethyl)-14-methyl-6-methylidene-5-oxopentadeca-9,13-dienoic acid | **Diterpenoid** (Acyclic) | 0.148 | 0.5 | [31] compound 65 |
| 86 | 13412 | 421.2582 | 8.495 | CC(=CCCC(=CCCC(=CCCC(=CCO)CO)COC(=O)C)COC(=O)C)CO | 2-(4,8,12-Trimethyltrideca-3,7,11-trienyl)but-2-enedioic acid | **Diterpenoid** (Acyclic) | 0.02 | 0.5 | |
| 87 | 11835 | 373.2005 | 7.881 | CC1CCC2(C(C1(C)CCC3=CCOC3=O)CC(C=C2CO)O)CO | 2-(5-acetyloxy-4-methylpent-3-enyl)-6-methyl-9-(5-oxo-2H-furan-4-yl)nona-2,6-dienoic acid | **Sesterterpenoid Acetate** (Irregular, but related to | 0.168 | 0.995 | [31] compound 280 |

| | | | | | | | | | |
|---|---|---|---|---|---|---|---|---|---|
| | | | | | | sesterterpenoids) | | | |
| 88 | 9267 | 349.2009 | 6.98 | CC(=CCCC1=CCOC1=O)CCC=C(CCC=C(C)CO)C(=O)O | 2-(5-hydroxy-4-methylpent-3-enyl)-6-methyl-9-(5-oxo-2H-furan-4-yl)nona-2,6-dienoic acid | **Sesterterpenoid Acetate** (Irregular, but related to sesterterpenoids) | 0.04 | 0.5 | [31] compound 280 |
| 89 | 10144 | 455.2639 | 7.341 | CC(=O)OC1CC2C(C3CCC4C(C3(CC(C2(C1(C)C)O)OC(=O)C)CC4(C)O)O)(C)O | 2-(5-hydroxy-4-methylpent-3-enyl)-6-methyl-9-(5-oxo-2H-furan-4-yl)nona-2,6-dienoic acid | **Sesterterpenoid Acetate** (Irregular, but related to sesterterpenoids) | 0.086 | 0.5 | [31] compound 280 |

| | | | | | | | | | |
|---|---|---|---|---|---|---|---|---|---|
| 90 | 10956 | 331.1904 | 7.636 | CC(=CCCC1=CCOC1=O)CCC=C(CC=C(C)CO)C(=O)O | 2-(5-hydroxy-4-methylpent-3-enyl)-6-methyl-9-(5-oxo-2H-furan-4-yl)nona-2,6-dienoic acid | **Sesterterpenoid Acetate** (Irregular, but related to sesterterpenoids) | 0.019 | 0.5 | [31] compound 280 |
| 91 | 10995 | 368.2432 | 7.697 | CC1CCC2(C(C1(C)CCC(C)CC(=O)O)CC(=O)C=C2C(=O)O)C | 2-(5-hydroxy-4-methylpent-3-enyl)-6-methyl-9-(5-oxo-2H-furan-4-yl)nona-2,6-dienoic acid | **Sesterterpenoid Acetate** (Irregular, but related to sesterterpenoids) | 0.213 | 0.333 | [31] compound 280 |

| | | | | | | | | | |
|---|---|---|---|---|---|---|---|---|---|
| 92 | 11821 | 351.2167 | 7.906 | CC1CCC2(C(C1(C)CCC(C)CC(=O)O)CC(=O)C=C2C(=O)O)C | 2-(5-hydroxy-4-methylpent-3-enyl)-6-methyl-9-(5-oxo-2H-furan-4-yl)nona-2,6-dienoic acid | **Sesterterpenoid Acetate** (Irregular, but related to sesterterpenoids) | 0.218 | 0.499 | [31] compound 280 |
| 93 | 13604 | 409.2219 | 8.541 | CC1C2C(CC3(C1=CC(=O)O3)O)C4(CCCC(C4C(C2O)OC(=O)C)(C)CO)C | 2-(5-hydroxy-4-methylpent-3-enyl)-6-methyl-9-(5-oxo-2H-furan-4-yl)nona-2,6-dienoic acid | **Sesterterpenoid Acetate** (Irregular, but related to sesterterpenoids) | 0.356 | 0.5 | [31] compound 280 |

| | | | | | | | | | |
|---|---|---|---|---|---|---|---|---|---|
| 94 | 14120 | 331.1903 | 8.719 | CC(=CCCC1=CCOC1=O)CCC=C(CCC=C(C)CO)C(=O)O | 2-(5-hydroxy-4-methylpent-3-enyl)-6-methyl-9-(5-oxo-2H-furan-4-yl)nona-2,6-dienoic acid | **Sesterterpenoid Acetate** (Irregular, but related to sesterterpenoids) | 0.035 | 0.5 | [31] compound 280 |
| 95 | 14338 | 331.1903 | 8.729 | CC(=CCCC1=CCOC1=O)CCC=C(CCC=C(C)CO)C(=O)O | 2-(5-hydroxy-4-methylpent-3-enyl)-6-methyl-9-(5-oxo-2H-furan-4-yl)nona-2,6-dienoic acid | **Sesterterpenoid Acetate** (Irregular, but related to sesterterpenoids) | 0.039 | 0.5 | [31] compound 280 |

| | | | | | | | | | |
|---|---|---|---|---|---|---|---|---|---|
| 96 | 15489 | 331.1904 | 9.057 | CC(=CCCC(=CCCC(=CC(=O)O)C)C(=O)O)CCC=C(C)C=O | 2-(5-hydroxy-4-methylpent-3-enyl)-6-methyl-9-(5-oxo-2H-furan-4-yl)nona-2,6-dienoic acid | **Sesterterpenoid Acetate** (Irregular, but related to sesterterpenoids) | 0.022 | 0.5 | [31] compound 280 |
| 97 | 17964 | 349.2009 | 9.916 | CC12CCCC(C1CC(C34C2C(CC(C3)C(=C)C4=O)O)O)(C)C(=O)O | 2,11-Dihydroxy-5,9-dimethyl-14-methylidene-15-oxotetracyclo[11.2.1.01,10.04,9]hexadecane-5-carboxylic acid | **Diterpenoid** (Tetracyclic, e.g., Grayanane-type) | 0.123 | 0.5 | [31] compound 280 |
| 98 | 19670 | 349.2009 | 10.377 | CC12CCCC(C1CC(C34C2C(CC(C3)C(=C)C4=O)O)O)(C)C(=O)O | 2,11-Dihydroxy-5,9-dimethyl-14-methylidene-15-oxotetracyclo[11.2.1.01,10.04,9]hexadecane-5-carboxylic acid | **Diterpenoid** (Tetracyclic, e.g., Grayanane-type) | 0.204 | 0.5 | [31] compound 280 |

| | | | | | | | | | |
|---|---|---|---|---|---|---|---|---|---|
| 99 | 20296 | 349.201 | 10.529 | CC12CCCC(C1CC(C34C2C(CC(C3)C(=C)C4=O)O)O)(C)C(=O)O | 2,11-Dihydroxy-5,9-dimethyl-14-methylidene-15-oxotetracyclo[11.2.1.01,10.04,9]hexadecane-5-carboxylic acid | **Diterpenoid** (Tetracyclic, e.g., Grayanane-type) | 0.179 | 0.5 | [31] compound 280 |
| 100 | 11566 | 386.2536 | 7.829 | CCCCCC(C=CC1C(CC(=O)C1CC(=O)CCCCC(=O)O)O)O | 2,2'-[1,2-Propanediylbis(iminomethylidene)]bis(5,5-dimethyl-1,3-cyclohexanedione) | Non-terpenoid Dione/Schiff Base Derivative | 0.104 | 0.333 | |
| 101 | 19285 | 498.3415 | 10.26 | CC12CCC3C(=CC(=O)C4C3(CC(C(C4)O)O)C)C1(CCC2C(C)(C(CCC(C)(C)O)O)O)O | 2,3,14,20,22,25-Hexahydroxycholest-7-en-6-one | **Triterpenoid/Steroid** (Specifically, a phytosteroid or | 0.047 | 0.333 | [31] compound 164 |

| | | | | | | | | | |
|---|---|---|---|---|---|---|---|---|---|
| | | | | | | ecdysteroid-like, | | | |
| 102 | 18135 | 526.3384 | 9.961 | CC12CCC3C(=CC(=O)C4C3(CC(C(C4)O)O)C)C1(CCC2C(C)(C(CC(=C)C(C)(CO)O)O)O)O)O | 2,3,14-trihydroxy-10,13-dimethyl-17-(2,3,6,7-tetrahydroxy-6-methyl-5-methylideneheptan-2-yl)-2,3,4,5,9,11,12,15,16,17-decahydro-1H-cyclopenta[a]phenanthren-6-one | **Steroid** (e.g., C27 steroid | 0.1 | 0.332 | [31] compound 155 |
| 103 | 13743 | 303.2318 | 7.208 | CC1=C(C2(CCCC(C2CC1=O)(C)C)C)CCC(=CCO)CO | 2,3-Dimethoxy-5-undecyl-1,4-benzoquinone | | 0.565 | 0.5 | |
| 104 | 11599 | 375.2165 | 7.217 | CCCCCC1C(O1)CC=CC=CC(CC=CCCCC(=O)O)OO | 2,5-Dihexyl-3,6-dihydroxybenzo-1,4-quinone | | 0.024 | 0.159 | |
| 105 | 17804 | 387.2524 | 9.904 | CCCCCCCCC1=C(C(=O)C(=C(C1=O)O)CCCCCCCC)O | 2,5-Dihydroxy-3,6-dioctylcyclohexa-2,5-diene-1,4-dione | **Benzoquinone Derivative** | 0.047 | 0 | |

| | | | | | | | | | |
|---|---|---|---|---|---|---|---|---|---|
| | | | | | | (Not a terpenoid) | | | |
| 106 | 15638 | 409.2584 | 9.192 | CC(=O)CCCC(=O)CCCC(=O)CCCC(=O)CCCC(=O)CCCC(=O)C | 2,6,10,14,18,22-Tricosanehexone | **Fatty Acid Derivative/Polyketide** (Not a terpenoid | 0.06 | 0.5 | |
| 107 | 15665 | 426.2845 | 9.204 | CC(=O)CCCC(=O)CCCC(=O)CCCC(=O)CCCC(=O)CCCC(=O)C | 2,6,10,14,18,22-Tricosanehexone | **Fatty Acid Derivative/Polyketide** (Not a terpenoid | 0.053 | 0.333 | |
| 108 | 9652 | 361.2008 | 7.202 | CCCCCCCCCC(=CC1=C(C(=O)C(=C(C1=O)OC)OC)C)C(=O)O | 2-[(4,5-Dimethoxy-2-methyl-3,6-dioxocyclohexa-1,4-dien-1-yl)methylidene]undecanoic acid | **Benzoquinone Derivative** (Not a terpenoid) | 0.05 | 0.5 | [31] compound no 244 |

| | | | | | | | | | |
|---|---|---|---|---|---|---|---|---|---|
| 109 | 9902 | 361.2009 | 7.224 | CCCCCCCCCC(=CC1=C(C(=O)C(=C(C1=O)OC)OC)C)C(=O)O | 2-[(4,5-Dimethoxy-2-methyl-3,6-dioxocyclohexa-1,4-dien-1-yl)methylidene]undecanoic acid | **Benzoquinone Derivative** (Not a terpenoid) | 0.05 | 0.5 | [31] compound no 244 |
| 110 | 19864 | 465.285 | 10.39 | CC12CCC(C(C1CCC34C2CCC(C3)C(=C)C4O)(C)COC5C(C(C(C(O5)CO)O)O)O)O | 2-[(6,15-Dihydroxy-5,9-dimethyl-14-methylidene-5-tetracyclo[11.2.1.01,10.04,9]hexadecanyl)methoxy]-6-(hydroxymethyl)oxane-3,4,5-triol | **Diterpenoid Glycoside** (Tetracyclic | 0.026 | 0.5 | |
| 111 | 10828 | 409.222 | 7.597 | CC1C=C(C(=O)C2(C1CC3C4(C2C(C(C(C4(CC(=O)O3)O)C)OC)O)C)C)OC | 2-[[1,8-Bis(oxiran-2-yl)-6-(oxiran-2-ylmethoxy)-3,5-bis(oxiran-2-ylmethyl)octan-4-yl]oxymethyl]oxirane | **Polyether/Epoxy Compound** (Not a terpenoid) | 0.162 | 0.5 | |
| 112 | 11669 | 377.2323 | 7.844 | CC(=O)C=CC1C(=CC(C2C1(CCCC2(C)C)C)OC(=O)C)COC(=O)C | 2-[2-(1,2,4a,5-Tetramethyl-2,3,4,7,8,8a- | **Diterpenoid** (Bicyclic, | 0.326 | 0.5 | |

| | | | | | | | | | |
|---|---|---|---|---|---|---|---|---|---|
| | | | | | hexahydronaphthalen-1-yl)ethyl]but-2-enedioic acid | e.g., Labdane-type derivative) | | | |
| 113 | 13545 | 461.2895 | 8.517 | CC(=O)OC1CC2C(CCC3C2(CCC4C3(CCCC4(C)C)C)C)C5OC6C1(O5)OC(=O)C6 | 2-[2-(1,2,4a,5-Tetramethyl-2,3,4,7,8,8a-hexahydronaphthalen-1-yl)ethyl]but-2-enedioic acid | **Diterpenoid** (Bicyclic, e.g., Labdane-type derivative) | 0.185 | 0.5 | |
| 114 | 20359 | 407.2786 | 10.551 | CC(CC(C(=O)O)O)C1CCC2C1(C(CC3C2C(CC4C3(CCC(C4)O)C)O)O)C | 23-Hydroxycholic acid | **Steroid/Bile Acid** (Not a terpenoid) | 0.082 | 0.5 | [31] compound 129 |
| 115 | 4897 | 332.2221 | 5.428 | CC1CCC2C(C1(C)CC3=CC(=O)C=C(C3=O)O)CCC=C2C | 2-hydroxy-6-[(1,2,5-trimethyl-3,4,4a,7,8,8a-hexahydro-2H-naphthalen-1- | **Diterpenoid-Quinone** (Combined | 0.132 | 0.332 | |

| | | | | | | | | | |
|---|---|---|---|---|---|---|---|---|---|
| | | | | | yl)methyl]cyclohexa-2,5-diene-1,4-dione | diterpenoid and quinone structure) | | | |
| 116 | 8803 | 332.222 | 6.891 | CC1CCC2C(C1(C)CC3=CC(=O)C=C(C3=O)O)CCC=C2C | 2-hydroxy-6-[(1,2,5-trimethyl-3,4,4a,7,8,8a-hexahydro-2H-naphthalen-1-yl)methyl]cyclohexa-2,5-diene-1,4-dione | **Diterpenoid -Quinone** (Combined diterpenoid and quinone structure) | 0.068 | 0.333 | |
| 117 | 11896 | 301.2162 | 7.924 | CC1=C(C2(CCCC(C2CC1=O)(C)C)C)CCC(=CC(=O)O)C | 3-(Acetyloxymethyl)-5-(2-formyl-4-hydroxy-5,5,8a-trimethyl-1,4,4a,6,7,8-hexahydronaphthalen-1-yl)pent-2-enoic acid | **Diterpenoid Acetate** (Bicyclic, e.g., Labdane-type derivative) | 0.349 | 0.5 | [31] compound 105 |

| | | | | | | | | | |
|---|---|---|---|---|---|---|---|---|---|
| 118 | 14199 | 410.2535 | 8.745 | CC(=O)OCC(=CC(=O)O)CCC1C(=CC(C2C1(CCCC2(C)C)C)O)C=O | 3-(Acetyloxymethyl)-5-(2-formyl-4-hydroxy-5,5,8a-trimethyl-1,4,4a,6,7,8-hexahydronaphthalen-1-yl)pent-2-enoic acid | **Diterpenoid Acetate** (Bicyclic, e.g., Labdane-type derivative) | 0.118 | 0.333 | [31] compound 105 |
| 119 | 11699 | 452.2642 | 7.873 | CC(=O)OC1C(C2(CC(=O)OC2O1)OC(=O)C)C34CCC5C(CCCC5(C3C4)C)(C)C | 3-(Acetyloxymethyl)-5-(2-formyl-5,5,8a-trimethyl-1,4,4a,6,7,8-hexahydronaphthalen-1-yl)pent-2-enoic acid | **Diterpenoid Acetate** (Bicyclic, e.g., Labdane-type derivative) | 0.087 | 0.333 | [31] compound 105 |

| | | | | | | | | | |
|---|---|---|---|---|---|---|---|---|---|
| 120 | 15286 | 359.2217 | 9.182 | CC(=O)OCC(=CC(=O)O)CCC1C(=CCC2C1(CCCC2(C)C)C)C=O | 3-(Acetyloxymethyl)-5-(2-formyl-5,5,8a-trimethyl-1,4,4a,6,7,8-hexahydronaphthalen-1-yl)pent-2-enoic acid | **Diterpenoid Acetate** (Bicyclic, e.g., Labdane-type derivative) | 0.195 | 0.5 | [31] compound 105 |
| 121 | 15465 | 377.2322 | 9.161 | CC(=O)OCC(=CC(=O)O)CCC1C(=CCC2C1(CCCC2(C)C)C)C=O | 3-(Acetyloxymethyl)-5-(2-formyl-5,5,8a-trimethyl-1,4,4a,6,7,8-hexahydronaphthalen-1-yl)pent-2-enoic acid | **Diterpenoid Acetate** (Bicyclic, e.g., Labdane-type derivative) | 0.149 | 0.5 | [31] compound 105 |

| | | | | | | | | | |
|---|---|---|---|---|---|---|---|---|---|
| 122 | 20072 | 359.2215 | 10.468 | CC(=O)OCC(=CC(=O)O)CCC1C(=CCC2C1(CCCC2(C)C)C)C=O | 3-(Acetyloxymethyl)-5-(2-formyl-5,5,8a-trimethyl-1,4,4a,6,7,8-hexahydronaphthalen-1-yl)pent-2-enoic acid | **Diterpenoid Acetate** (Bicyclic, e.g., Labdane-type derivative) | 0.276 | 0.5 | [31] compound 105 |
| 123 | 10074 | 333.206 | 7.28 | CC1=C(CCC2C1CCC3C2(CCCC3(C)C(=O)O)C(=O)O)C=C | 3-(acetyloxymethyl)-5-(4-hydroxy-5,5,8a-trimethyl-2-methylidene-3,4,4a,6,7,8-hexahydro-1H-naphthalen-1-yl)pent-2-enoic acid | **Diterpenoid Acetate** (Bicyclic, e.g., Labdane-type derivative) | 0.12 | 0.5 | [31] compound 105 |
| 124 | 12082 | 359.1831 | 7.98 | CC12CCC(=O)C=C1CCC3C2C(CC4(C3CCC4(C(=O)CO)O)C=O)O | 3-(Dodecylsulfanyl)-1,6-dioxecane-2,5-dione | **Macrocyclic Ester/Sulfid** | 0.052 | 0.5 | |

| | | | | | | | | | |
|---|---|---|---|---|---|---|---|---|---|
| | | | | | | **e** (Not a terpenoid) | | | |
| 125 | 19352 | 407.2425 | 10.277 | CCC(C)C(=O)OC1CC(C=C2C1C(C(C=C2)C)CCC(CC(CC(=O)O)O)O)O | 3,5-Dihydroxy-7-[6-hydroxy-2-methyl-8-(2-methyl-1-oxobutoxy)-1,2,6,7,8,8a-hexahydronaphthalen-1-yl]heptanoic acid | **Decalin-Lactone/Polyketide Hybrid** (Not a classic terpenoid, though contains a terpenoid-like bicyclic moiety) | 0.07 | 0.5 | |
| 126 | 19448 | 407.2426 | 10.264 | CCC(C)C(=O)OC1CC(C=C2C1C(C(C=C2)C)CCC(CC(CC(=O)O)O)O)O | 3,5-Dihydroxy-7-[6-hydroxy-2-methyl-8-(2-methyl-1-oxobutoxy)-1,2,6,7,8,8a- | **Decalin-Lactone/Polyketide Hybrid** (Not | 0.093 | 0.5 | |

| | | | | | | | | | |
|---|---|---|---|---|---|---|---|---|---|
| | | | | | hexahydronaphthalen-1-yl]heptanoic acid | a classic terpenoid, though contains a terpenoid-like bicyclic moiety) | | | |
| 127 | 8686 | 441.2482 | 6.768 | CC1C=CC2=CC(CC(C2C1CCC(CC(CC(=O)O)O)O)OC(=O)C(C)C(C)O)O | 3,5-Dihydroxy-7-[6-hydroxy-8-(3-hydroxy-2-methylbutanoyl)oxy-2-methyl-1,2,6,7,8,8a-hexahydronaphthalen-1-yl]heptanoic acid | **Decalin-Lactone/Polyketide Hybrid** (Not a classic terpenoid, though contains a terpenoid- | 0.111 | 0.433 | |

| | | | | | | | | | |
|---|---|---|---|---|---|---|---|---|---|
| | | | | | | like bicyclic moiety) | | | |
| 128 | 9036 | 441.2483 | 6.943 | CC1C=CC2=CC(CC(C2C1CCC(CC(CC(=O)O)O)O)OC(=O)C(C)C(C)O)O | 3,5-Dihydroxy-7-[6-hydroxy-8-(3-hydroxy-2-methylbutanoyl)oxy-2-methyl-1,2,6,7,8,8a-hexahydronaphthalen-1-yl]heptanoic acid | **Decalin-Lactone/Polyketide Hybrid** (Not a classic terpenoid, though contains a terpenoid-like bicyclic moiety) | 0.061 | 0 | |
| 129 | 10102 | 351.2166 | 7.297 | CC(CCCC(C=CC1C=CC(=O)C1CC=CCCCC(=O)O)O)O | 3,5-Dihydroxy-7-[6-hydroxy-8-(3-hydroxy-2-methylbutanoyl)oxy-2-methyl-1,2,6,7,8,8a- | **Decalin-Lactone/Polyketide Hybrid** (Not | 0.03 | 0.5 | |

| | | | | | | | | | |
|---|---|---|---|---|---|---|---|---|---|
| | | | | | hexahydronaphthalen-1-yl]heptanoic acid | a classic terpenoid, though contains a terpenoid-like bicyclic moiety) | | | |
| 130 | 10242 | 350.2325 | 7.335 | CC1=CC(=O)C2C(CCCC2(C1CCC(=CC(=O)O)C=O)C)(C)C | 3,5-Dihydroxy-7-[6-hydroxy-8-(3-hydroxy-2-methylbutanoyl)oxy-2-methyl-1,2,6,7,8,8a-hexahydronaphthalen-1-yl]heptanoic acid | **Decalin-Lactone/Polyketide Hybrid** (Not a classic terpenoid, though contains a terpenoid- | 0.046 | 0.331 | |

| | | | | | | | | | |
|---|---|---|---|---|---|---|---|---|---|
| | | | | | | like bicyclic moiety) | | | |
| 131 | 11037 | 369.2465 | 7.662 | CC1=C(C(C(=C(C1(OC)OC)C)CO[Si](C)(C)C(C)(C)C)(OC)OC)C | 3,5-Dihydroxy-7-[6-hydroxy-8-(3-hydroxy-2-methylbutanoyl)oxy-2-methyl-1,2,6,7,8,8a-hexahydronaphthalen-1-yl]heptanoic acid | **Decalin-Lactone/Polyketide Hybrid** (Not a classic terpenoid, though contains a terpenoid-like bicyclic moiety) | 0.01 | 0.198 | |
| 132 | 11446 | 443.2832 | 7.741 | CCCCCCO[Si](OCCCCCC)(OCCCCCC)OC1CC(=O)OC(=O)C1 | 3,5-Dihydroxy-7-[6-hydroxy-8-(3-hydroxy-2-methylbutanoyl)oxy-2-methyl-1,2,6,7,8,8a- | **Decalin-Lactone/Polyketide Hybrid** (Not | 0.013 | 0 | |

| | | | | | | | | | |
|---|---|---|---|---|---|---|---|---|---|
| | | | | | hexahydronaphthalen-1-yl]heptanoic acid | a classic terpenoid, though contains a terpenoid-like bicyclic moiety) | | | |
| 133 | 11581 | 458.2748 | 7.93 | CC1C=CC2=CC(CC(C2C1CCC(CC(CC(=O)O)O)O)OC(=O)C(C)C(C)O)O | 3,5-Dihydroxy-7-[6-hydroxy-8-(3-hydroxy-2-methylbutanoyl)oxy-2-methyl-1,2,6,7,8,8a-hexahydronaphthalen-1-yl]heptanoic acid | **Decalin-Lactone/Polyketide Hybrid** (Not a classic terpenoid, though contains a terpenoid- | 0.107 | 0.328 | |

| | | | | | | | | | |
|---|---|---|---|---|---|---|---|---|---|
| | | | | | | like bicyclic moiety) | | | |
| 134 | 11613 | 480.1439 | 7.873 | CC(C)(C)OC(=O)N1CC(=O)CC1C(C(CO)[Se]C2=CC=CC=C2)OCOCCOC | 3,5-Dihydroxy-7-[6-hydroxy-8-(3-hydroxy-2-methylbutanoyl)oxy-2-methyl-1,2,6,7,8,8a-hexahydronaphthalen-1-yl]heptanoic acid | **Decalin-Lactone/Polyketide Hybrid** (Not a classic terpenoid, though contains a terpenoid-like bicyclic moiety) | #NAME? | 0.002 | |
| 135 | 11871 | 415.2092 | 7.925 | CC1CCC2(C(C1(C)CCC(=CC(=O)O)COC(=O)C)CCC=C2C(=O)O)C | 3,5-Dihydroxy-7-[6-hydroxy-8-(3-hydroxy-2-methylbutanoyl)oxy-2-methyl-1,2,6,7,8,8a- | **Decalin-Lactone/Polyketide Hybrid** (Not | 0.152 | 0.997 | |

| | | | | | | | | | |
|---|---|---|---|---|---|---|---|---|---|
| | | | | | hexahydronaphthalen-1-yl]heptanoic acid | a classic terpenoid, though contains a terpenoid-like bicyclic moiety) | | | |
| 136 | 12043 | 463.2301 | 7.971 | CC1C=CC2=CC(CC(C2C1CCC(CC(CC(=O)O)O)O)OC(=O)C(C)C(C)O)O | 3,5-Dihydroxy-7-[6-hydroxy-8-(3-hydroxy-2-methylbutanoyl)oxy-2-methyl-1,2,6,7,8,8a-hexahydronaphthalen-1-yl]heptanoic acid | **Decalin-Lactone/Polyketide Hybrid** (Not a classic terpenoid, though contains a terpenoid- | 0.1 | 1 | |

| | | | | | | | | | |
|---|---|---|---|---|---|---|---|---|---|
| | | | | | | like bicyclic moiety) | | | |
| 137 | 12044 | 442.2798 | 7.974 | CC(=O)OC1CCC2(C(O2)CC(CCC(C3CCC1(O3)C)(C)O)C(=C)C(=O)OC)C | 3,5-Dihydroxy-7-[6-hydroxy-8-(3-hydroxy-2-methylbutanoyl)oxy-2-methyl-1,2,6,7,8,8a-hexahydronaphthalen-1-yl]heptanoic acid | **Decalin-Lactone/Polyketide Hybrid** (Not a classic terpenoid, though contains a terpenoid-like bicyclic moiety) | 0.132 | 0.333 | |
| 138 | 14177 | 423.2376 | 8.682 | CC1C=CC2=CC(CC(C2C1CCC(CC(CC(=O)O)O)O)OC(=O)C(C)C(C)O)O | 3,5-Dihydroxy-7-[6-hydroxy-8-(3-hydroxy-2-methylbutanoyl)oxy-2-methyl-1,2,6,7,8,8a- | **Decalin-Lactone/Polyketide Hybrid** (Not | 0.101 | 0.5 | |

| | | | | | | | | | |
|---|---|---|---|---|---|---|---|---|---|
| | | | | | hexahydronaphthalen-1-yl]heptanoic acid | a classic terpenoid, though contains a terpenoid-like bicyclic moiety) | | | |
| 139 | 14442 | 327.1954 | 8.811 | CC1(CCCC2(C1CCC(=C)C2CC3=C(C(=O)C=C(C3=O)O)O)C)C | 3-[(5,5,8a-trimethyl-2-methylidene-3,4,4a,6,7,8-hexahydro-1H-naphthalen-1-yl)methyl]-2,5-dihydroxycyclohexa-2,5-diene-1,4-dione | **Diterpenoid-Quinone** (Combined diterpenoid and quinone structure) | 0.072 | 0.5 | |
| 140 | 17802 | 382.2949 | 9.917 | CCCCCCCCCCCCCCCCC1=C(C(=O)C=C(C1=O)O)O | 3-Hexadecyl-2,5-dihydroxycyclohexa-2,5-diene-1,4-dione | **Benzoquinone Derivative** | 0.069 | 0.333 | |

| | | | | | | | | | |
|---|---|---|---|---|---|---|---|---|---|
| | | | | | | (Not a terpenoid) | | | |
| 141 | 13528 | 317.211 | 8.489 | CC1CCC2(C(C1(C)CCC(=CC(=O)O)C(=O)O)CCC=C2C)C | 3-hydroxy-4,4,8,11b-tetramethyl-2,3,4a,5,6,10a,11,11a-octahydro-1H-naphtho[2,1-f][1]benzofuran-9-one | **Diterpenoid** (Polycyclic, Naphthobenzofuran derivative) | 0.154 | 0.5 | |
| 142 | 8922 | 367.2115 | 6.891 | CCCCCC(=CC(=C(CCCC)C(=O)O)C=C(CC)C(=O)O)C(=O)O | 4-(1-Carboxypentylidene)-2-ethyl-6-pentylhepta-2,5-dienedioic acid | **Complex Fatty Acid Derivative/Polyketide** (Not a terpenoid) | 0.03 | 0.5 | |
| 143 | 15195 | 349.201 | 9.099 | CCCCCC(=CC(=C(CCCC)C(=O)O)C=C(CC)C(=O)O)C(=O)O | 4-(1-Carboxypentylidene)-2-ethyl-6-pentylhepta-2,5-dienedioic acid | **Complex Fatty Acid Derivative/Polyketide** | 0.163 | 0.5 | |

| | | | | | | | | | |
|---|---|---|---|---|---|---|---|---|---|
| | | | | | | (Not a terpenoid) | | | |
| 144 | 11160 | 347.1854 | 7.742 | CC(=CCCC(=CC(=O)O)C)CCC=C(CCC=C(C)C(=O)O)C(=O)O | 4-(2,2-Dimethyl-6-{[4-(2-methylpropoxy)phenyl]methoxy}-tetrahydro-2H-furo[2,3-D][1,3]dioxol-5-YL)-2,2-dimethyl-1,3-dioxolane | **Complex Carbohydrate/Aromatic Ether** (Not a terpenoid) | 0.076 | 0.5 | [31] compound 260 |
| 145 | 15892 | 506.3116 | 9.289 | CC1=C(C(=O)OC(C1)C(C)C2(CCC3(C2(CCC4C3CC(C5(C4(C(=O)C=CC5)C)O)O)C)O)O)C | 4,5-dimethyl-2-[1-(5,6,14,17-tetrahydroxy-10,13-dimethyl-1-oxo-6,7,8,9,11,12,15,16-octahydro-4H-cyclopenta[a]phenanthren-17-yl)ethyl]-2,3-dihydropyran-6-one | **Steroid Lactone/Glycoside** (Not a terpenoid) | 0.084 | 0.278 | [31] compound 164 |
| 146 | 11865 | 423.2376 | 7.947 | CC1C=CC2=CC(CC(C2C1CCC(CC(CC(=O)O)O)O)O)OC(=O)C(C)C(C)O)O | 4-[2-(6,7-dihydroxy-1,2,4a-trimethylspiro[3,4,6,7,8,8a-hexahydro-2H-naphthalene-5,2'- | **Diterpenoid** (Spiro-oxirane, Furanone, | 0.111 | 0.5 | [31] compound 71 |

| | | | | | | | | | |
|---|---|---|---|---|---|---|---|---|---|
| | | | | | oxirane]-1-yl)ethyl]-2-methoxy-2H-furan-5-one | Bicyclic derivative) | | | |
| 147 | 14476 | 317.2111 | 8.871 | CC1C(C(=O)C2C(CCCC2(C1(CCC3=COC=C3)O)C)(C)C)O | 4-[2-(Furan-3-yl)ethyl]-2,4-dihydroxy-3,4a,8,8-tetramethyl-2,3,5,6,7,8a-hexahydronaphthalen-1-one | Diterpenoid (Bicyclic, e.g., Labdane-type derivative with furan) | 0.515 | 0.5 | |
| 148 | 15534 | 317.211 | 9.184 | CC1C(C(=O)C2C(CCCC2(C1(CCC3=COC=C3)O)C)(C)C)O | 4-[2-(Furan-3-yl)ethyl]-2,4-dihydroxy-3,4a,8,8-tetramethyl-2,3,5,6,7,8a-hexahydronaphthalen-1-one / Leoheterin | Diterpenoid (Bicyclic, e.g., Labdane-type derivative with furan) | 0.353 | 0.5 | [31] Compound 72 |

| | | | | | | | | | |
|---|---|---|---|---|---|---|---|---|---|
| 149 | 11913 | 319.2267 | 7.939 | CC1C(=O)CC2C(CCCC2(C1(CCC3=COC=C3)O)C)(C)C | 4-[2-(Furan-3-yl)ethyl]-3-hydroxy-3,4a,8,8-tetramethyl-2,4,5,6,7,8a-hexahydronaphthalen-1-one | Diterpenoid (Bicyclic, e.g., Labdane-type derivative with furan) | 0.681 | 0.5 | |
| 150 | 19807 | 319.2267 | 10.445 | CC1(CCCC2(C1C(=O)CC(C2CCC3=COC=C3)(C)O)C)C | 4-[2-(Furan-3-yl)ethyl]-3-hydroxy-3,4a,8,8-tetramethyl-2,4,5,6,7,8a-hexahydronaphthalen-1-one | Diterpenoid (Bicyclic, e.g., Labdane-type derivative with furan) | 0.726 | 0.5 | |
| 151 | 9006 | 301.2162 | 6.832 | CC1=C(C2(CCCC(C2CC1=O)(C)C)C)CCC(=CC(=O)O)C | 4-[2-(Furan-3-yl)ethyl]-4-hydroxy-3,4a,8,8-tetramethyl- | Diterpenoid (Bicyclic, e.g., | 0.555 | 0.5 | |

| | | | | | | | | | |
|---|---|---|---|---|---|---|---|---|---|
| | | | | | 1,3,5,6,7,8a-hexahydronaphthalen-2-one | Labdane-type derivative with furan) | | | |
| 152 | 11915 | 354.2639 | 7.912 | CCCCCC(C=CC=CC=CC(CC=CCCCC(=O)O)O)O | 4-[2-(Furan-3-yl)ethyl]-4-hydroxy-3,4a,8,8-tetramethyl-1,3,5,6,7,8a-hexahydronaphthalen-2-one | Diterpenoid (Bicyclic, e.g., Labdane-type derivative with furan) | 0.021 | 0.333 | |
| 153 | 18380 | 319.2267 | 10.015 | CC1C(=O)CC2C(CCCC2(C1(CCC3=COC=C3)O)C)(C)C | 4-[2-(Furan-3-yl)ethyl]-4-hydroxy-3,4a,8,8-tetramethyl-1,3,5,6,7,8a-hexahydronaphthalen-2-one | Diterpenoid (Bicyclic, e.g., Labdane-type | 0.519 | 0.5 | |

| | | | | | | | | | |
|---|---|---|---|---|---|---|---|---|---|
| | | | | | | derivative with furan) | | | |
| 154 | 12089 | 456.2957 | 7.976 | CC(=CCC(C=C(CCO)CO)C(=O)OCC(=CC(CC=C(C)C)C(=O)O)CCO)C | 4-[2-[6-hydroxy-5-(hydroxymethyl)-5,8a-dimethyl-2-methylidene-3,4,4a,6,7,8-hexahydro-1H-naphthalen-1-yl]-1-methoxyethyl]-2H-furan-5-one<br><br>See image right | **Diterpenoid** (Bicyclic, e.g., Labdane-type derivative with furanone) | 0.015 | 0.333 | [31] compound 67 |
| 155 | 10797 | 426.2486 | 7.617 | CC1C=C(C(=O)C2(C1CC3C4(C2C(C(C(C4(CC(=O)O3)O)C)OC)O)C)C)OC | 4-[2-[6-hydroxy-5-(hydroxymethyl)-5,8a-dimethyl-2-methylidene-3,4,4a,6,7,8-hexahydro-1H-naphthalen-1-yl]ethenyl]-2-methoxy-2H-furan-5-one | **Diterpenoid** (Bicyclic, e.g., Labdane-type derivative | 0.096 | 0.333 | [31] compound no 66 |

| | | | | | | | | | |
|---|---|---|---|---|---|---|---|---|---|
| | | | | | | with furanone) | | | |
| 156 | 10433 | 444.259 | 7.412 | CC(=O)OCC1(C2CCC3C(C2)(C1O)C4(C(C5C3(CO4)C(CCC5(C)C)O)O)O)O | 4-Hydroxy-8-(1-hydroxy-4,4-dimethyl-3-oxo-1,5,6,7-tetrahydro-2-benzofuran-5-yl)-2-methyl-6-methylideneoct-2-enal | **Modified Diterpenoid** (Irregular, or a combination of terpenoid-like and non-terpenoid elements) | 0.088 | 0.214 | |
| 157 | 8334 | 315.1954 | 6.74 | CC1CCC2(C(C1(C)CCC(=CC(=O)O)C=O)CC(=O)C=C2C)C | 5-(1,2,4a,5-tetramethyl-7-oxo-3,4,8,8a-tetrahydro-2H-naphthalen-1-yl)-3-formylpent-2-enoic acid | **Diterpenoid** (Tricyclic, e.g., Abietane-type derivative) | 0.068 | 0.5 | [31] compound 105 |

| | | | | | | | | | |
|---|---|---|---|---|---|---|---|---|---|
| 158 | 15474 | 352.248 | 9.164 | CC1=CCC2C(CCCC2(C1CC(C(=CC(=O)O)C=O)O)C)(C)C | 5-(2,5,5,8a-Tetramethyl-1,4,4a,6,7,8-hexahydronaphthalen-1-yl)-3-formyl-4-hydroxypent-2-enoic acid | **Diterpenoid** (Bicyclic, e.g., Labdane-type derivative) | 0.169 | 0.333 | [31] compound 109 |
| 159 | 9052 | 319.2267 | 6.881 | CC1=C(C2(CCCC(C2CC1=O)(C)C)C)CCC(=CC(=O)O)C | 5-(2,5,5,8a-tetramethyl-3-oxo-4a,6,7,8-tetrahydro-4H-naphthalen-1-yl)-3-methylpent-2-enoic acid | **Diterpenoid** (Bicyclic, e.g., Labdane-type derivative) | 0.28 | 0.5 | [31] compound 105 |
| 160 | 10331 | 349.2008 | 7.416 | CC(=CC(CC(=C)CCC1CCC2=C(C1(C)C)C(=O)OC2O)O)C=O | 5-(2,5,5,8a-tetramethyl-4-oxo-4a,6,7,8-tetrahydro-1H-naphthalen-1-yl)-3-formylpent-2-enoic acid | **Diterpenoid** (Bicyclic, e.g., Labdane- | 0.123 | 0.5 | [31] compound 105 |

| | | | | | | | | | |
|---|---|---|---|---|---|---|---|---|---|
| | | | | | | type derivative) | | | |
| 161 | 8545 | 351.2167 | 6.839 | CC1(CCCC2(C1C(C=C(C2CCC(=CC(=O)O)CO)C=O)O)C)C | 5-(2-Formyl-4-hydroxy-5,5,8a-trimethyl-1,4,4a,6,7,8-hexahydronaphthalen-1-yl)-3-(hydroxymethyl)pent-2-enoic acid | **Diterpenoid** (Bicyclic, e.g., Labdane-type derivative) | 0.287 | 0.5 | [31] compound 105 |
| 162 | 10522 | 423.2376 | 7.489 | CC(=O)OC1CC2CC3(C1C45CCCC(C4C(C3(OC5)O)O)(C)C)C(=O)C2COC | 5-(2-Formyl-4-hydroxy-5,5,8a-trimethyl-1,4,4a,6,7,8-hexahydronaphthalen-1-yl)-3-(hydroxymethyl)pent-2-enoic acid | **Diterpenoid** (Bicyclic, e.g., Labdane-type derivative) | 0.254 | 0.5 | [31] compound 105 |

| | | | | | | | | | |
|---|---|---|---|---|---|---|---|---|---|
| 163 | 15209 | 351.2167 | 9.073 | CC1(CCCC2(C1C(C=C(C2CCC(=CC(=O)O)CO)C=O)O)C)C | 5-(2-Formyl-4-hydroxy-5,5,8a-trimethyl-1,4,4a,6,7,8-hexahydronaphthalen-1-yl)-3-(hydroxymethyl)pent-2-enoic acid | **Diterpenoid** (Bicyclic, e.g., Labdane-type derivative) | 0.172 | 0.5 | [31] compound 105 |
| 164 | 11698 | 359.2215 | 7.855 | CC(=O)OCC(=CC(=O)O)CCC1C(=CCC2C1(CCCC2(C)C)C)C=O | 5-(2-Formyl-5,5,8a-trimethyl-1,4,4a,6,7,8-hexahydronaphthalen-1-yl)-3-(hydroxymethyl)pent-2-enoic acid | **Diterpenoid** (Bicyclic, e.g., Labdane-type derivative) | 0.331 | 0.5 | [31] compound 105 |
| 165 | 15513 | 335.2216 | 9.173 | CC1(CCCC2(C1CC=C(C2CCC(=CC(=O)O)CO)C=O)C)C | 5-(2-Formyl-5,5,8a-trimethyl-1,4,4a,6,7,8-hexahydronaphthalen-1-yl)-3-(hydroxymethyl)pent-2-enoic acid | **Diterpenoid** (Bicyclic, e.g., Labdane- | 0.052 | 0.5 | [31] compound 105 |

| | | | | | | | | | |
|---|---|---|---|---|---|---|---|---|---|
| | | | | | | type derivative) | | | |
| 166 | 11892 | 375.2165 | 7.924 | CC(=O)OCC(=CC(=O)O)CCC1C(=CC(C2C1(CCCC2(C)C)C)O)C=O | 5-(4-carboxy-2-hydroxy-3-methylbut-3-enyl)-1,4a-dimethyl-6-methylidene-3,4,5,7,8,8a-hexahydro-2H-naphthalene-1-carboxylic acid | **Diterpenoid** (Bicyclic, e.g., Labdane-type derivative) | 0.199 | 0.5 | [31] compound 105 |
| 167 | 9497 | 331.1903 | 7.133 | CC1CCC2(C(C1(C)CCC(=CC(=O)O)C)CC(=O)C=C2C(=O)O)C | 5-(4-carboxy-3-methylbut-3-enyl)-5,6,8a-trimethyl-3-oxo-4a,6,7,8-tetrahydro-4H-naphthalene-1-carboxylic acid | **Diterpenoid** (Tricyclic, e.g., Abietane-type derivative) | 0.191 | 0.5 | [31] compound 105 |

| | | | | | | | | | |
|---|---|---|---|---|---|---|---|---|---|
| 168 | 15719 | 349.2009 | 9.22 | CC1CCC2(C(C1(C)CCC(=CC(=O)O)C)CC(=O)C=C2C(=O)O)C | 5-(4-carboxy-3-methylbut-3-enyl)-5,6,8a-trimethyl-3-oxo-4a,6,7,8-tetrahydro-4H-naphthalene-1-carboxylic acid | **Diterpenoid** (Tricyclic, e.g., Abietane-type derivative) | 0.081 | 0.5 | [31] compound 105 |
| 169 | 8470 | 333.206 | 6.808 | CC1CCC2(C(C1(C)CCC(C)CC(=O)O)CC(=O)C=C2C(=O)O)C | 5-(4-carboxy-3-methylbutyl)-5,6,8a-trimethyl-3-oxo-4a,6,7,8-tetrahydro-4H-naphthalene-1-carboxylic acid | **Diterpenoid** (Tricyclic, e.g., Abietane-type derivative) | 0.337 | 0.5 | |
| 170 | 8985 | 351.2167 | 6.966 | CC1CCC2(C(C1(C)CCC(C)CC(=O)O)CC(=O)C=C2C(=O)O)C | 5-(4-carboxy-3-methylbutyl)-5,6,8a-trimethyl-3-oxo-4a,6,7,8-tetrahydro-4H-naphthalene-1-carboxylic acid | **Diterpenoid** (Tricyclic, e.g., Abietane- | 0.393 | 0.5 | |

| | | | | | | | | | |
|---|---|---|---|---|---|---|---|---|---|
| | | | | | | type derivative) | | | |
| 171 | 9694 | 351.2166 | 7.177 | CC1CCC2(C(C1(C)CCC(C)CC(=O)O)CC(=O)C=C2C(=O)O)C | 5-(4-carboxy-3-methylbutyl)-5,6,8a-trimethyl-3-oxo-4a,6,7,8-tetrahydro-4H-naphthalene-1-carboxylic acid | **Diterpenoid** (Tricyclic, e.g., Abietane-type derivative) | 0.367 | 0.5 | |
| 172 | 11058 | 406.2589 | 7.667 | CC(=O)C1(CCC2C1(CCC3C2CCC4=C(C(=O)CCC34C)O)C)OC(=O)C | 5-(4-carboxy-3-methylbutyl)-5,6,8a-trimethyl-3-oxo-4a,6,7,8-tetrahydro-4H-naphthalene-1-carboxylic acid | **Diterpenoid** (Tricyclic, e.g., Abietane-type derivative) | 0.043 | 0.333 | |
| 173 | 11223 | 393.2273 | 7.736 | CC(=O)OC1C2C(CCCC23COC1(C45C3CC(C(C4)C(=C)C5O)O)O)(C)C | 5-(4-carboxy-3-methylbutyl)-5,6,8a-trimethyl-3-oxo-4a,6,7,8- | **Diterpenoid** (Tricyclic, e.g., | 0.175 | 0.5 | |

| | | | | | | | | | |
|---|---|---|---|---|---|---|---|---|---|
| | | | | | tetrahydro-4H-naphthalene-1-carboxylic acid | Abietane-type derivative) | | | |
| 174 | 11834 | 391.2108 | 7.877 | CC(=CCCC1=CCOC1=O)CCC=C(CCC=C(C)COC(=O)C)C(=O)O | 5-(4-carboxy-3-methylbutyl)-5,6,8a-trimethyl-3-oxo-4a,6,7,8-tetrahydro-4H-naphthalene-1-carboxylic acid | **Diterpenoid** (Tricyclic, e.g., Abietane-type derivative) | 0.063 | 0.5 | |
| 175 | 12018 | 470.311 | 7.975 | CCCCCC(C=CC1C(CC(C1CC=CCCC(=O)OC)OC(=O)C)O)OC(=O)C | 5-(4-carboxy-3-methylbutyl)-5,6,8a-trimethyl-3-oxo-4a,6,7,8-tetrahydro-4H-naphthalene-1-carboxylic acid | **Diterpenoid** (Tricyclic, e.g., Abietane-type derivative) | 0.056 | 0.333 | |
| 176 | 17918 | 273.2578 | 9.912 | CC(=CCO)CCC1C(=C)CCC2C1(CCCC2(C)C)C | 5-(5,5,8a-trimethyl-2-methylidene-3,4,4a,6,7,8- | **Diterpenoid Alcohol** | 0.744 | 0.5 | [32] |

| | | | | | | | | | |
|---|---|---|---|---|---|---|---|---|---|
| | | | | | hexahydro-1H-naphthalen-1-yl)-3-methylpent-2-en-1-ol/ (+)-copalol | (Bicyclic, e.g., Labdane-type derivative) | | | |
| 177 | 18177 | 289.2524 | 9.981 | CC1(CCCC2(C1CCC(=C)C2CCC(=CCO)CO)C)C | 5-(5,5,8a-trimethyl-2-methylidene-3,4,4a,6,7,8-hexahydro-1H-naphthalen-1-yl)-3-methylpent-2-en-1-ol | **Diterpenoid Alcohol** (Bicyclic, e.g., Labdane-type derivative) | 0.158 | 0.5 | [31] compound 105 |
| 178 | 14300 | 287.2369 | 8.799 | CC(=CC(=O)O)CCC1C(=C)CCC2C1(CCCC2(C)C)C | 5-(5,5,8a-trimethyl-2-methylidene-3,4,4a,6,7,8-hexahydro-1H-naphthalen-1-yl)-3-methylpent-2-enoic acid | **Diterpenoid (Bicyclic, e.g., Labdane-** | 0.206 | 0.5 | [31] compound 105 |

| | | | | | | | | | |
|---|---|---|---|---|---|---|---|---|---|
| | | | | | | **type derivative)** | | | |
| 179 | 14551 | 349.2 371 | 8.858 | CC(=CC(=O)OC)CCC1C(=C)CCC2 C1(CCCC2(C)C(=O)O)C | 5-(5-methoxy-3-methyl-5-oxopent-3-enyl)-1,4a-dimethyl-6-methylidene-3,4,5,7,8,8a-hexahydro-2H-naphthalene-1-carboxylic acid | **Methylated Diterpenoid** (Bicyclic, e.g., Labdane-type derivative) | 0.21 | 0.5 | [31] compound 99 |
| 180 | 15616 | 349.2 373 | 9.236 | CC(=CC(=O)OC)CCC1C(=C)CCC2 C1(CCCC2(C)C(=O)O)C | 5-(5-methoxy-3-methyl-5-oxopent-3-enyl)-1,4a-dimethyl-6-methylidene-3,4,5,7,8,8a-hexahydro-2H-naphthalene-1-carboxylic acid | **Methylated Diterpenoid** (Bicyclic, e.g., Labdane-type derivative) | 0.054 | 0.5 | [31] compound 99 |

| | | | | | | | | | |
|---|---|---|---|---|---|---|---|---|---|
| 181 | 10178 | 495.2567 | 7.308 | CC(C(C(CC(CC#CCOC1CCCCO1)OCOC)C(=O)OC)O)OC2CCCCO2 | 5,12-Dihydroxyicosa-6,8,10,14-tetraenedioic acid | **Eicosanoid** (Dicarboxylic fatty acid derivative) | 0.048 | 0 | |
| 182 | 9202 | 384.2376 | 7.003 | CC12CCC(C(C1C(C34C2CCC(C3)C(C4)(C)O)C(=O)O)(C)C(=O)O)O | 5,13-Dihydroxy-4,8,13-trimethyltetracyclo[10.2.1.01,9.03,8]pentadecane-2,4-dicarboxylic acid | **Diterpenoid** (Tetracyclic, complex rearrangement) | 0.131 | 0.333 | |
| 183 | 9626 | 386.2537 | 7.142 | CC1(C2C(O2)C3C1(C(CC45CC(C(C4O)CCC5C3(C)O)(C)O)O)O)C | 5,5,10,15-Tetramethyl-7-oxapentacyclo[12.2.1.01,11.04,9.06,8]heptadecane-3,4,10,15,17-pentol | **Diterpenoid** (Pentacyclic, e.g., Kaurane-type derivative) | 0.144 | 0.333 | |

| | | | | | | | | | |
|---|---|---|---|---|---|---|---|---|---|
| 184 | 14354 | 345.2059 | 8.785 | CC12CCCC(C1CCC(=C)C2CCC3=CC(OC3=O)OC)(C)C(=O)O | 5-[2-(2-methoxy-5-oxo-2H-furan-4-yl)ethyl]-1,4a-dimethyl-6-methylidene-3,4,5,7,8,8a-hexahydro-2H-naphthalene-1-carboxylic acid | **Methylated Diterpenoid** (Bicyclic, e.g., Labdane-type derivative with furanone) | 0.302 | 0.5 | [31] compound 66 |
| 185 | 14247 | 363.2165 | 8.76 | CC1CCC2(C(C1(C)CCC3C(C(OC3=O)OC)O)CCC=C2C(=O)O)C | 5-[2-(4-Hydroxy-5-methoxy-2-oxooxolan-3-yl)ethyl]-5,6,8a-trimethyl-3,4,4a,6,7,8-hexahydronaphthalene-1-carboxylic acid | **Methylated Diterpenoid** (Bicyclic, e.g., Labdane-type derivative | 0.273 | 0.5 | [31] compound 68 |

| | | | | | | | | | |
|---|---|---|---|---|---|---|---|---|---|
| | | | | | | with oxolanone) | | | |
| 186 | 15577 | 363.2165 | 9.211 | CC1CCC2(C(C1(C)CCC3C(C(OC3=O)OC)O)CCC=C2C(=O)O)C | 5-[2-(4-Hydroxy-5-methoxy-2-oxooxolan-3-yl)ethyl]-5,6,8a-trimethyl-3,4,4a,6,7,8-hexahydronaphthalene-1-carboxylic acid | **Methylated Diterpenoid** (Bicyclic, e.g., Labdane-type derivative with oxolanone) | 0.602 | 0.5 | [31] compound 68 |
| 187 | 15780 | 381.2273 | 9.306 | CC1CCC2(C(C1(C)CCC3C(C(OC3=O)OC)O)CCC=C2C(=O)O)C | 5-[2-(4-Hydroxy-5-methoxy-2-oxooxolan-3-yl)ethyl]-5,6,8a-trimethyl-3,4,4a,6,7,8-hexahydronaphthalene-1-carboxylic acid | **Methylated Diterpenoid** (Bicyclic, e.g., Labdane-type | 0.493 | 0.5 | [31] Compound 68 |

| | | | | | | | | | |
|---|---|---|---|---|---|---|---|---|---|
| | | | | | | derivative with oxolanone) | | | |
| 188 | 11552 | 414.285 | 7.824 | CCCCCCCCCCCCC(C1(C(C=CC1=O)OC(=O)C)O)OC(=O)C | 5-[2-(4-Hydroxy-5-methoxy-2-oxooxolan-3-yl)ethyl]-5,6,8a-trimethyl-3,4,4a,6,7,8-hexahydronaphthalene-1-carboxylic acidpicture see right | **Methylated Diterpenoid** (Bicyclic, e.g., Labdane-type derivative with oxolanone) | 0.153 | 0.063 | [31] compound 68 |
| 189 | 13589 | 349.2009 | 8.541 | CC(=CCCC1=CCOC1=O)CCC=C(CCC=C(C)CO)C(=O)O | 5-[3,4-dihydroxy-3-methyl-5-(3-methyl-5-oxofuran-2-ylidene)pentyl]-6-hydroxy-1,4a,6-trimethyl-3,4,5,7,8,8a-hexahydro-2H-naphthalene-1-carboxylic acid | **Sesterterpenoid** (Bicyclic, e.g., Decalin- | 0.034 | 0.5 | [31] compound 66 |

| | | | | | | | | | |
|---|---|---|---|---|---|---|---|---|---|
| | | | | | | based derivative with furanone) | | | |
| 190 | 14020 | 468.2955 | 8.684 | CC1=CC(=O)OC1=CC(C(C)(CCC2C3(CCCC(C3CCC2(C)O)(C)C(=O)O)C)O)O | 5-[3,4-dihydroxy-3-methyl-5-(3-methyl-5-oxofuran-2-ylidene)pentyl]-6-hydroxy-1,4a,6-trimethyl-3,4,5,7,8,8a-hexahydro-2H-naphthalene-1-carboxylic acid | **Sesterterpenoid** (Bicyclic, e.g., Decalin-based derivative with furanone) | 0.384 | 0.333 | [31] compound 66 |
| 191 | 14272 | 468.2954 | 8.781 | CC1=CC(=O)OC1=CC(C(C)(CCC2C3(CCCC(C3CCC2(C)O)(C)C(=O)O)C)O)O | 5-[3,4-dihydroxy-3-methyl-5-(3-methyl-5-oxofuran-2-ylidene)pentyl]-6-hydroxy-1,4a,6- | **Sesterterpenoid** (Bicyclic, e.g., | 0.14 | 0.333 | [31] compound 66 |

| | | | | | | | | | |
|---|---|---|---|---|---|---|---|---|---|
| | | | | | trimethyl-3,4,5,7,8,8a-hexahydro-2H-naphthalene-1-carboxylic acid | Decalin-based derivative with furanone) | | | |
| 192 | 15642 | 454.3162 | 9.192 | CC1=CC(=O)OC1=CC(C(C)(CCC2C3(CCC(C(C3CCC2(C)O)(C)C)O)C)O)O | 5-[5-(2,6-dihydroxy-2,5,5,8a-tetramethyl-3,4,4a,6,7,8-hexahydro-1H-naphthalen-1-yl)-2,3-dihydroxy-3-methylpentylidene]-4-methylfuran-2-one | **Sesterterpenoid** (Bicyclic, e.g., Decalin-based derivative with furanone) | 0.163 | 0.009 | [31] compound 66 |
| 193 | 11846 | 363.2165 | 7.926 | CC1CCC2(C(C1(C)CCC3=CC(OC3=O)OC)CC(C(C24CO4)O)O)C | 6-(6-Hydroxy-4-methylhex-4-enylidene)-2-(4-methylpent-3-enyl)hept-2-enedioic acid | **Diterpenoid** (Acyclic, but | 0.292 | 0.5 | |

| | | | | | | | | | |
|---|---|---|---|---|---|---|---|---|---|
| | | | | | | highly branched) | | | |
| 194 | 9266 | 331.1904 | 6.965 | CC12CCC(C(C1CCC=C2C(=O)O)(C)CCC3=CCOC3=O)CO | 6-(hydroxymethyl)-5,8a-dimethyl-5-[2-(5-oxo-2H-furan-4-yl)ethyl]-3,4,4a,6,7,8-hexahydronaphthalene-1-carboxylic acid | **Diterpenoid** (Bicyclic, e.g., Labdane-type derivative with furanone) | 0.196 | 0.5 | [31] compound 65 |
| 195 | 13717 | 305.2111 | 8.607 | CCCCCCCCCCC1=CC(=O)C(=C(C1=O)OC)OC | 6,14-Dimethyl-3-methylidene-2-oxo-3a,4,5,8,9,12,13,15a-octahydrocyclotetradeca[b]furan-10-carboxylic acid | **Diterpenoid** (Macrocyclic) | 0.017 | 0.5 | |
| 196 | 14172 | 313.1798 | 8.727 | CC1CCC(=C)C(=CC=CCC1(C)CCC2=CC(=O)OC2)C(=O)O | 6,14-Dimethyl-3-methylidene-2-oxo-3a,4,5,8,9,12,13,15a- | **Diterpenoid** (Macrocyclic) | 0.117 | 0.5 | |

| | | | | | | | | | |
|---|---|---|---|---|---|---|---|---|---|
| | | | | | octahydrocyclotetradeca[b]furan-10-carboxylic acid | | | | |
| 197 | 15248 | 620.3798 | 9.134 | CCC(C)C1C(C(CC2(O1)CC3CC(O2)CC=C(C(C(C=CC=C4COC5C4(C(C=C(C5O)C)C(=O)O3)O)C)O)C)O)C | 6'-Butan-2-yl-4',12,21,24-tetrahydroxy-5',11,13,22-tetramethylspiro[3,7,19-trioxatetracyclo[15.6.1.14,8.020,24]pentacosa-10,14,16,22-tetraene-6,2'-oxane]-2-one | **Triterpenoid/Steroid Glycoside** (Highly complex polycyclic structure, likely a saponin or similar) | 0.097 | 0.328 | |
| 198 | 12093 | 347.2216 | 7.976 | CC1(CCC2C3(CCCC(C3CCC2(C1)C=O)(C)C(=O)O)C)C(=O)OC | 6-Hydroxy-4-[[6-hydroxy-4-(hydroxymethyl)-2-(3-methylbut-2-enyl)hex-3-enoyl]oxymethyl]-2-(3-methylbut-2-enyl)hex-3-enoic acid | **Sesterterpenoid Ester** (Acyclic/Highly Branched) | 0.143 | 0.5 | |

| | | | | | | | | | |
|---|---|---|---|---|---|---|---|---|---|
| 199 | 9312 | 367.2115 | 7.032 | C1C2(CC3(CC1(CC(C2)(C3)O)O)O)C45CC6(CC(C4)(CC(C5)(C6)O)O)O | 7-(3,5,7-Trihydroxy-1-adamantyl)adamantane-1,3,5-triol | **Adamantane Derivative** (Complex polycyclic, but not a terpenoid) | 0.015 | 0.5 | |
| 200 | 10123 | 349.2008 | 7.316 | C(CCC(=O)O)CC=CCC(C=CC=CC=CC(CCCC(=O)O)O)O | 7-[2-(3,7-Dihydroxyoct-1-enyl)-5-oxocyclopent-3-en-1-yl]hept-5-enoic acid | **Eicosanoid** (Prostaglandin derivative) | 0.025 | 0.499 | |
| 201 | 10994 | 423.2376 | 7.724 | CC1C=CC2=CC(CC(C2C1CCC(CC(CC(=O)O)O)O)OC(=O)C(C)C(C)O)O | 7-[2-(3,7-Dihydroxyoct-1-enyl)-5-oxocyclopent-3-en-1-yl]hept-5-enoic acid | **Eicosanoid** (Prostaglandin derivative) | 0.06 | 0.5 | |
| 202 | 13815 | 351.2166 | 8.614 | CC(CCCC(C=CC1=C(C(=O)CC1)CC=CCCCC(=O)O)O)O | 7-[2-(3,7-Dihydroxyoct-1-enyl)-5-oxocyclopenten-1-yl]hept-5-enoic acid | **Eicosanoid** (Prostaglandin derivative) | 0.08 | 0.5 | |

| | | | | | | | | | |
|---|---|---|---|---|---|---|---|---|---|
| 203 | 18515 | 405.2637 | 10.028 | CC(C)(CCCCCOC1=CC(=CC=C1)OCCCCCC(C)(C)C(=O)O)C(=O)O | 7-[3-(6-Carboxy-6-methylheptoxy)phenoxy]-2,2-dimethylheptanoic acid | **Phenolic Ether/Fatty Acid Derivative** (Not a terpenoid) | 0.008 | 0.5 | |
| 204 | 13390 | 409.222 | 8.46 | CCC(C)C(=O)OCC1=C2C(C=C(C2C(OC1OC)OC(=O)C(C)CC)CO)OC | 8-(8-Acetyloxy-2,6-dimethylocta-2,6-dienoyl)oxy-2,6-dimethylocta-2,6-dienoic acid | **Diterpenoid Ester** (Acyclic/Irregular) | 0.029 | 0.5 | [31] Compound 279 |
| 205 | 11920 | 287.2005 | 7.932 | CC1CCC2C(C1(C)CCC(=CC(=O)O)C=O)CCC=C2C | 8,15-Dihydroxyicosa-5,9,11,13-tetraenoic acid | **Eicosanoid** (Polyunsaturated fatty acid derivative) | 0.119 | 0.5 | [31] Compound 279 |

| | | | | | | | | | |
|---|---|---|---|---|---|---|---|---|---|
| 206 | 9949 | 347.1853 | 7.263 | CC(=CCCC(=CC(=O)O)C)CCC=C(CCC=C(C)C(=O)O)C(=O)O | 8-Hydroperoxy-13-(3-pentyloxiran-2-yl)trideca-5,9,11-trienoic acid | **Eicosanoid** (Oxygenated fatty acid derivative) | 0.06 | 0.5 | [31] Compound 279 |
| 207 | 11327 | 365.1958 | 7.783 | CC=CC=CC(=O)OOC(C)(C)C=CC(C)(C)OOC(=O)C=CC=CC | CID 78384936 | **Galeopsin** | 0.025 | 0.5 | [31] Compound 76 |
| 208 | 19612 | 359.2216 | 10.3 | CC(=O)OC1(C(=O)CC2C(CCCC2(C1(CCC3=COC=C3)O)C)(C)C)C | CID 78384936 | **Galeopsin** | 0.613 | 0.5 | [31] Compound 76 |
| 209 | 18013 | 578.3691 | 9.901 | CC(=O)OC(C)(C)CCC(=O)C(C)(C1C(CC2(C1(CC(=O)C3(C2CC=C4C3CC(C(=O)C4(C)C)O)C)C)C)O)O | Cucurbitacin B, dihydro- | **Triterpenoid** (Specifically, a Cucurbitacin) | 0.099 | 0.333 | [31] compound 129 and 151 |

| | | | | | | | | | |
|---|---|---|---|---|---|---|---|---|---|
| 210 | 11229 | 359.2215 | 7.787 | CC(=O)OC1(C(=O)CC2C(CCCC2(C1(CCC3=COC=C3)O)C)(C)C)C | Methyl 2-(11-acetyloxy-2,7-dihydroxy-2,8,12-trimethyl-15-oxabicyclo[10.2.1]pentadec-8-en-5-yl)prop-2-enoate | **Diterpenoid Ester** (Bicyclic, e.g., Taxane-type or similar) | 0.828 | 0.5 | |
| 211 | 11458 | 461.2145 | 7.814 | CC1CC(C(=O)C2(C1CC3C4(C2C(C(C(C4(CC(=O)O3)O)C)OC)OC(=O)C)C)C)O | Methyl 2-(2-acetyloxy-12-hydroxy-1,5,12-trimethyl-6,16-dioxatricyclo[11.2.1.05,7]hexadecan-9-yl)prop-2-enoate | **Diterpenoid Ester** (Tricyclic, with ether bridges) | 0.081 | 1 | |
| 212 | 12045 | 337.2009 | 7.975 | CC(=CCOC(=O)C)CC(C=C(C)C(CC=C(C)COC(=O)C)O)O | Methyl 2-(2-acetyloxy-12-hydroxy-1,5,12-trimethyl-6,16-dioxatricyclo[11.2.1.05,7]hexadecan-9-yl)prop-2-enoate | **Diterpenoid Ester** (Tricyclic, with ether bridges) | 0.011 | 0.5 | |

| | | | | | | | | | |
|---|---|---|---|---|---|---|---|---|---|
| 213 | 14340 | 364.2481 | 8.791 | CC(=CCCC(=CCCC(=CCCC1=CCOC1=O)C)C(=O)OC)C | methyl 6-methyl-2-(4-methylpent-3-enyl)-9-(5-oxo-2H-furan-4-yl)nona-2,6-dienoate | **Sesterterpenoid Ester** (Irregular, but related to sesterterpenoids) | 0.138 | 0.333 | |
| 214 | 12039 | 470.2746 | 7.991 | CC(=O)OC1CC23CC(C(C2OC(=O)C)CCC3C(C4C1(C(C5C4O5)(C)C)O)(C)O)(C)O | Methyl 7-[5-acetyloxy-2-(3-acetyloxyoct-1-enyl)-3-hydroxycyclopentyl]hept-5-enoate | **Eicosanoid Ester** (Prostaglandin derivative) | 0.073 | 0.333 | |
| 215 | 8873 | 463.3251 | 6.894 | CCCCCCCCCC(=O)OCCOCCOCCOCCOCCOCCOCCO | PEG7 Monodecanoate | **Polyether Ester** (Not a terpenoid) | 0.029 | 0.285 | |
| 216 | 4947 | 428.264 | 5.49 | CC(=O)OC1CC23CC(C(C2O)CCC3C(C4C1(C(C5C4O5)(C)C)O)(C)O)(C)O | Rhodojaponin-II | **Diterpenoid** (Specifically, a Grayanane- | 0.368 | 0.333 | |

| | | | | | | | | | |
|---|---|---|---|---|---|---|---|---|---|
| | | | | | | type diterpenoid) | | | |
| 217 | 9111 | 428.2642 | 6.998 | CC(=O)OC1CC23CC(C(C2O)CCC3C(C4C1(C(C5C4O5)(C)C)O)(C)O)(C)O | Rhodojaponin-II | **Diterpenoid** (Specifically, a Grayanane-type diterpenoid) | 0.377 | 0.332 | |
| 218 | 9659 | 428.2643 | 7.184 | CC(=O)OC1CC23CC(C(C2O)CCC3C(C4C1(C(C5C4O5)(C)C)O)(C)O)(C)O | Rhodojaponin-II | **Diterpenoid** (Specifically, a Grayanane-type diterpenoid) | 0.239 | 0.333 | |
| 219 | 10251 | 426.2485 | 7.414 | CC1C=C(C(=O)C2(C1CC3C4(C2C(C(C(C4(CC(=O)O3)O)C)OC)O)C)C)OC | Rhodojaponin-II | **Diterpenoid** (Specifically, a | 0.054 | 0.333 | |

| | | | | | | | | | |
|---|---|---|---|---|---|---|---|---|---|
| | | | | | | Grayanane-type diterpenoid) | | | |
| 220 | 11782 | 361.2009 | 8 | CC1(CCCC2(C1CC(C3=CC4=C(C(=O)OC4(C(C32)O)O)CO)OC)C)C | Rhodojaponin-II | **Diterpenoid** (Specifically, a Grayanane-type diterpenoid) | 0.156 | 0.5 | |
| 221 | 12121 | 454.2799 | 7.916 | CC(=O)OC1CC2CC3(C1C4(C(CC3OC(=O)C)C(C(CC4O)O)(C)C)C)C(C2=C)O | Rhodojaponin-II | **Diterpenoid** (Specifically, a Grayanane-type diterpenoid) | 0.032 | 0.333 | |

| | | | | | | | | | |
|---|---|---|---|---|---|---|---|---|---|
| 222 | 11059 | 405.2257 | 7.724 | CC(C)COC1=CC=C(C=C1)COC2C(OC3C2OC(O3)(C)C)C4COC(O4)(C)C | Tert-butyl-dimethyl-[(3,3,6,6-tetramethoxy-2,4,5-trimethylcyclohexa-1,4-dien-1-yl)methoxy]silane | **Organosilane/Quinone Derivative** (Not a terpenoid) | 0.008 | 0 | |
| 223 | 13640 | 454.2799 | 8.642 | CC(=O)OC1CC2C(C(CC(C2(C3C14CC(CC3O)C(=C)C4O)C)OC(=O)C)O)(C)C | Tetracyclopentyl methanetetracarboxylate | **Ester/Cyclic Ester** (Not a terpenoid) | 0.119 | 0.333 | |
| 224 | 9161 | 425.2171 | 6.979 | CCOC(=O)C1(CC=CCC(CC=CC1)(C(=O)OCC)C(=O)OCC)C(=O)OCC | Tetraethyl cyclodeca-3,8-diene-1,1,6,6-tetracarboxylate | **Macrocyclic Ester** (Not a terpenoid) | 0.037 | 0.5 | |
| 225 | 14119 | 455.2638 | 8.719 | CC(C)(C)OC(=O)C=C(C(=CC(=O)OC(C)(C)C)C(=O)OC(C)(C)C)C(=O)OC(C)(C)C | Tetratert-butyl buta-1,3-diene-1,2,3,4-tetracarboxylate | **Aliphatic Ester** (Not a terpenoid) | 0.026 | 0.5 | |
| 226 | 11959 | 443.2832 | 7.955 | CCCCCCO[Si](OCCCCCC)(OCCCCCC)OC1CC(=O)OC(=O)C1 | Trimethyl 1,9,13-trimethyltetradeca-1,5,9,13-tetraene-1,5,14-tricarboxylate | **Diterpenoid Ester** (Acyclic) | 0.019 | 0.001 | |

| | | | | | | | | | |
|---|---|---|---|---|---|---|---|---|---|
| 227 | 13880 | 429.2249 | 8.63 | CC(=CCCC(=CC(=O)OC)C)CCC=C(CCC=C(C)C(=O)OC)C(=O)OC | Trimethyl 1,9,13-trimethyltetradeca-1,5,9,13-tetraene-1,5,14-tricarboxylate | **Diterpenoid Ester** (Acyclic) | 0.113 | 1 | |
| 228 | 14525 | 389.2322 | 8.819 | CC(=CCCC(=CC(=O)OC)C)CCC=C(CCC=C(C)C(=O)OC)C(=O)OC | Trimethyl 1,9,13-trimethyltetradeca-1,5,9,13-tetraene-1,5,14-tricarboxylate | **Diterpenoid Ester** (Acyclic) | 0.054 | 0.5 | |
| 229 | 15073 | 484.3263 | 9.073 | CC1(CCC2C(=CCC3C2(CCC(C3(C)COC4C(C(C(C(O4)CO)O)O)O)O)C)C1)C=C | Virescenoside B | **Diterpenoid Glycoside** (Specifically, a Fusicoccane-type or similar) | 0.104 | 0.333 | |
| 230 | 4167 | 395.2428 | 5.148 | CC(=O)OC1C2CCC3C1(CC4C5(C(C3(O4)C)CC(C5(C)C)O)O)CC2(C)O | | | 0.267 | 0.5 | |

| | | | | | | | | | |
|---|---|---|---|---|---|---|---|---|---|
| 231 | 4483 | 347.1851 | 5.345 | CC(=CCCC(=CC(=O)O)C)CCC=C(CCC=C(C)C(=O)O)C(=O)O | | | 0.056 | 0.5 | |
| 232 | 4900 | 411.2374 | 5.458 | CC1C(C(C2C(CCC(C2(C13C(=O)CC(O3)(C)C=C)C)O)(C)C)O)(O)OC(=O)C | | | 0.288 | 0.5 | |
| 233 | 8822 | 444.2593 | 6.902 | CC(=O)OCC1(C2CCC3C(C2)(C1O)C4(C(C5C3(CO4)C(CCC5(C)C)O)O)O)O | | | 0.094 | 0.333 | |
| **Flavonoids** | | | | | | | | | |
| S.NO | Cluster Index | parent mass | Retention Time In Minutes | SMILES | Compound IUPAC Name | Final classification | Confidence Score Exact | Sirius Score Normalized | Reference of derivative |
| 1 | 8420 | 569.1075 | 6.734 | C=CC(=O)OCOC1=CC=C(C=C1)C(=O)OC2=CC=C(C=C2)C(=O)OC3=CC=C(C=C3)C(=O)OCOC(=O)C=C | [4-[4-(Prop-2-enoyloxymethoxycarbonyl)phenoxy]carbonylphenyl] 4-(prop-2-enoyloxymethoxy)benzoate | **Complex Aromatic Ester** | 0.213 | 0.999 | |

| | | | | | | | | | |
|---|---|---|---|---|---|---|---|---|---|
| 2 | 15455 | 373.1777 | 9.151 | C=CCOC1=CC=C(C=C1)C=C2CCC(=CC3=CC=C(C=C3)OCC=C)C2=O | | | 0.185 | 0.043 | |
| 3 | 3016 | 303.0499 | 4.49 | C1=C(C=C(C(=C1O)O)O)C2C(C(=O)C3=C(C=C(C=C3O2)O)O)O | 3,5,7-Trihydroxy-2-(3,4,5-trihydroxyphenyl)chroman-4-one | **Flavonoid** (Flavonol, specific type of polyphenol) | 0.259 | 0.499 | [31] compound 216 |
| 4 | 3986 | 303.0499 | 4.951 | C1=C(C=C(C(=C1O)O)O)C2C(C(=O)C3=C(C=C(C=C3O2)O)O)O | 3,5,7-Trihydroxy-2-(3,4,5-trihydroxyphenyl)chroman-4-one | **Flavonoid** (Dihydroflavonol, specific type of polyphenol) | 0.147 | 0.099 | [31] compound 216 |
| 5 | 2974 | 301.0343 | 4.45 | C1=C(C=C2C(=C1O)C(=O)C(=C(O2)C3=CC(=C(C=C3O)O)O)O)O | 5'-Hydroxymorin | **Flavonoid** (Flavonol, specific type | 0.121 | 0.5 | [31] compound 210 |

| | | | | | | | | | |
|---|---|---|---|---|---|---|---|---|---|
| | | | | | | of polyphenol) | | | |
| 6 | 2975 | 325.0917 | 4.466 | C1=CC(=C(C=C1C=CC(=O)OC2C(C(C(C(O2)CO)O)O)O)O)O | [3,4,5-Trihydroxy-6-(hydroxymethyl)oxan-2-yl] 3-(3,4-dihydroxyphenyl)prop-2-enoate | **Phenylpropanoid Glycoside** (Specifically, a Caffeoyl Glycoside) | 0.117 | 0.486 | [31] compound 241 |
| 7 | 4290 | 775.2074 | 5.175 | C1=CC(=C(C=C1C=CC(=O)OCC2C(C(C(C(O2)OC3=C(OC4C=C(C=C(C4=C3)OC5C(C(C(C(O5)CO)O)O)O)O)O)C6=CC(=C(C=C6)O)O)O)O)O)O)O | [6-[[2-(3,4-dihydroxyphenyl)-7-hydroxy-5-[3,4,5-trihydroxy-6-(hydroxymethyl)oxan-2-yl]oxy-8aH-chromen-3-yl]oxy]-3,4,5-trihydroxyoxan-2-yl]methyl 3-(3,4-dihydroxyphenyl)prop-2-enoate | **Flavonoid Glycoside Ester** (Complex flavonoid with sugar and ester linkages, not a terpenoid) | 0.193 | 0.458 | [31] compound 223 |

| | | | | | | | | | |
|---|---|---|---|---|---|---|---|---|---|
| 8 | 4534 | 775.2075 | 5.274 | C1=CC(=C(C=C1C=CC(=O)OCC2C(C(C(C(O2)OC3=C(OC4C=C(C=C(C4=C3)OC5C(C(C(C(O5)CO)O)O)O)O)C6=CC(=C(C=C6)O)O)O)O)O)O)O | [6-[[2-(3,4-dihydroxyphenyl)-7-hydroxy-5-[3,4,5-trihydroxy-6-(hydroxymethyl)oxan-2-yl]oxy-8aH-chromen-3-yl]oxy]-3,4,5-trihydroxyoxan-2-yl]methyl 3-(3,4-dihydroxyphenyl)prop-2-enoate | **Flavonoid Glycoside Ester** (Complex flavonoid with sugar and ester linkages, not a terpenoid) | 0.136 | 0.481 | [31] compound 223 |
| 9 | 3025 | 465.1026 | 4.496 | C1=CC(=C(C=C1C2=C(C(=O)C3=C(C=C(C=C3O2)O)O)OC4C(C(C(C(O4)CO)O)O)O)O)O | ISOquercitin/ other name 2-(3,4-dihydroxyphenyl)-5,7-dihydroxy-3-[(2S,3R,4S,5S,6R)-3,4,5-trihydroxy-6-(hydroxymethyl)oxan-2-yl]oxychromen-4-one | **Flavonoid Glycoside** (Specifically, Quercetin 3-O-glucoside) | 0.666 | 0.498 | [31] compound 220 or 221 |

| | | | | | | | | | |
|---|---|---|---|---|---|---|---|---|---|
| 10 | 3056 | 487.0847 | 4.5 | C1=CC(=C(C=C1C2=C(C(=O)C3=C(C=C(C=C3O2)O)O)OC4C(C(C(C(O4)CO)O)O)O)O)O | ISOquercitin/ other name 2-(3,4-dihydroxyphenyl)-5,7-dihydroxy-3-[(2S,3R,4S,5S,6R)-3,4,5-trihydroxy-6-(hydroxymethyl)oxan-2-yl]oxychromen-4-one | **Flavonoid Glycoside** (Specifically, Quercetin 3-O-glucoside) | 0.522 | 0.991 | [31] compound 220 or 221 |
| 11 | 3440 | 449.108 | 4.657 | C1=CC(=C(C=C1C2=CC(=O)C3=C(C=C(C=C3O2)OC4C(C(C(C(O4)CO)O)O)O)O)O)O | Luteolin 7-galactoside | **Flavonoid Glycoside** (Specifically, Luteolin 7-O-galactoside) | 0.227 | 0.059 | [31] compound 207 |
| 12 | 3581 | 493.0973 | 4.741 | C1=CC(=C(C=C1C2=CC(=O)C3=C(C=C(C=C3O2)OCOC(=O)C4C(C(C(C(O4)O)O)O)O)O)O)O | | | 0.219 | 0.232 | |

| | | | | | | | | | |
|---|---|---|---|---|---|---|---|---|---|
| 13 | 3989 | 465.1034 | 4.981 | C1=CC(=C(C=C1C2=CC(=O)C3=C(O2)C=C(C(=C3O)OC4C(C(C(C(O4)CO)O)O)O)O)O)O | 2-(3,4-Dihydroxyphenyl)-5,7-dihydroxy-6-[3,4,5-trihydroxy-6-(hydroxymethyl)oxan-2-yl]oxychromen-4-one | **Flavonoid Glycoside** (Specifically, a Quercetin derivative or Isorhamnetin derivative) | 0.046 | 0.127 | [31] compound 211 |
| 14 | 3785 | 271.0602 | 4.873 | C1=CC(=C(C=C1O)O)C(=CC(=O)C2=C(C=C(C=C2)O)O)O | 1,3-Bis(2,4-dihydroxyphenyl)-3-hydroxyprop-2-en-1-one | **Chalcone** (A precursor to flavonoids, type of polyphenol) | 0.019 | 0.488 | [31] compound 246 |
| 15 | 9354 | 725.1856 | 7.004 | C1=CC(=CC=C1C=CC(=O)OCC2C(C(C(C(O2)OC3=CC=C(C=C3)C4=CC(=O)C5=C(C=C(C=C5O4)O)O)O)OC(=O)C=CC6=CC=C(C=C6)O)O)O)O | [6-[4-(5,7-Dihydroxy-4-oxochromen-2-yl)phenoxy]-3,4-dihydroxy-5-[3-(4-hydroxyphenyl)prop-2- | **Flavonoid Glycoside Ester** (Complex flavonoid | 0.298 | 0.5 | [31] compound 214 |

| | | | | | | | | | |
|---|---|---|---|---|---|---|---|---|---|
| | | | | | enoyloxy]oxan-2-yl]methyl 3-(4-hydroxyphenyl)prop-2-enoate | with sugar and ester linkages, not a terpenoid) | | | |
| 16 | 3596 | 449.1077 | 4.757 | C1=CC(=CC=C1C2=C(C(=O)C3=C(C=C(C=C3O2)O)O)OC4C(C(C(C(O4)CO)O)O)O)O | 5,7-Dihydroxy-2-(4-hydroxyphenyl)-3-[3,4,5-trihydroxy-6-(hydroxymethyl)oxan-2-yl]oxychromen-4-one | **Flavonoid Glycoside** (Specifically, Kaempferol 3-O-glucoside) | 0.408 | 0.5 | [31] compound 220 221 |
| 17 | 4352 | 283.06 | 5.208 | C1=CC(=CC=C1C2=CC(=O)C3=C(O2)C=C(C(=C3O)CO)O)O | Other name 6-hydroxymethyl**apigenin**.<br><br>5,7-Dihydroxy-6-(hydroxymethyl)-2-(4-hydroxyphenyl)chromen-4-one | **Flavonoid** (Specifically, a C-methylated Apigenin derivative) | 0.173 | 0.008 | [31] compound 205 |

| | | | | | | | | | |
|---|---|---|---|---|---|---|---|---|---|
| 18 | 9049 | 271.0601 | 6.927 | C1=CC=C(C(=C1)C(=O)C=C(C2=C(C(=C(C=C2)O)O)O)O)O | 3-Hydroxy-1-(2-hydroxyphenyl)-3-(2,3,4-trihydroxyphenyl)prop-2-en-1-one | **Chalcone** (A precursor to flavonoids, type of polyphenol) | 0.049 | 0.007 | [31] compound 246 |
| 19 | 2955 | 283.06 | 4.518 | C1=CC=C(C=C1)C2=CC(=O)C3=C(O2)C=C(C(=C3O)C=O)O | 5,7-Dihydroxy-4-oxo-2-phenylchromene-6-carbaldehyde | **Flavonoid** (Flavone derivative) | 0.288 | 0.492 | [31] compound 209 |
| 20 | 3439 | 435.0917 | 4.736 | C1C(C(C(C(O1)OC2=C(OC3=CC(=CC(=C3C2=O)O)O)C4=CC(=C(C=C4)O)O)O)O)O | Reynoutrin | **Flavonoid Glycoside** (Specifically, Quercetin 3-O-rhamnoside) | 0.515 | 0.5 | [31] compound 217 |
| 21 | 10247 | 745.1059 | 7.338 | C1C(C(C(OC1C(=O)O)OC2C(C(C(OC2C(=O)O)OC3C(C(C(OC3C(=O) | | | 0.021 | 0.227 | |

| | | | | | | | | | |
|---|---|---|---|---|---|---|---|---|---|
| | | | | O)OC4C(C(C(OC4C(=O)O)O)O)O)O)O)O)O)O)O | | | | | |
| 22 | 4338 | 316.0815 | 5.193 | C1OC2=C(O1)C(=C3C(=C2)OC(=CC3=O)C4=CC=C(C=C4)O)O | Kanzakiflavone 2 | **Flavonoid** (Specifically, a Flavone derivative) | 0.336 | 0.333 | [31] compound 252 |
| 23 | 4346 | 356.1492 | 5.197 | CC(=CCC1=C(C2=C(C=C1O)OC=C(C2=O)C3=CC=C(C=C3)O)O)C | Wighteone | **Isoflavonoid** (Specifically, a Prenylated Isoflavone) | 0.326 | 0.328 | [31] compound 223 |
| 24 | 10951 | 765.311 | 7.636 | CC(=O)OC1C(C(C(OC1OC2C(C(C(OC2OCC=C)C(=O)OC)OOC)OOC)COCC3=CC=CC=C3)OCC4=CC=CC=C4)OCC5=CC=CC=C5 | | | 0.244 | 0.285 | |

| | | | | | | | | | |
|---|---|---|---|---|---|---|---|---|---|
| 25 | 2783 | 377.0866 | 4.345 | CC(=O)OC1C(OC2=C(C1=O)C(=C(C(=C2)O)OC)O)C3=CC(=C(C=C3)O)O | [2-(3,4-Dihydroxyphenyl)-5,7-dihydroxy-6-methoxy-4-oxo-2,3-dihydrochromen-3-yl] acetate | **Flavonoid Acetate** (Dihydroflavonol derivative) | 0.36 | 0.5 | [31] compound 221 |
| 26 | 15747 | 633.1598 | 9.227 | CC(=O)OCC1C(OC2=C(O1)C=CC(=C2)C3=CC(=O)C4=C(O3)C=C(C=C4OC(=O)C)OC(=O)C)C5=CC(=C(C=C5)OC(=O)C)OC | | | 0.141 | 0.498 | |
| 27 | 9944 | 783.3218 | 7.228 | CC(C12COC(=O)C=CC1C3(C=CC4(C(C3C(C2OC(=O)C)OC(=O)C)(C(C(C5(C4(CC(=C)C6C5(O6)C(=O)OC)O)C)OC(=O)C)OC(=O)C)C)C)C)OC(=O)C | | | 0.079 | 0.498 | |
| 28 | 10475 | 783.3224 | 7.427 | CC(C12COC(=O)C=CC1C3(C=CC4(C(C3C(C2OC(=O)C)OC(=O)C)(C(C(C5(C4(CC(=C)C6C5(O6)C(=O)O | | | 0.069 | 0.452 | |

| | | | | | | | | | |
|---|---|---|---|---|---|---|---|---|---|
| | | | | C)O)C)OC(=O)C)OC(=O)C)C)C)C)OC(=O)C | | | | | |
| 29 | 5015 | 559.1442 | 5.468 | CC(CC(=O)O)(CC(=O)OCC1C(C(C(C(O1)OC2=CC(=C3C(=C2)OC(=CC3=O)C4=CC=C(C=C4)O)O)O)O)O)O | Chamaemeloside | **Flavonoid Glycoside** (Specifically, Apigenin 7-O-glucoside) | 0.882 | 0.413 | [31] compound 214 |
| 30 | 13312 | 809.3369 | 8.443 | CC=C(C)C(=O)OC1CC(=CCCC(=CC2C1C(=C)C(=O)O2)C)C(=O)OC(C)C(C)(C(=O)OC3C4C(C=C(CCC=C(C3OC(=O)C)C(=O)OC)C)OC(=O)C4=C)O | | | 0.134 | 0.493 | |
| 31 | 9946 | 807.3222 | 7.228 | CC1=C2C(C(=C(C(CC(C(=CC(C(C2(C)C)CC1OC(=O)C)OC(=O)C)COC(=O)C)OC(=O)C(=CC3=CC=CC=C3)OC(=O)C)OC(=O)C)C)OC(=O)C)OC(=O)C | [2,7,9,10,13-Pentaacetyloxy-4-(acetyloxymethyl)-8,12,15,15-tetramethyl-5-bicyclo[9.3.1]pentadeca-3,8,11- | **Diterpenoid Ester** (Highly acetylated, complex | 0.019 | 0.497 | |

| | | | | | | | | | |
|---|---|---|---|---|---|---|---|---|---|
| | | | | | trienyl] 2-acetyloxy-3-phenylprop-2-enoate | bicyclic, often a Taxane-type derivative. Not a typical terpenoid, but still falls under diterpenoid classification due to C20 skeletal origin.) | | | |
| 32 | 3599 | 530.1655 | 4.757 | CC1=CC(=CC(=C1C(=O)O)O)OC(=O)C2=C(C=C(C=C2C)OC(=O)C3=C(C(=C(C=C3C)O)OC)OC)O | 2-Hydroxy-4-[2-hydroxy-4-(4-hydroxy-2,3-dimethoxy-6-methylbenzoyl)oxy-6-methylbenzoyl)oxy-6- | **Polyphenol Ester** (Complex benzoic acid | 0.39 | 0.31 | [31] compound 270 |

| | | | | | | | | | |
|---|---|---|---|---|---|---|---|---|---|
| | | | | | methylbenzoyl]oxy-6-methylbenzoic acid | ester, not a terpenoid) | | | |
| 33 | 4252 | 338.1387 | 5.152 | CC1=CC2=C(C=C1)OC3=CC4=C(C=CC(=C4C=C3C2=O)OC)OC | 7,10-Dimethoxy-2-methylbenzo[b]xanthen-12-one | **Xanthone Derivative** (Not a terpenoid) | 0.218 | 0.321 | |
| 34 | 4494 | 819.2247 | 5.258 | CC1=CCOC(=O)C(CC(=O)OCC2=C3CC4C(C5CC5C4(COC1=O)O)(C6C3(C7C(=C(C)C(=O)OC)C(=O)C(C8(C7(C(=C6)C9C8C9)OO)C)O)OC2=O)C)O | Methyl 2-(37-hydroperoxy-9,18,30-trihydroxy-14,22,29-trimethyl-3,7,10,15,31-pentaoxo-2,6,11,16-tetraoxanonacyclo[16.15.3.125,29.01,23.04,34.019,21.022,36.026,28.033,37]heptatriaconta-4(34),13,24-trien-32-ylidene)propanoate | **Complex Macrolide/Polyketide** (Not a terpenoid) | 0.058 | 0.923 | |

| | | | | | | | | | |
|---|---|---|---|---|---|---|---|---|---|
| 35 | 9798 | 689.1488 | 7.194 | CC1C(C(C(C(O1)OC2=C(OC3=CC(=C(C(=C3C2=O)O)OC)OC4C(C(C(C(O4)C)OC(=O)C)O)O)C5=CC=C(C=C5)O)O)O)O | [4,5-Dihydroxy-6-[5-hydroxy-2-(4-hydroxyphenyl)-6-methoxy-4-oxo-3-(3,4,5-trihydroxy-6-methyloxan-2-yl)oxychromen-7-yl]oxy-2-methyloxan-3-yl] acetate | **Flavonoid Glycoside Acetate** (Complex flavonoid with sugar and ester linkages, not a terpenoid) | 0.306 | 0.989 | [31] compound 211 |
| 36 | 3121 | 595.1649 | 4.551 | CC1C(C(C(C(O1)OC2=CC(=C3C(=C2)OC(=C(C3=O)OC4C(C(C(C(O4)C)O)O)O)C5=CC(=C(C=C5)O)O)O)O)O)O | Quercetin 3,7-dirhamnoside | **Flavonoid Glycoside** (Specifically, a Quercetin glycoside) | 0.587 | 0.312 | [31] compound 220 221 |

| | | | | | | | | | |
|---|---|---|---|---|---|---|---|---|---|
| 37 | 3483 | 579.1706 | 4.692 | CC1C(C(C(C(O1)OC2=CC(=C3C(=C2)OC(=C(C3=O)OC4C(C(C(C(O4)C)O)O)O)C5=CC=C(C(=C5)O)O)O)O)O)O | Lespedin;Lespenephryl | **Flavonoid** (Specific methylated flavonoid) | 0.529 | 0.496 | [31] compound 223 |
| 38 | 3340 | 595.1649 | 4.618 | CC1C(C(C(C(O1)OC2C(C(C(OC2OC3=C(OC4=CC(=CC(=C4C3=O)O)O)C5=CC=C(C=C5)O)CO)O)O)O)O)O | Kaempferol-3-O-glucorhamnoside | **Flavonoid Glycoside** (Specifically, a Kaempferol glycoside, i.e., Kaempferitrin) | 0.369 | 0.5 | [31] compound 218 |
| 39 | 3664 | 579.1705 | 4.806 | CC1C(C(C(C(O1)OC2C(C(C(OC2OC3=CC(=C4C(=O)CC(OC4=C3)C5=CC(=C(C=C5)O)O)O)CO)O)O)O)O)O | Eriodictyol-7-O-neohesperidoside | **Flavonoid Glycoside** (Specifically, a | 0.233 | 0.492 | [31] compound 222 |

| | | | | | | | | | |
|---|---|---|---|---|---|---|---|---|---|
| | | | | | | Hesperidin-like flavonoid glycoside) | | | |
| 40 | 3994 | 791.2025 | 4.993 | CC1C(C(C(C(O1)OC2C(C(COC2OC3=C(OC4=CC(=CC(=C4C3=O)O)O)C5=CC(=C(C=C5)O)O)OC(=O)C)OC(=O)C)OC(=O)C)OC(=O)C)OC(=O)C | | | 0.455 | 0.498 | |
| 41 | 2808 | 611.16 | 4.343 | CC1C(C(C(C(O1)OCC2C(C(C(C(O2)OC3=C(OC4=CC(=CC(=C4C3=O)O)O)C5=CC(=C(C=C5)O)O)O)O)O)O)O)O | Vitamin P | **Flavonoid** (General term) | 0.814 | 0.5 | [31] compound 227 |
| 42 | 4492 | 817.217 | 5.238 | CC1C(C(C(C(O1)OCC2C(C(C(C(O2)OC3=C(OC4=CC(=CC(=C4C3=O)O)O)C5=CC(=C(C=C5)O)O)OC(= | [2-[2-(3,4-Dihydroxyphenyl)-5,7-dihydroxy-4-oxochromen-3-yl]oxy-4,5-dihydroxy-6-[(3,4,5-trihydroxy-6-methyloxan-2- | **Flavonoid Glycoside Ester** (Complex | 0.798 | 0.493 | [31] compound 237 |

| | | | | | | | | | |
|---|---|---|---|---|---|---|---|---|---|
| | | | | O)C=CC6=CC(=C(C(=C6)OC)O)OC)O)O)O)O)O | yl)oxymethyl]oxan-3-yl] 3-(4-hydroxy-3,5-dimethoxyphenyl)prop-2-enoate | flavonoid with sugar and ester linkages, not a terpenoid) | | | |
| 43 | 4568 | 787.2073 | 5.29 | CC1C(C(C(C(O1)OCC2C(C(C(C(O2)OC3=C(OC4=CC(=CC(=C4C3=O)O)O)C5=CC(=C(C=C5)O)O)OC(=O)C=CC6=CC(=C(C=C6)O)OC)O)O)O)O)O | [2-[2-(3,4-Dihydroxyphenyl)-5,7-dihydroxy-4-oxochromen-3-yl]oxy-4,5-dihydroxy-6-[(3,4,5-trihydroxy-6-methyloxan-2-yl)oxymethyl]oxan-3-yl] 3-(4-hydroxy-3-methoxyphenyl)prop-2-enoate | **Flavonoid Glycoside Ester** (Complex flavonoid with sugar and ester linkages, not a terpenoid) | 0.486 | 0.487 | [31] compound 234 |

| | | | | | | | | | |
|---|---|---|---|---|---|---|---|---|---|
| 44 | 4531 | 625.1761 | 5.274 | CC1C(C(C(C(O1)OCC2C(C(C(C(O2)OC3=C(OC4=CC(=CC(=C4C3=O)O)OC)C5=CC(=C(C=C5)O)O)O)O)O)O)O)O | 2-(3,4-Dihydroxyphenyl)-5-hydroxy-7-methoxy-3-[3,4,5-trihydroxy-6-[(3,4,5-trihydroxy-6-methyloxan-2-yl)oxymethyl]oxan-2-yl]oxychromen-4-one | **Flavonoid Glycoside** (Complex glycosylated flavonoid, not a terpenoid) | 0.405 | 0.5 | [31] compound 227 |
| 45 | 2435 | 757.2177 | 4.085 | CC1C(C(C(C(O1)OCC2C(C(C(C(O2)OC3=C(OC4=CC(=CC(=C4C3=O)O)OC5C(C(C(C(O5)C)O)O)O)C6=CC(=C(C=C6)O)O)O)O)O)O)O)O | 2-(3,4-Dihydroxyphenyl)-5-hydroxy-7-(3,4,5-trihydroxy-6-methyloxan-2-yl)oxy-3-[3,4,5-trihydroxy-6-[(3,4,5-trihydroxy-6-methyloxan-2-yl)oxymethyl]oxan-2-yl]oxychromen-4-one | **Flavonoid Glycoside** (Complex glycosylated flavonoid, not a terpenoid) | 0.702 | 0.5 | [31] compound 227 |

| | | | | | | | | | |
|---|---|---|---|---|---|---|---|---|---|
| 46 | 3600 | 777.1861 | 4.757 | CC1C(C(C(C(O1)OCC2C(C(C(C(O2)OC3=CC(=C4C(=C3)OC(=CC4=O)C5=CC=C(C=C5)O)O)OC6C(C(C(C(O6)C(=O)O)O)O)O)O)O)O)O)O | 6-[4,5-Dihydroxy-2-[5-hydroxy-2-(4-hydroxyphenyl)-4-oxochromen-7-yl]oxy-6-[(3,4,5-trihydroxy-6-methyloxan-2-yl)oxymethyl]oxan-3-yl]oxy-3,4,5-trihydroxyoxane-2-carboxylic acid | **Flavonoid Diglycoside Carboxylic Acid** (Complex glycosylated flavonoid, not a terpenoid) | 0.643 | 0.073 | [31] compound 213 |
| 47 | 3706 | 479.1183 | 4.824 | COC1=C(C=C2C(=C1O)C(=O)C(=CO2)C3=CC(=C(C=C3)O)O)OC4C(C(C(C(O4)CO)O)O)O | 3-(3,4-Dihydroxyphenyl)-5-hydroxy-6-methoxy-7-[3,4,5-trihydroxy-6-(hydroxymethyl)oxan-2-yl]oxychromen-4-one | **Flavonoid Glycoside** (Specifically, an Isoflavonoid Glycoside) | 0.092 | 0.328 | [31] compound 211 |

| | | | | | | | | | |
|---|---|---|---|---|---|---|---|---|---|
| 48 | 5011 | 461.1077 | 5.468 | COC1=C(C=C2C(=C1O)C(=O)C=C(O2)C3=CC(=C(C=C3)O)O)OC4C(C(C(C(O4)CO)O)O)O | 2-(3,4-Dihydroxyphenyl)-5-hydroxy-6-methoxy-7-{[3,4,5-trihydroxy-6-(hydroxymethyl)oxan-2-yl]oxy}chromen-4-one | **Flavonoid Glycoside** (Specifically, a Quercetin derivative) | 0.19 | 0.471 | [31] compound 214 |
| 49 | 2782 | 331.0812 | 4.345 | COC1=C(C=CC(=C1)C2=C(C(=O)C3=C(C=C(C=C3O2)O)O)OC)O | Quercetin 3,3'-dimethyl ether | **Flavonoid** (Methylated Flavonol) | 0.285 | 0.5 | [31] compound 216 |
| 50 | 14495 | 633.1598 | 8.84 | COC1=C(C=CC(=C1)C2C(OC3=C(O2)C=C(C=C3)C4C(C(=O)C5=C(C=C(C=C5O4)OC(=O)C6=CC(=C(C(=C6)O)OC)O)O)CO)O | | | 0.286 | 0.5 | |
| 51 | 12071 | 633.1597 | 7.978 | COC1=C(C2=C(C(=C1)O)C(=C3C(=C2)CC(OC3=O)CC(=O)OC)O)C4=C(C=C(C5=C4C=C(C(=O)C5=O)CC(CC(=O)OC)O)O)OC | Methyl 4-[8-[9,10-dihydroxy-7-methoxy-3-(2-methoxy-2-oxoethyl)-1-oxo-3,4-dihydrobenzo[g]isochromen-6- | **Polyketide/ Aromatic Lactone** | 0.098 | 0.485 | |

| | | | | | | | | | |
|---|---|---|---|---|---|---|---|---|---|
| | | | | | yl]-5-hydroxy-7-methoxy-3,4-dioxonaphthalen-2-yl]-3-hydroxybutanoate | (Not a terpenoid) | | | |
| 52 | 10267 | 315.0499 | 7.355 | COC1=C(OC2=C(C1=O)C(=C(C(=C2)O)O)O)C3=CC(=C(C=C3)O)O | Quercetagetin 3-methyl ether | **Flavonoid** (Methylated Flavonol) | 0.183 | 0.5 | [31] compound 216 |
| 53 | 9129 | 318.0972 | 6.969 | COC1=C2C(=C(C(=C1O)O)O)C(=O)C=C(O2)C3=CC=CC=C3 | 6-Hydroxywogonin | **Flavonoid** (Flavone derivative) | 0.17 | 0.333 | [31] compound 209 |
| 54 | 10723 | 433.151 | 7.56 | COC1=CC(=C(C2=C1C(=O)C=C(O2)C3=CC(=C(C(=C3OC)OC)OC)OC)OC)OC | 5,7,8-Trimethoxy-2-(2,3,4,5-tetramethoxyphenyl)chromen-4-one | **Polymethoxylated Flavonoid** (Highly methylated flavone) | 0.236 | 0.1 | [31] compound 210 |

| | | | | | | | | | |
|---|---|---|---|---|---|---|---|---|---|
| 55 | 8754 | 569.1078 | 6.842 | COC1=CC(=C2C(=C1)OC(=C(C2=O)C3=C(C4=C(C=C3O)OC(=CC4=O)C5=CC(=C(C=C5)O)O)O)C6=CC=C(C=C6)O)O | 3-[2-(3,4-Dihydroxyphenyl)-5,7-dihydroxy-4-oxochromen-6-yl]-5-hydroxy-2-(4-hydroxyphenyl)-7-methoxychromen-4-one | **Biflavonoid** (Dimers of flavonoids) | 0.131 | 0.493 | |
| 56 | 3591 | 287.0549 | 4.713 | COC1=CC(=C2C(=C1)OC3=C(C(=C(C=C3C2=O)OC)O)O)O | 1,5,6-Trihydroxy-3,7-dimethoxyxanthone | **Xanthone** (Polyphenolic compound, not a terpenoid) | 0.029 | 0.5 | |
| 57 | 3322 | 287.0549 | 4.669 | COC1=CC(=C2C(=C1)OC3=C(C2=O)C(=C(C(=C3)O)OC)O)O | 1,3,8-Trihydroxy-2,6-dimethoxy-9H-xanthen-9-one | **Xanthone** (Polyphenolic compound, not a terpenoid) | 0.04 | 0.5 | |
| 58 | 8712 | 341.0632 | 6.827 | COC1=CC(=C2C(=C1)OC3=CC(=C(C(=C3C2=O)O)OC)OC)O | 1,8-Dihydroxy-2,3,6-trimethoxyxanthen-9-one | **Xanthone** (Polyphenolic compound, | 0.092 | 1 | |

| | | | | | | | | | |
|---|---|---|---|---|---|---|---|---|---|
| | | | | | | not a terpenoid) | | | |
| 59 | 3549 | 523.1443 | 4.724 | COC1=CC(=CC(=C1O)OC)C(=O)OCC2C(C(C(C(O2)OC(=O)C3=CC(=C(C(=C3)OC)O)OC)O)O)O | [3,4,5-Trihydroxy-6-(4-hydroxy-3,5-dimethoxybenzoyl)oxyoxan-2-yl]methyl 4-hydroxy-3,5-dimethoxybenzoate | **Polyphenol Ester/Glycoside** (Not a terpenoid, sugar-ester of gallic acid derivatives) | 0.054 | 0.222 | [31] compound 275 |
| 60 | 4532 | 655.1654 | 5.262 | COC1=CC(=CC(=C1O)OC)C2=[O+]C3=CC(=CC(=C3C=C2OC4C(C(C(C(O4)COC(=O)C=CC5=CC(=C(C=C5)O)O)O)O)O)O)O | | | 0.166 | 0.359 | |
| 61 | 9644 | 841.3267 | 7.13 | COC1=CC(=CC(=C1O)OC)C2C3COC(C3CO2)C4=CC(=C(C(=C4)OC)OC(CO)C(C5=CC(=C(C(=C5)OC)O | 2-[4-[1,3-Dihydroxy-2-[4-[3-(4-hydroxy-3,5-dimethoxyphenyl)-1,3,3a,4,6,6a-hexahydrofuro[3,4-c]furan-6-yl]-2,6- | **Lignan/Complex Phenolic Polymer** | 0.033 | 0.498 | [31] compound 262 |

| | | | | | | | | | |
|---|---|---|---|---|---|---|---|---|---|
| | | | | C(CO)C(C6=CC(=C(C=C6)O)OC)O )OC)O)OC | dimethoxyphenoxy]propyl]-2,6-dimethoxyphenoxy]-1-(4-hydroxy-3-methoxyphenyl)propane-1,3-diol | (Not a terpenoid) | | | |
| 62 | 10476 | 841.3 275 | 7.427 | COC1=CC(=CC(=C1O)OC)C2C3C OC(C3CO2)C4=CC(=C(C(=C4)OC) OC(CO)C(C5=CC(=C(C(=C5)OC)O C(CO)C(C6=CC(=C(C=C6)O)OC)O )OC)O)OC | 2-[4-[1,3-Dihydroxy-2-[4-[3-(4-hydroxy-3,5-dimethoxyphenyl)-1,3,3a,4,6,6a-hexahydrofuro[3,4-c]furan-6-yl]-2,6-dimethoxyphenoxy]propyl]-2,6-dimethoxyphenoxy]-1-(4-hydroxy-3-methoxyphenyl)propane-1,3-diol | **Lignan/Complex Phenolic Polymer** (Not a terpenoid) | 0.034 | 0.499 | [31] compound 262 |
| 63 | 10380 | 479.1 342 | 7.374 | COC1=CC(=CC(=C1O)OC)C2CC(= O)OC3=C2C4=C(CC(C(O4)C5=CC( =C(C=C5)O)O)O)C(=C3)O | 2-(3,4-dihydroxyphenyl)-3,5-dihydroxy-10-(4-hydroxy-3,5-dimethoxyphenyl)-3,4,9,10-tetrahydro-2H-pyrano[2,3-f]chromen-8-one | **Flavonolignan/Complex Polyphenol** (Hybrid | 0.085 | 0.476 | |

| | | | | | | | | | |
|---|---|---|---|---|---|---|---|---|---|
| | | | | | | structure, not a terpenoid) | | | |
| 64 | 10099 | 375.1073 | 7.291 | COC1=CC(=CC(=C1OC(=O)C2=CC(=C(C(=C2)OC)OC)OC)OC)C(=O)O | 3,5-Dimethoxy-4-(3,4,5-trimethoxybenzoyl)oxybenzoic acid | **Polyphenol Ester** (Benzoic acid derivative) | 0.134 | 0.491 | [31] compound 275 |
| 65 | 10764 | 433.1128 | 7.591 | COC1=CC(=CC2=C1OCO2)C3=C(C(=O)C4=C(C(=C(C(=C4O3)OC)OC)OC)O)OC | 5-Hydroxy-3,6,7,8-tetramethoxy-2-(7-methoxy-1,3-benzodioxol-5-yl)chromen-4-one | **Polymethoxylated Flavonoid** (Flavone derivative) | 0.16 | 0.028 | |
| 66 | 10266 | 345.0968 | 7.351 | COC1=CC=C(C=C1)C2=CC(=O)C3=C(C(=C(C(=C3O2)OC)O)OC)O | Nevadensin | **Flavonoid** (Specifically, a Flavone derivative) | 0.364 | 0.5 | |

| | | | | | | | | | |
|---|---|---|---|---|---|---|---|---|---|
| 67 | 2817 | 613.166 | 4.364 | COC1=CC=C(C=C1)C2=CC(=O)C3=C(C(=C(C=C3O2)OC4C(C(C(C(O4)C(=O)O)(CC5CNCN5)O)O)O)OCO)O | 3,4,5-Trihydroxy-6-[5-hydroxy-6-(hydroxymethoxy)-2-(4-methoxyphenyl)-4-oxochromen-7-yl]oxy-3-(imidazolidin-4-ylmethyl)oxane-2-carboxylic acid | **Flavonoid Glycoside/Hybrid** (Complex glycosylated flavonoid with a rare imidazolidine moiety, not a terpenoid) | 0.079 | 0.012 | |

### S1.3. Structure validation of target protein

Ramachandran plot is a graphical representation that visualizes energetically favorable positions of a protein backbone based on torsion angles phi ($\phi$) and psi ($\Psi$) [21]. The Ramachandran plot is categorized into four regions: disallowed, generously allowed, additional allowed, and most favored regions. A protein structure is deemed high-quality when more than 90% of its residues are located within the most favored region [22]. The Ramachandran plots analysis of PDB ID: 4ASE showed that 91.7% of the residues were in allowed regions, indicating a stable and high-quality structure. The Ramachandran plot analysis of PDB ID: 4ASE is presented in Fig. S2.

Amino acids and atomic level binding interactions were assessed using ERRAT, which evaluates the frequency of noncovalent interactions among carbon (C), nitrogen (N), and oxygen (O) atoms. The analysis considers six types of noncovalent interactions: CC, CN, CO, NN, NO, and OO. High-resolution protein structures are defined by an ERRAT quality factor of $>$ 95% [23]. The ERRAT value of PDB ID: 4ASE was 98.32%.

### S1.4. Molecular Docking

The Gibbs free energy (kcal/mol), reflect stability of the ligand and receptor interaction. The more negative the value means stronger binding [24]. The inhibition constant (KI) shows the drug effectiveness in inhibiting receptor activity. The lower the values meaning stronger inhibition [25]. Hydrogen bonds play a key role in stabilizing ligand binding in proteins by precisely aligning atoms, which enhances binding affinity and improves docking scores [26]. Van der Waals interaction strength, which is influenced by the distance between atom [27], also plays a crucial role in the formation of protein-ligand complexes [28].

Additionally, carbon-hydrogen bond interactions further supports the stability of these complexes [29, 30].

## S2. Supplemental Methods

### S2.1. Isolation of Total DNA, PCR amplification and Sequencing

The quality and concentration of the extracted genomic DNA were evaluated using a NanoDrop One UV-Vis spectrophotometer (Thermo Scientific, Massachusetts, USA). Its integrity was verified through agarose gel electrophoresis. The amplification reactions were performed using a T960 PCR thermal cycler (Drawell, China) in a final reaction volume of 25 µL. Each PCR reaction mixture consisted of approximately 100 ng of genomic DNA, 12.5 µL of KOD One™ PCR Master Mix (TOYOBO Life Science, Japan), 0.75 µL of forward and reverse primers (10 pmol/µL each), and PCR-grade water to reach the final volume. The PCR thermal cycling conditions involved an initial denaturation step at 98 °C for 10 seconds, followed by 40 cycles of denaturation at 98 °C for 10 seconds, annealing at 52 °C (*mat*K and *rbc*L) or 55 °C (ITS and *trn*H-*psb*A) for 5 seconds, and extension at 68 °C for 3 seconds. This protocol ensured precise amplification of target regions, facilitating accurate genetic identification of the *L. japonicus* samples. The PCR products were subjected to analysis using 1.5% agarose gel electrophoresis. The gel was stained with RedSafe nucleic acid staining solution (iNtRON Biotechnology, South Korea) to ensure the visualization of DNA bands under UV light, which was performed using a Gel Doc™ EZ Imager (Bio-Rad, USA). Successfully amplified PCR products were subsequently sequenced in both forward and reverse directions using an ABI PRISM 3730XL sequencer (Applied Biosystems, USA), ensuring high accuracy in nucleotide sequence determination. The resulting nucleotide sequences were meticulously reviewed and manually corrected using BioEdit software version

7.2.6 [1] to address potential sequencing errors. The corrected sequences were then aligned using MUSCLE [2], integrated within the Molecular Evolutionary Genetics Analysis (MEGA) software version 11 [3] to establish accurate alignments for downstream analyses. To verify the genetic identity of the samples, the sequences were compared to reference sequences available in the NCBI nucleotide database through the Basic Local Alignment Search Tool (BLAST) [4].

### S2.2. LC-MS/MS Data pre-processing parameters

Chromatograms were generated using ADAP chromatogram builder with the following parameters: a minimum consecutive scans, 7; minimum intensity for consecutive scans, 1000; minimum absolute height, 1000; *m/z* tolerance = 0.005 or 20 ppm. Feature detection using the local minimum feature resolver was performed with the following settings: MS1 to MS2 tolerance of 0.010 *m/z* or 10 ppm; minimum relative feature height, 25%; minimum absolute height, 1000; minimum scans, 3. The $^{13}C$ isotope filter was applied using an *m/z* tolerance, 0.0050 or 2.5 ppm; retention time tolerance, 0.15. For the Join aligner and feature finder (multithreaded) parameters were set to a *m/z* tolerance 0.0030 or 5 ppm; retention time tolerance, 0.15.

### S2.3. Isolation method

The LJ-M fraction was separated using normal-phase flash column chromatography (NP-FCC) with a stepwise gradient of *n*-hexane, ethyl acetate, acetone, and methanol, yielding eight fractions (1-8). Fraction 2 (2.38 g) was further purified by normal-phase silica gel column chromatography (NP-SGC) using gradient elution of *n*-hexane-acetone, resulting in nine subfractions (2.1-2.9). Subfraction 2.4 (742 mg) was subjected to NP-SGC, producing seven fractions (2.4.1 to 2.4.7). Further purification on subfraction 2.4.2 (292 mg) yielded six subfractions (2.4.2.1 to 2.4.2.6) by NP-SGC. From subfraction 2.4.2.2 (23 mg), compound **3**

(1.2 mg) and compound **2** (2.7 mg) were isolated using NP-SGC with *n*-hexane-acetone solvent system. Compound **2** (12 mg) was also obtained from subfraction 2.3 (86 mg) using NP-SGC with *n*-hexane-ethyl acetate gradients.

Subfraction 2.4.2.3 (103 mg) was purified by NP-SGC with *n*-hexane-acetone elution, yielding four fractions (2.4.2.3.1 to 2.4.2.3.4). Fraction 2.4.2.3.2 (88 mg) was further purified using HPLC-DAD with a chiral column with an isocratic solvent system of *n*-hexane-isopropanol (20:80), and compound **1** (5 mg) was isolated.

Subfraction 2.5 (709 mg) was subjected to NP-SGC, with gradient elution of *n*-hexane-acetone, producing seven subfractions (2.5.1 to 2.5.7). Subsequent purification of subfraction 2.5.3 led to isolation of compound **4** (7 mg). Subfraction 2.5.4 (194 mg) was further purified using reverse-phase silica gel column chromatography (RP-SGC) with a water-methanol gradient, yielding seven subfractions (2.5.4.1 to 2.5.4.7). Subfraction 2.5.4.4 was further purified and compound **6** (6.5 mg) was isolated. Fraction 4 (3.5 g) was purified by NP-SGC with *n*-hexane-acetone elution, yielding nine subfractions (4.1 to 4.9). Subfraction 4.5 (1.1 g) was further purified, producing ten subfractions (4.5.1 to 4.5.10). Subfraction 4.5.10 (185 mg) was purified using RP-SGC with a water-methanol gradient, and compound **5** (5.6 mg) was obtained.

### S2.4. Anti-angiogenesis activity

The sulforhodamine B (SRB) assay was conducted as previously described [5], to evaluate the effects of LJ partition fractions (LJ-H, LJ-M, LJ-B, and LJ-W) on the cell viability of human CD34-positive endothelial progenitor cells (EPCs). Briefly, The EPCs were isolated and cultured according to established protocols described in previous studies [6]. The EPCs

were maintained in MV2 (Endothelial Cell Growth Medium MV2) culture medium according to an established protocol [7, 8]. EPCs were seeded in 96-well plates at a density of 5 × $10^3$ cells per well and incubated overnight. The following day, the culture medium was replaced with MV2 medium containing the test samples and maintained for 48 hours. EPCs growth was evaluated using a previously reported method [8, 9]. For the capillary tube formation assay, EPCs were plated at the density of 1.25×$10^4$ cells per well on Matrigel-coated 96-well plates. Cells were then incubated in MV2 complete medium suspended with 2% fetal bovine serum (FBS), and the test compounds were applied at a specified concentration for 24 hours at 37 °C. Cell differentiation and the formation of capillary-tube-like structures were evaluated through microscopic imaging. Photomicrographs were captured using an inverted phase-contrast microscope to document morphological changes. The long axis of each structure was measured in three randomly chosen fields per well using MacBiophotonics ImageJ software [10].

### S2.5. Molecular docking and dynamics

#### S2.5.1. Tools and materials

The study utilized Chem3D (version 17.1) to prepare test compounds and standard drugs, while Molegro Molecular Viewer (MWV, version 2.5) was used to separate native ligands and proteins. The AutoDockTools (version 1.5.7) was utilized for molecular docking, while Maestro (version 12.7, 2021) by Schrodinger was employed for molecular dynamics simulation. BIOVIA Discovery Studio Visualizer (version 21.1.0.20298) and PyMOL were used to visualize molecular docking results and native ligand-protein structure preparation, respectively. Additionally, online tools such as Protein Data Bank (PDB), SAVES for protein structure validation, PubChem, PDBsum for structural analysis, pkCSM for small-molecule pharmacokinetics prediction, and Lipinski's Rule of Five to evaluate drug-likeness of a

compound, were utilized. The hardware included a PC equipped with an Intel® Core™ i7-10700 CPU @ 2.90 GHz (8 cores), NVIDIA GeForce RTX 3050 (4 GB), 24 GB DDR4 RAM, running Windows 10 Enterprise (64-bit), and a PC with identical specifications operated on Linux Ubuntu 18.04.5 LTS.

**S2.5.2. Protein/ receptor preparation**

The crystal structure of VEGFR-2 (PDB ID: 4ASE, resolution: 1.83 Å) was downloaded in .pdb format from the Protein Data Bank (RCSB) website (https://www.rcsb.org). The receptor was evaluated using the Ramachandran Plot, with disallowed region values below 0.8%, analyzed on PDBsum (http://www.ebi.ac.uk/pdbsum/) using the PDB receptor code. The Target receptor, with an ERRAT quality score above 95%, was selected and analyzed on the SAVES server (https://saves.mbi.ucla.edu/) [11-13]. The retrieved high-resolution receptor was prepared by removing water molecules, conformers, and native ligands using the Molegro Molecular Viewer. Charges and hydrogens were added to the native ligand and receptor using BIOVIA Discovery Studio 2021, and the structure was saved in .pdb format [14].

**S2.5.3. Ligand preparation**

The native ligand, tivozanib, retrieved from PDB ID: 4ASE, resolution: 1.83 Å (the crystal structure of VEGFR-2), was used to validate receptor docking. The 2D structures of isolated compounds were prepared in .sdf format using Chemdraw (version 17.1). All ligands underwent energy minimization with the Molecular Mechanics 2 (MM2) force field in Chem3D, which calculates steric and thermal energies. The lowest energy conformers were saved in .mol2 format [15, 16]. The MM2 force field demonstrated strong performance in the conformational analysis of organic molecules [17].

### S2.5.4. Molecular docking and interaction analysis

The prepared ligands and receptors were validated by redocking the native ligand (tivozanib) into the receptor's active site using AutoDockTools. A grid box with dimensions of 40 x 40 x 40 Å was defined. The box aligned along the x, y, and z axes to cover the receptor's active site. The docking protocol was considered acceptable when the RMSD value ≤ 2 Å. The binding site coordinates were -22.541, 0.537, and -10.478 for x, y, and z, respectively. Docking was performed for all isolated compounds against the target protein using the Lamarckian GA algorithm with 300 runs, following the same protocol as for the native ligand (tivozanib). The result provided the inhibition constant (KI) in molarity (M) and free binding energy (ΔG) in kcal/mol [18]. The ligand-receptor conformations with the lowest binding energies were determined and saved in .pdb format. Discovery Studio 2021 and Pymol were employed to visualize and analyze the 2D and 3D interactions between the ligands and receptors [19, 20].

### S2.5.5. Molecular dynamic simulation

MD simulations of the Protein-ligand complex were conducted using Maestro software. The protein-ligand complex, with water molecules and ions (needed for stability), was placed inside an orthorhombic box with buffer sizes of 10 Å along each axis. The stability of the compounds with the lowest binding energy (from molecular docking) was assessed through MD simulations. The protein preparation wizard in Maestro software was used to pre-process complexes, including adding hydrogens and assigning bond orders. MD simulations were conducted using the transferable intermolecular potential 3-point (TIP3P) water model, which accelerates kinetics, preserves the structure of thermodynamically stable conformers, lowers energy barriers between states, and improves sampling during simulations. The TIP3P model also mimics physiological ionic concentration (0.15 M NaCl).

**Table S1. $^1$H and $^{13}$C NMR data of compounds 2 and 3 (600 and 150 MHz; *δ* in ppm, *J* in Hz), compound 4 (400 and 100 MHz; *δ* in ppm, *J* in Hz) in $CDCl_3$.**

| No | 2 | | 3 | | 4 | |
|---|---|---|---|---|---|---|
| | $\delta_H$ (multi.; *J* in Hz.) | $\delta_C$, type | $\delta_H$ (multi.; *J* in Hz.) | $\delta_C$, type | $\delta_H$ (multi.; *J* in Hz.) | $\delta_C$, type |
| 1 | 1.42, td (12.9, 3.7)<br>1.97, m | 36.1 $CH_2$ | 1.39, m<br>1.69, td (9.54, 4.64) | 35.5 $CH_2$ | 1.43, m * | 32.3 $CH_2$ |
| 2 | 1.60, m<br>1.69, dt (13.6, 3.4) | 18.8 $CH_2$ | 2.61, m<br>2.50, m | 19.4 $CH_2$ | 1.09, m<br>1.67, m | 18.0 $CH_2$ |
| 3 | 1.25, m<br>1.50, dtd (13.3, 3.3, 1.5) | 41.5 $CH_2$ | 1.31, m<br>1.47, m | 41.0 $CH_2$ | 1.15, m<br>1.43, m * | 41.3 $CH_2$ |
| 4 | | 33.3 C | | 34.1 C | | 34.5 C |
| 5 | 1.73, m | 50.5 CH | 2.50, m | 42.8 CH | 1.76, dd (14.0, 2.5) | 50.0 CH |
| 6 | 2.51, m *<br>2.38, dd (17.5, 14.4) | 35.4 $CH_2$ | 2.11, dd (17.6, 5.2)<br>2.39, m | 40.8 $CH_2$ | 2.35, dd (11.6, 2.5)<br>2.57, m | 36.2 $CH_2$ |
| 7 | | 200.4 C | | 210.7 C | | 207.5 C |
| 8 | | 130.5 C | 2.39, m | 36.0 $CH_2$ | | 88.6 C |
| 9 | | 167.2 C | | 215.6 C | | 82.2 C |
| 10 | | 41.1 C | | 52.5 C | | 44.8 C |
| 11 | 2.45, m * | 30.4 $CH_2$ | 2.85, ddd (17.6, 9.1, 5.7)<br>2.69, ddd (17.6, 9.1, 5.9) | 37.8 $CH_2$ | 2.12, m | 30.8 $CH_2$ |
| 12 | 2.56, m * | 24.4 $CH_2$ | 2.50, m<br>2.61, m | 19.9 $CH_2$ | 2.52, m | 21.4 $CH_2$ |
| 13 | | 124.7 C | | 124.5 C | | 124.9 C |
| 14 | 6.31, dd (1.8, 0.9) | 110.7 CH | 6.28, dd (1.8, 0.8) | 111.3 CH | 6.30, dd (1.9, 0.9) | 111.0 CH |
| 15 | 7.38, t (1.7) | 143.2 CH | 7.32, t (1.7) | 142.8 CH | 7.38, t (1.7) | 143.3 CH |
| 16 | 7.27, m | 138.8 CH | 7.22, m | 139.2 CH | 7.26, m * | 138.9 CH |
| 17 | 1.80, s | 11.6 $CH_3$ | 1.02, t (7.3, $CH_3$) | 8.1 $CH_3$ | 1.49, s | 15.3 $CH_3$ |
| 18 | 0.90, s | 32.7 $CH_3$ | 0.80, s | 33.0 $CH_3$ | 0.90, s | 33.3 $CH_3$ |

| | | | | | | |
|---|---|---|---|---|---|---|
| 19 | 0.93, s | 21.5 $CH_3$ | 0.88, s | 23.0 $CH_3$ | 0.86, s | 21.7 $CH_3$ |
| 20 | 1.10, s | 18.3 $CH_3$ | 1.10, s | 17.5 $CH_3$ | 1.20, s | 16.8 $CH_3$ |
| 21-C OAc | | | | | | 169.3 C |
| 22-CH3 OAc | | | | | 2.09, s | 21.6 $CH_3$ |

* - Overlapped signals

**Table S2. $^1$H and $^{13}$C NMR data of compound 5 (400 and 100 MHz; $\delta$ in ppm, $J$ in Hz) in $C_5D_5N$ and compound 6 (600 and 150 MHz; $\delta$ in ppm, $J$ in Hz) in $CDCl_3$.**

| No | 5 | | 6 | |
|---|---|---|---|---|
| | $\delta_H$ (multi.; $J$ in Hz.) | $\delta_C$, type | $\delta_H$ (multi.; $J$ in Hz.) | $\delta_C$, type |
| 1 | | | | |
| 2 | | 164.9 C | | 163.9 C |
| 3 | 6.94, s | 104.3 CH | 6.57, s | 103.9 CH |
| 4 | | 183.2 C | | 183.1 C |
| 5 | | 163.1 C | | 148.5 C |
| 6 | 6.77, d (2.0) | 100.4 CH | | 130.9 C |
| 7 | | 166.3 C | | 148.5 C |
| 8 | 6.84, d (2.1) | 95.3 CH | | 127.6 C |
| 9 | | 158.9 C | | 146.0 C |
| 10 | | 105.4 C | | 104.6 C |
| 1ˊ | | 122.7 C | | 123.8 C |
| 2ˊ | 7.95, d (8.7) | 129.3 CH | 7.89, d (8.4) | 128.2 CH |
| 3ˊ | 7.24, d (11.1) | 117.3 CH | 7.04, d (8.7) | 114.8 CH |
| 4ˊ | | 163.6 C | | 162.8 C |
| 5ˊ | 7.24, d (11.1) | 117.3 CH | 7.04, d (8.7) | 114.8 CH |
| 6ˊ | 7.95, d (8.7) | 129.3 CH | 7.89, d (8.4) | 128.2 CH |
| 5-OH | 13.8, s (OH) | | 12.78, s (OH) | |
| 6-$OCH_3$ | | | 4.04, s | 62.0 $CH_3$ |
| 7-$OCH_3$ | | | | |
| 8-$OCH_3$ | | | 4.01, s | 61.2 $CH_3$ |
| 3'-$OCH_3$ | | | | |
| 4'-$OCH_3$ | | | 3.90, s | 55.7 $CH_3$ |

**Tables S3 are shown below for anti-angiogenesis activity**

**Table S3.1 Cytotoxicity effect of crude fractions on EPCs that may indicate anti-angiogenesis.**

| **Cell survival EPCs** | | |
|---|---|---|
| **Treatment-48h** | **CTL** | **50 µg/mL** |
| LJ-H | 100±0% | **74±1%** |
| LJ-M | 100±0% | **23±2%** |
| LJ-B | 100±0% | 96±2% |
| LJ-W | 100±0% | 96±3% |

Results represented as mean ± S.E.M (n = 2).

**Table S3.2 Cytotoxicity effect of isolated compounds on EPCs that may indicate anti-angiogenesis.**

| **EPCs survival (%)** | | | |
|---|---|---|---|
| **Compound** | **CTL** | **30 µM** | **100 µM** |
| **1** | 100 ± 0% | 88 ± 4% | 21 ± 2% |
| **2** | 100 ± 0% | 91 ± 5% | 43 ± 0% |
| **3** | 100 ± 0% | 90 ± 1% | 57 ± 2% |
| **4** | 100 ± 0% | 79 ± 4% | 14 ± 3% |
| **5** | 100 ± 0% | 43 ± 1% | <0% |
| **6** | 100 ± 0% | 100 ± 3% | 76 ± 5% |

Results represented as mean ± S.E.M (n = 3). The treatment was performed for 48 hours. Cytotoxicity effect of compounds on Human endothelial progenitor cells (EPCs).

**Table S4. Effect of isolated compounds on tube formation and LDH release in EPCs after a 48-hour treatment period.**

| | Tube length (% of control) |
|---|---|
| **Compounds** | **50 µM** |
| Compound **1** | 79.17 ± 3.22* |
| Compound **4** | 68.23 ± 7.12* |
| Compound **5** | 53.63 ± 10.86* |
| | **10 µM** |
| Sorafenib (standard) | 46.50 ± 10.98* |

All samples were nontoxic to EPCs using LDH assay.
Data represent the mean ± S.E.M. of at least three independent experiments. *$p < 0.05$ compared with the control group, in human endothelial progenitor cells (EPCs).

**Table S5. Receptor resolution.**

| **Receptor type** | **PDB** | **Resolution (Å)** | **Receptor** | **Ligand** |
|---|---|---|---|---|
| VEGFR-2 | 4ASE | 1.83 Å | 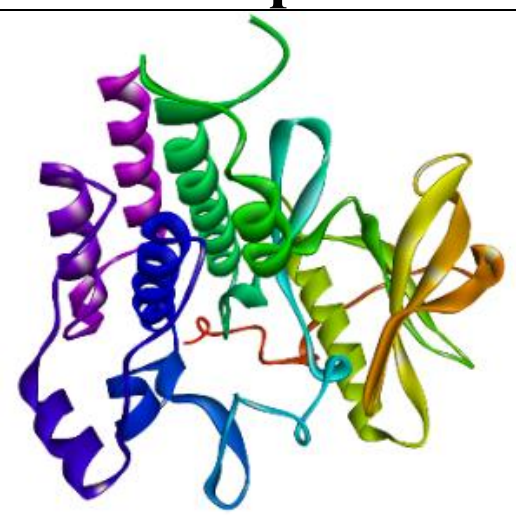 | 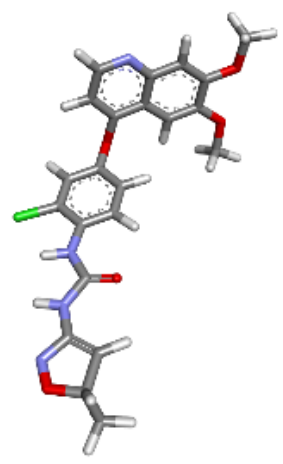 |

**Table S6. Molecular docking validation results.**

| Native Ligand | PDB | Grid Box Dimension X | Grid Box Dimension Y | Grid Box Dimension Z | Grid Center X | Grid Center Y | Grid Center Z | Spacing (Å) | RMSD (Å) | Binding Affinity (kcal/mol) |
|---|---|---|---|---|---|---|---|---|---|---|
| AV9 | 4ASE | -22.54 | 0.53 | -10.47 | 40 | 40 | 40 | 0.37 | 1.42 | -12.13 |

**Table S7. Molecular docking results of isolated compounds (1-6), standard drugs, and a native ligand with PDB ID: 4ASE.**

| **PDB ID** | **Compound** | **Binding affinity (kcal/mol)** | **Inhibition constant** | **Conventional hydrogen bonds/ distance (Å)** | **Van Der Waals** | **Carbon hydrogen bond** |
|---|---|---|---|---|---|---|
| 4ASE | **1a** | -9.24 | 169.74 nM | Asp A:1046 (2x) (3.99, 4.29), | Glu A:917, Phe A:918, | Glu A:885 |

| | | | | | | |
|---|---|---|---|---|---|---|
| | | | | Cys A:919 (3.73), | Phe A:1047, Val A:848 | |
| 4ASE | **1b** | -9.58 | 94.66 nM | Cys A:919 (3.64), Lys A:868 (3.76) | Asp A:1046, Glu A: 885, Leu A:840, Phe A:918, Val A:867, Val A:914 | Glu A:917 |
| 4ASE | **2** | -9.13 | 201.50 nM | Cys A:919 (3.64), Lys A:868 (3.76) | Asp A:1046, Glu A:885, Leu A:840, Phe A:918, Val A:867, Val A:914 | Glu A:917 |
| 4ASE | **3** | -9.35 | 140.11 nM | Asp A:1046 (4.09), Cys A:919 (3.50) | Glu A:885 | Glu A:917 |
| 4ASE | **4** | -7.20 | 5.27 µM | AspA:1046 (2.84) | Asp A:814, Glu A:885, His A:1024, Ile A:1044, Ile A:892, Leu A:889, Leu A:1019, Phe A:1047, Val A:898 | - |
| 4ASE | **5** | -7.94 | 1.52 µM | Asp A:1046 (3.08), Cys A:919 (3.09), Glu A:885 (5.92) Lys A:868 (3.90) | Glu A:917, Gly A:922, Leu A:840, Leu A:889, Phe A:1047, Phe A:918, Val A:867, Val A:914 | - |
| 4ASE | **6** | -8.60 | 493.68 nM | Lys A:868 (4.07), | Asp A:1046, | Glu A:885 Lys A:920 |

| | | | | | | |
|---|---|---|---|---|---|---|
| | | | | Cys A:919 (2x) (3.32, 2.49) | Glu A:917, Gly A:841, Gly A:922, Val A:916 | |

| PDB ID | Standard drug | Binding affinity (kcal/mol) | Inhibition constant | Conventional hydrogen bonds/ distance (Å) | Van Der Waals | Carbon hydrogen bond/ distance (Å) |
|---|---|---|---|---|---|---|
| 4ASE | Axitinib | -11.52 | 3.59 nM | Cys A:919 (3.78), GluA:917 (4.36), Glu A:885 (4.98) | Asp A:1046 Gly A:922, Phe A:918, Phe A:1047 | Lys A:920 |
| 4ASE | Cabozantinib | -11.29 | 5.3 nM | Asp A:1046 (3.85), Lys A:868 (5.20), Cyc A:919 (3.01) | Gly A:841, Gly A:885, Ile A:1044, Leu A:1019, Val A:898, Val A:914 | Glu A:917, Gly A:922, His A:1026, Lys A:920 |
| 4ASE | Lenvatinib | -11.60 | 3.14 nM | Asp A:1046 (4.21), Glu A:885 (2x) (3.92, 5.31) Leu A:840 (5.26), Cys A:919 (3.44) | Gly A:841, Phe A:1047, Val A:899 | Gly A:922, Glu A:917, Lys A:920 |
| 4ASE | Sunitinib | -9.52 | 104.28 nM | Asp A:1046 2x (3.91, 4.35), Glu A:885 (5.33) | Glu A:917, Gly A:922, His A:1026, Ile A:888, Ile A:892, Ile A:1044, Leu A:1019, Phe A:918 | - |
| 4ASE | Sorafenib | -11.92 | 1.82 nM | Asp A:1046 (4.27), | Gly A:922, Leu A:840 Lys A: 920 Phe A:918 | Glu A:917, His A:1026, |

| | | | | | | |
|---|---|---|---|---|---|---|
| | | | | Glu A:885 (4.16 and 5.38)<br>Cys A:919 (4.24, 3.61 and 3.39) | Val A:899 | |

| **PDB ID** | **Native ligand** | **Binding affinity (kcal/mol)** | **Inhibition constant** | **Conventional hydrogen bonds/ distance (Å)** | **Van Der Waals** | **Carbon hydrogen bond** |
|---|---|---|---|---|---|---|
| 4ASE | Tivozanib | -12.13 | 1.29 nM | GluA:885 (2x) (4.04, 5.42), AspA:1046 (4.39), CysA:919 (3.53) | GlyA:922, LysA:920, GlyA:841, IleA:888, ValA:899 | GluA:917 (5.52), |

**Table S8. Amount and percentage of residue in favored, allowed, and disallowed regions of isolated compounds (1-6)-4ASE complex and sunitinib-4ASE complex.**

| **Compound** | **Number and percentage of residues in favored region** | **Number and percentage of residues in allowed region** | **Number and percentage of residues in disallowed region** |
|---|---|---|---|
| (**A**) **1a-**4ASE | 218 (82.0 %) | 48 (18.0 %) | 0 (0 %) |
| (**B**) **1b-**4ASE | 213 (80.0 %) | 53 (20.0 %) | 0 (0 %) |
| (**C**) **2-**4ASE | 223 (83.8 %) | 43 (16.2 %) | 0 (0 %) |
| (**D**) **3-**4ASE | 229 (86.1 %) | 37 (13.9 %) | 0 (0 %) |
| (**E**) **4-**4ASE | 216 (81.2 %) | 50 (18.8 %) | 0 (0 %) |
| (**F**) **5-**4ASE | 222 (83.5 %) | 44 (16.5 %) | 0 (0 %) |
| (**G**) **6** -4ASE | 224 (84.2 %) | 41 (15.5 %) | 1 (0.4 %) |
| **Standard drug** | | | |
| (**H**) Sunitinib-4ASE | 224 (84.2 %) | 42 (15.8 %) | 0 (0 %) |
| **(I)** Sorafenib-4ASE | 232 (87.2 %) | 31 (11.7 %) | 0 (0 %) |

This table presents the amount and percentage of amino acid residues located in the favored, allowed, and disallowed regions of the Ramachandran plot for VEGFR2 (PDB ID: 4ASE) complexes with isolated compounds (**1**-**6**) and the reference drug sunitinib. Data are provided for each distinct complex: (**A**) compound **1a**, (**B**) compound **1b**, (**C**) compound **2**, (**D**) compound **3**, (**E**) compound **4**, (**F**) compound **5**, (**G**) compound **6**, (**H**) sunitinib, and (**I**) sorafenib.

**Table S9. Average, minimum, and maximum RMSD of isolated compounds (1-6) and sunitinib complexes with VEGFR2 (PDB ID: 4ASE).**

| Complex | Average RMSD | Minimum RMSD | Maximum RMSD |
|---|---|---|---|
| (**A**) **1a-**4ASE | 1.227 | 0.768 | 2.000 |
| (**B**) **1b-**4ASE | 1.397 | 0.248 | 2.252 |
| (**C**) **2-**4ASE | 0.285 | 0.128 | 0.905 |
| (**D**) **3-**4ASE | 1.029 | 0.351 | 1.480 |
| (**E**) **4-**4ASE | 1.394 | 1.038 | 1.619 |
| (**F**) **5-**4ASE | 0.518 | 0.102 | 0.857 |
| (**G**) **6** -4ASE | 0.842 | 0.251 | 1.177 |
| **Standard drug** | | | |
| (**H**) Sunitinib-4ASE | 1.221 | 0.410 | 1.557 |
| **(I)** Sorafenib-4ASE | 0.954 | 0.276 | 1.263 |

This table presents the minimum, and maximum Root Mean Square Deviation (RMSD) values for VEGFR2 (PDB ID: 4ASE) complexes with isolated compounds (**1**-**6**) and the reference drug sunitinib. Data are provided for each distinct complex: (**A**) compound **1a**, (**B**) compound **1b**, (**C**) compound **2**, (**D**) compound **3**, (**E**) compound **4**, (**F**) compound **5**, (**G**) compound **6**, (**H**) sunitinib, and (**I**) sorafenib.

**Table S10. Average, minimum, and maximum RMSF of isolated compounds (1-6) and sunitinib complexes with VEGFR2 (PDB ID: 4ASE).**

| Complex | Average RMSF | Minimum RMSF | Maximum RMSF |
|---|---|---|---|
| (**A**) **1a-**4ASE | 1.392 | 0.468 | 5.963 |
| (**B**) **1b-**4ASE | 1.128 | 0.401 | 6.011 |
| (**C**) **2-**4ASE | 1.114 | 0.421 | 5.441 |
| (**D**) **3-**4ASE | 1.141 | 0.411 | 7.906 |
| (**E**) **4-**4ASE | 1.040 | 0.382 | 4.328 |
| (**F**) **5-**4ASE | 1.137 | 0.457 | 5.467 |
| (**G**) **6 -**4ASE | 1.221 | 0.398 | 5.815 |
| **Standard drug** | | | |
| (**H**) Sunitinib-4ASE | 1.179 | 0.382 | 5.985 |
| **(I)** Sorafenib-4ASE | 0.958 | 0.390 | 5.805 |

This table presents the average, minimum, and maximum Root Mean Square Fluctuation (RMSF) values for VEGFR2 (PDB ID: 4ASE) complexes with isolated compounds (**1**-**6**) and the reference drug sunitinib. Data are provided for each distinct complex: (**A**) compound **1a**, (**B**) compound **1b**, (**C**) compound **2**, (**D**) compound **3**, (**E**) compound **4**, (**F**) compound **5**, (**G**) compound **6**, (**H**) sunitinib, and (**I**) sorafenib.

**Table S11. Lipinski results of** isolated **compounds (1-6).**

| Compound | Molecular mass | Hydrogen bond donor | Hydrogen bond acceptor | Lipophilicity (LogP) | Molar refractivity | Complying with rule |
|---|---|---|---|---|---|---|
| **1a** | 376.0 | 0 | 5 | 4.524 | 102.116 | 5 |
| **1b** | 376.0 | 0 | 5 | 4.524 | 102.116 | 5 |
| **2** | 300.0 | 0 | 2 | 5.334 | 88.581 | 5 |
| **3** | 318.0 | 0 | 3 | 4.983 | 91.179 | 5 |
| **4** | 376.0 | 1 | 5 | 4.070 | 101.002 | 5 |
| **5** | 268.0 | 3 | 4 | 2.625 | 73.889 | 5 |
| **6** | 342.0 | 2 | 6 | 2.945 | 91.881 | 5 |

**Table S12. ADMET parameters of** isolated **compounds (1-6).**

| Property | Parameter (human) | Compound-**1a** | Compound-**1b** | Compound-**2** | Compound-**3** | Compound-**4** | Compound-**5** | Compound-**6** |
|---|---|---|---|---|---|---|---|---|
| Absorption | Water solubility (log mol/L) | -4.97 | -4.97 | -6002 | -5.594 | -4.292 | -3.407 | -3.54 |
| | Caco2 permeability (log Papp in 10 cm/s) | 0.895 | 0.895 | 1.682 | 1.5 | 0.875 | 1.09 | 0.285 |
| | Intestinal absorption (% Absorbed) | 97.5 | 97.5 | 98.962 | 97.742 | 97.144 | 91.639 | 80.052 |
| | Skin Permeability (log Kp) | -2.983 | -2.983 | -2.399 | -2.61 | -3.878 | -2.803 | -2.81 |
| | P-glycoprotein substrate (Yes/No) | No | No | No | No | No | Yes | Yes |
| | P-glycoprotein I inhibitor (Yes/No) | Yes | Yes | Yes | Yes | Yes | No | No |

| | | | | | | | | |
|---|---|---|---|---|---|---|---|---|
| | P-glycoprotein II inhibitor (Yes/No) | Yes | Yes | No | No | Yes | No | Yes |
| Distribution | VDss (log L/kg) | 0.155 | 0.155 | 0.644 | 0.438 | 0.086 | -0.075 | 0.071 |
| | Fraction unbound (Fu) | 0.043 | 0.043 | 0 | 0.007 | 0.1 | 0.221 | 0.191 |
| | BBB permeability (log BB) | -0.47 | -0.47 | 0.196 | -0.206 | -0.083 | -0.862 | -0.742 |
| | CNS permeability (log PS) | -2.171 | -2.171 | -2.104 | -2.255 | -2.78 | -2.126 | -3.12 |
| Metabolism | CYP2D6 substrate (Yes/No) | No | No | No | No | No | No | No |
| | CYP3A4 substrate (Yes/No) | Yes | Yes | Yes | Yes | No | No | No |
| | CYP1A2 inhibitor (Yes/No) | No | No | No | No | No | Yes | Yes |
| | CYP2C19 inhibitor (Yes/No) | No | No | Yes | Yes | Yes | Yes | Yes |
| | CYP2C9 inhibitor (Yes/No) | No | No | No | No | Yes | Yes | Yes |
| | CYP2D6 inhibitor (Yes/No) | No | No | No | No | No | No | No |
| | CYP3A4 inhibitor (Yes/No) | No | No | No | No | Yes | No | No |
| Excretion | Total Clearance (log ml/min/kg) | 0.976 | 0.976 | 1.055 | 1.314 | 0.737 | 0.575 | 0.638 |
| | Renal OCT2 substrate (Yes/No) | No | No | No | No | No | No | No |
| Toxicity | AMES toxicity (Yes/No) | No | No | No | No | No | No | No |
| | Max. tolerated dose (log mg/kg/day) | 0.597 | 0.597 | 0.084 | 0.339 | -0.358 | 0.615 | 0.587 |
| | hERG I inhibitor (Yes/No) | No | No | No | No | No | No | No |
| | hERG II inhibitor (Yes/No) | No | No | No | No | No | No | No |
| | Oral Rat Acute Toxicity (LD50) (mol/kg) | 2.212 | 2.212 | 2.087 | 2.255 | 2.968 | 2.045 | 2.356 |
| | Oral Rat Chronic Toxicity (LOAEL) (log mg/kg_bw/day) | 1.106 | 1.106 | 1.003 | 2.285 | 1.614 | 1.702 | 1.254 |
| | Hepatotoxicity (Yes/No) | No | No | Yes | No | No | No | No |

| | | | | | | | | |
|---|---|---|---|---|---|---|---|---|
| | Skin Sensitization (Yes/No) | No | No | No | No | No | No | No |
| | *T.Pyriformis* toxicity (log ug/L) | 1.616 | 1.616 | 2.442 | 2.657 | 0.707 | 0.553 | 0.386 |
| | Minnow toxicity (log mM) | -0.959 | -0.959 | -1.56 | -0.982 | -0.699 | 1.714 | 0.6 |

**Figure S1. Extraction and partition scheme.**

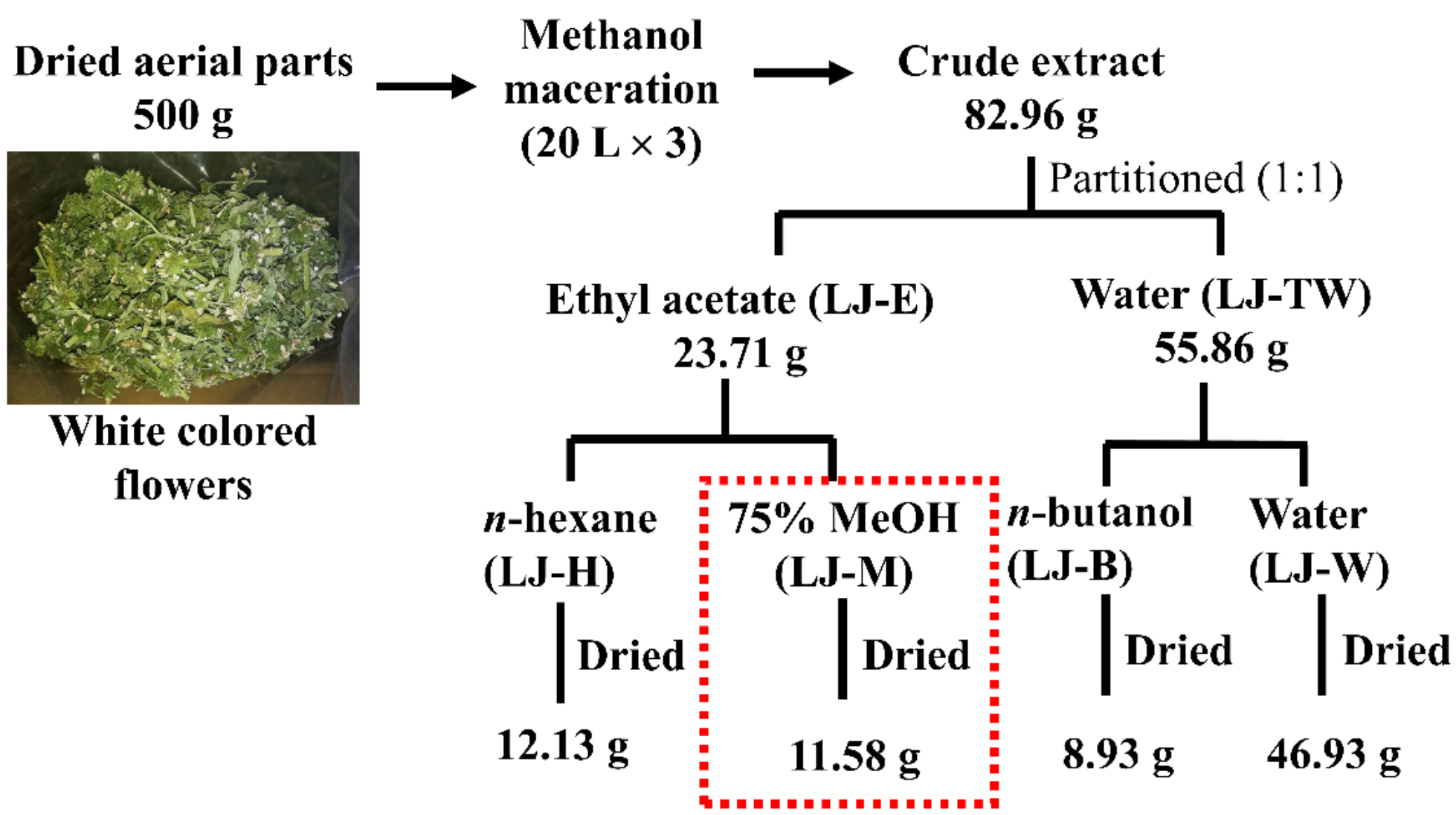


**Figure S1.1. HPLC isolation and chromatogram of chiral compound 1**

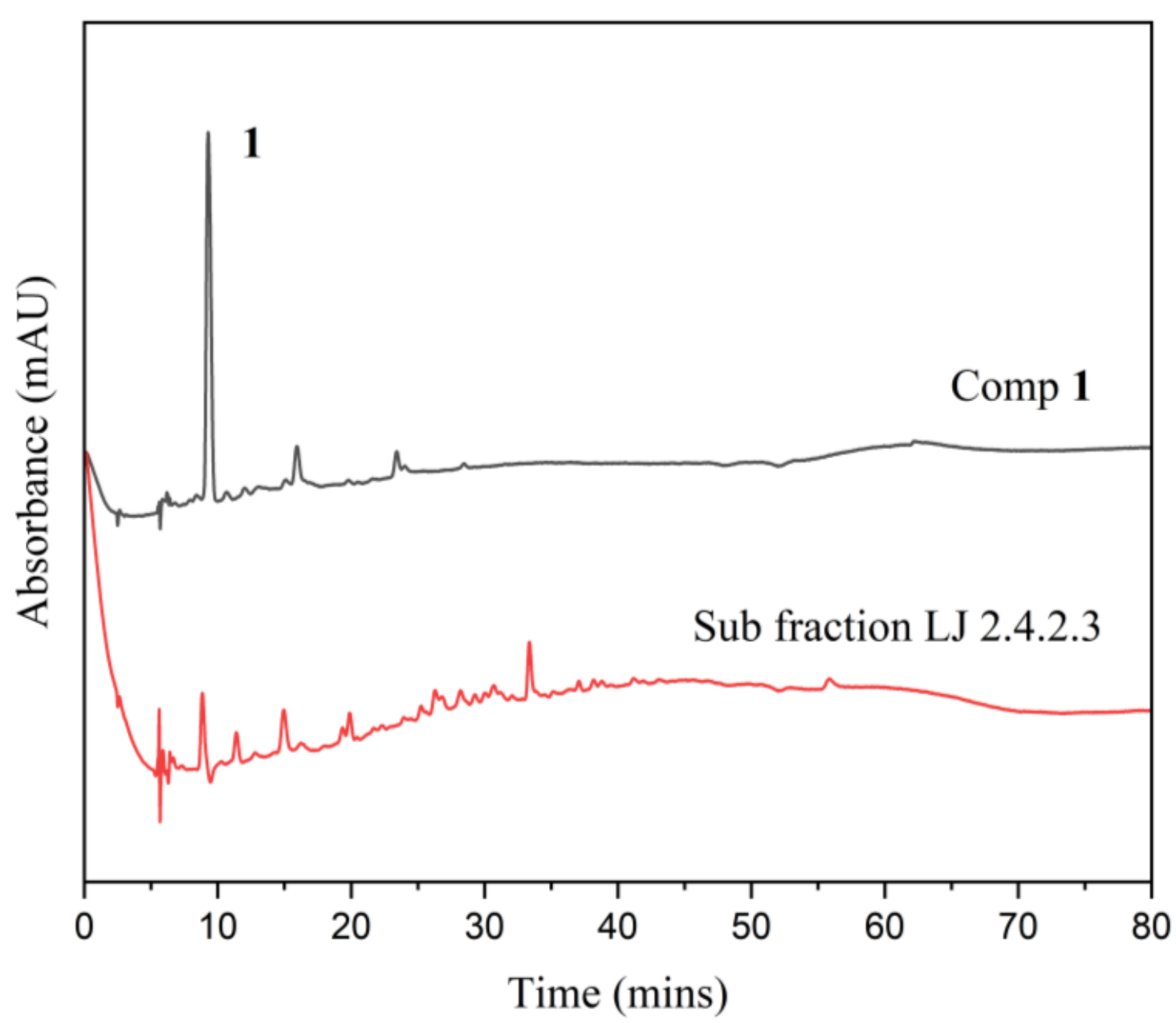

**Figure S1.2. LJ-M HPLC profiling chromatogram at 210 nm wavelength**

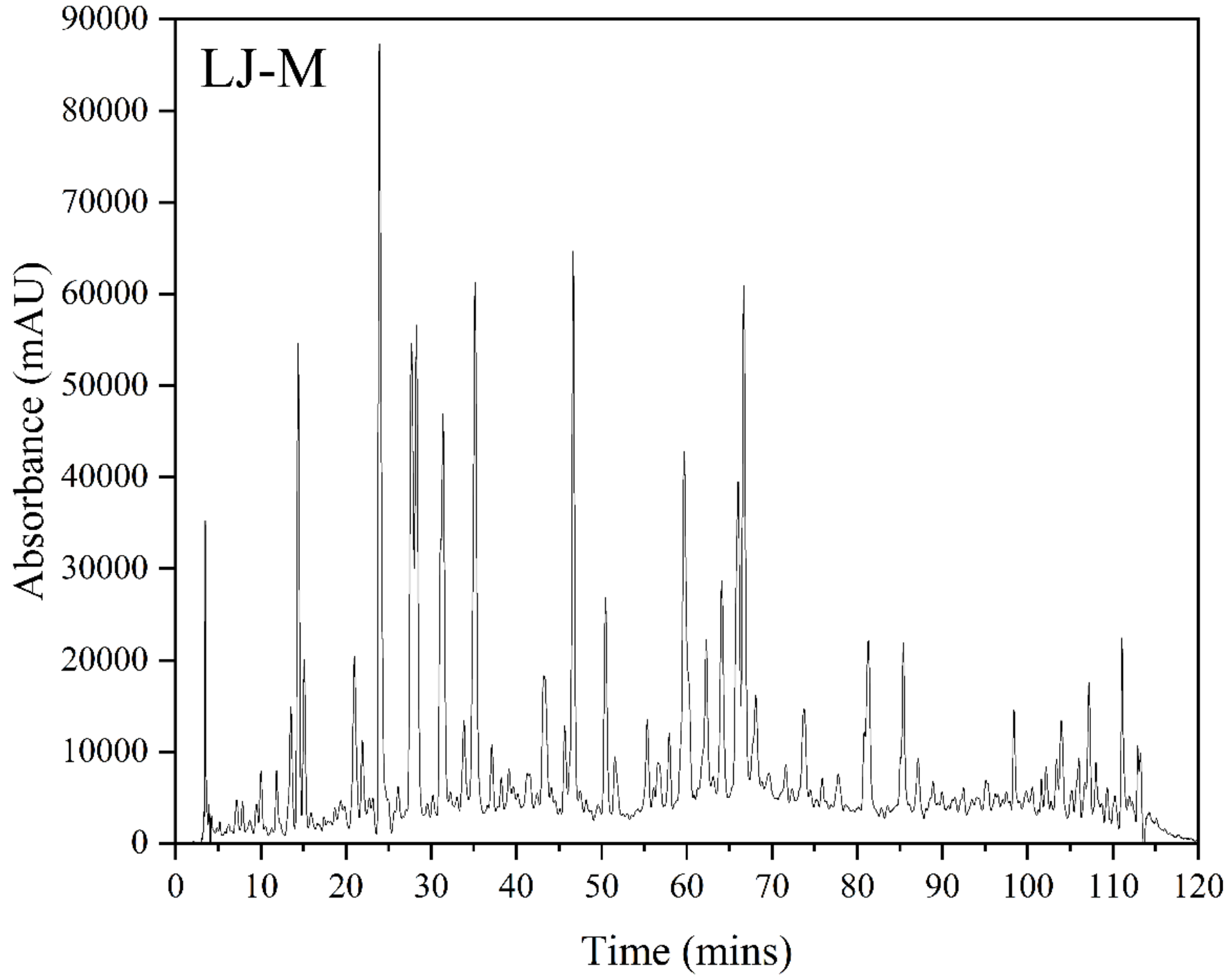


**Figure S1.3. HPLC profiling and purity of isolated compounds**

| Compound | Retention time (RT) | Wavelength (nm) | Purity percent (%) |
|---|---|---|---|
| **1** | 85.577 | 230 | 95.0 |
| **2** | 90.778 | 254 | 85.0 |
| **3*** | 62.377 | 200 | 89.0 |
| **4** | 79.309 | 230 | 94.0 |
| **5** | 50.491 | 254 | 93.0 |
| **6** | 64.179 | 254 | 98.3 |

* = Cannot quantify accurately due to limited amount of sample and low UV absorption.

**Figure S1.3.1. HPLC profile of compound 1**

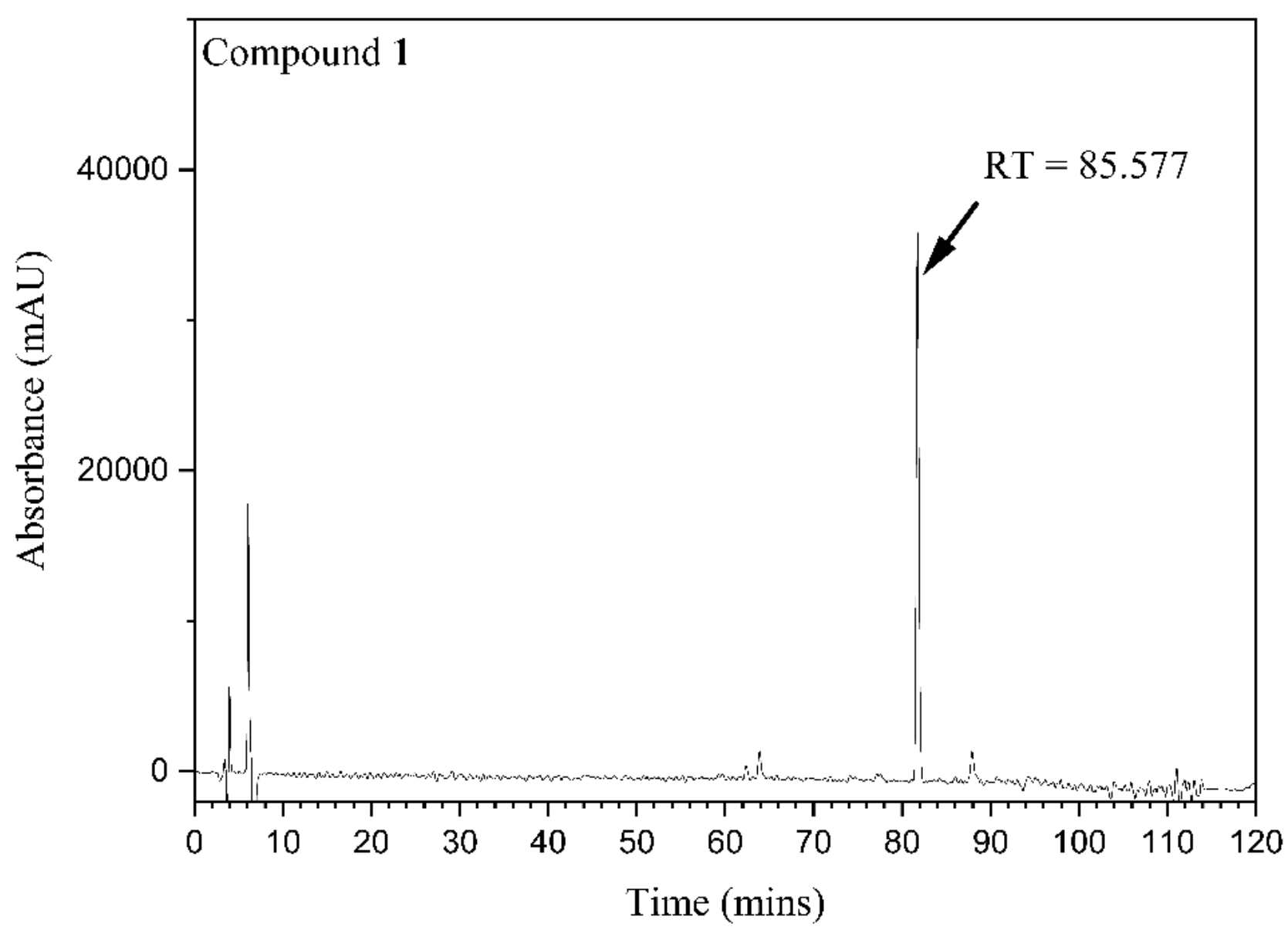


**Figure S1.3.2. HPLC profile compound 2**

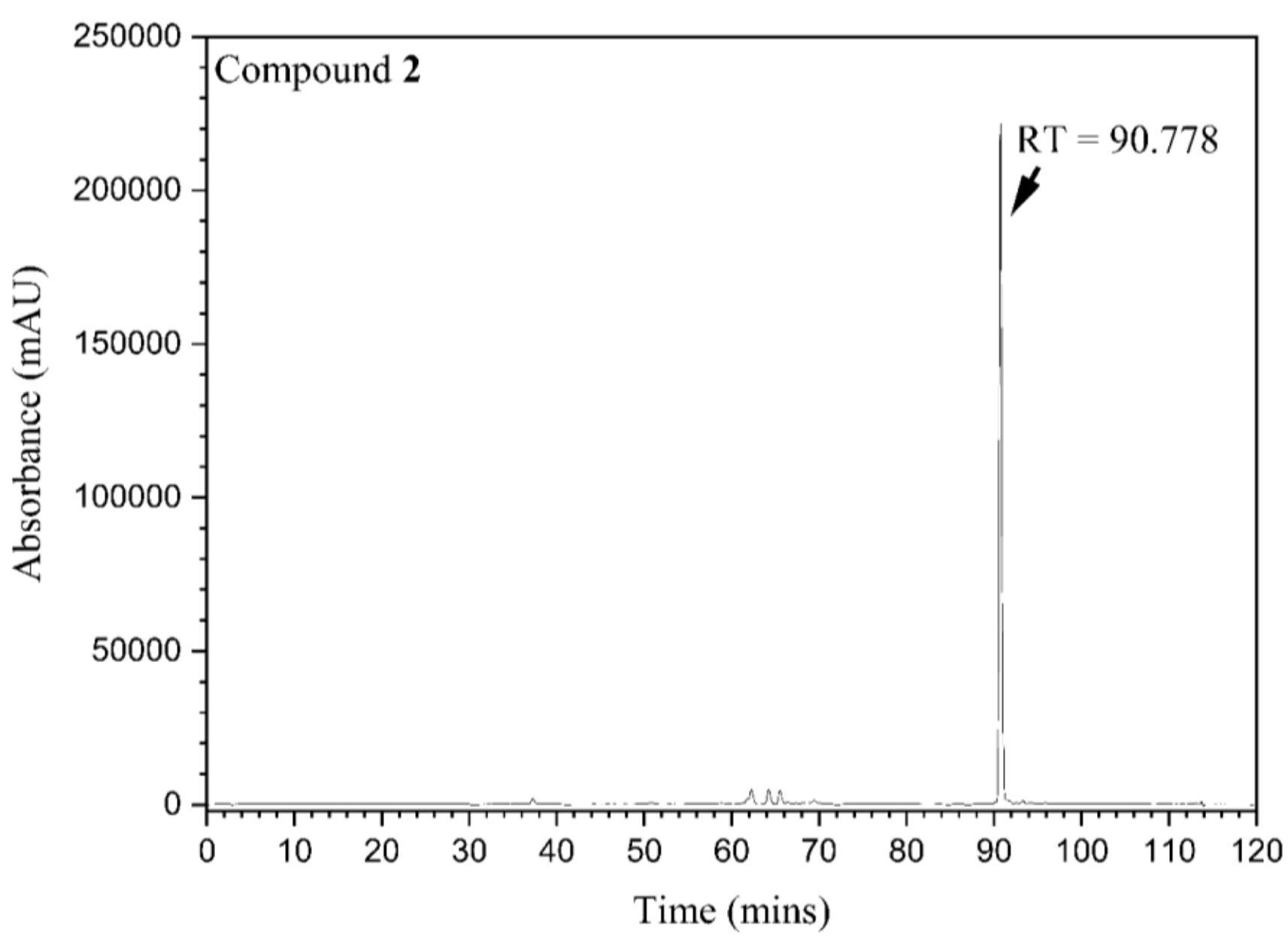

**Figure S1.3.3. HPLC profile compound 3**

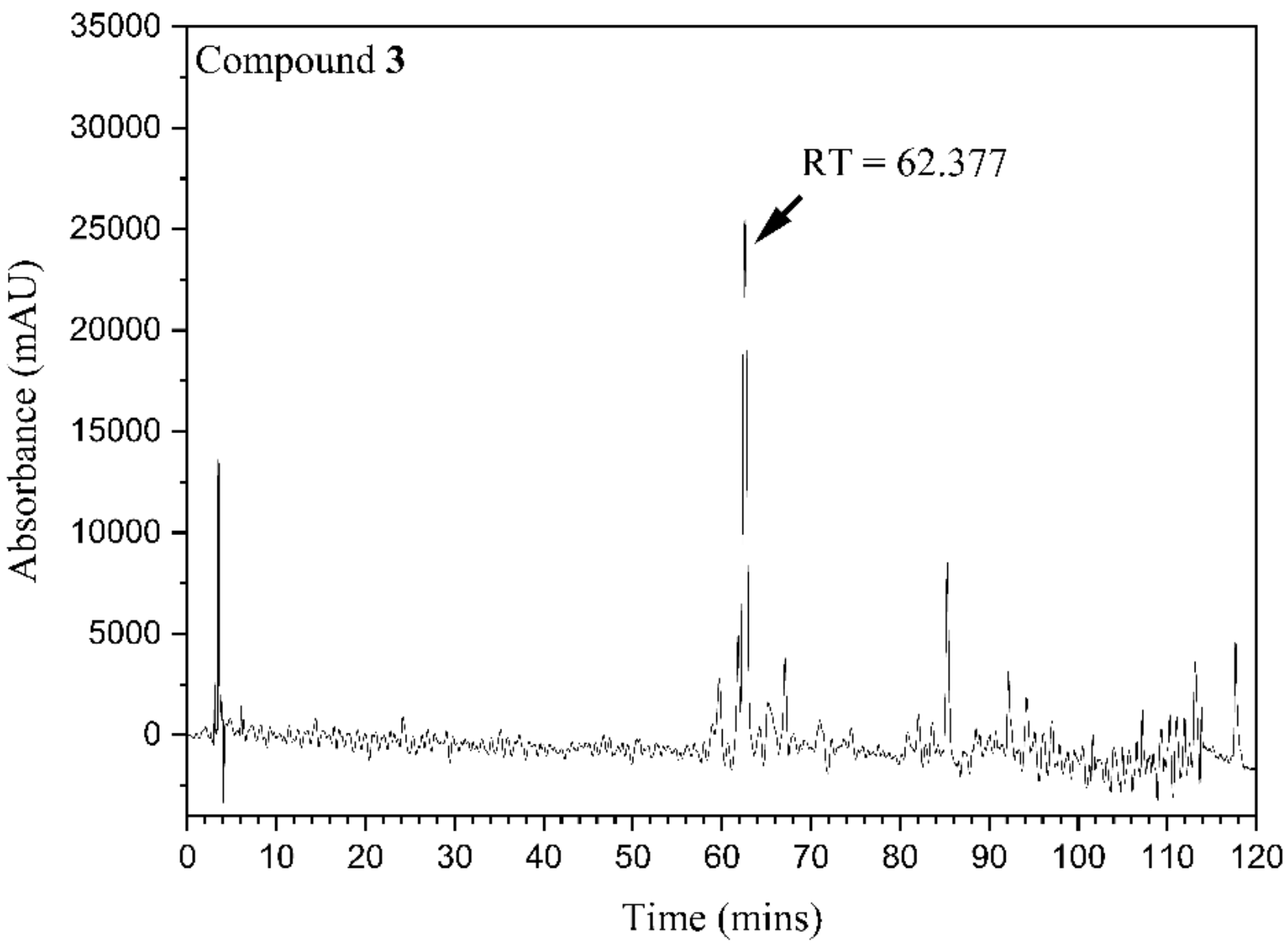


**Figure S1.3.4. HPLC profile compound 4**

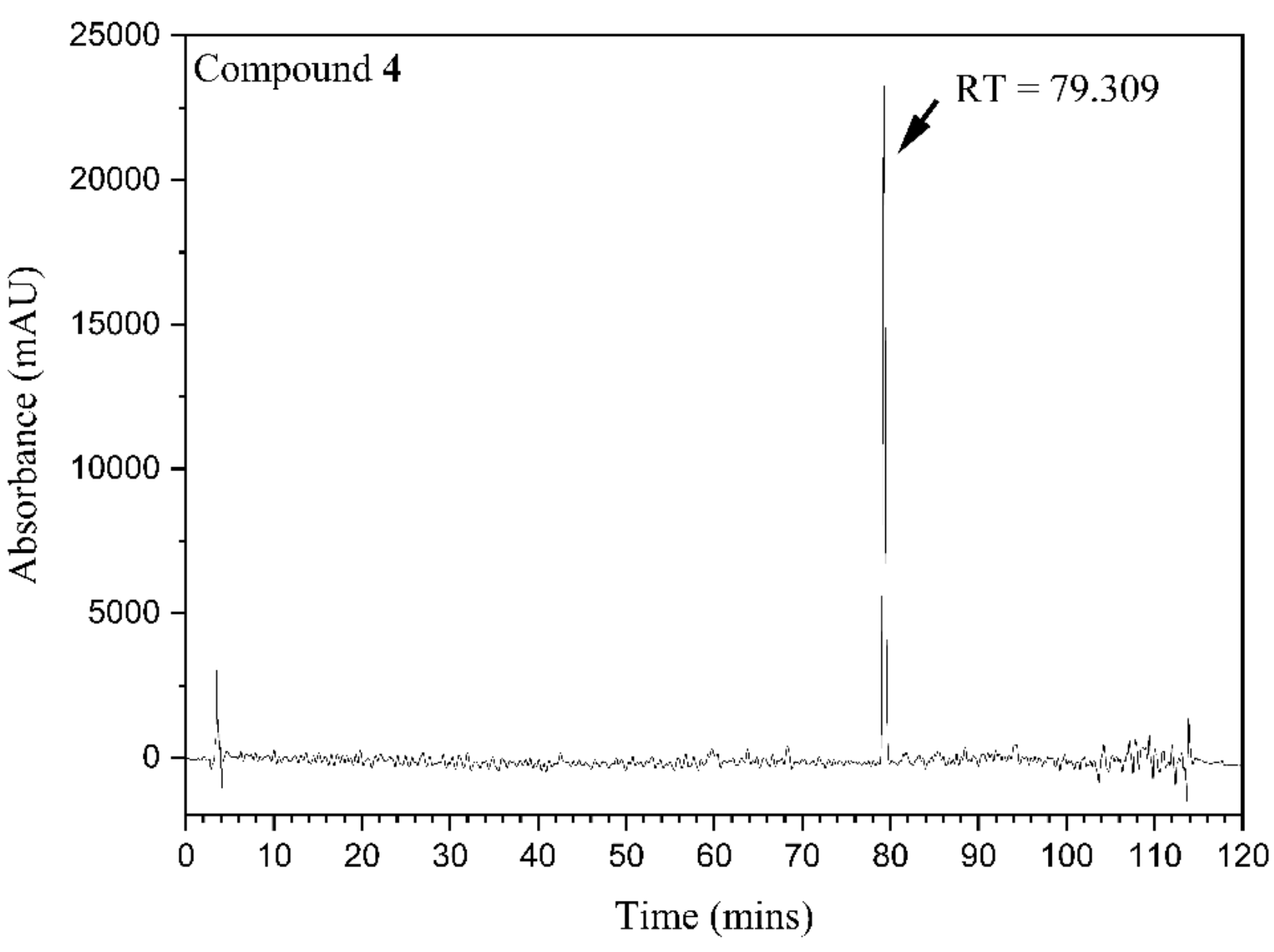

**Figure S1.3.5. HPLC profile compound 5**

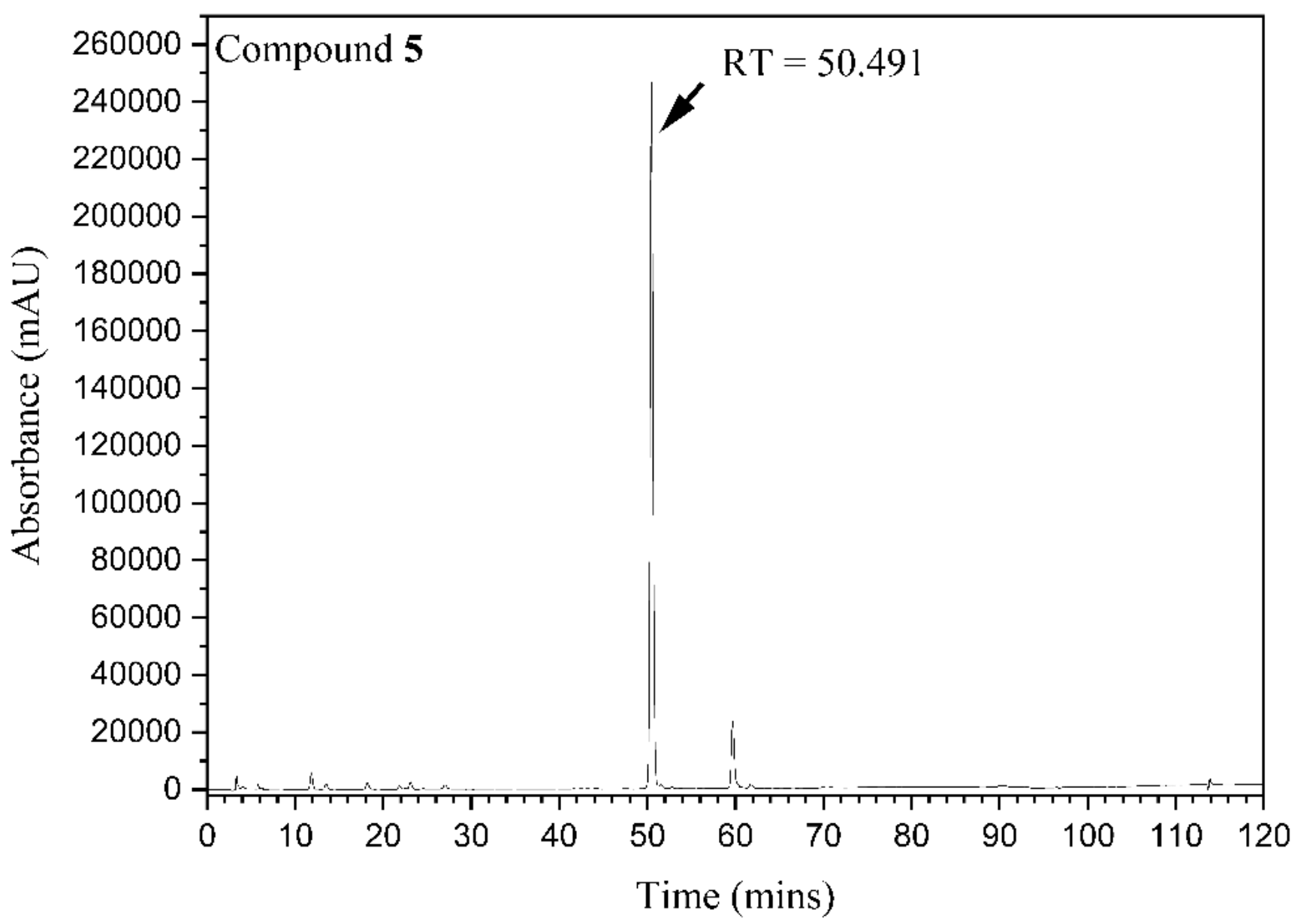


**Figure S1.3.6. HPLC profile compound 6**

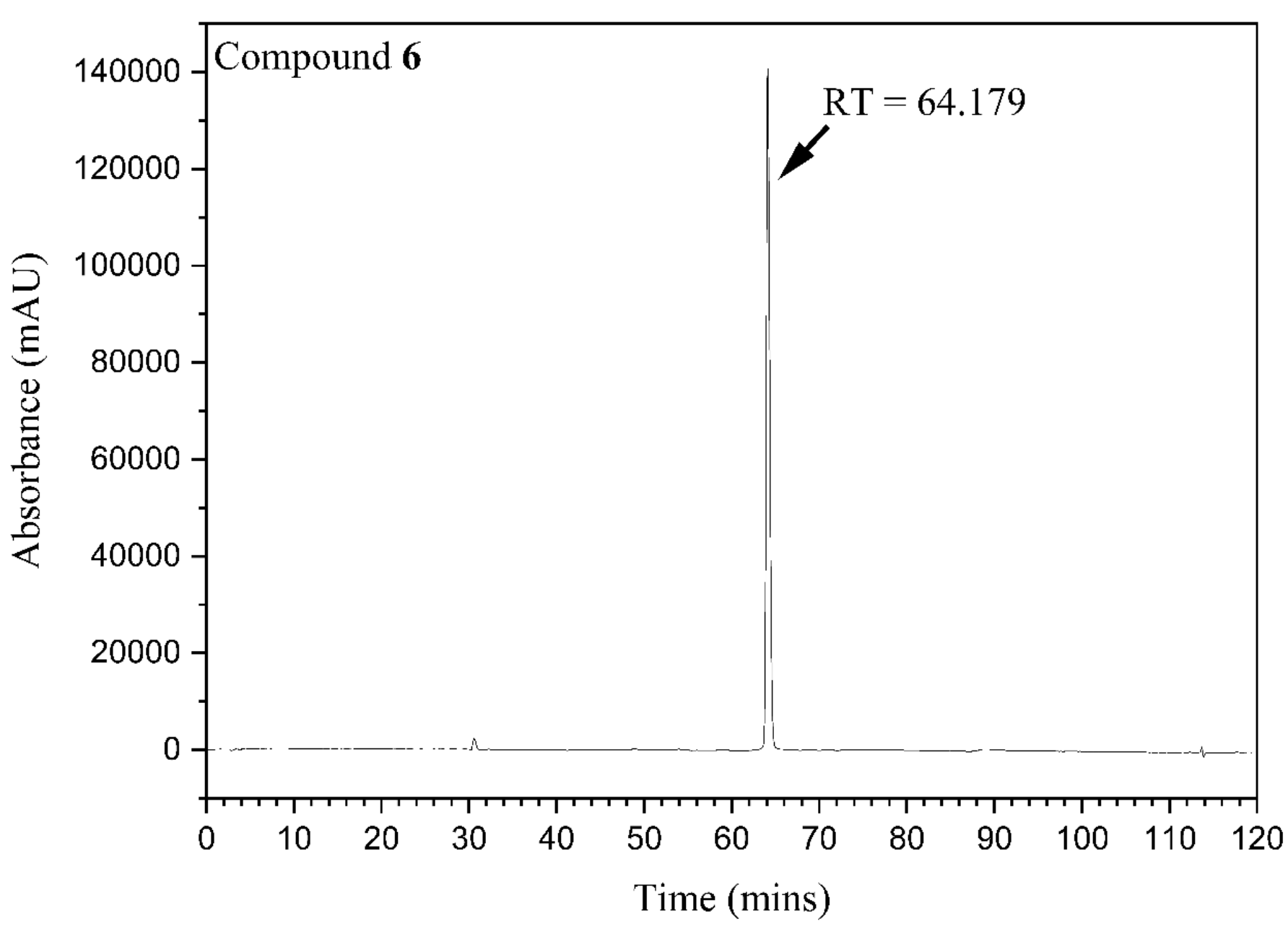

**Figure S2. The Ramachandran plot of PDB ID: 4ASE.**

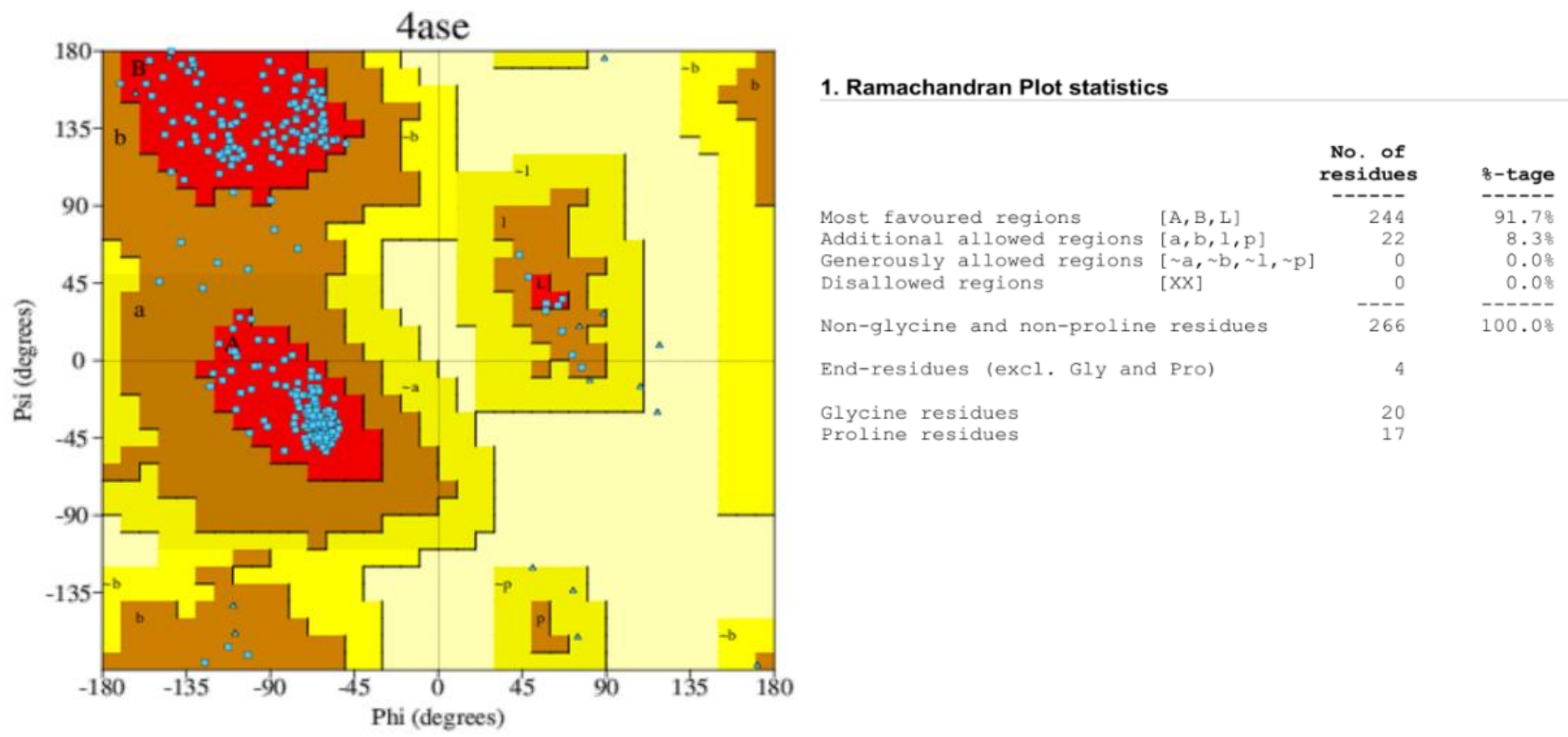


**Figure S3. ERRAT result value of PDB ID: 4ASE.**

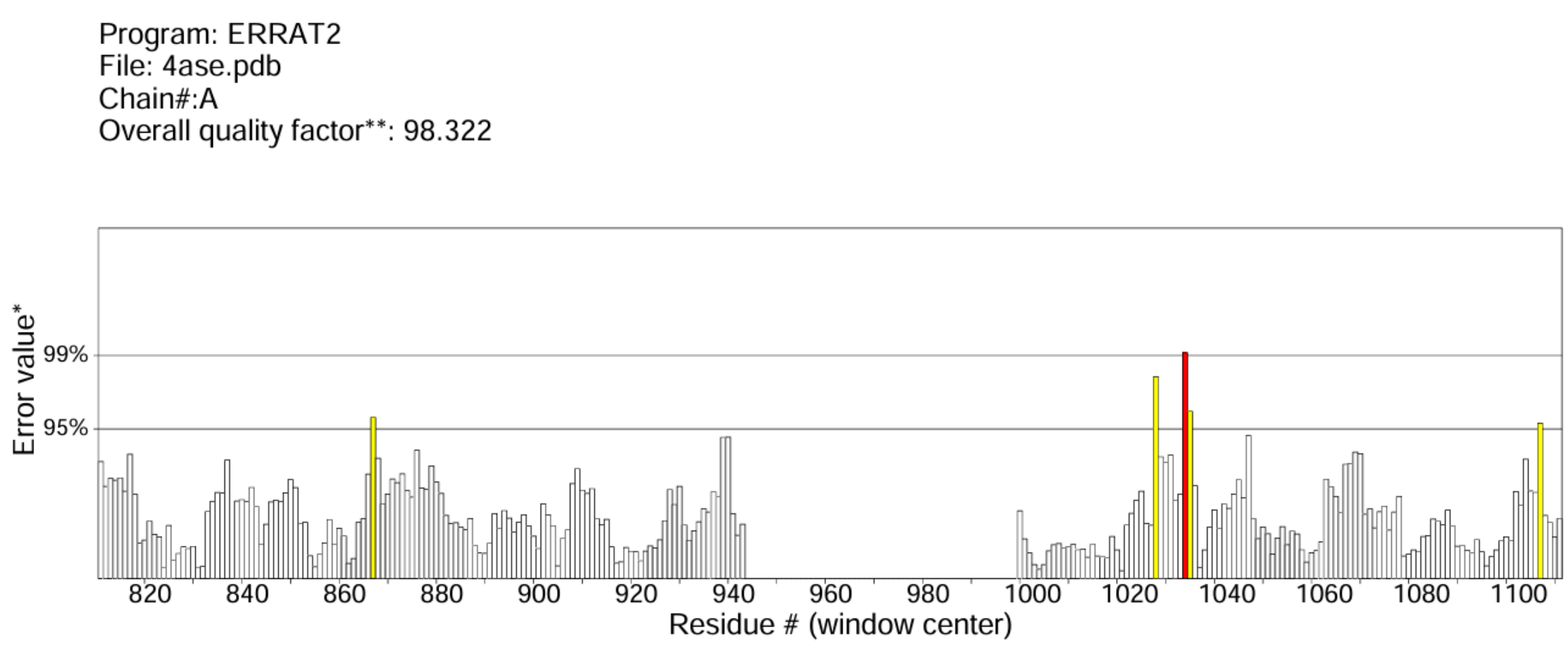

**Figure S4. Stack view of the native ligand crystallographic structure and its re-docking structure.**

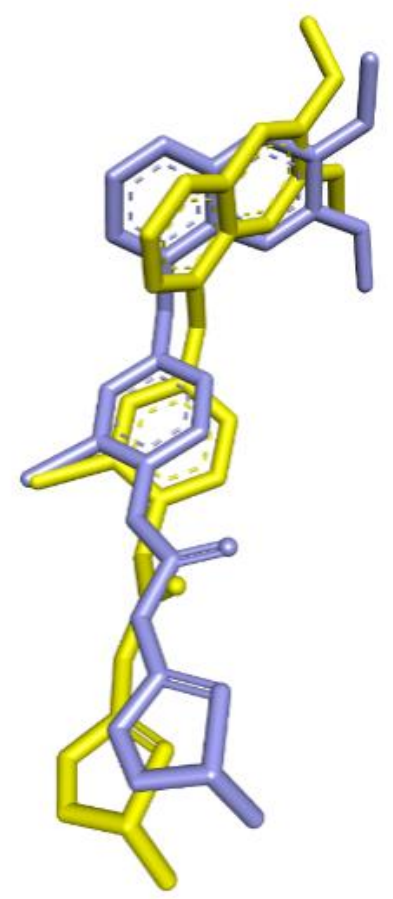

The yellow color represents native ligand conformation before docking. The violet color represents native ligand conformation after docking.

**Figure S5. Showing key difference between compound 1a (generated) and 1b (isolated).**

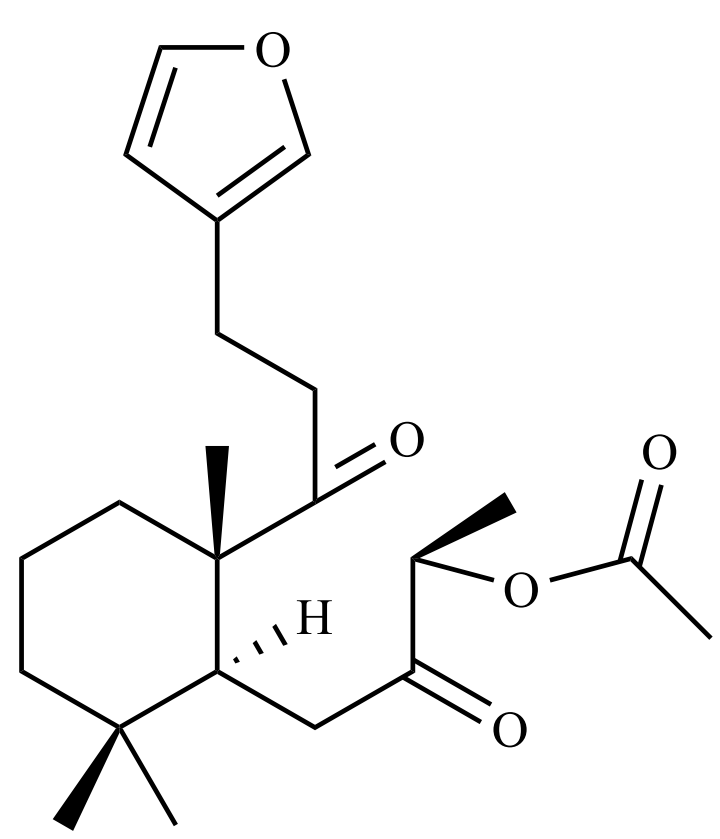

Compound **1a**

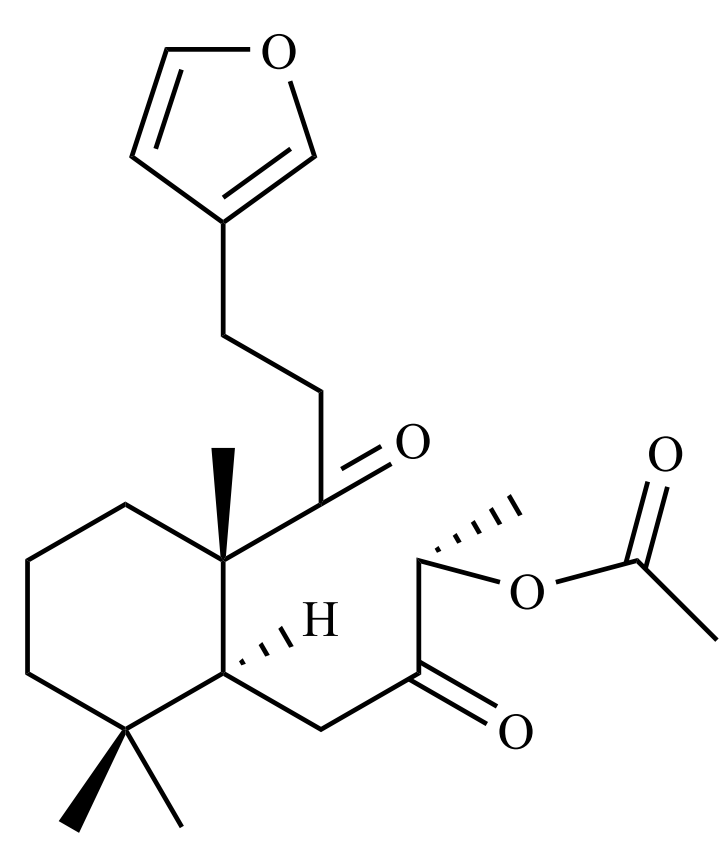

Compound **1b**

**Figure S6. Molecular docking interactions of standard drugs with VEGFR2.**

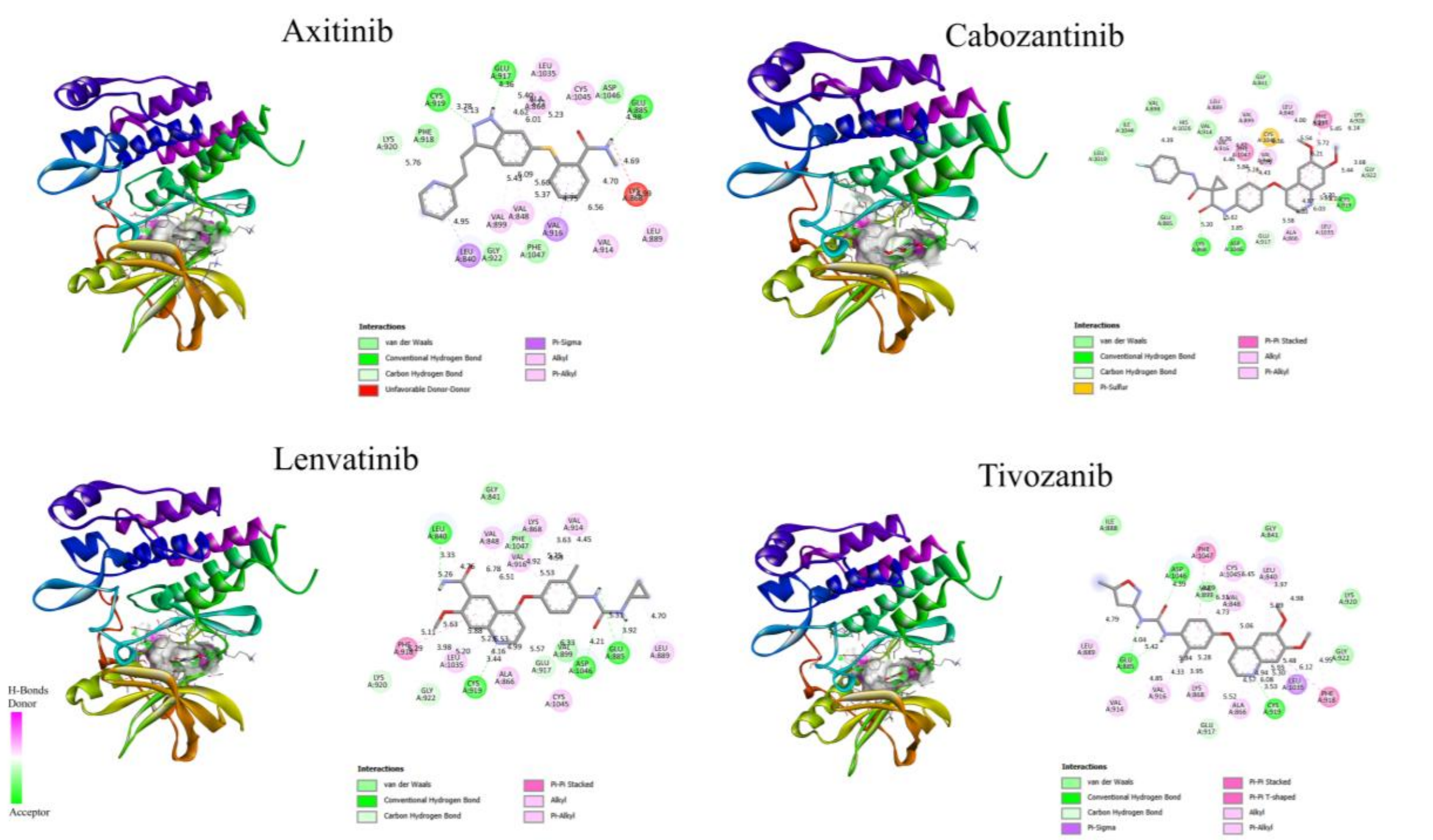


This figure presents the 2D and 3D molecular docking interactions of the standard drugs (axitinib, cabozantinib, and tivozanib) with VEGFR2 (PDB ID: 4ASE). Individual interaction maps for each drug are displayed within the 4ASE active site. Amino acid residues are indicated by their three-letter abbreviations and numbers. An interaction key highlights the specific types of bonds and accessibility: **dashed dark green lines** represent classical hydrogen bonds; **light green lines** indicate carbon-hydrogen bonds; **different shades of purple** denote pi-interactions and other weak non-covalent interactions; and a **blue cloud** illustrates solvent accessibility.

**Figure S7. Ramachandran plot results of isolated compound, sunitinib and sorafenib complexes with VEGFR2.**

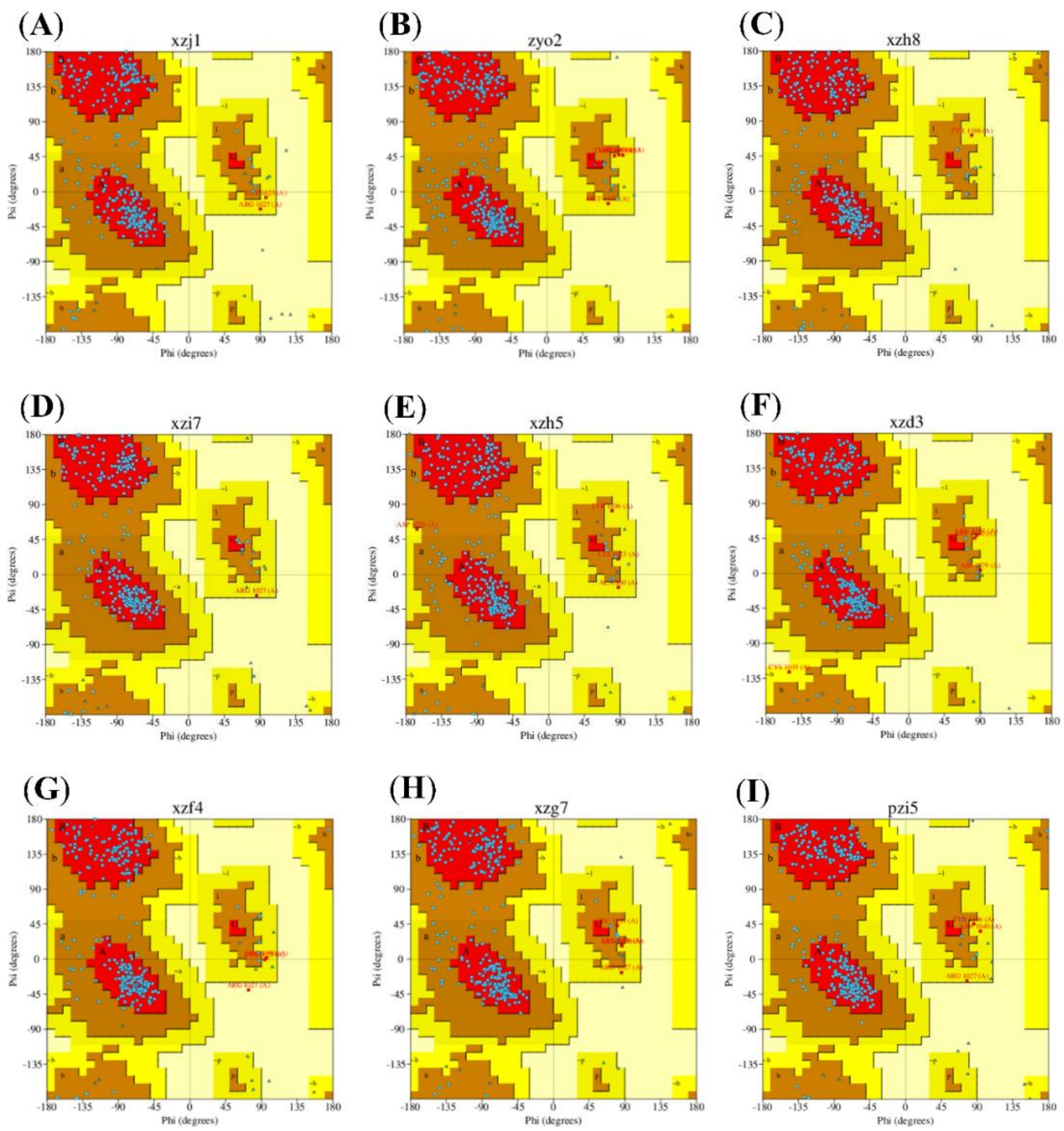


This figure displays the Ramachandran plot analysis for the VEGFR2 (PDB ID: 4ASE) complexes with isolated compounds (**1**-**6**) and the reference drug sunitinib after 100 ns molecular dynamics (MD) simulations. Each panel represents a distinct complex: (**A**) compound **1a**, (**B**) compound **1b**, (**C**) compound **2**, (**D**) compound **3**, (**E**) compound **4**, (**F**) compound **5**, (**G**) compound **6**, (**H**) sunitinib, and (**I**) sorafenib. The X-axis represents the phi dihedral angle and the Y-axis represents the psi dihedral angle.

**Figure S8. RMSF and residue contact analysis of isolated compound, sunitinib and sorafenib complexes with VEGFR2.**

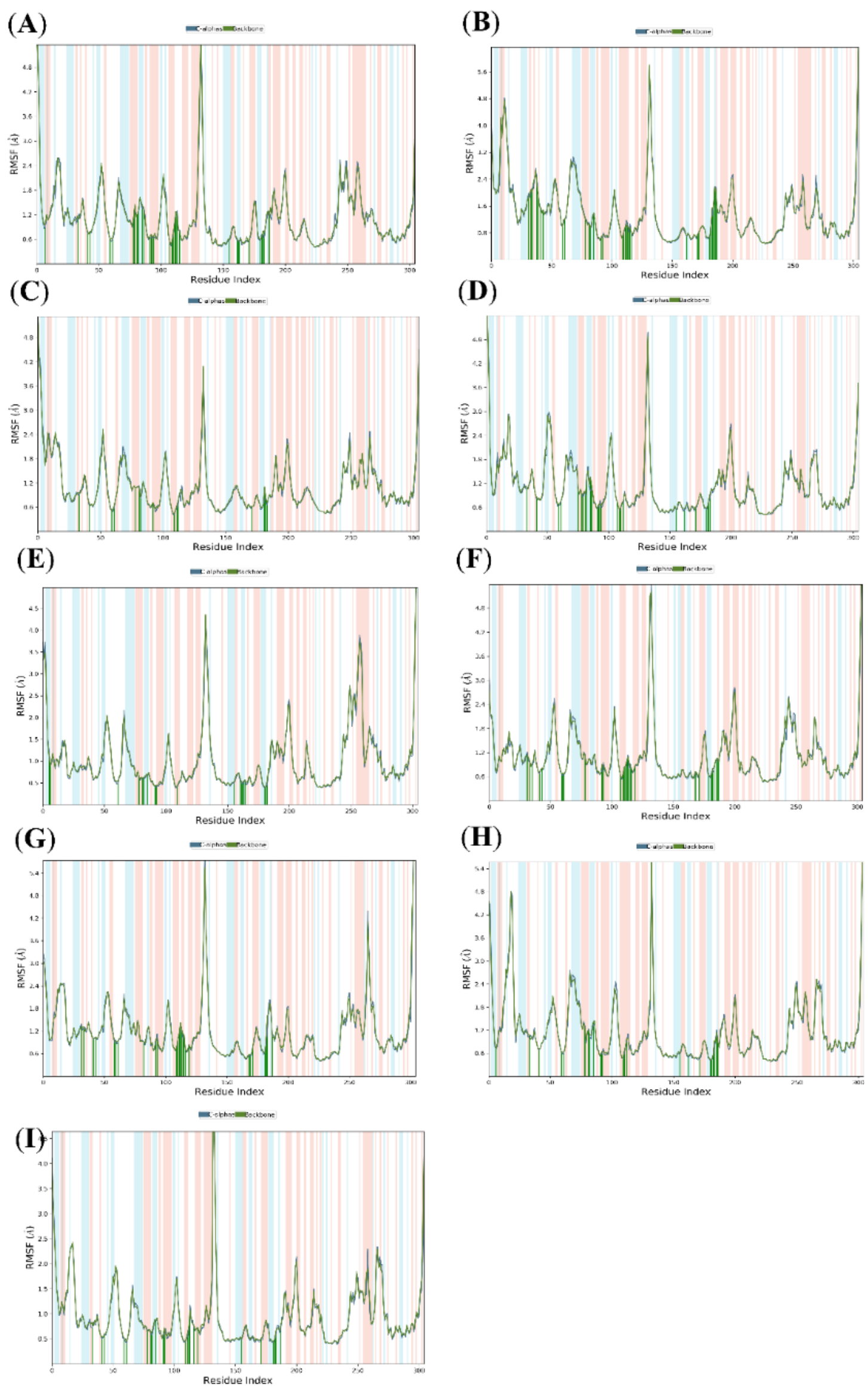


This figure displays the Root Mean Square Fluctuation (RMSF) and residue contact analysis for the VEGFR2 (PDB ID: 4ASE) complexes with isolated compounds (1-6) and the reference drug sunitinib. Each panel represents a distinct complex: (**A**) compound **1a**, (**B**) compound **1b**, (**C**) compound **2**, (**D**) compound **3**, (**E**) compound **4**, (**F**) compound **5**, (**G**) compound **6**, (**H**) sunitinib, and (**I**) sorafenib. The X-axis represents residue index, and the Y-axis represents the RMSF (Å). The **green and blue lines** represent the backbone and C-alpha regions of proteins, respectively.

**Figure S9. Ligand interaction properties of isolated compounds sunitinib and sorafenib complexes with VEGFR2 during MD simulation.**

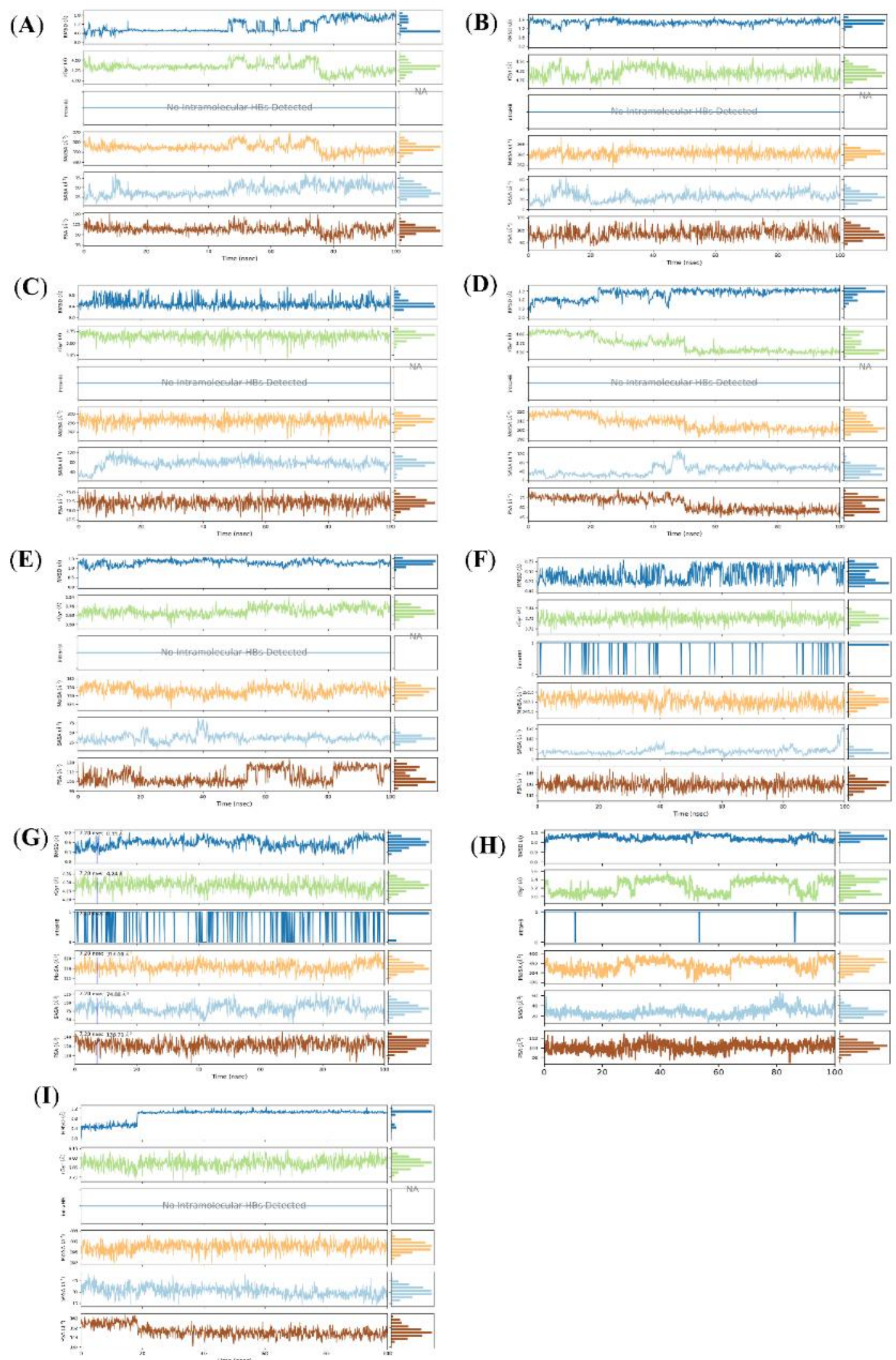

Each panel represents a distinct complex, showing its properties over time: **(A)** compound **1a**, **(B)** compound **1b**, **(C)** compound **2**, **(D)** compound **3**, **(E)** compound **4**, **(F)** compound **5**, **(G)** compound **6**, (**H**) sunitinib, and (**I**) sorafenib. The properties shown include Root Mean Square Deviation (RMSD), radius of gyration (rGyr), intramolecular hydrogen bonds (intraHB), Molecular Surface Area (MoISA), solvent accessible surface area (SASA), and polar surface area (PSA). The X-axis represents time (ns), and the Y-axis represents the corresponding property value.

**Figure S10. Conformational changes of isolated compounds and sunitinib complexes during molecular dynamics simulation**.

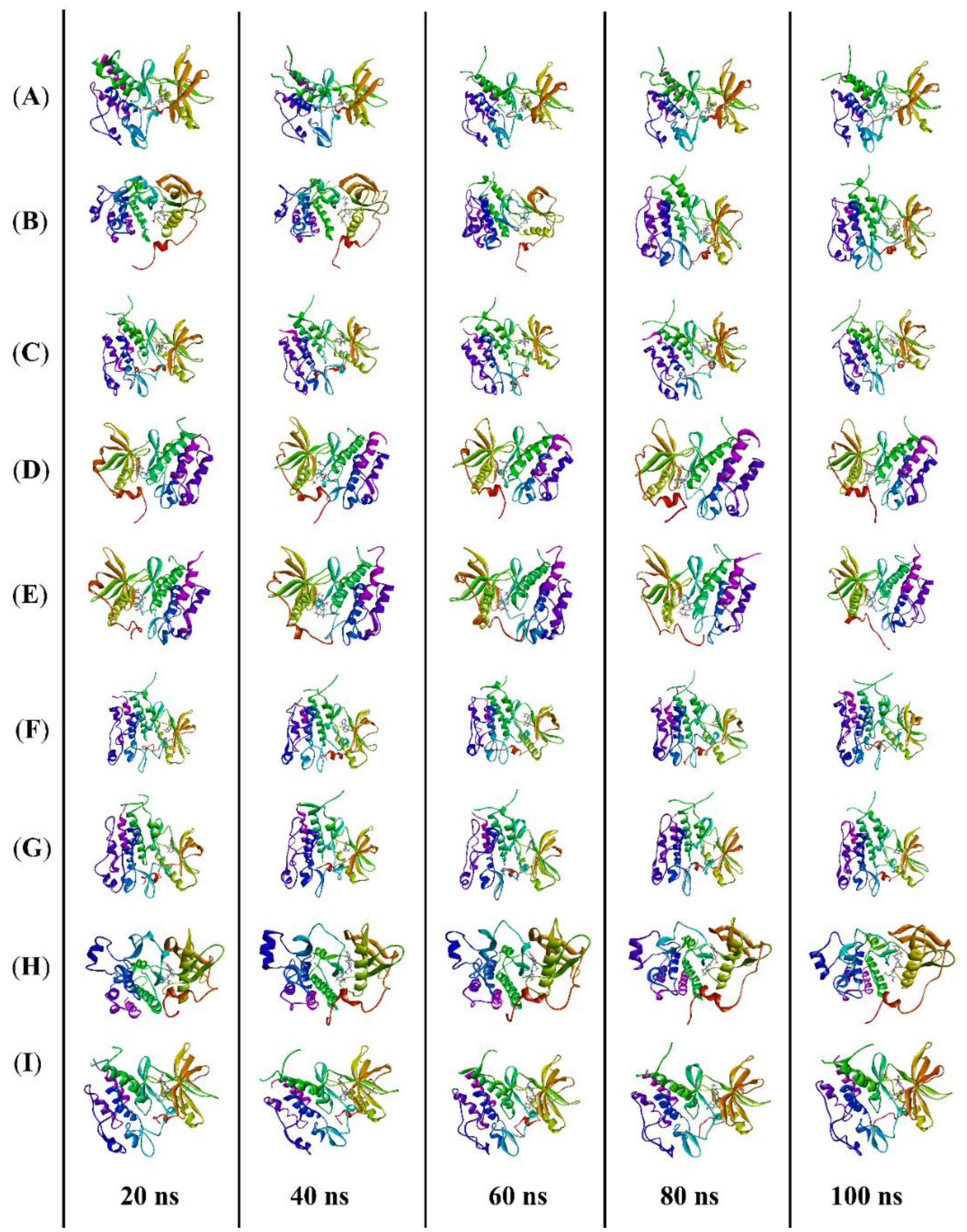


This figure visually illustrates the trajectory and conformational changes observed in the VEGFR2 (PDB ID: 4ASE) complexes with isolated compounds (1-6) and the reference drug sunitinib over the course of the molecular dynamics (MD) simulation. Each panel represents a distinct complex: **(A)** compound **1a**, **(B)** compound **1b**, **(C)** compound **2**, **(D)** compound **3**, **(E)** compound **4**, **(F)** compound **5**, **(G)** compound **6,** (**H**) sunitinib, and (**I**) sorafenib.

**Figure S11. $^1$H NMR spectrum of compound 1**

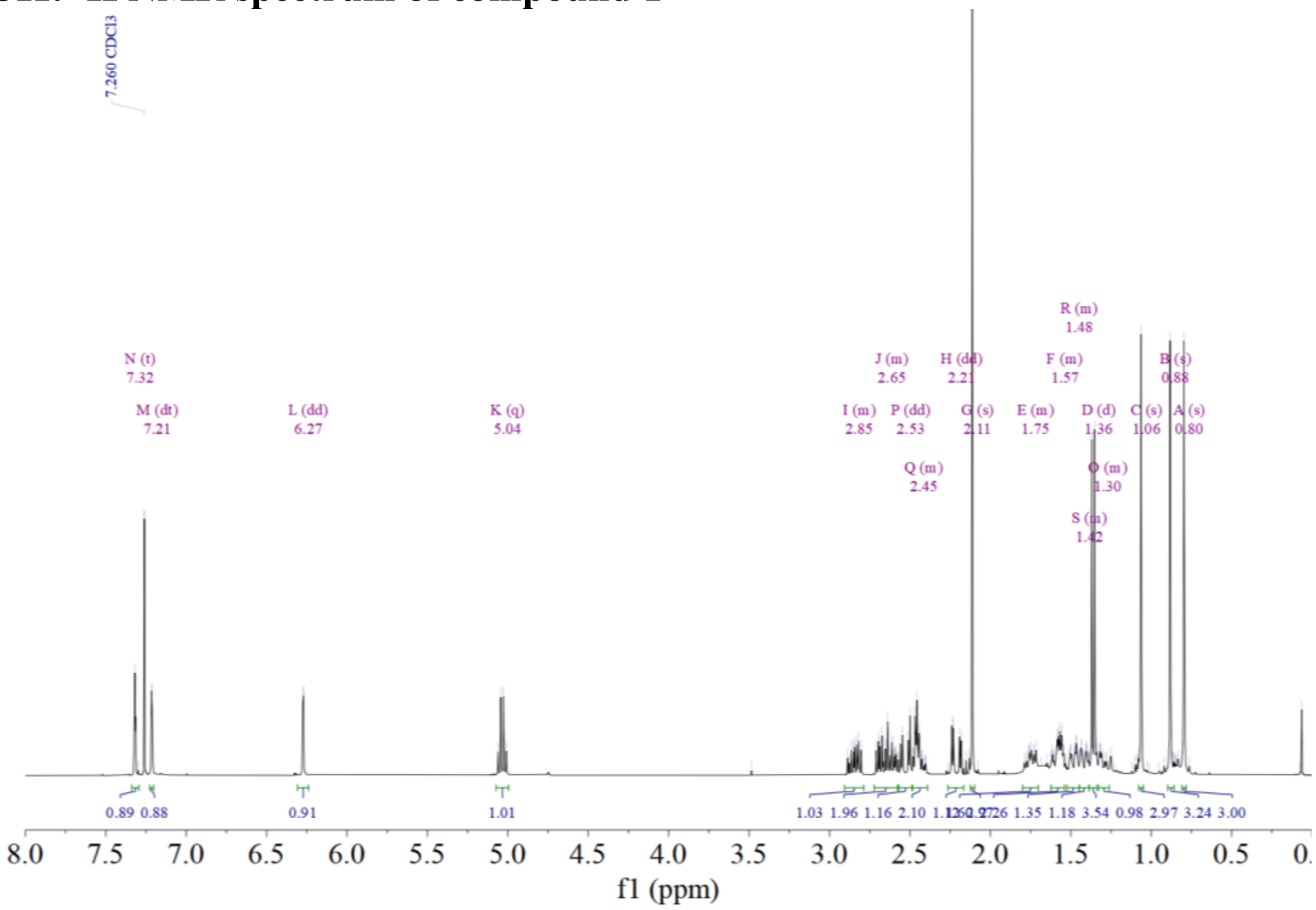


**Figure S12. $^{13}$C NMR spectrum of compound 1**

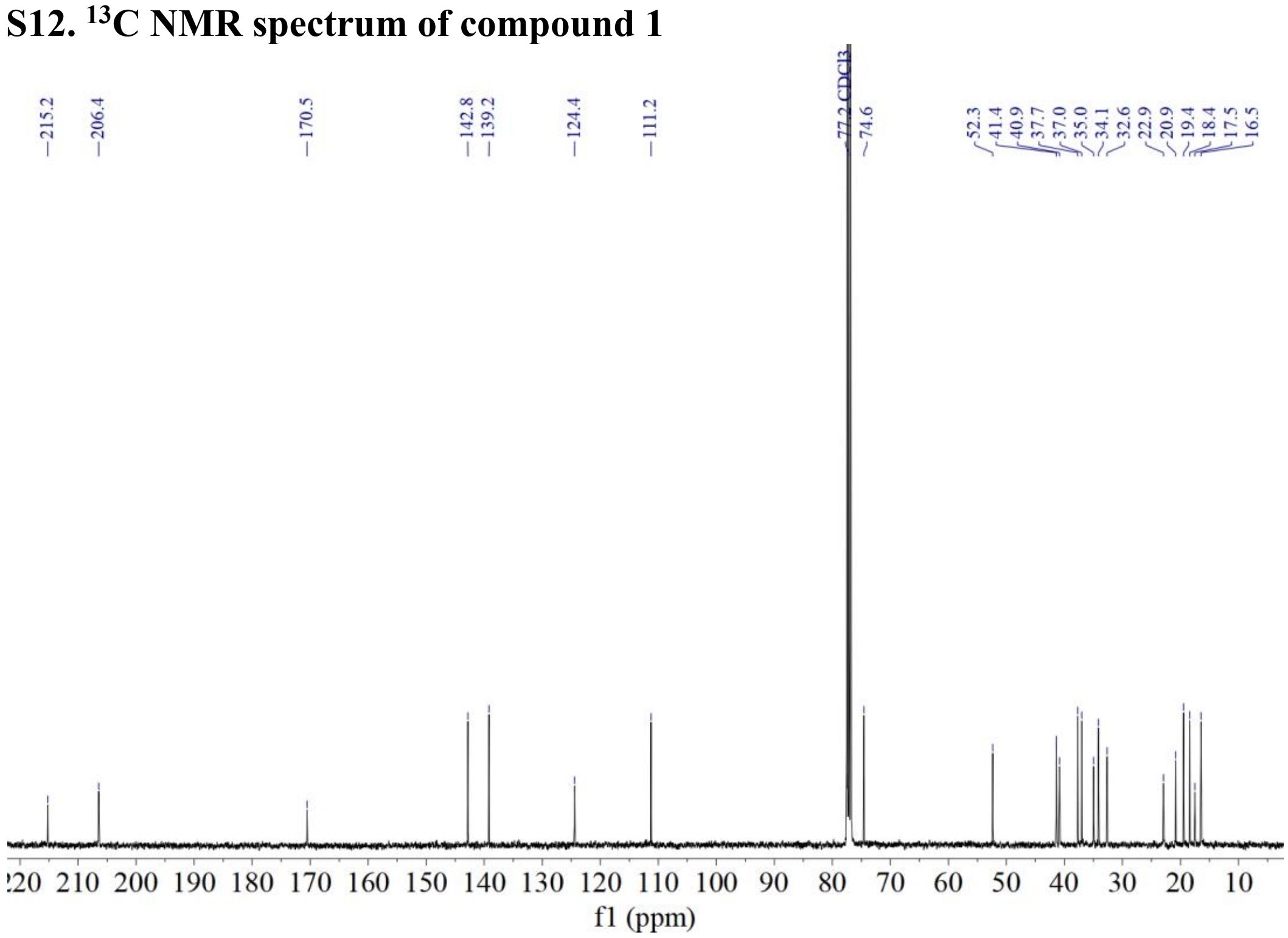

**Figure S13. DEPT NMR spectrum of compound 1**

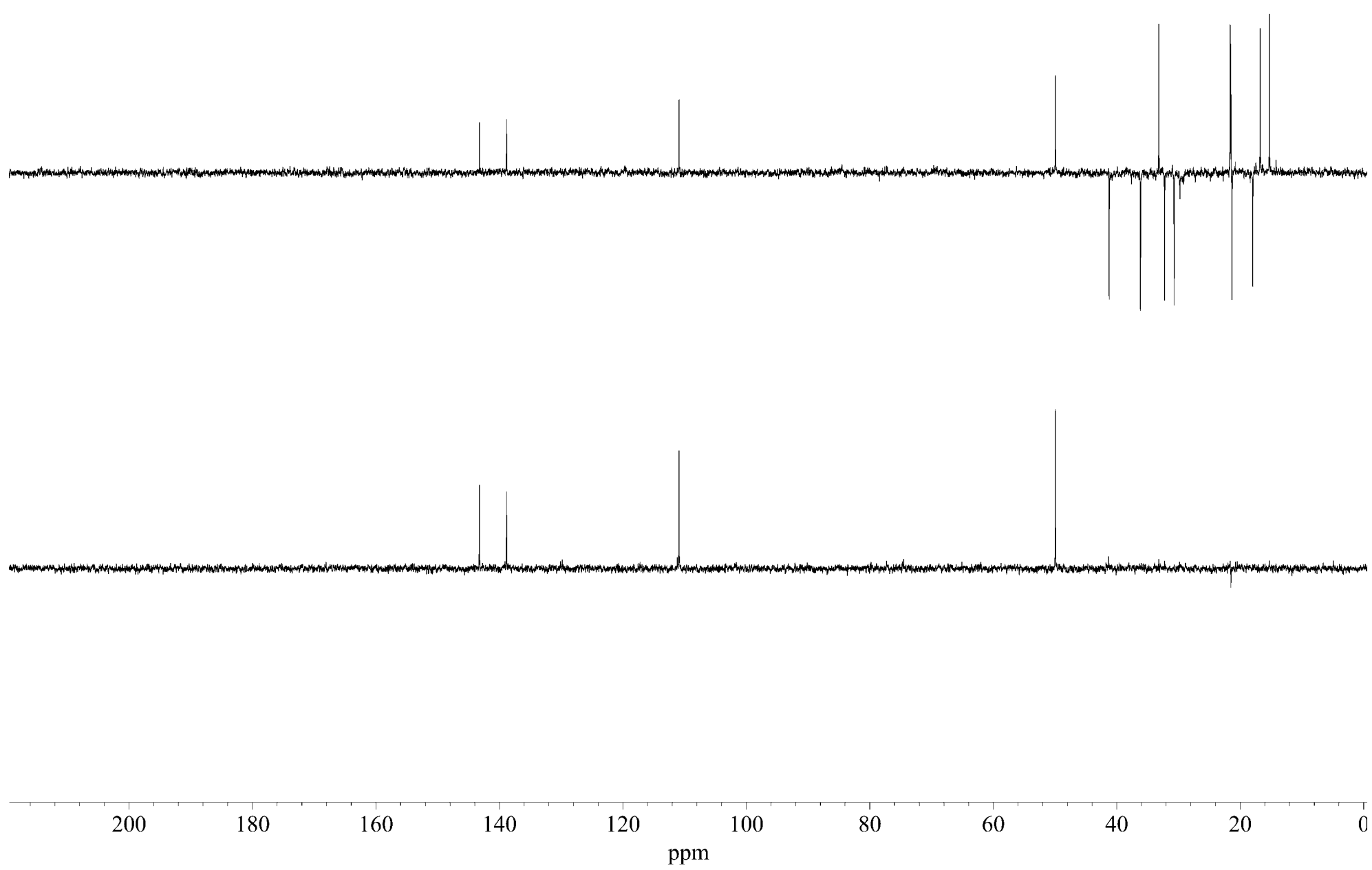


**Figure S14. COSY spectrum of compound 1**

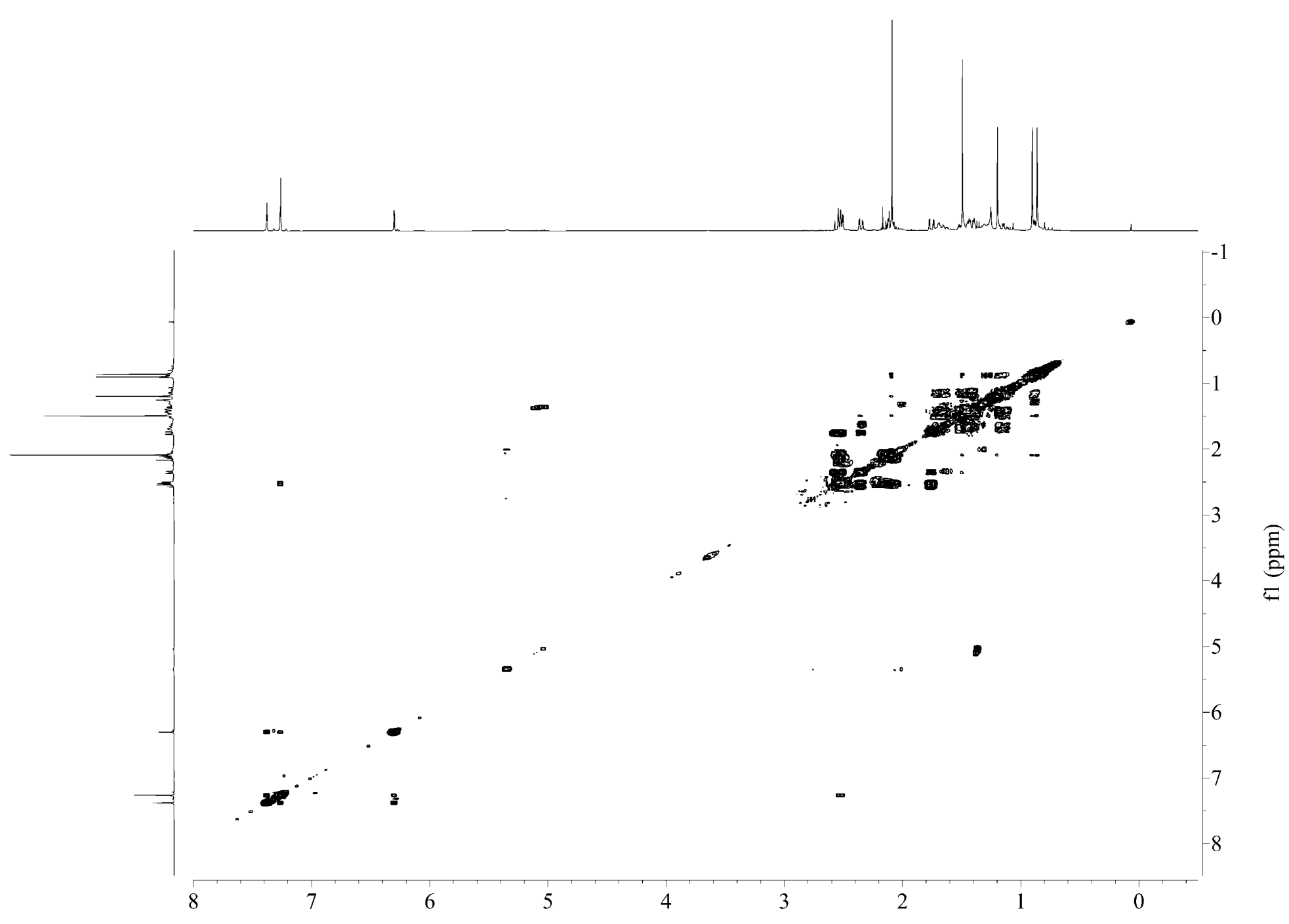

**Figure S15. HSQC spectrum of compound 1**

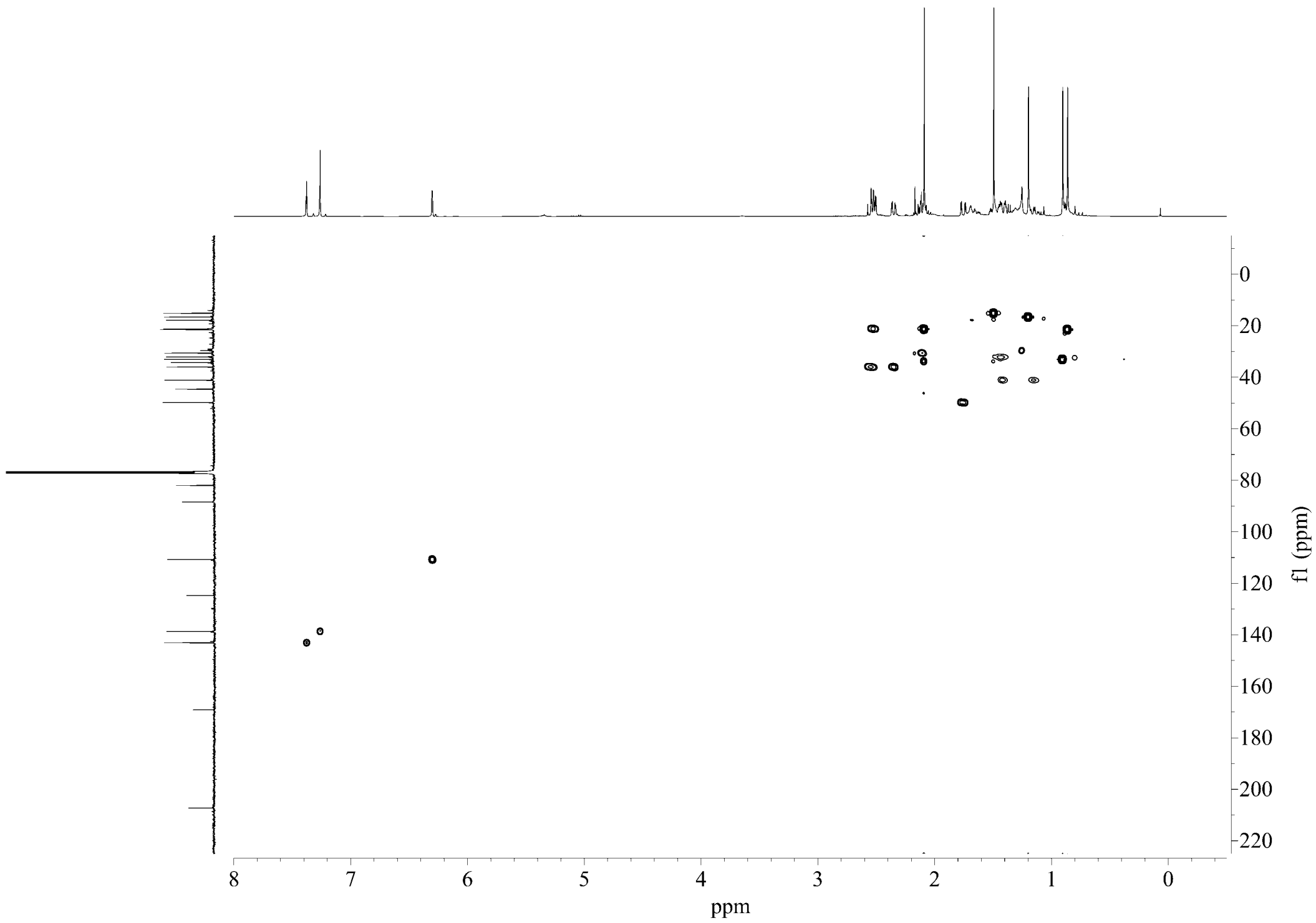


**Figure S16. HMBC spectrum of compound 1**

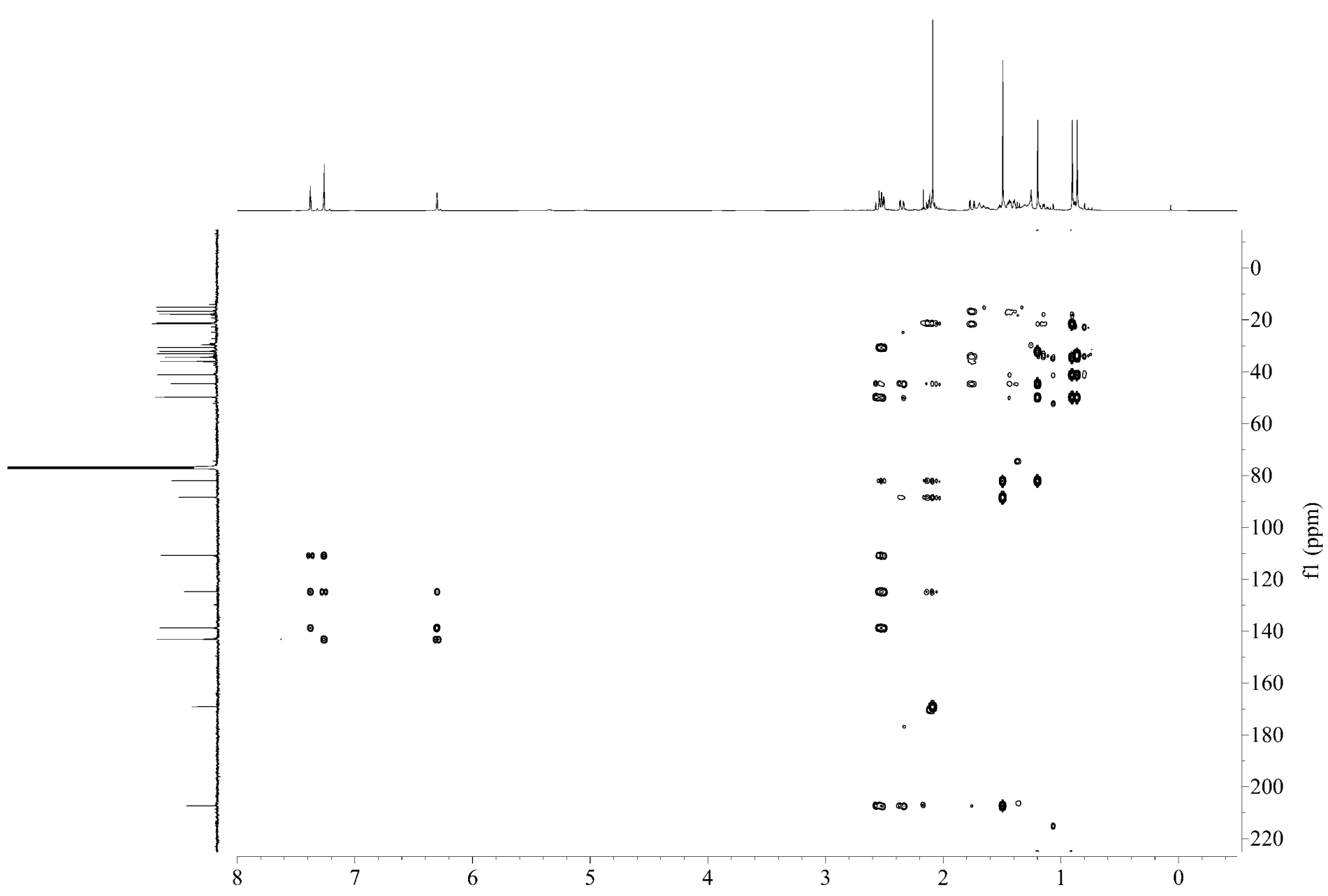

**Figure S17. NOESY spectrum of compound 1**

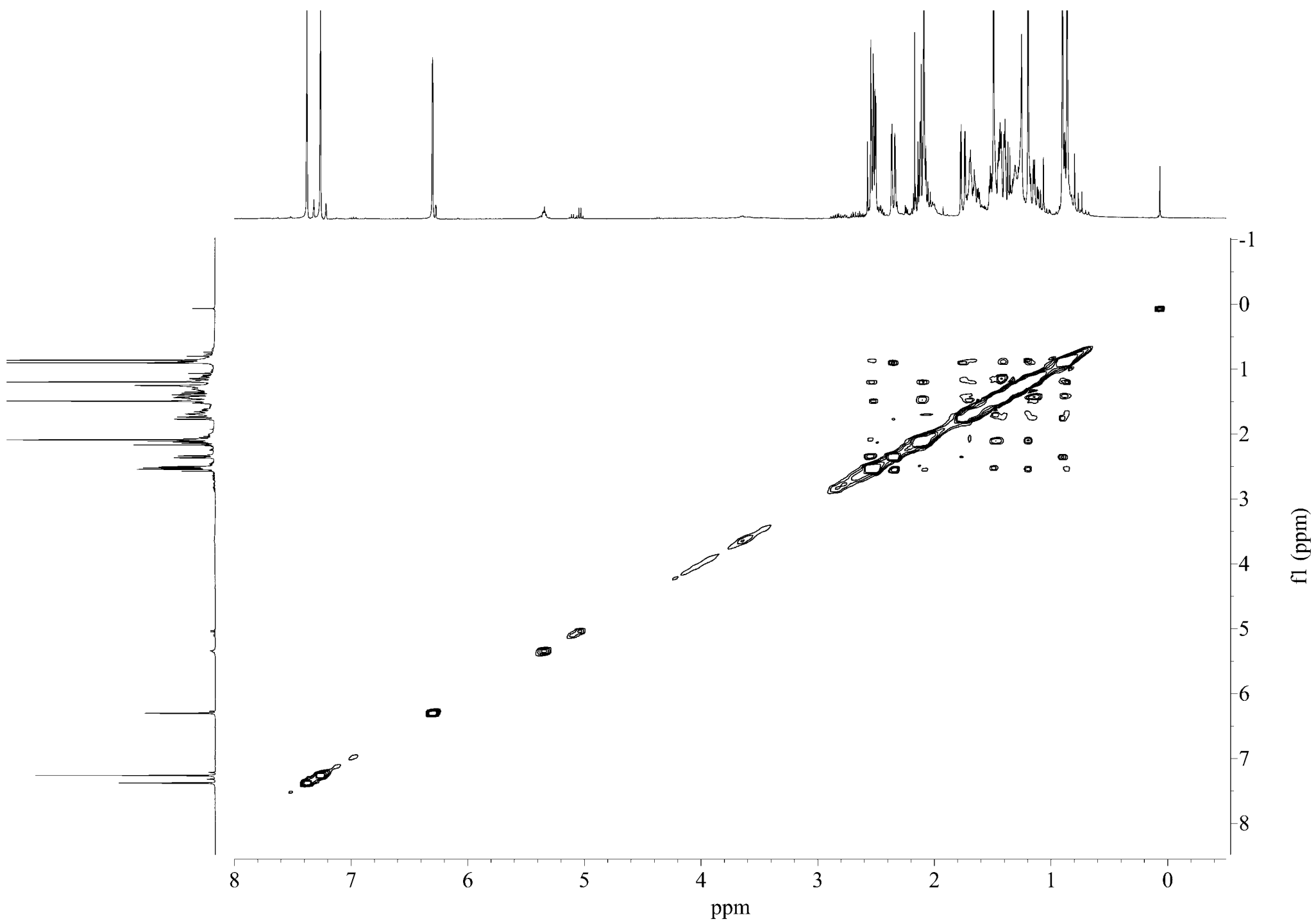


**Figure S18. HRESI-MS spectrum of compound 1**

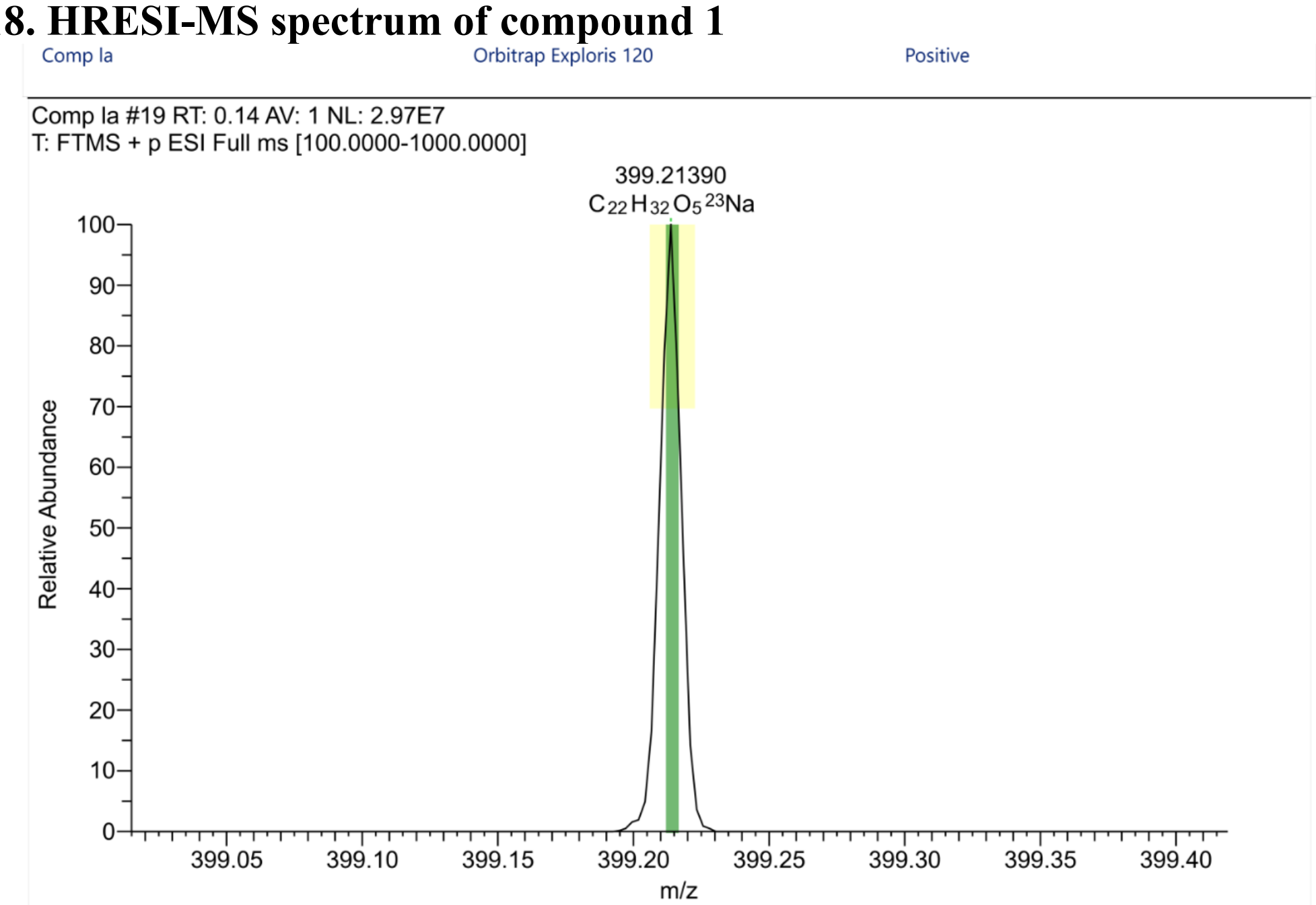


| Peak Mass | Display Formula | S Fit | RDB | Delta [ppm] | Theo. mass | Pattern Cov. [%] | MSMS Matched... |
|---|---|---|---|---|---|---|---|
| 399.21390 | $C_{22}H_{32}O_5{}^{23}Na$ | 79.5263603861601 | 6.50 | -0.75 | 399.21420 | 100 | (Collection) |

**Figure S19. UV and IR spectrum of compound 1**

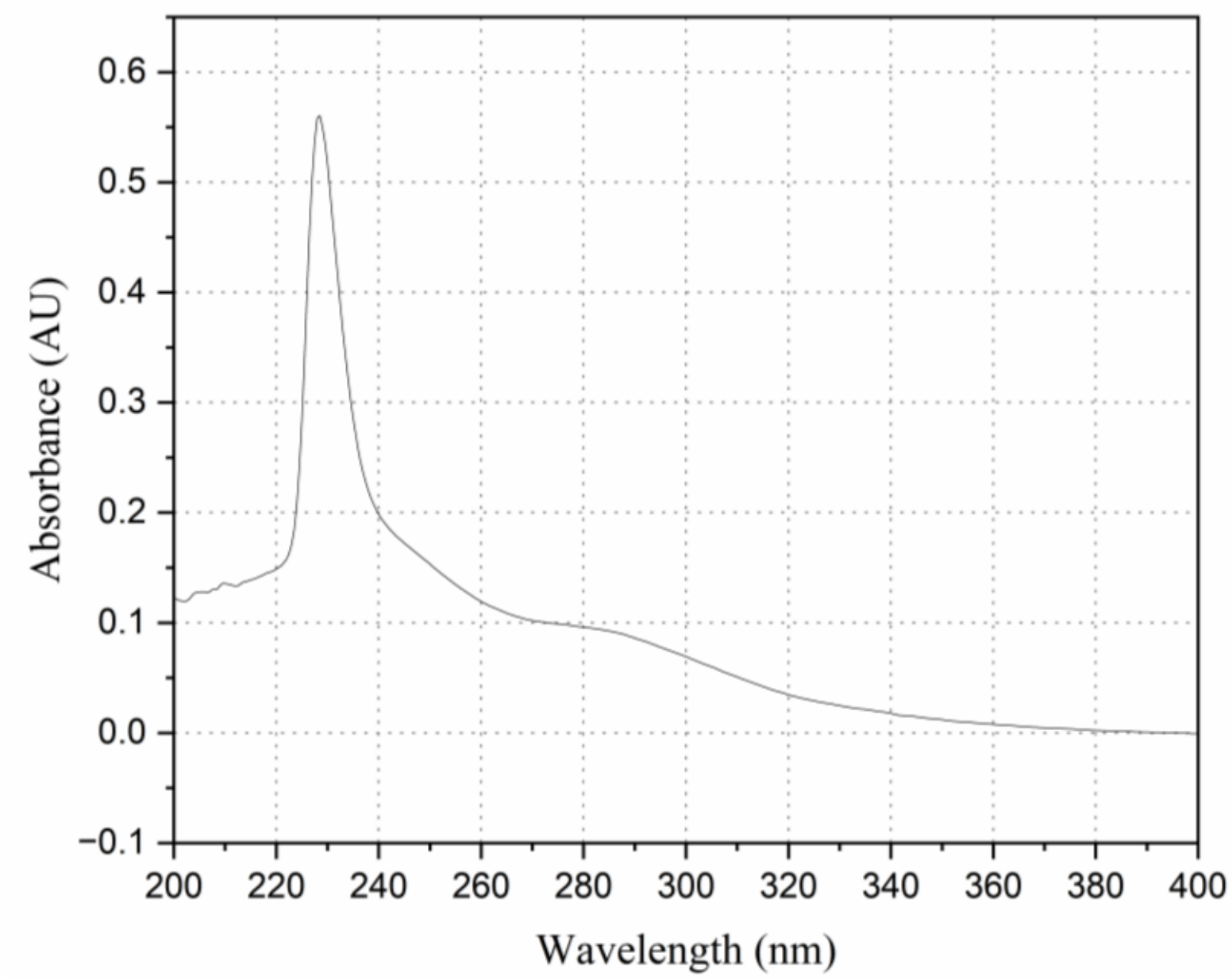


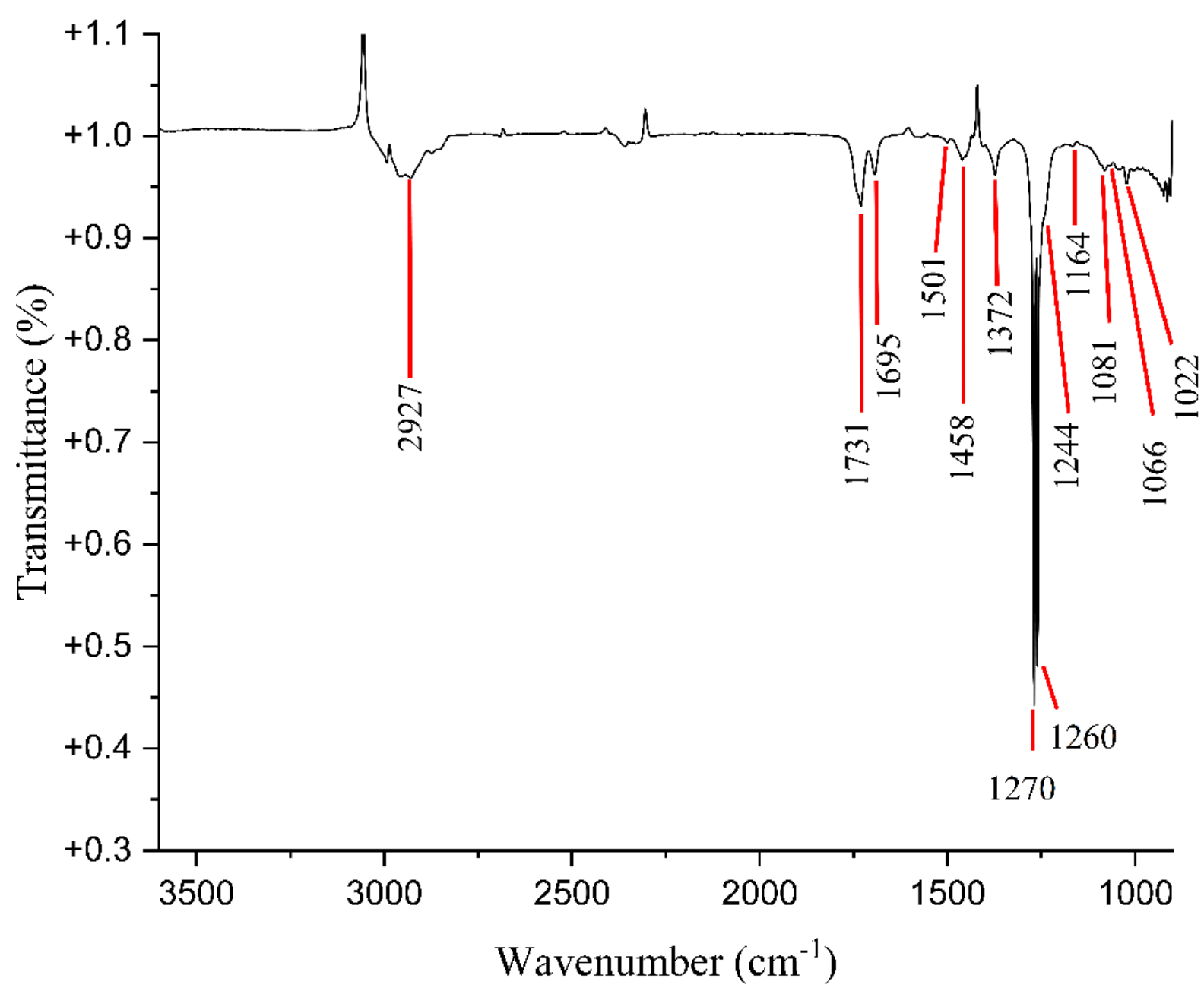

**Figure S20. $^{1}$H NMR spectrum of compound 2**

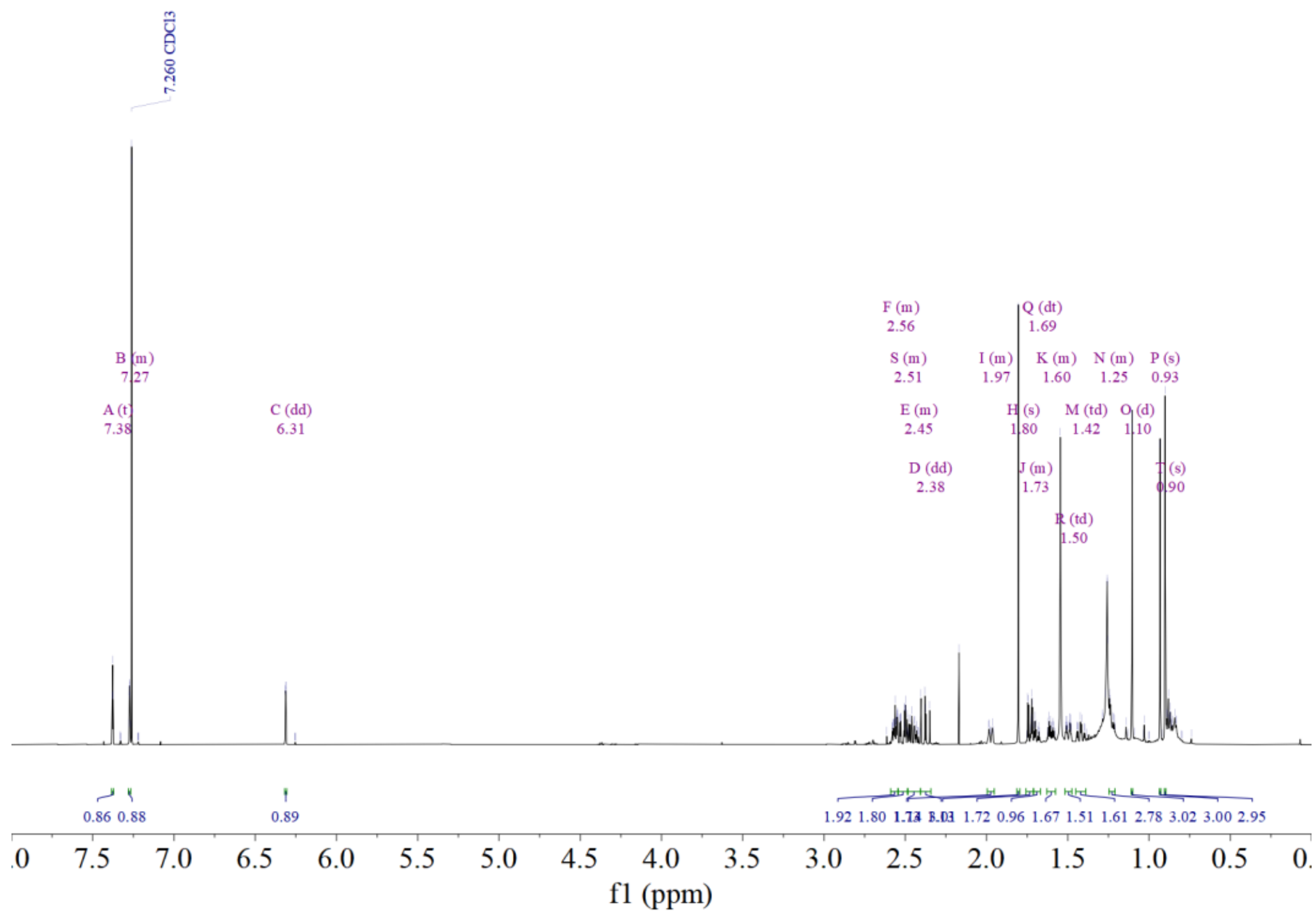


**Figure S21. $^{13}$C NMR spectrum of compound 2**

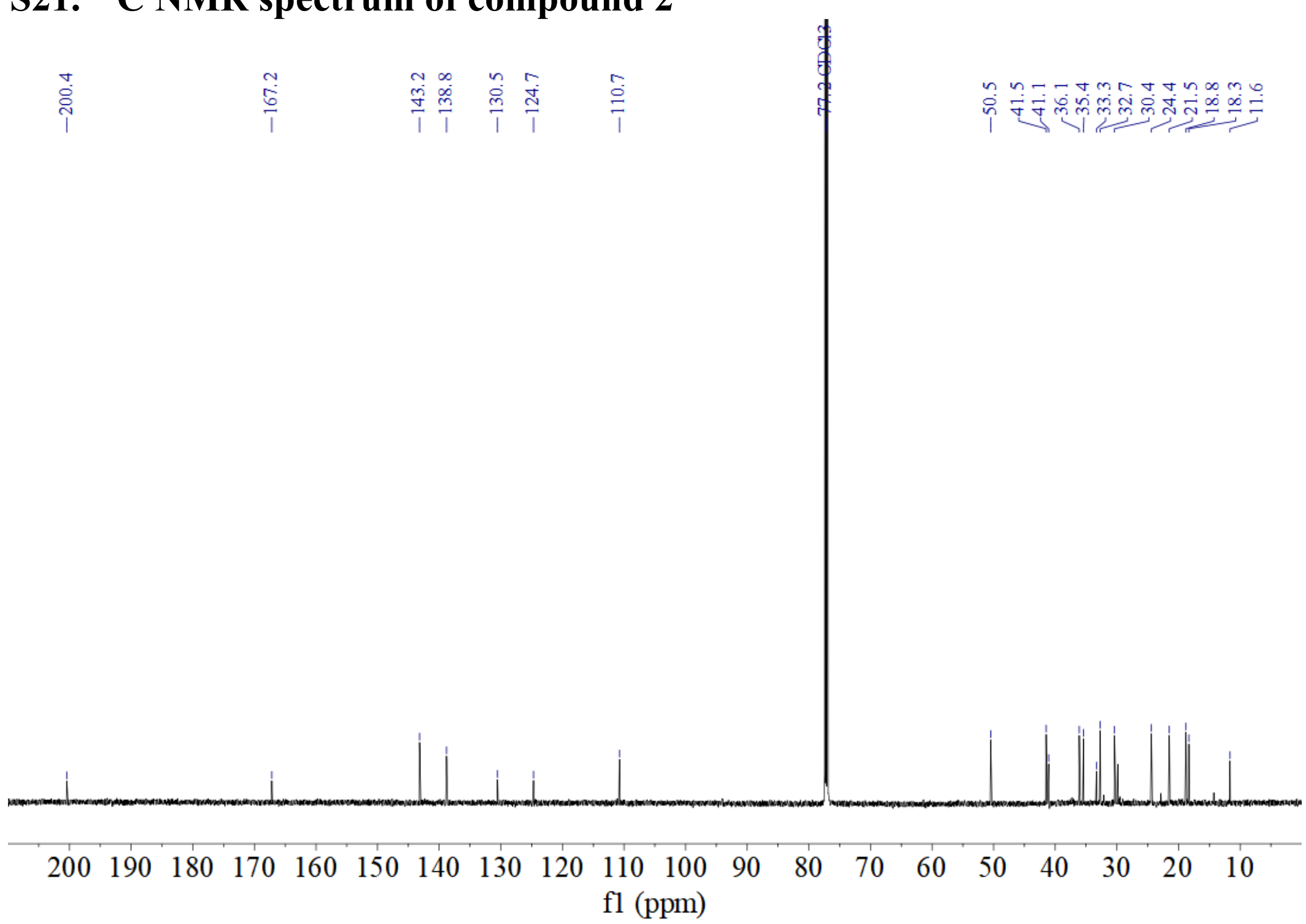

**Figure S22. DEPT NMR spectrum of compound 2**

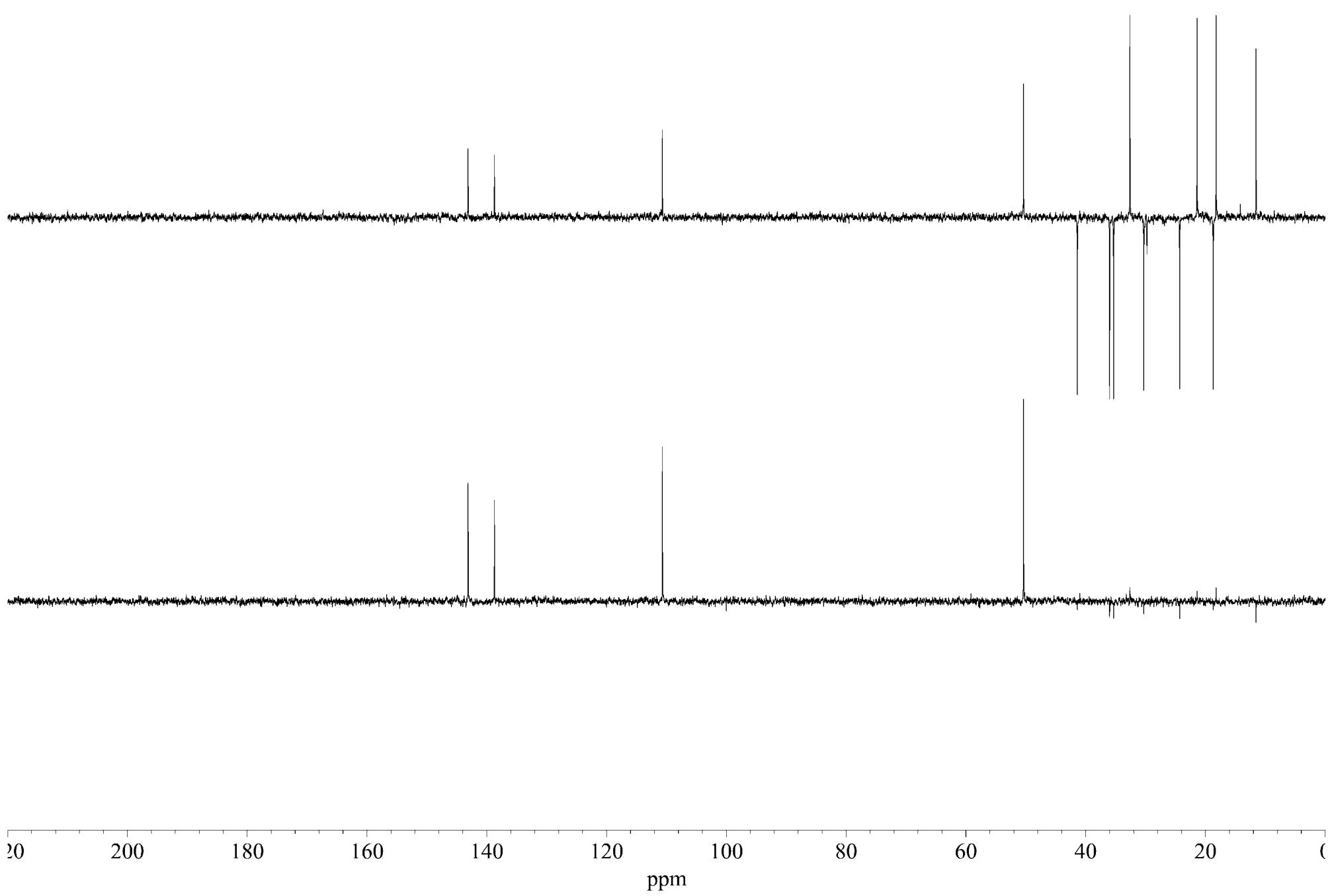


**Figure S23. COSY spectrum of compound 2**

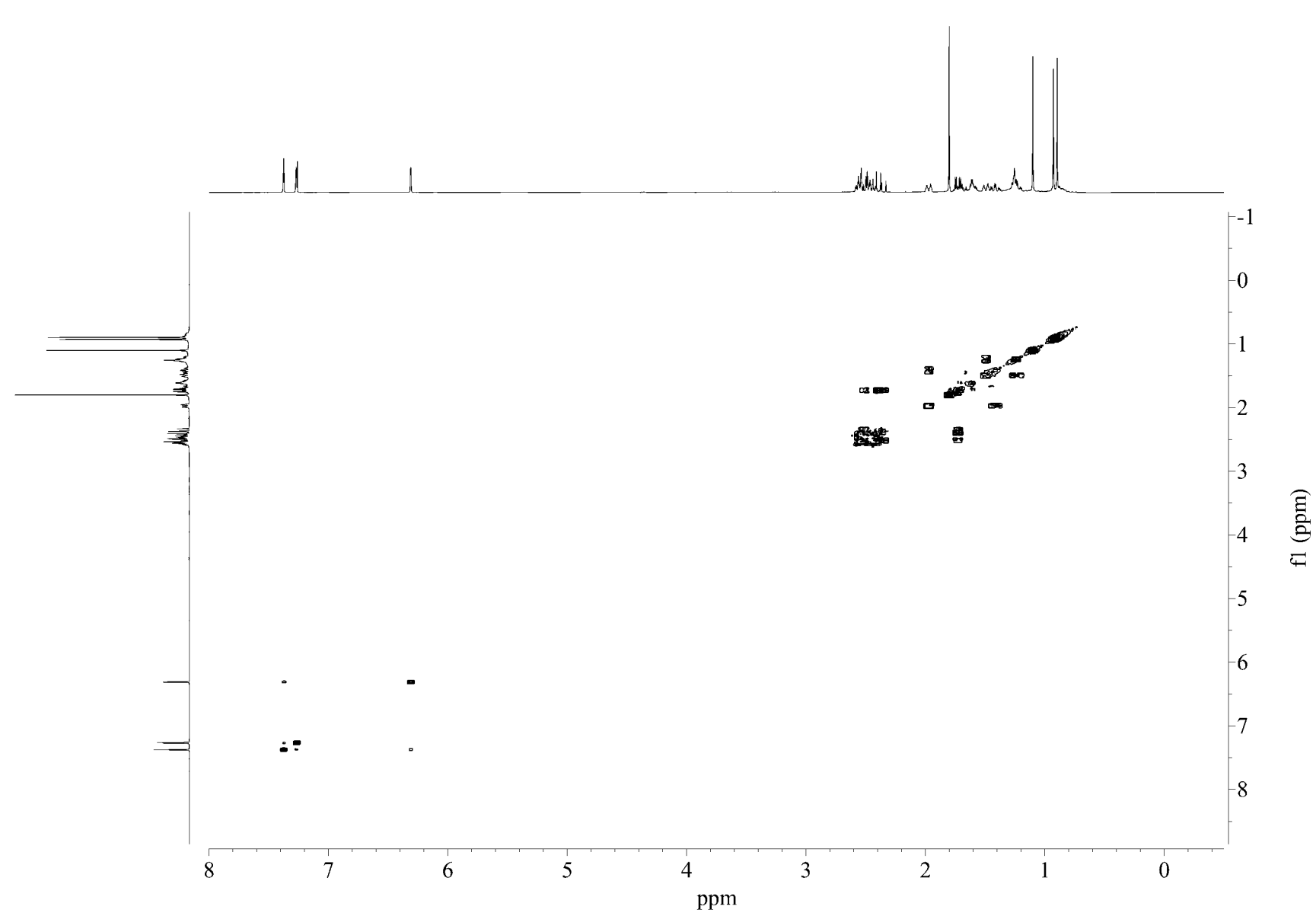

**Figure S24. HMQC spectrum of compound 2**

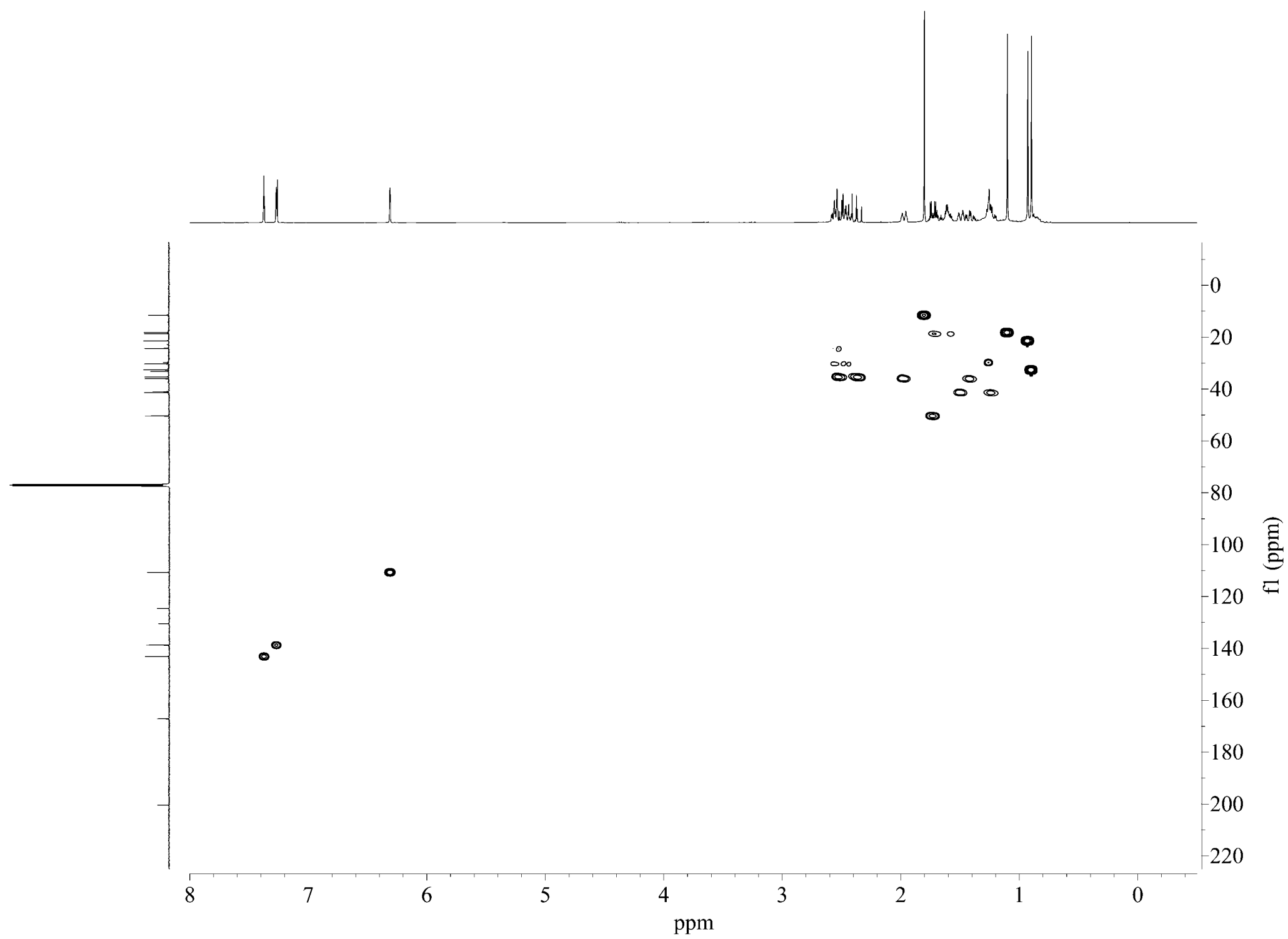


**Figure S25. HMBC spectrum of compound 2**

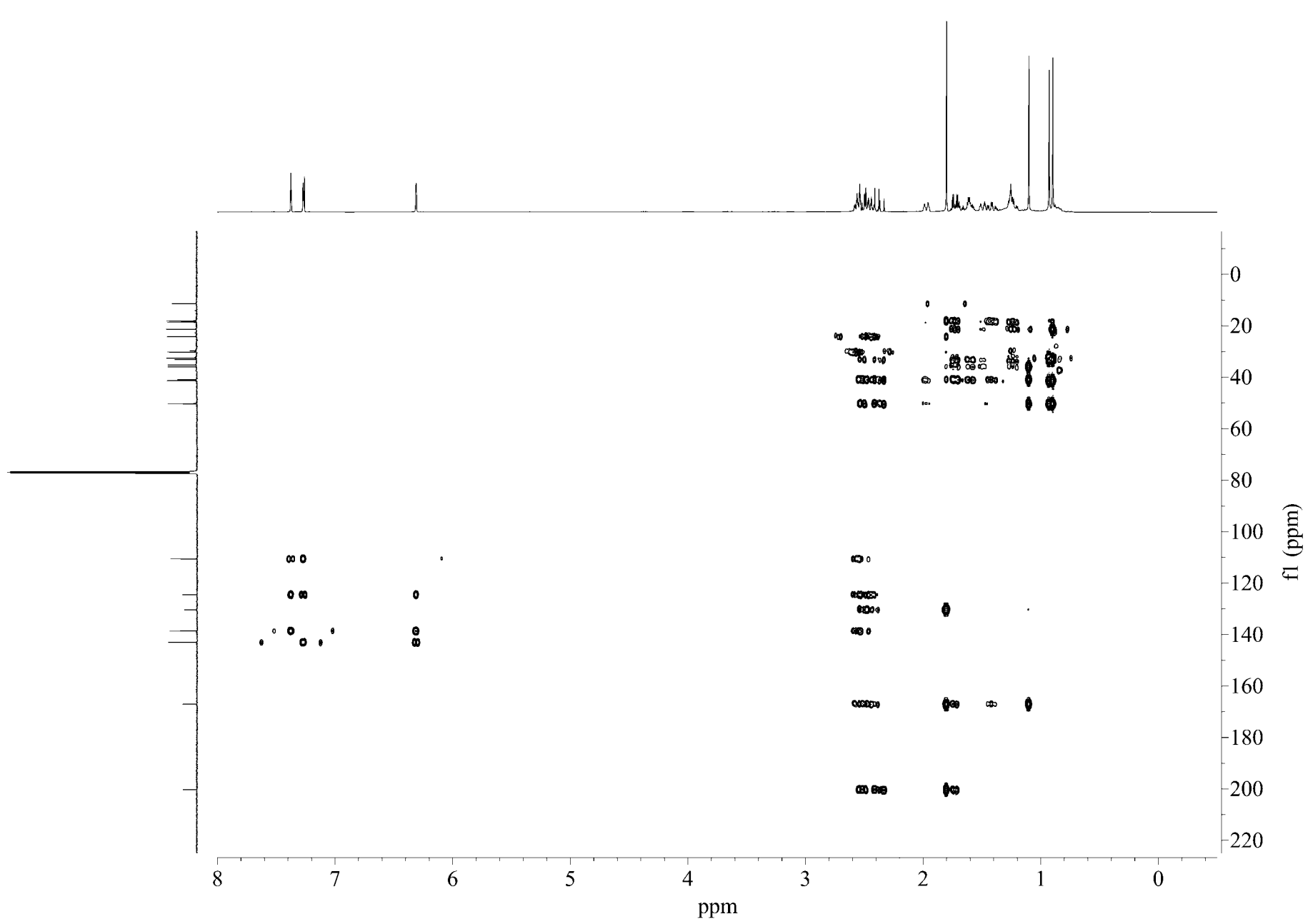

**Figure S26. NOESY spectrum of compound 2**

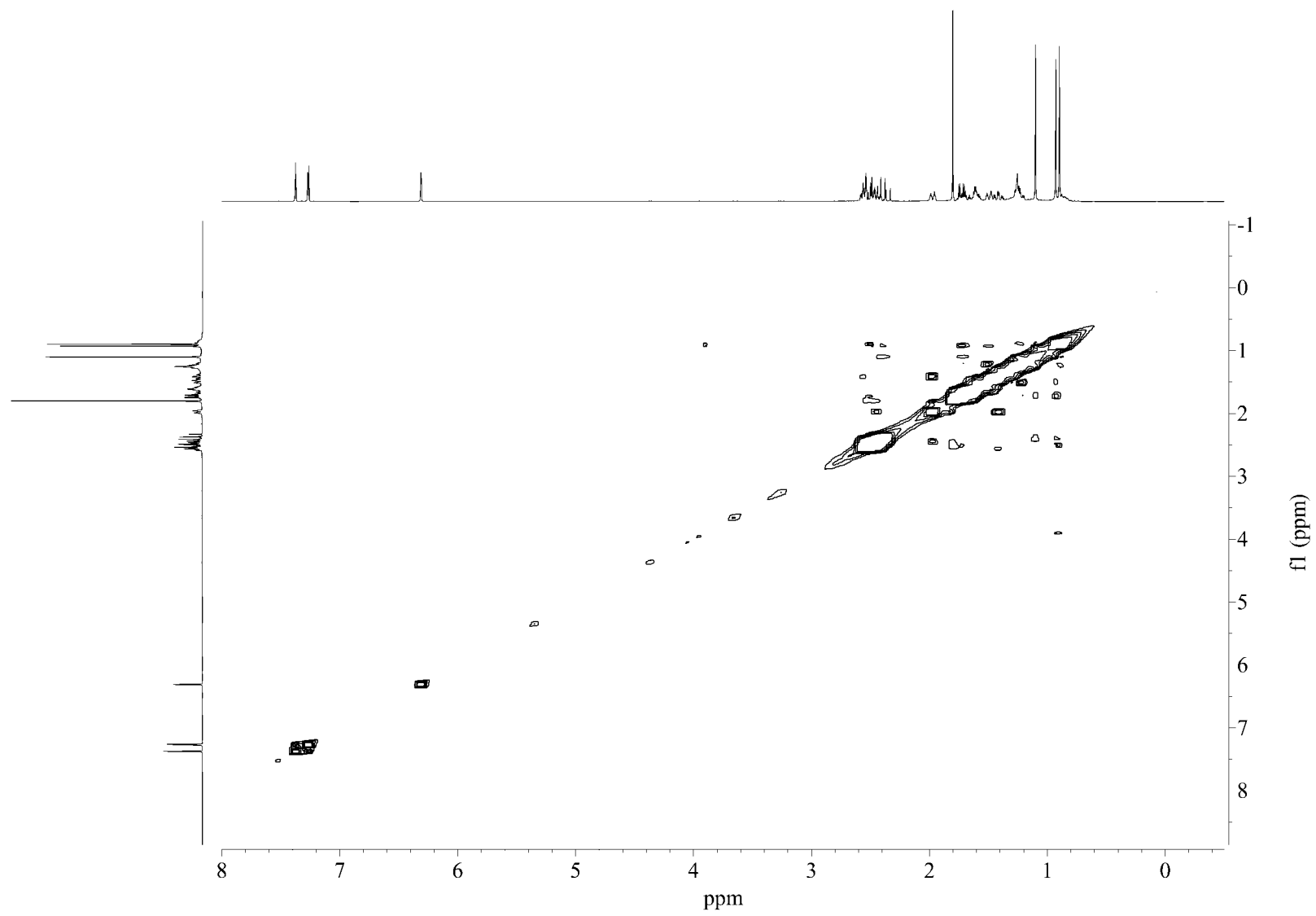


**Figure S27. ESI-MS spectrum of compound 2**

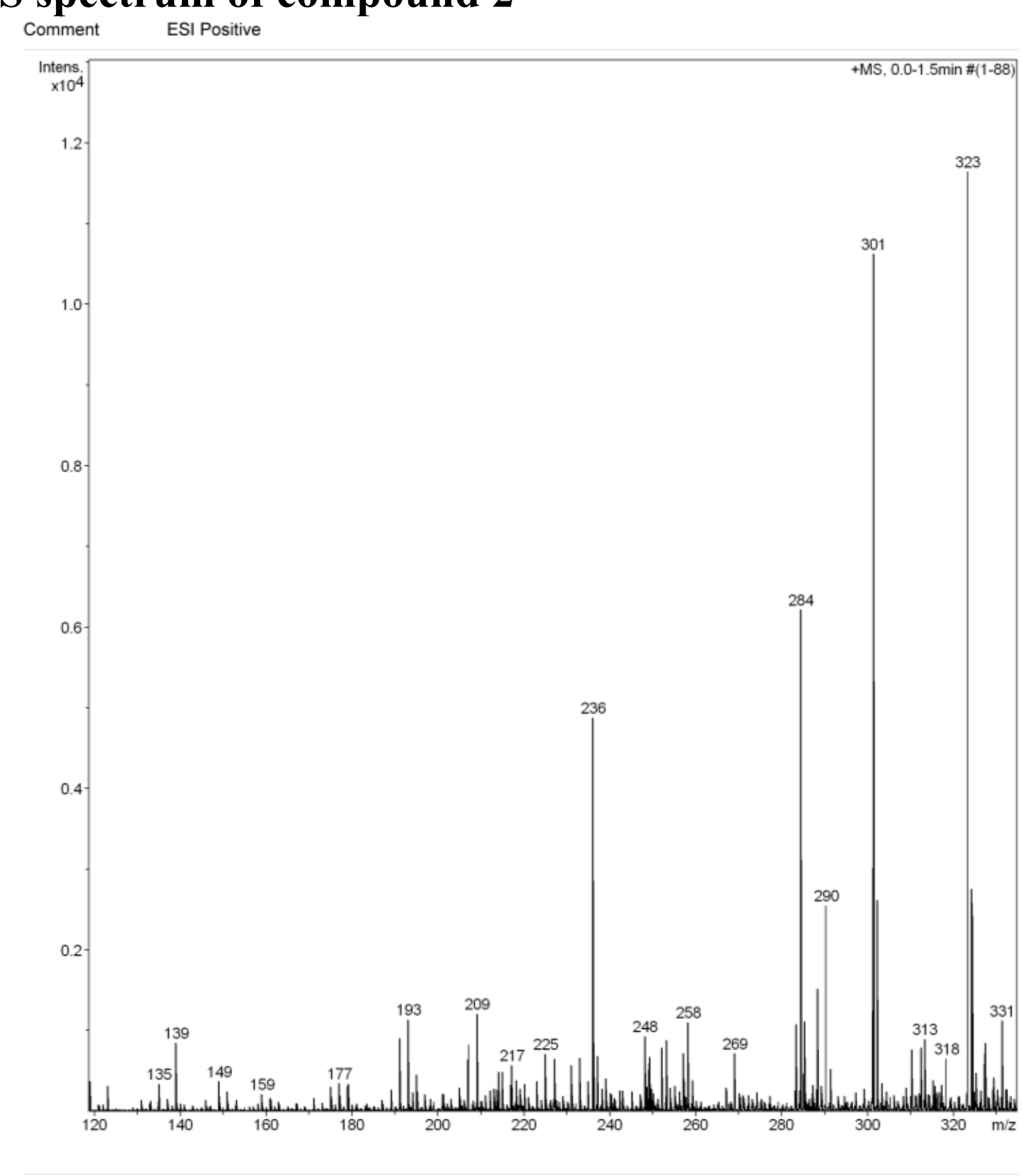

**Figure S28. UV and IR spectrum of compound 2**

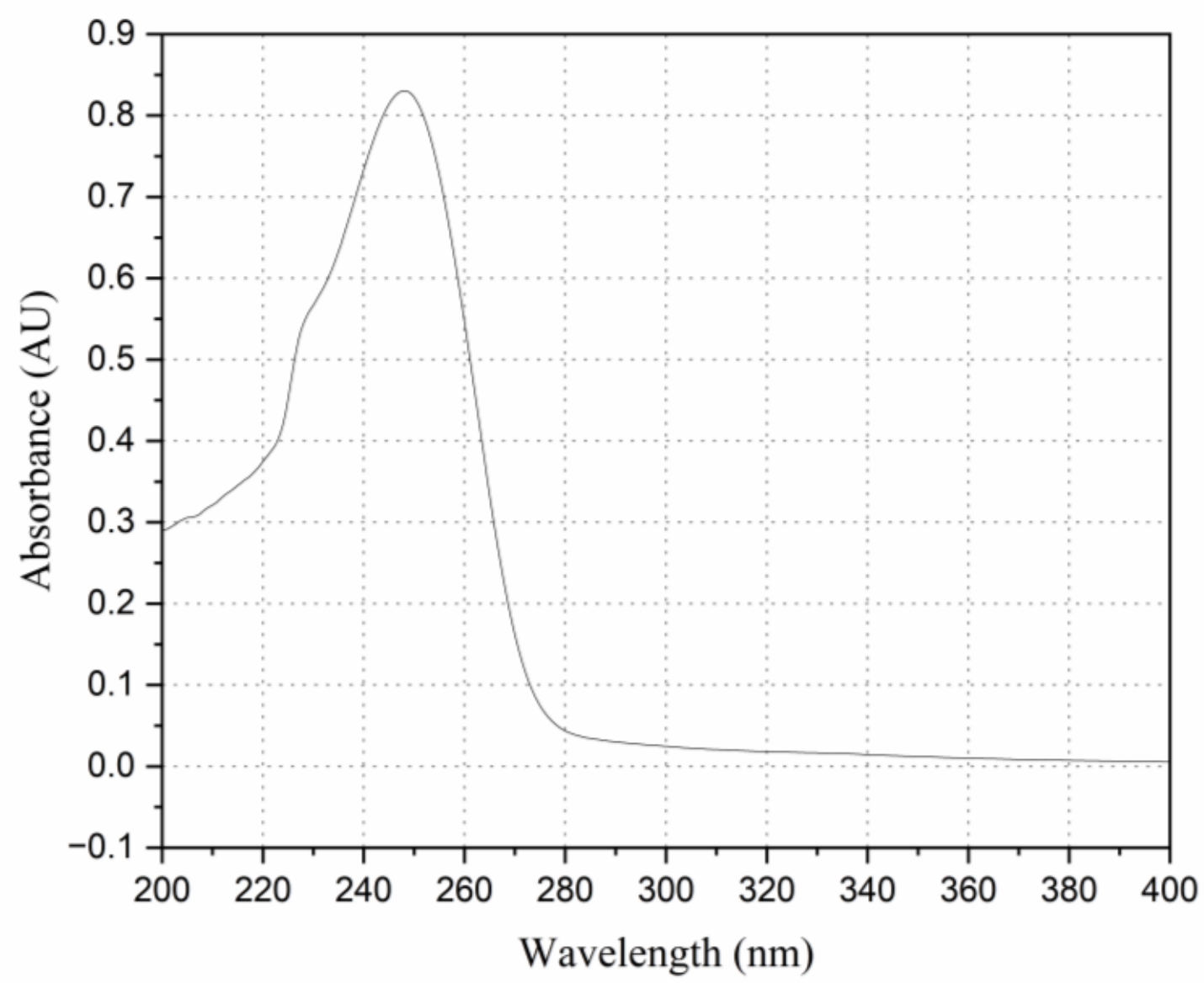


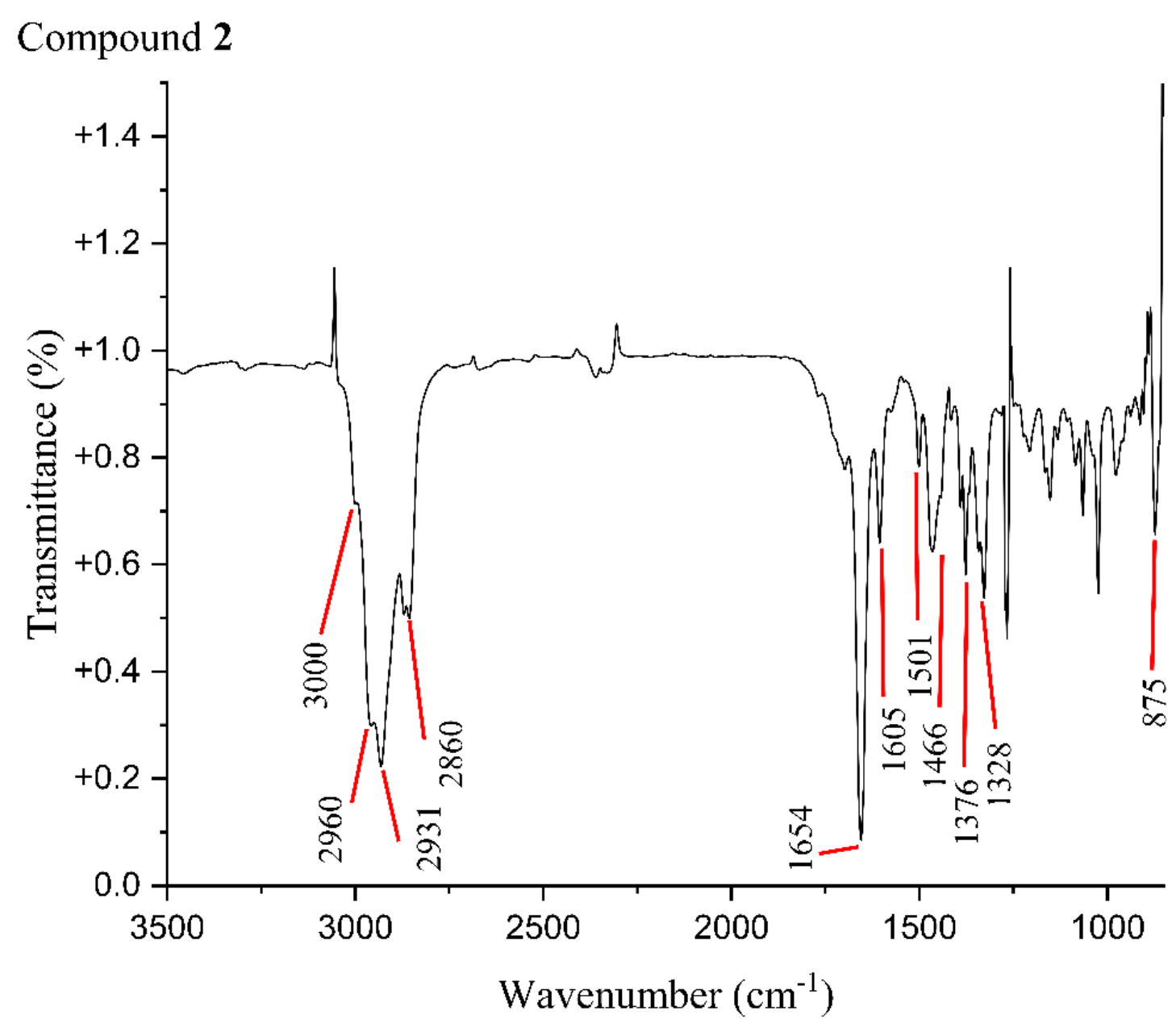


IR ($CH_2Cl_2$) $\nu_{max}$ 3000, 2960, 2931, 2860, 1654, 1605, 1501, 1466, 1376, 1328, 1024, 875 cm$^{-1}$.

**Figure S29. $^{1}$H NMR spectrum of compound 3**

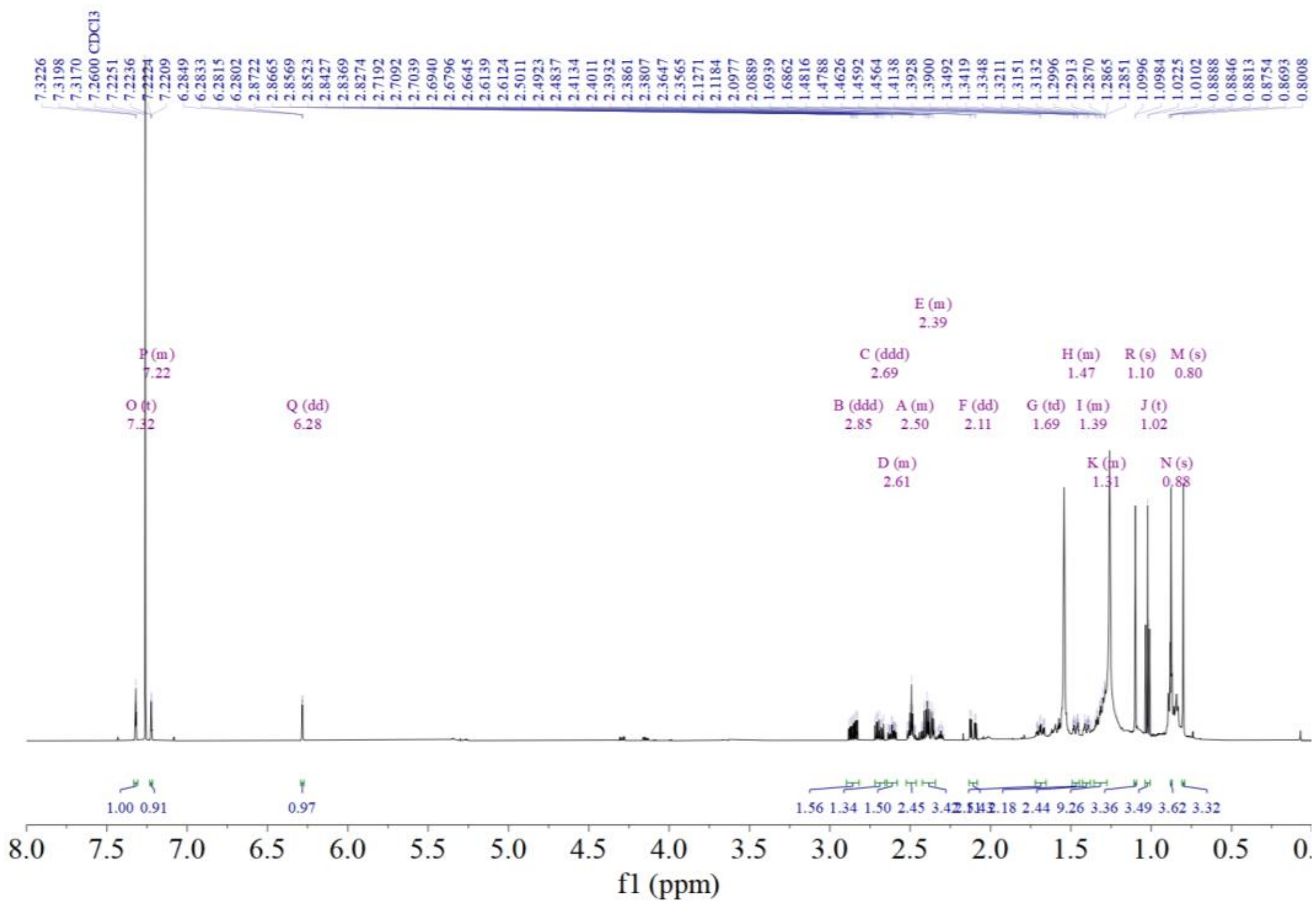

**Figure S30. $^{13}$C NMR spectrum of compound 3**

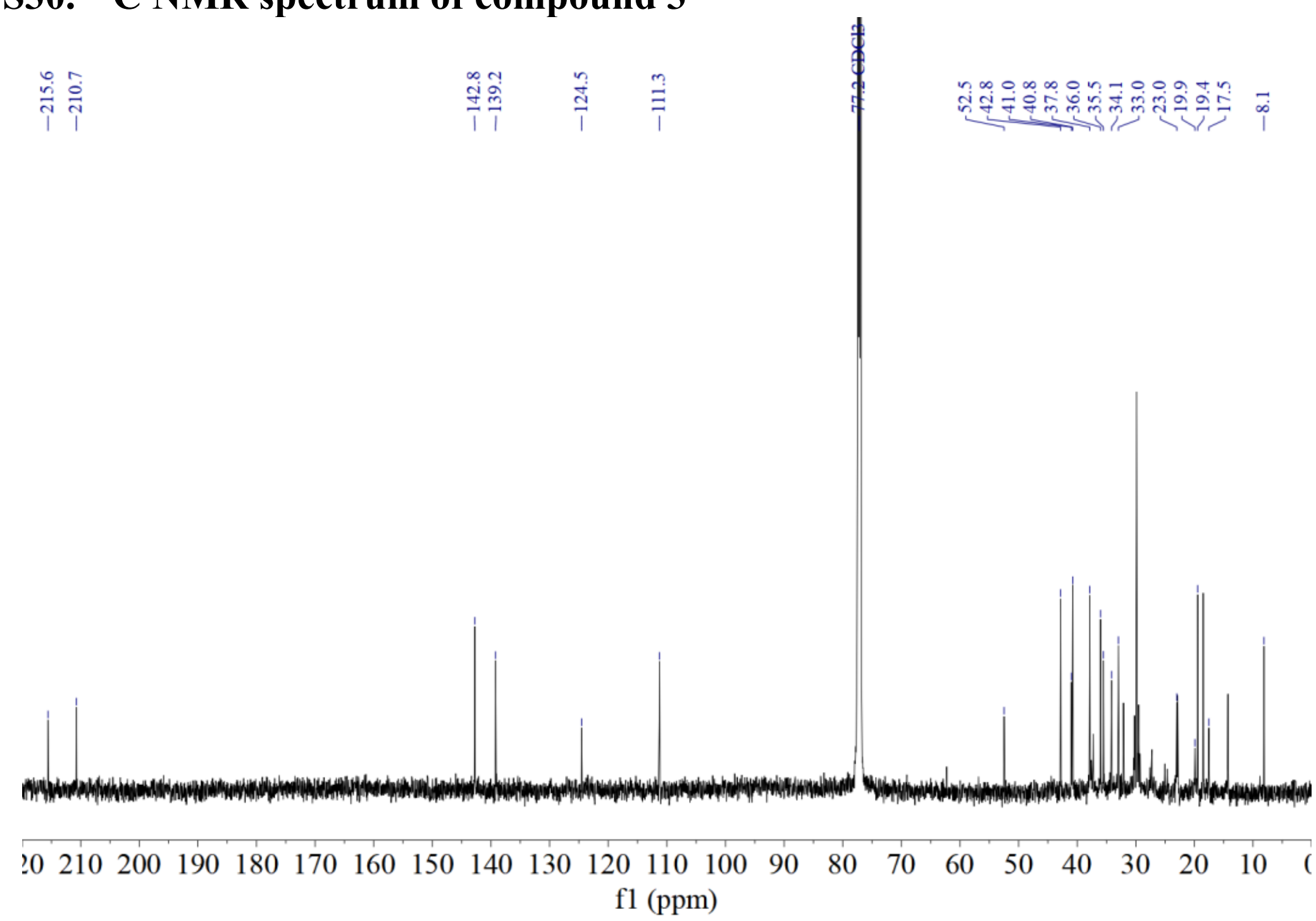

**Figure S31. DEPT NMR spectrum of compound 3**

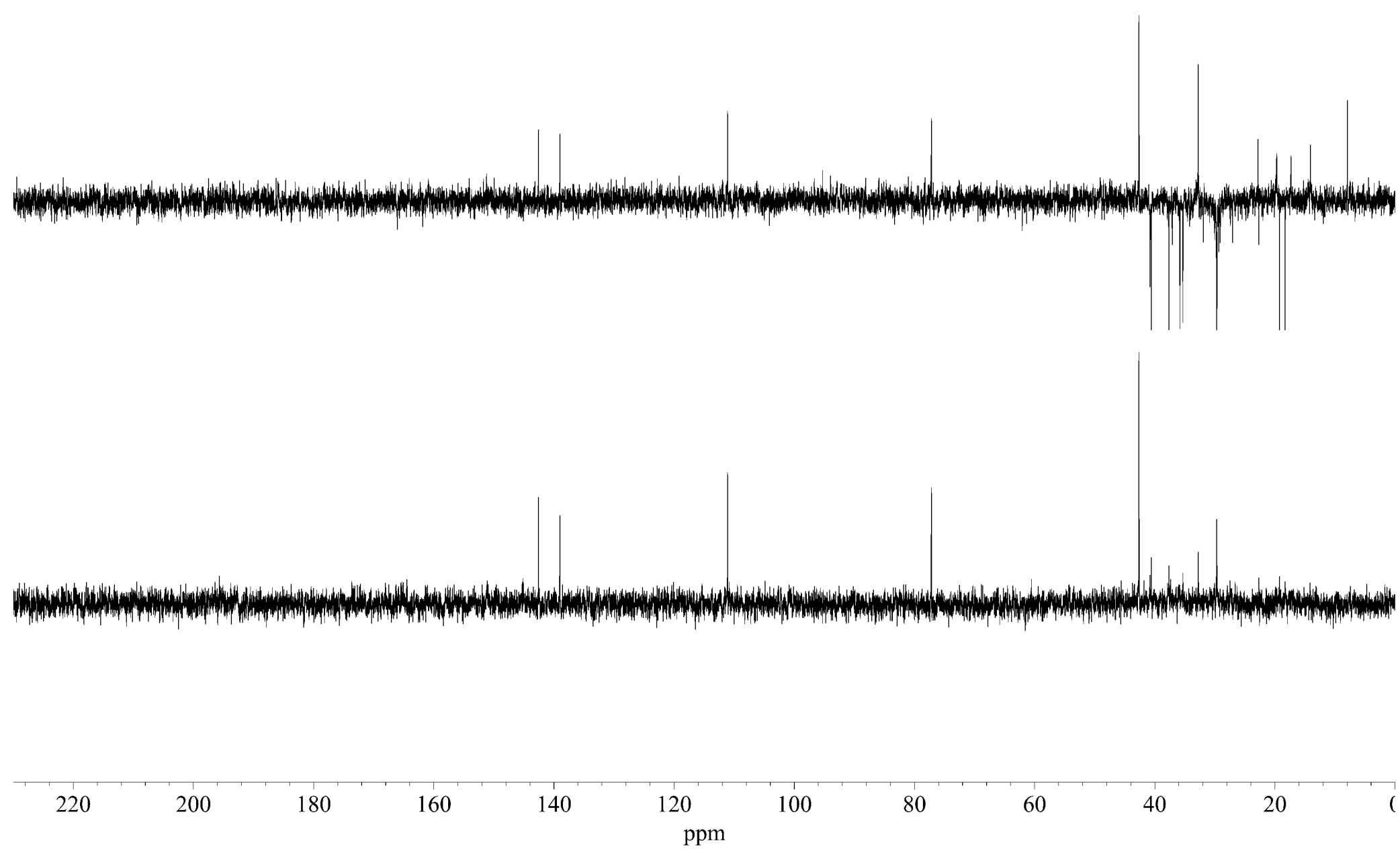


**Figure S32. COSY spectrum of compound 3**

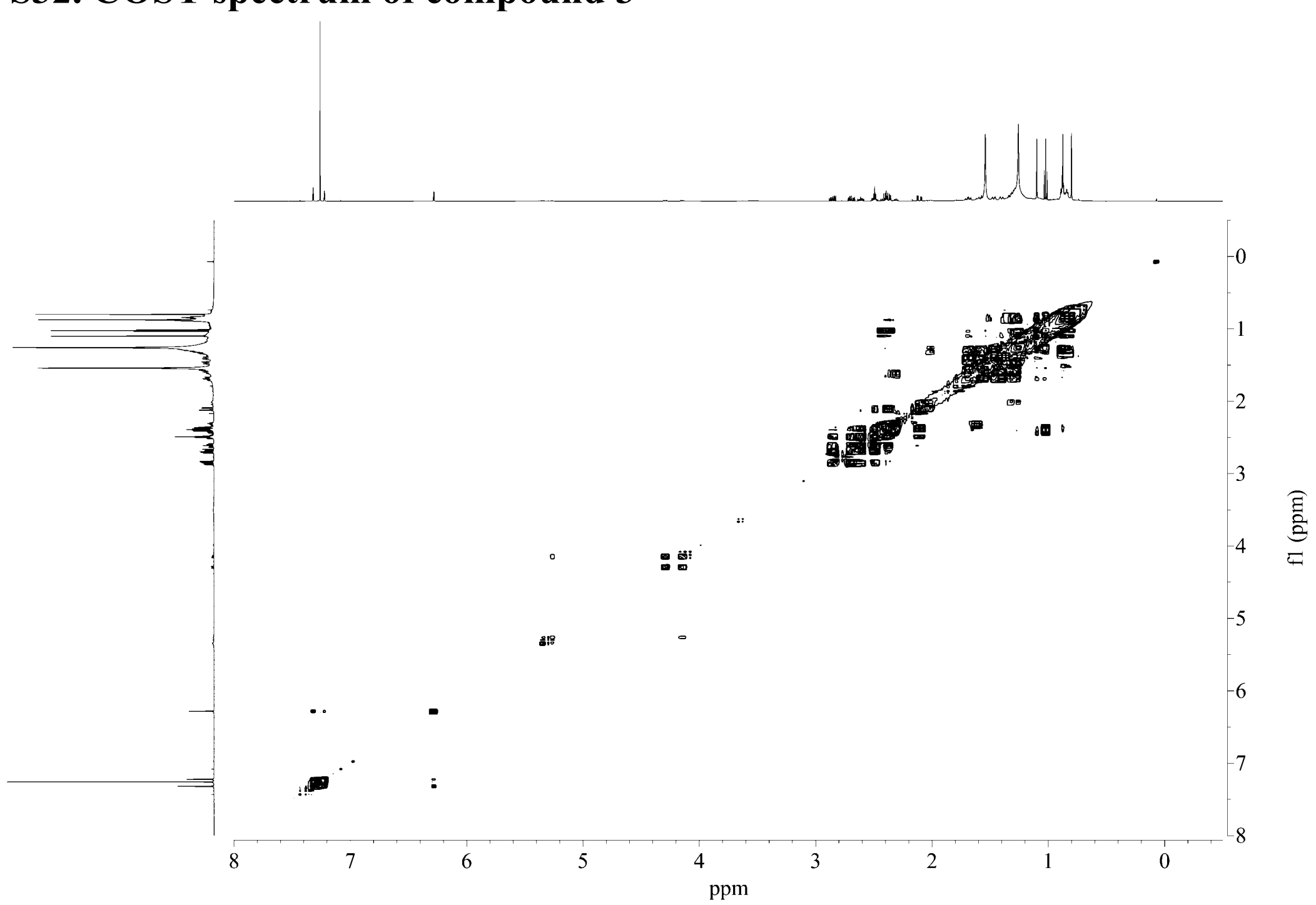

**Figure S33. HSQC spectrum of compound 3**

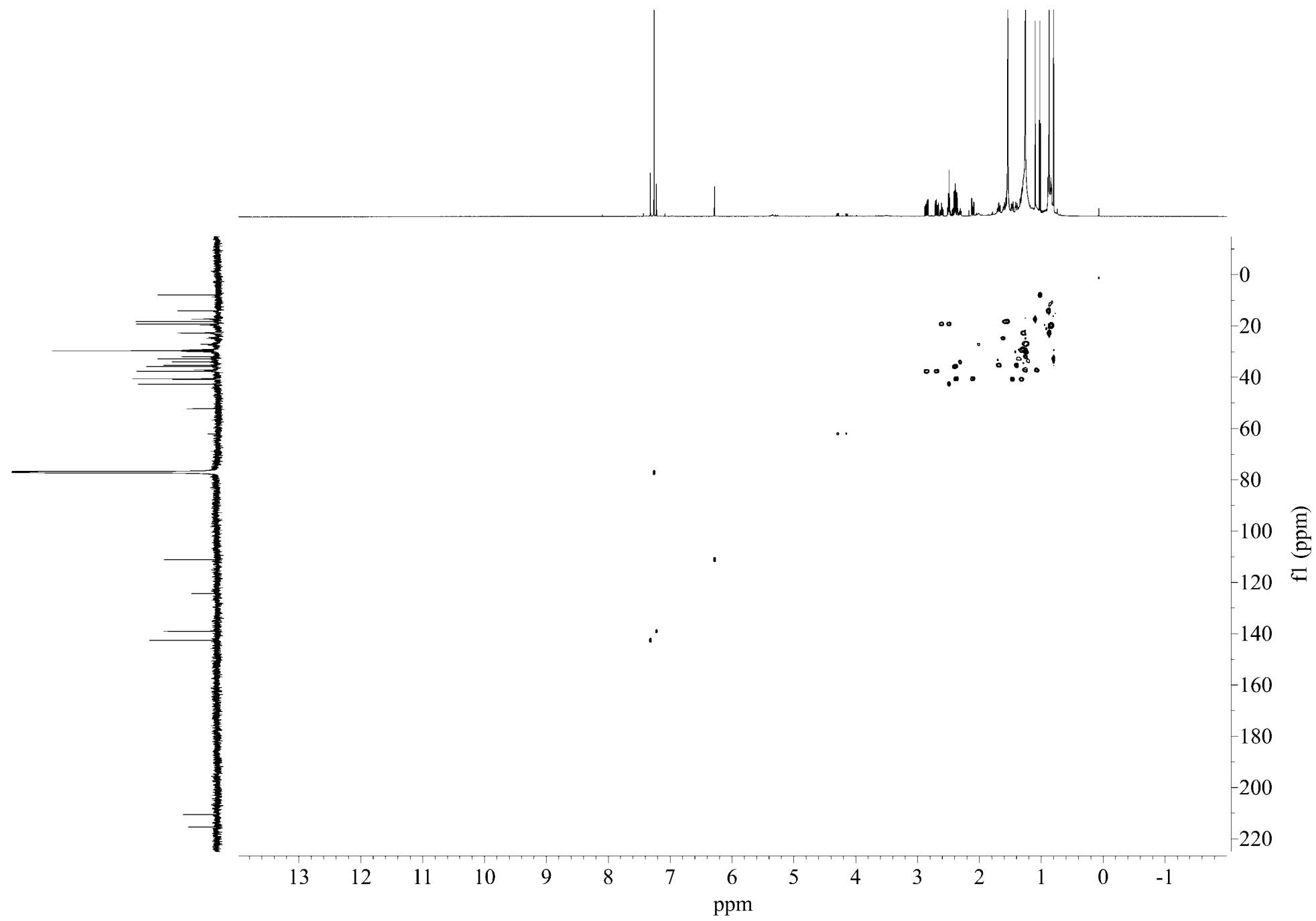


**Figure S34. HMBC spectrum of compound 3**

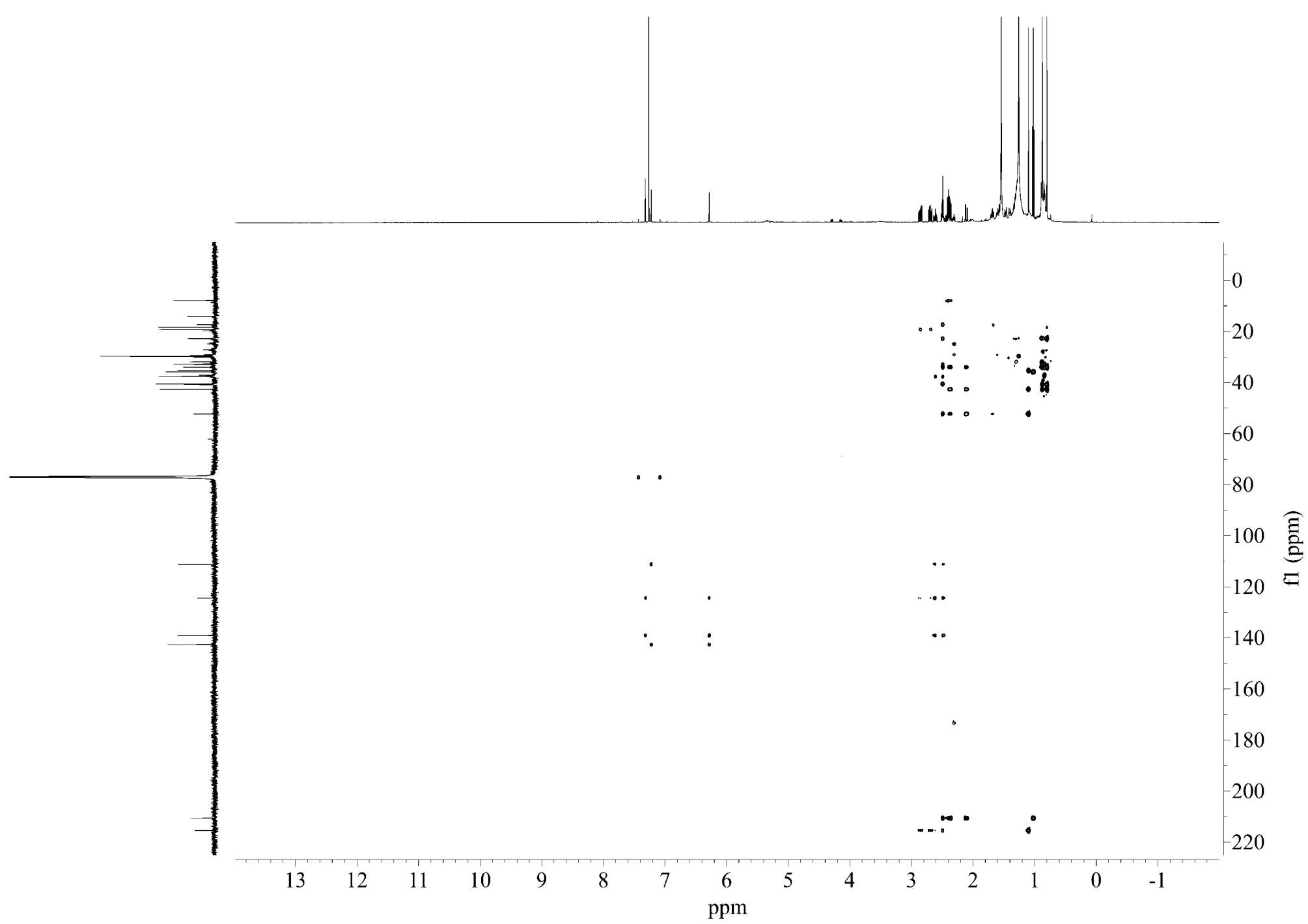

**Figure S35. NOESY spectrum of compound 3**

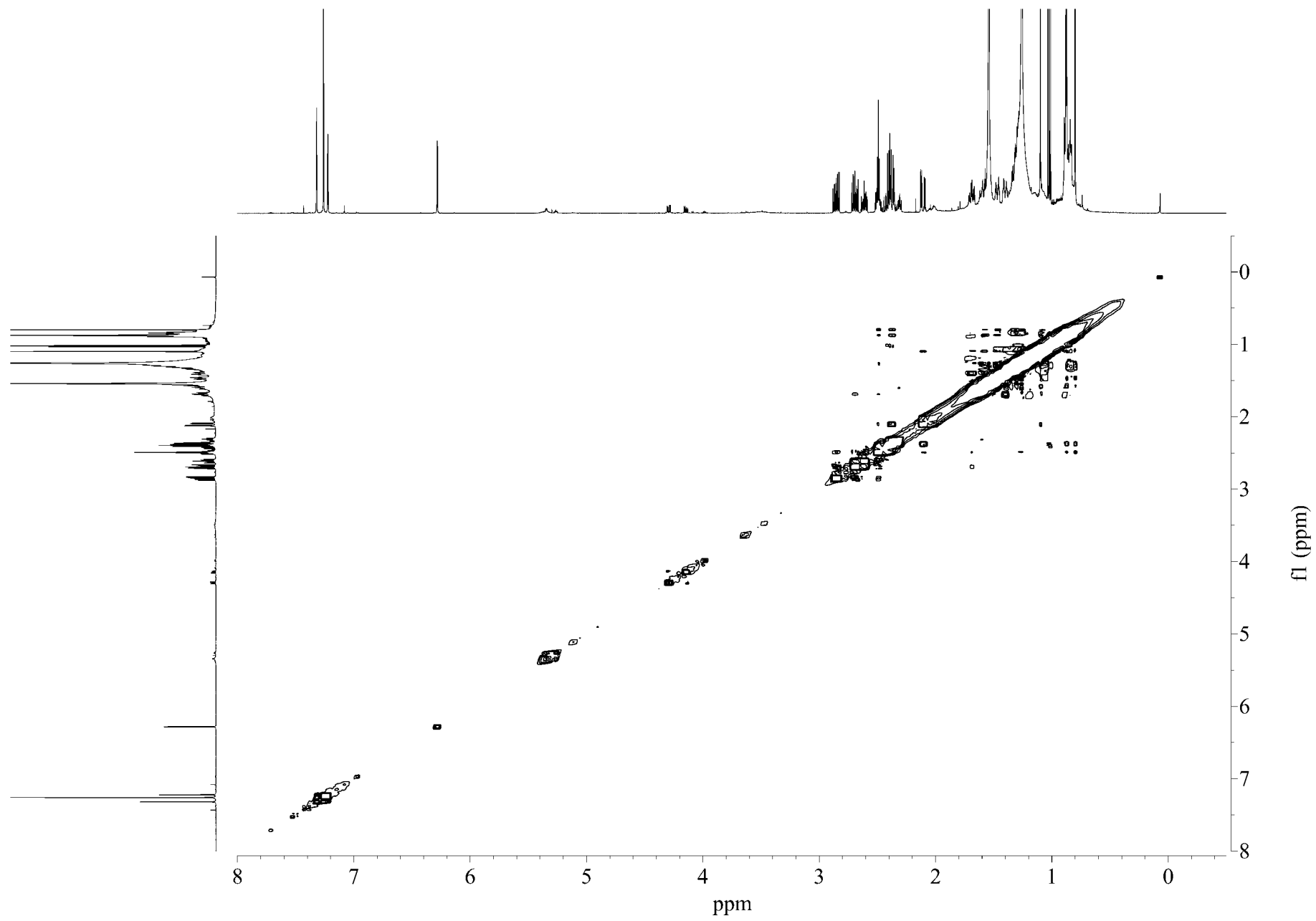


**Figure S36. ESI-MS spectrum of compound 3**

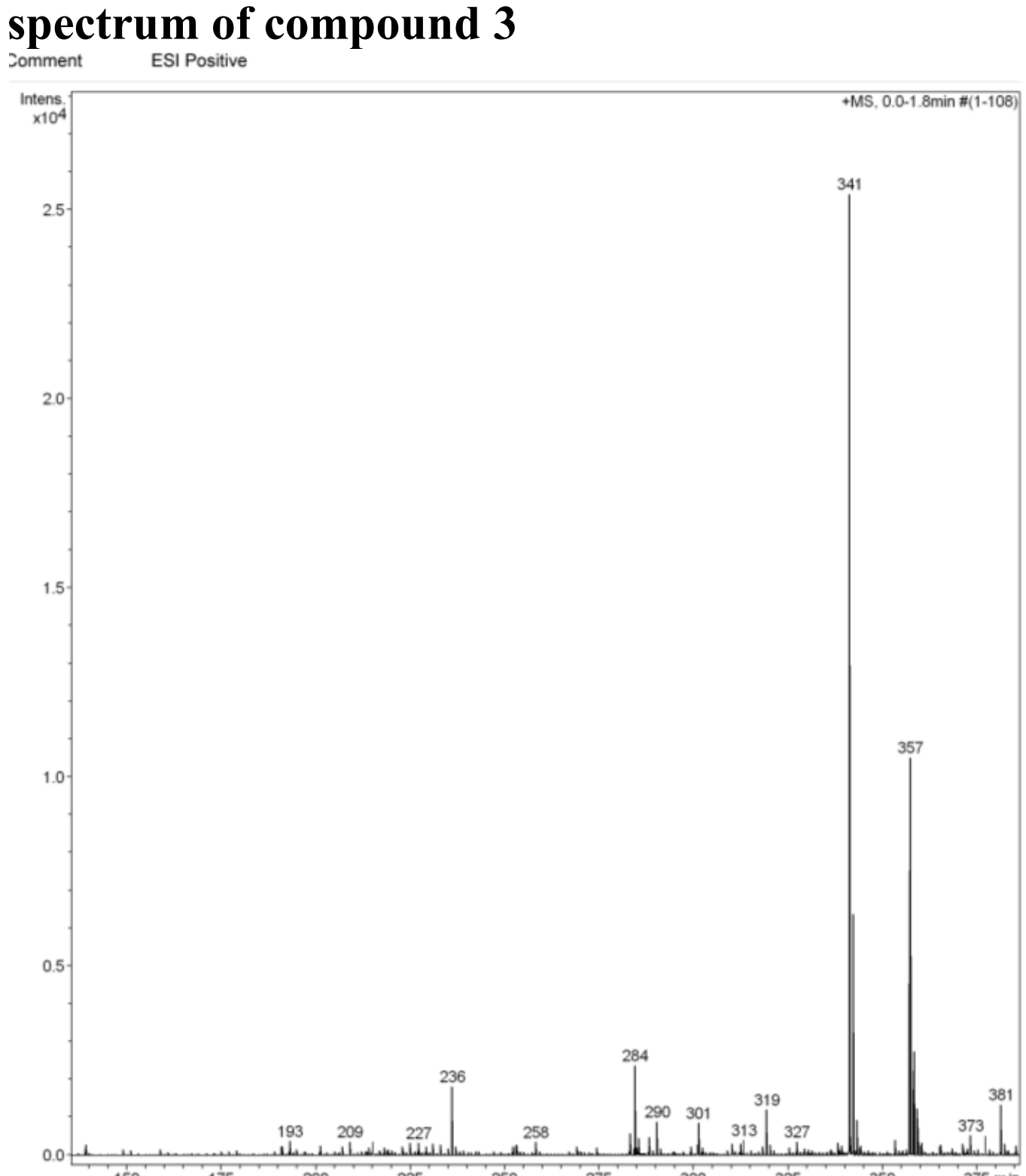

**Figure S37. UV and IR spectrum of compound 3**

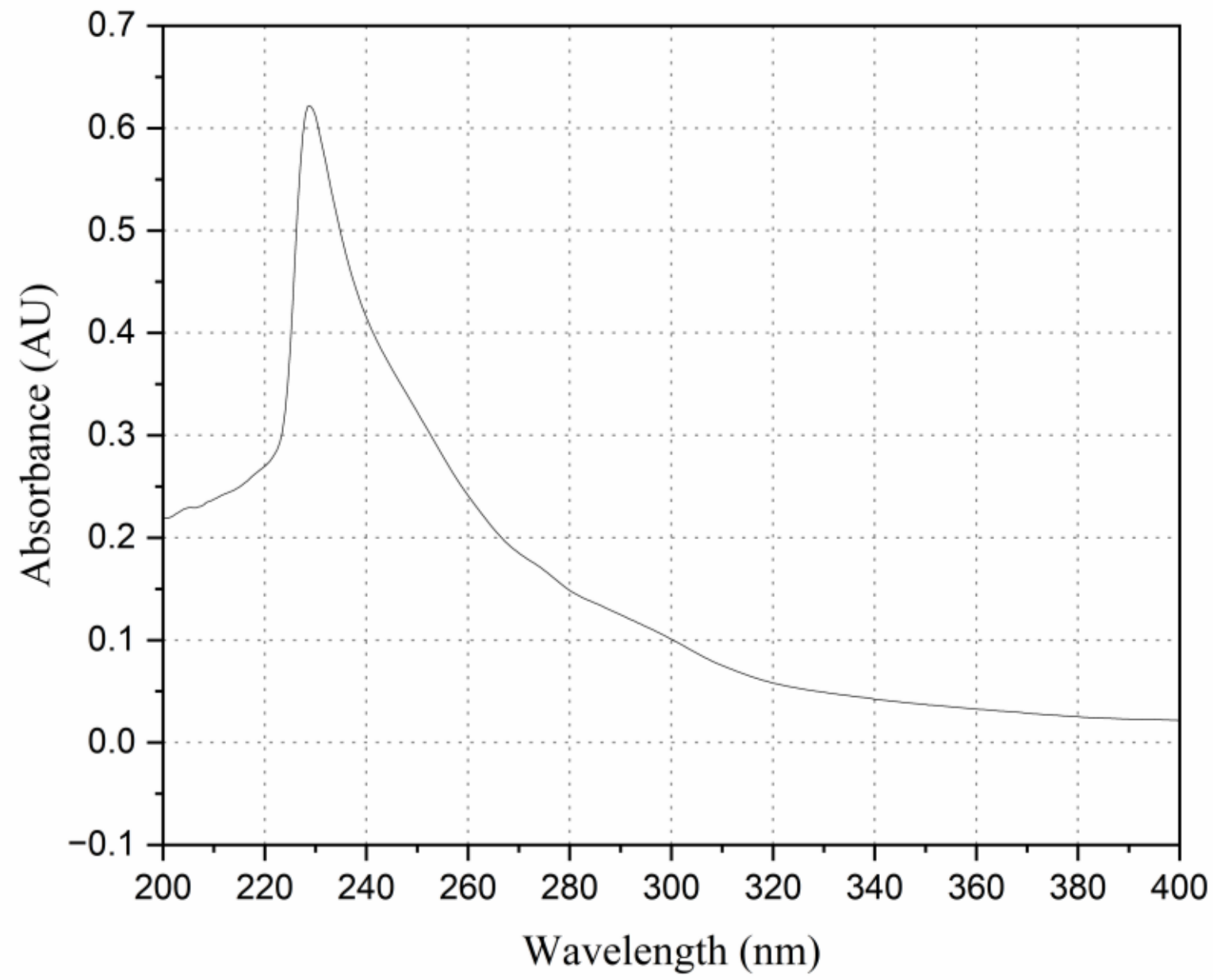


Compound **3**

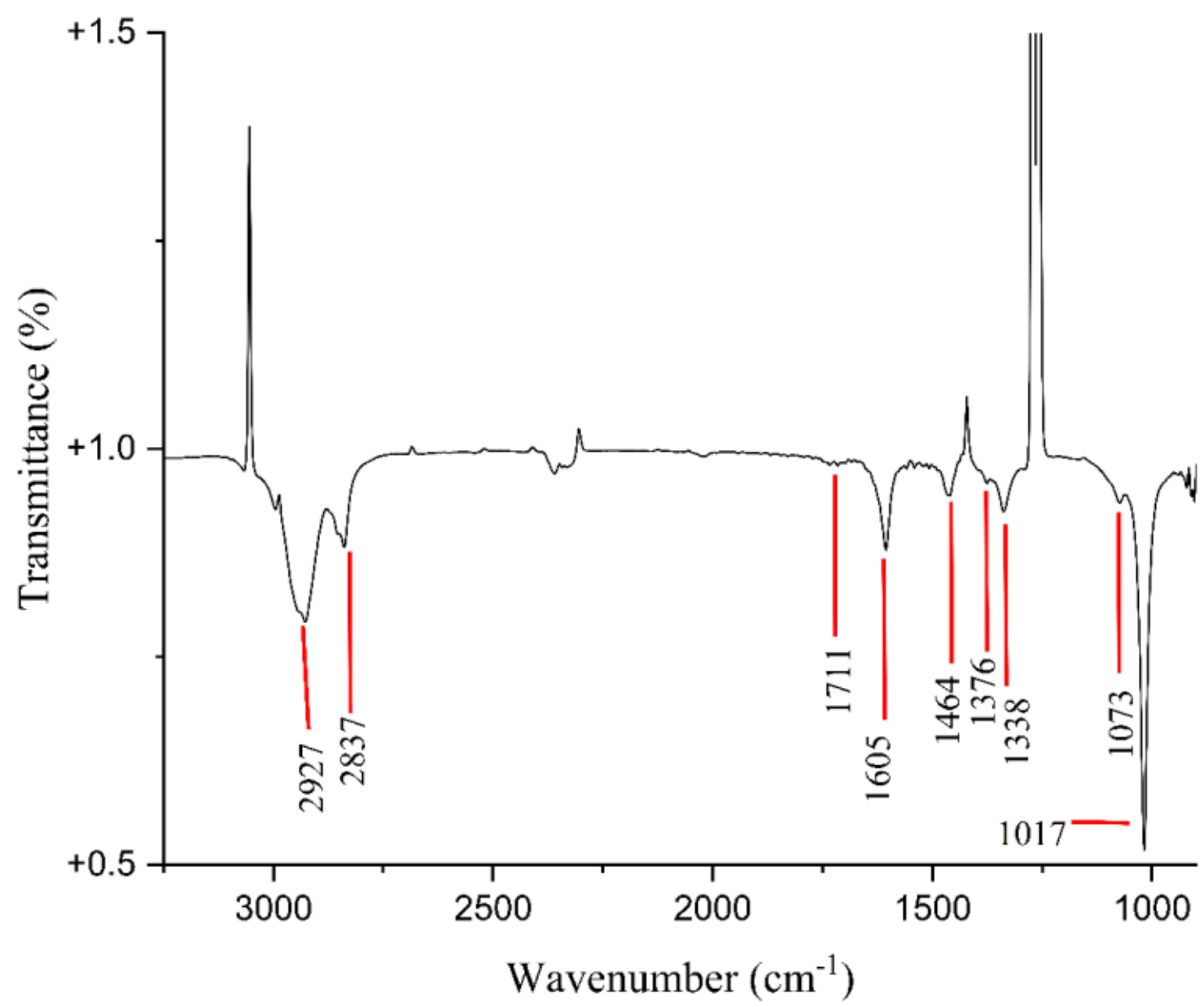


IR ($CH_2Cl_2$) $\nu_{max}$ 2927, 2837, 1711, 1605, 1464, 1376, 1338, 1073, 1017 cm$^{-1}$.

**Figure S38. $^1$H NMR spectrum of compound 4**

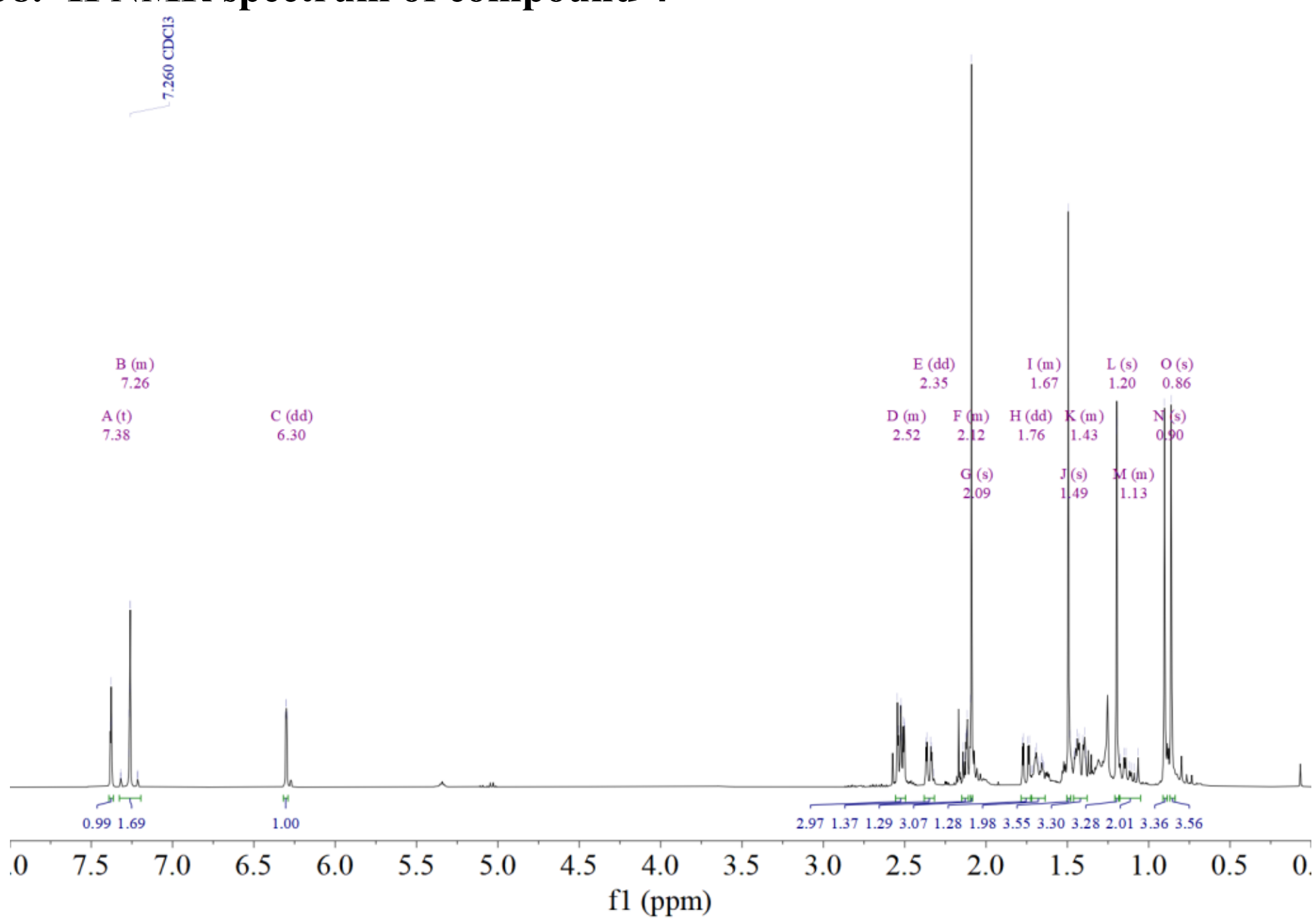


**Figure S39. $^{13}$C NMR spectrum of compound 4**

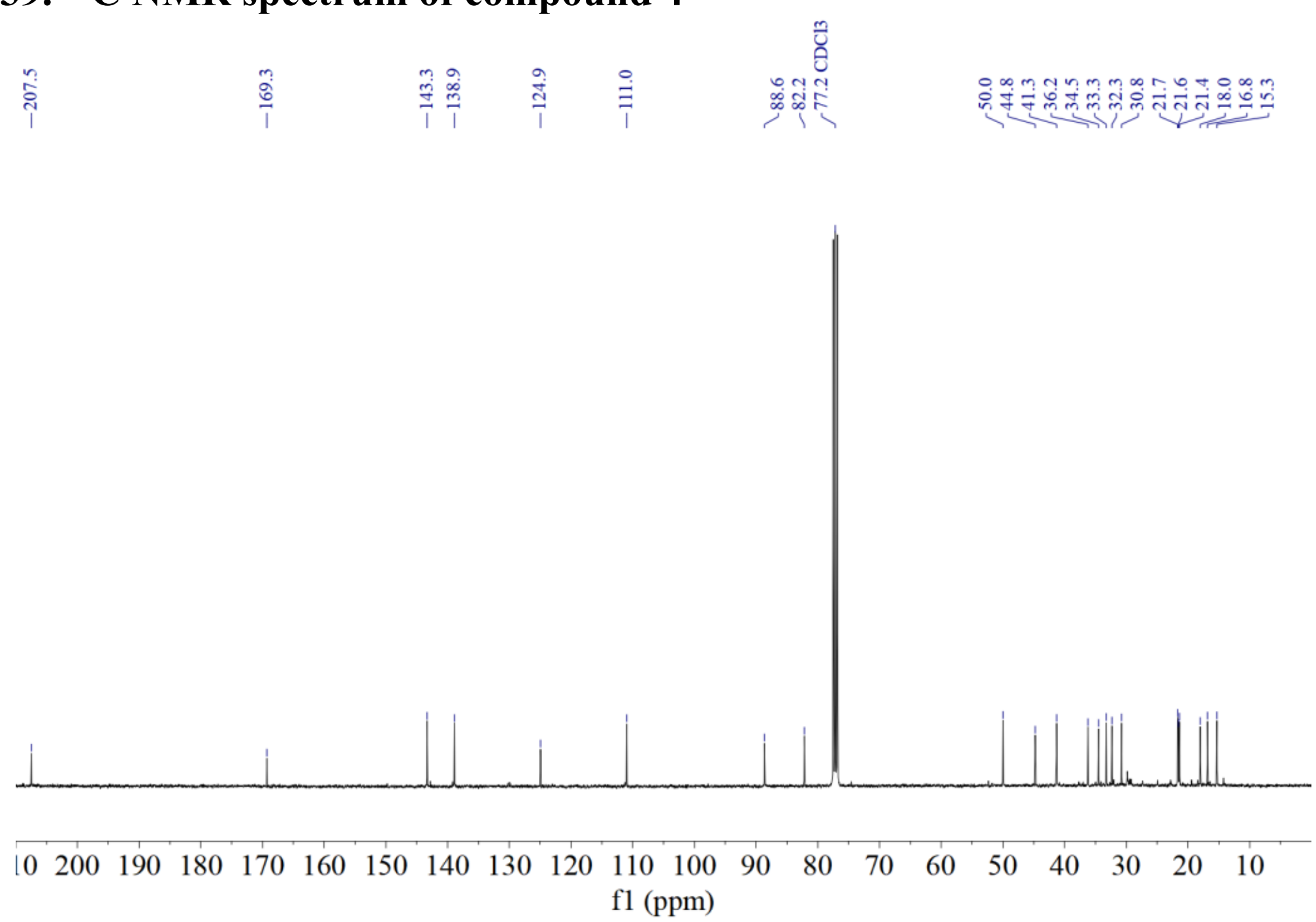

**Figure S40. DEPT NMR spectrum of compound 4**

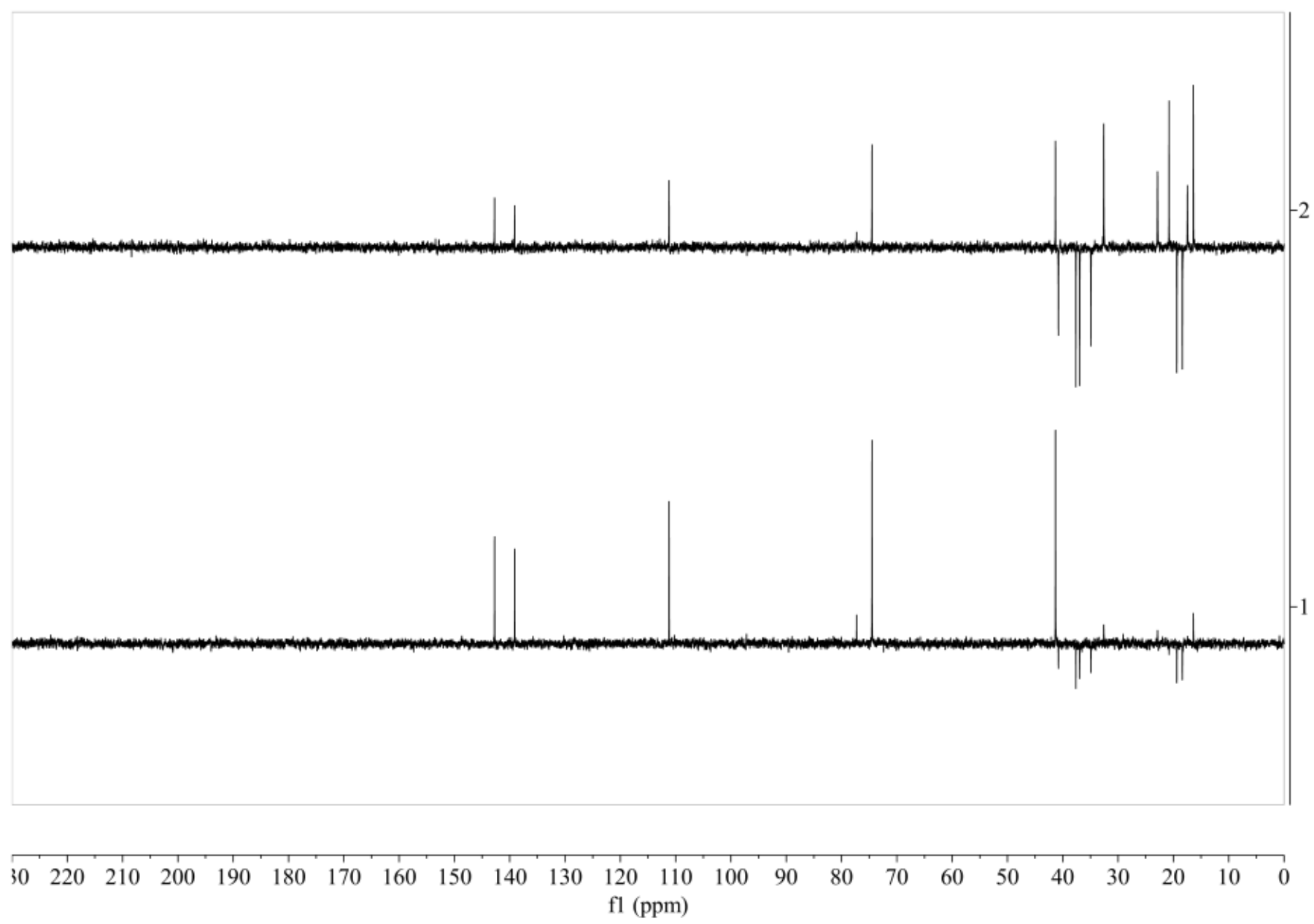


**Figure S41. COSY spectrum of compound 4**

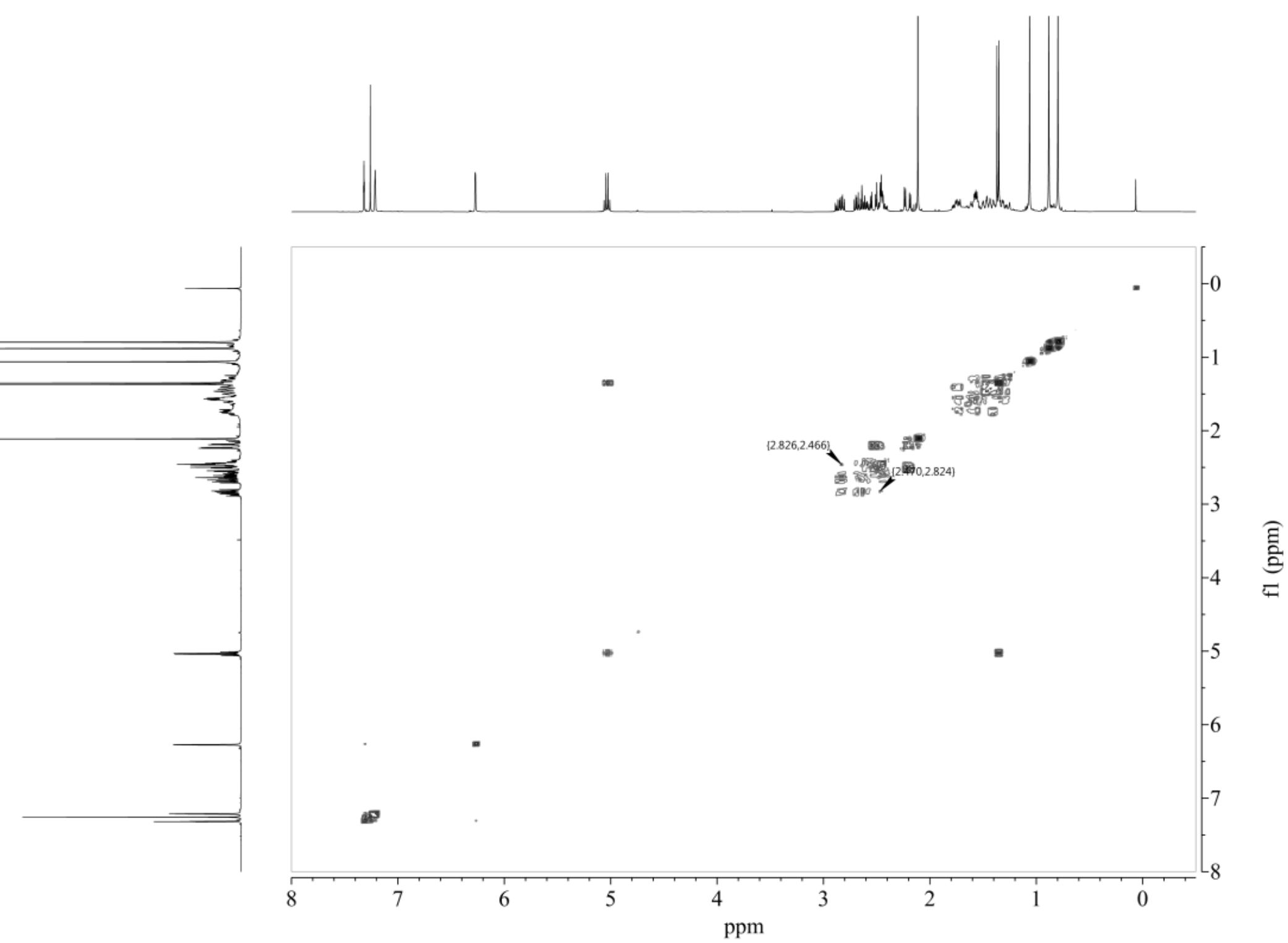

**Figure S42. HSQC spectrum of compound 4**

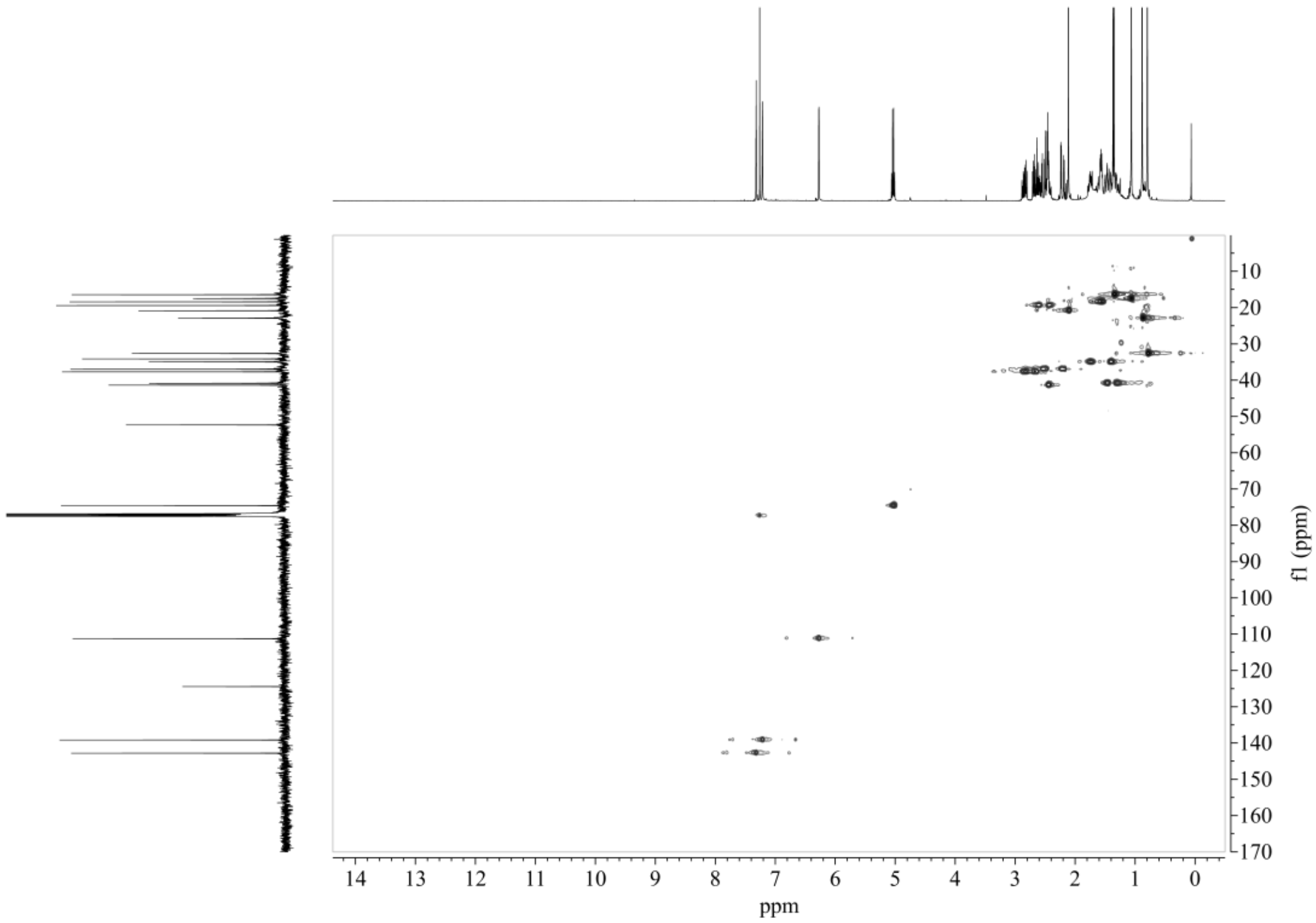


**Figure S43. HMBC spectrum of compound 4**

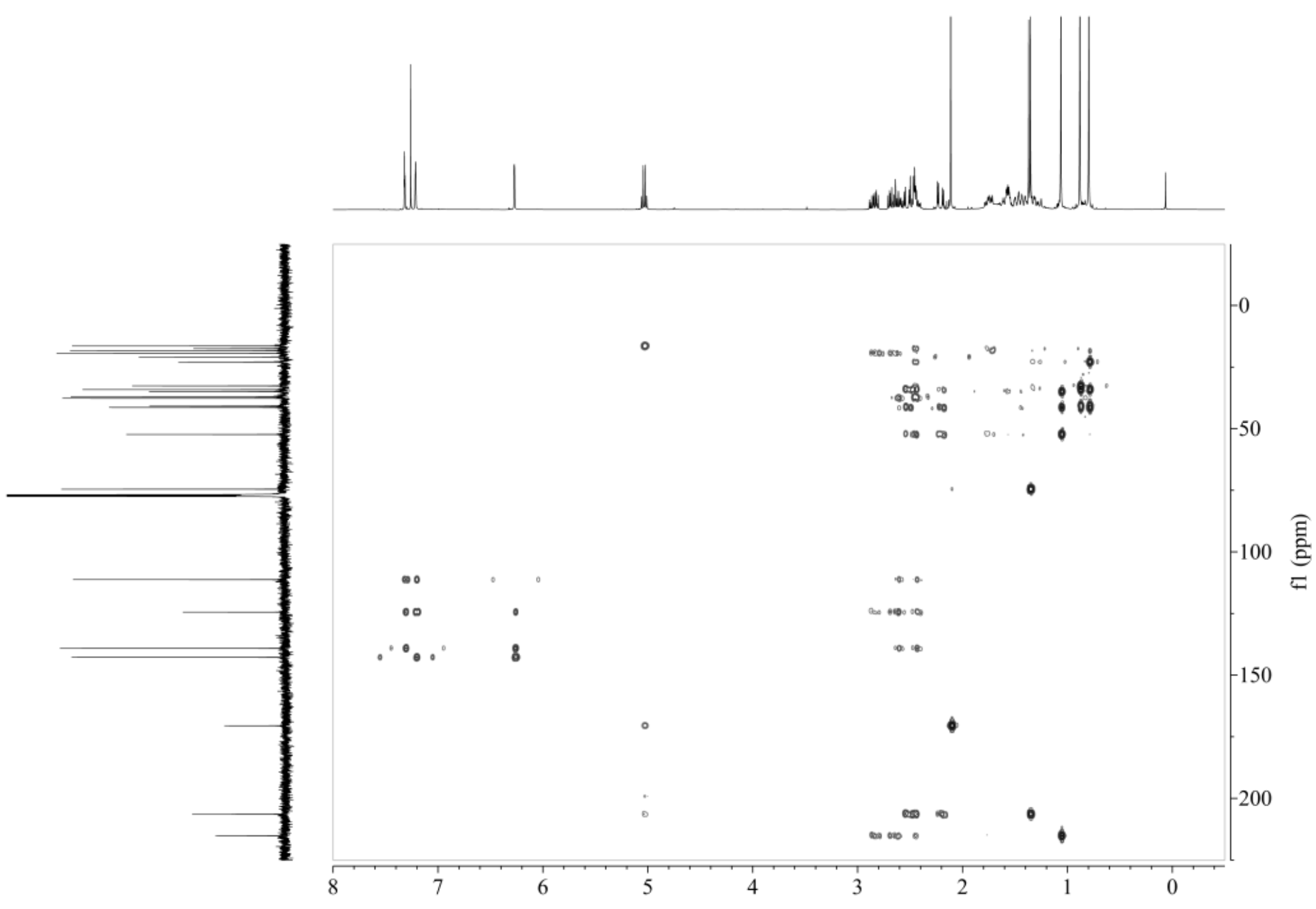

**Figure S44. ROESY spectrum of compound 4**

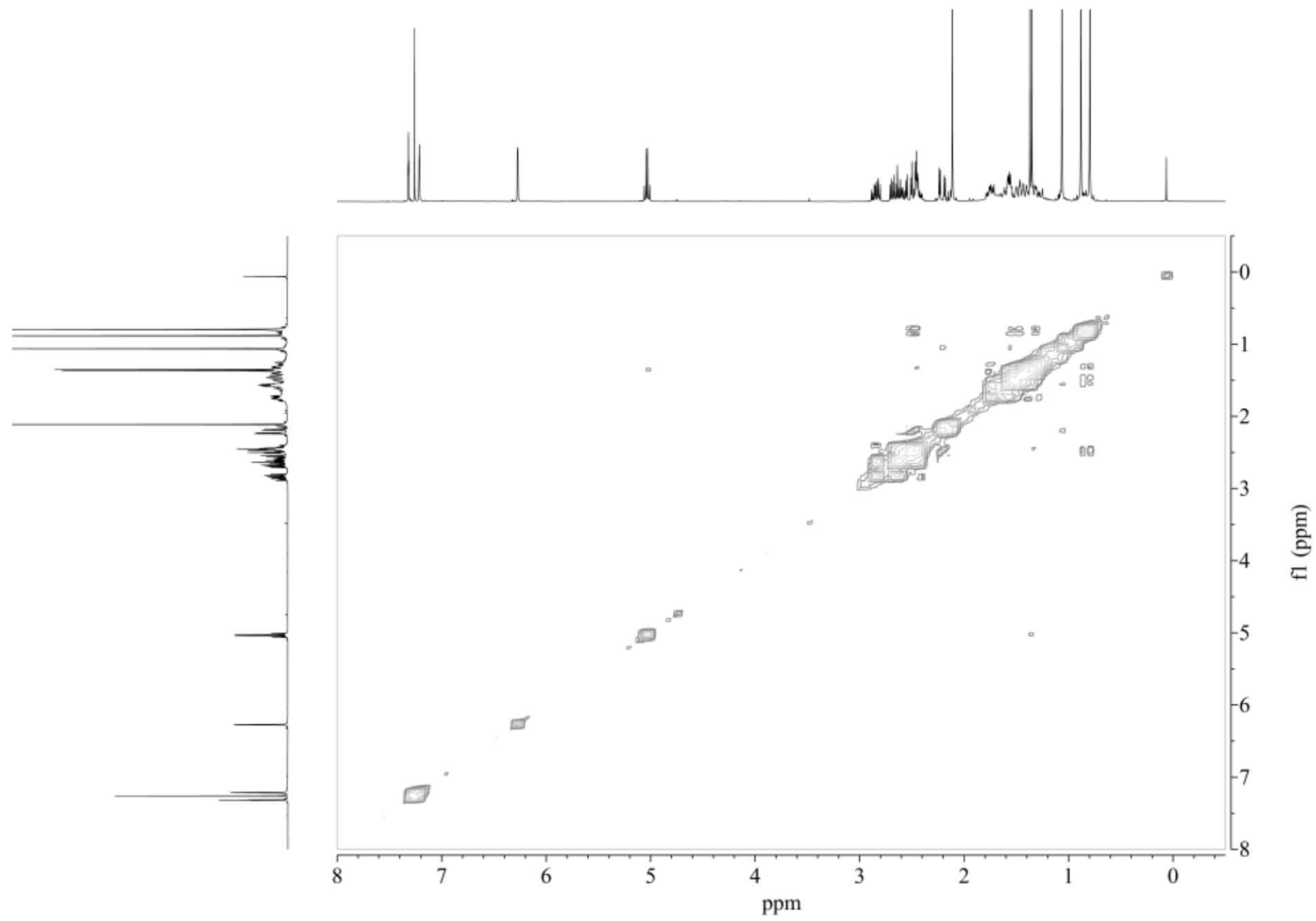


**Figure S45. ESI-MS spectrum of compound 4**

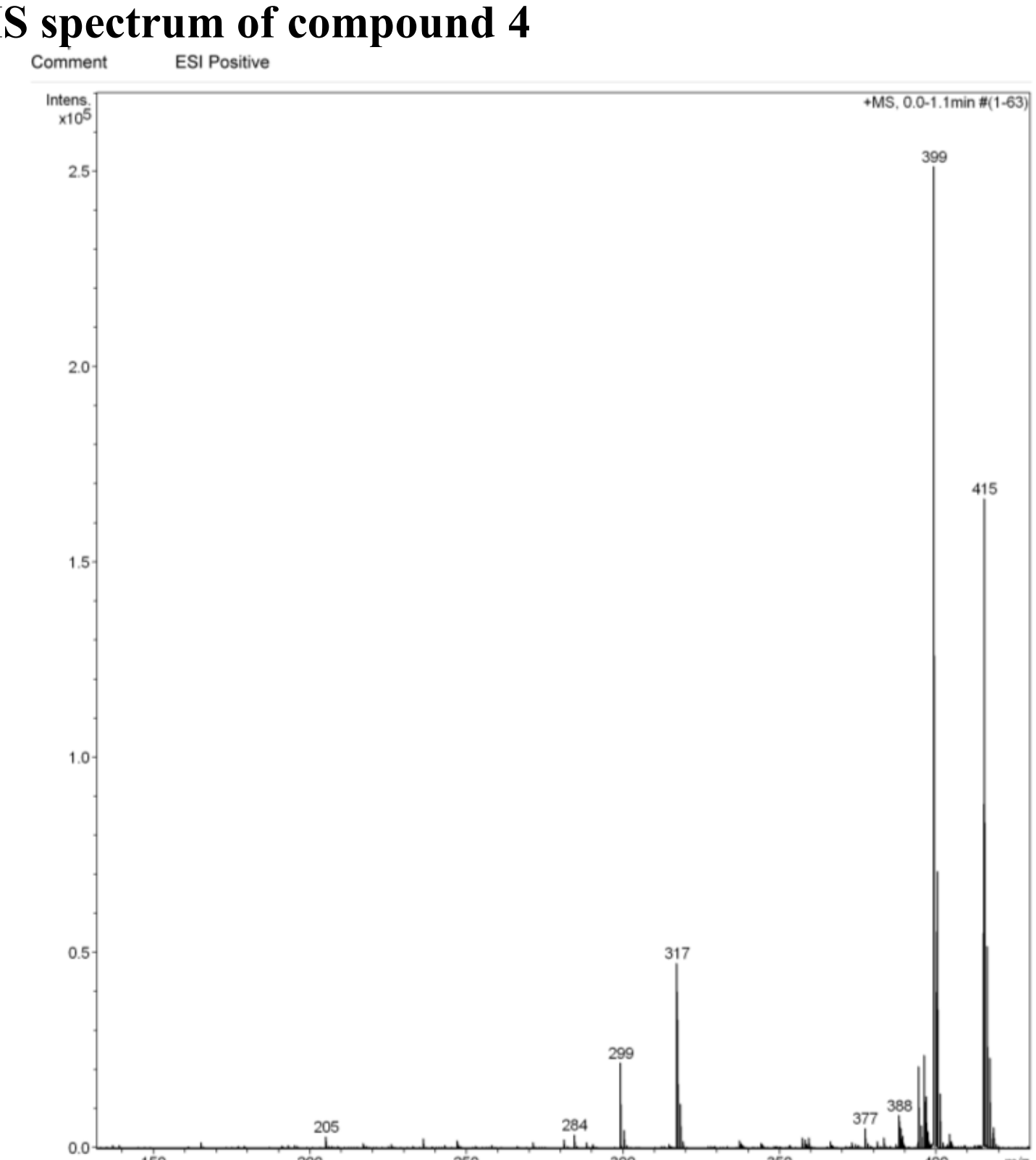

**Figure S46. UV and IR spectrum of compound 4**

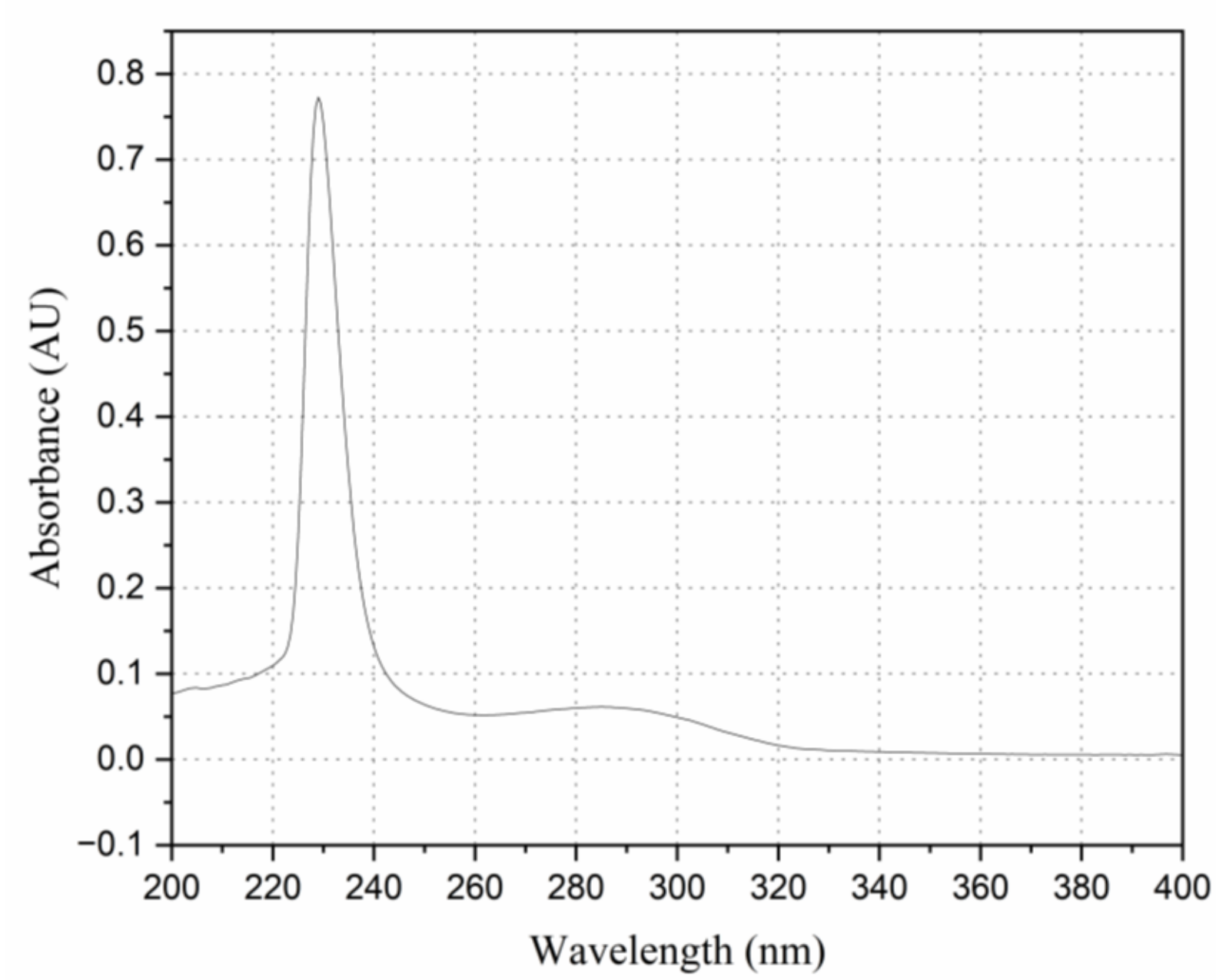


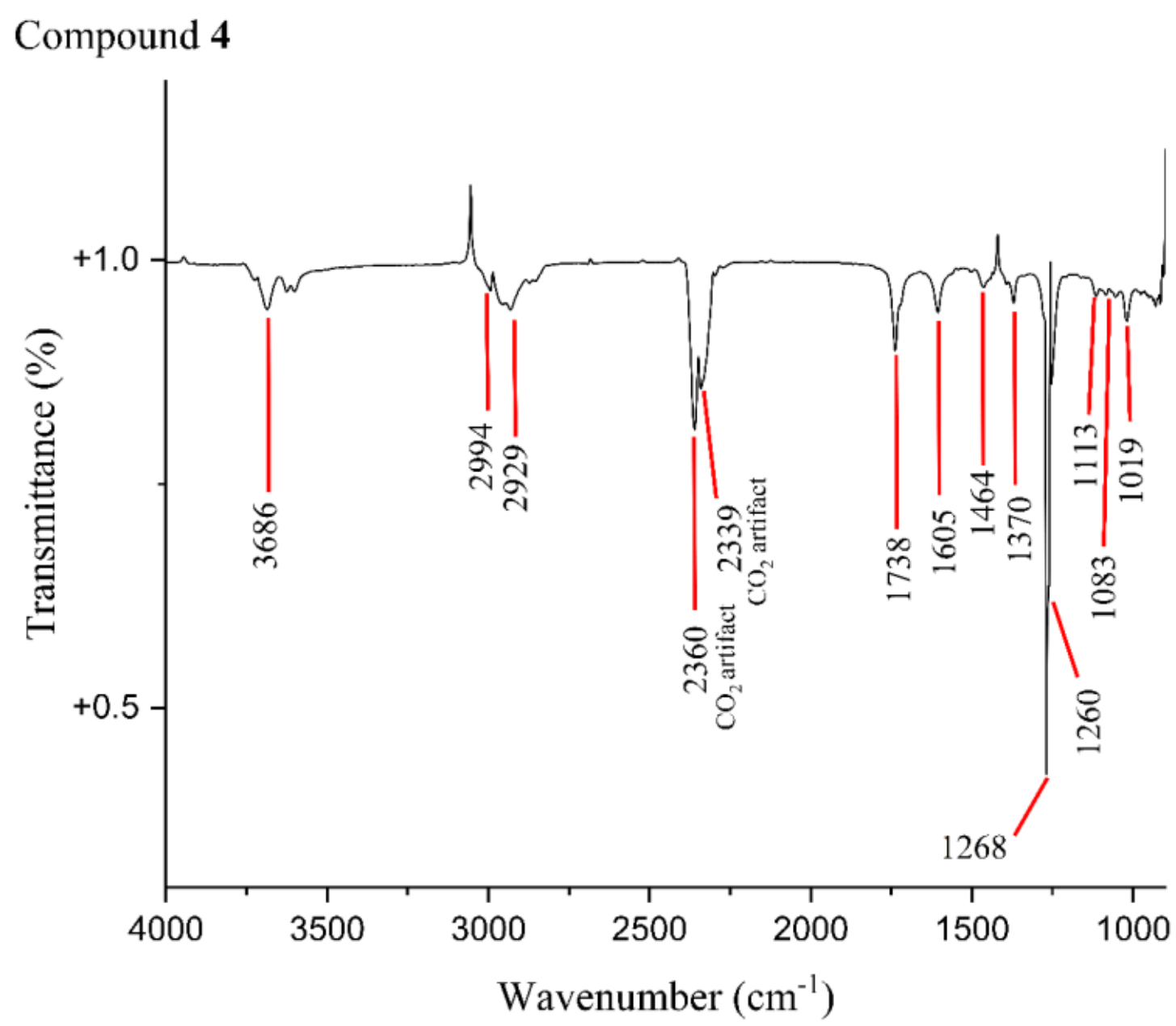


IR ($CH_2Cl_2$) $\nu_{max}$ 3686, 2994, 2929, 1738, 1605, 1464, 1370, 1268, 1260, 1113, 1083, 1019 cm$^{-1}$.

**Figure S47. $^{1}$H NMR spectrum of compound 5**

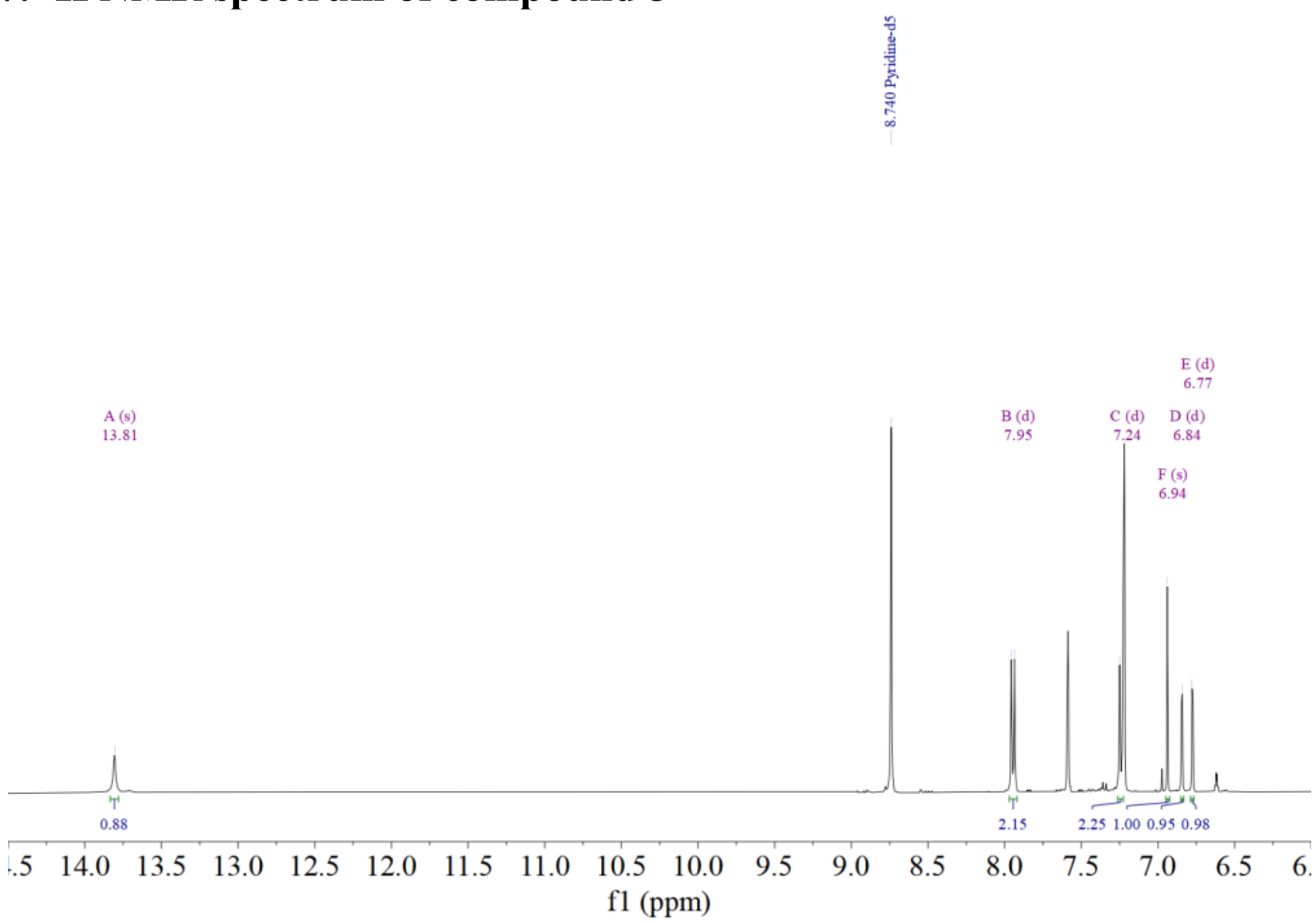


**Figure S48. $^{13}$C NMR spectrum of compound 5**

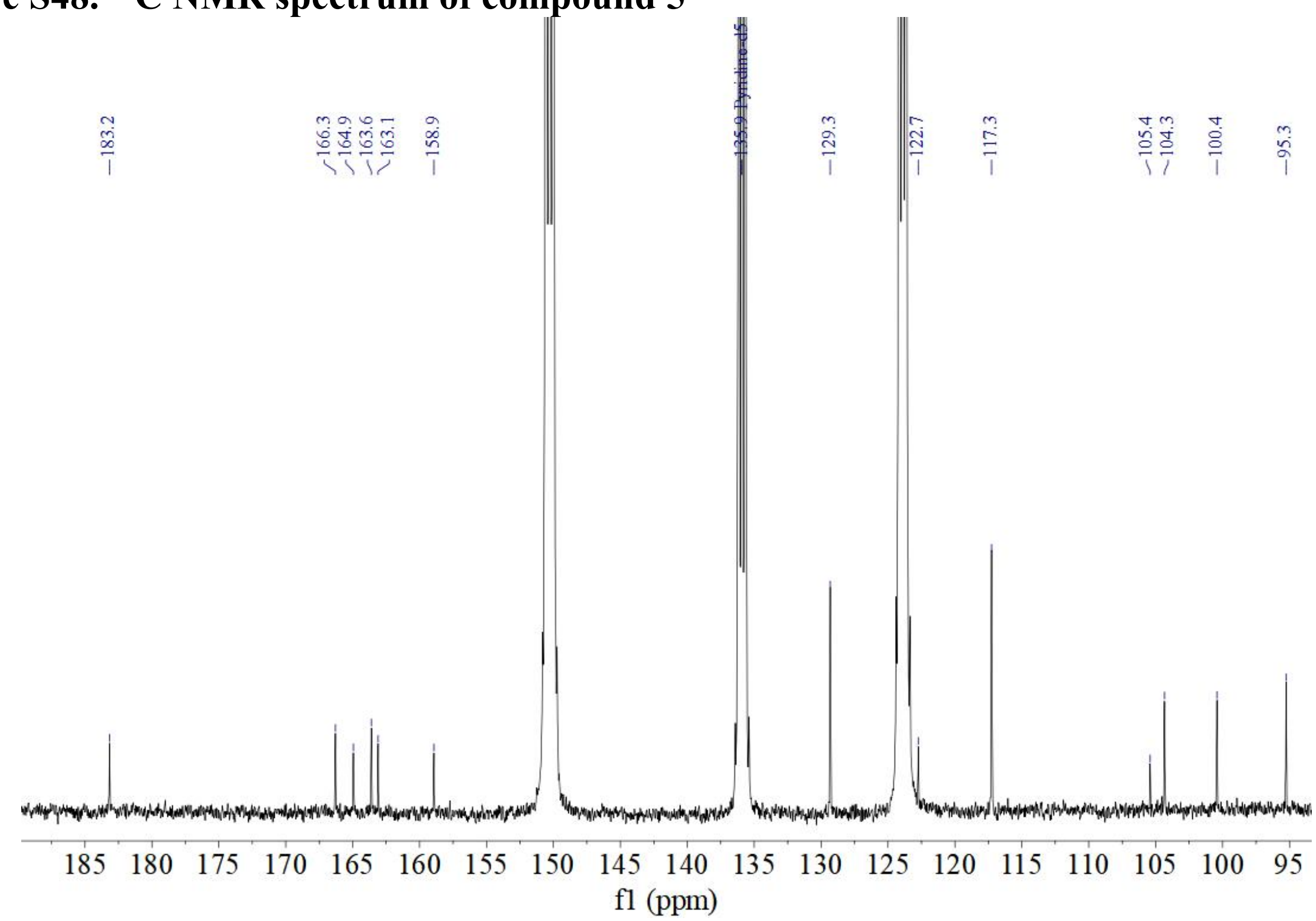

**Figure S49. DEPT NMR spectrum of compound 5**

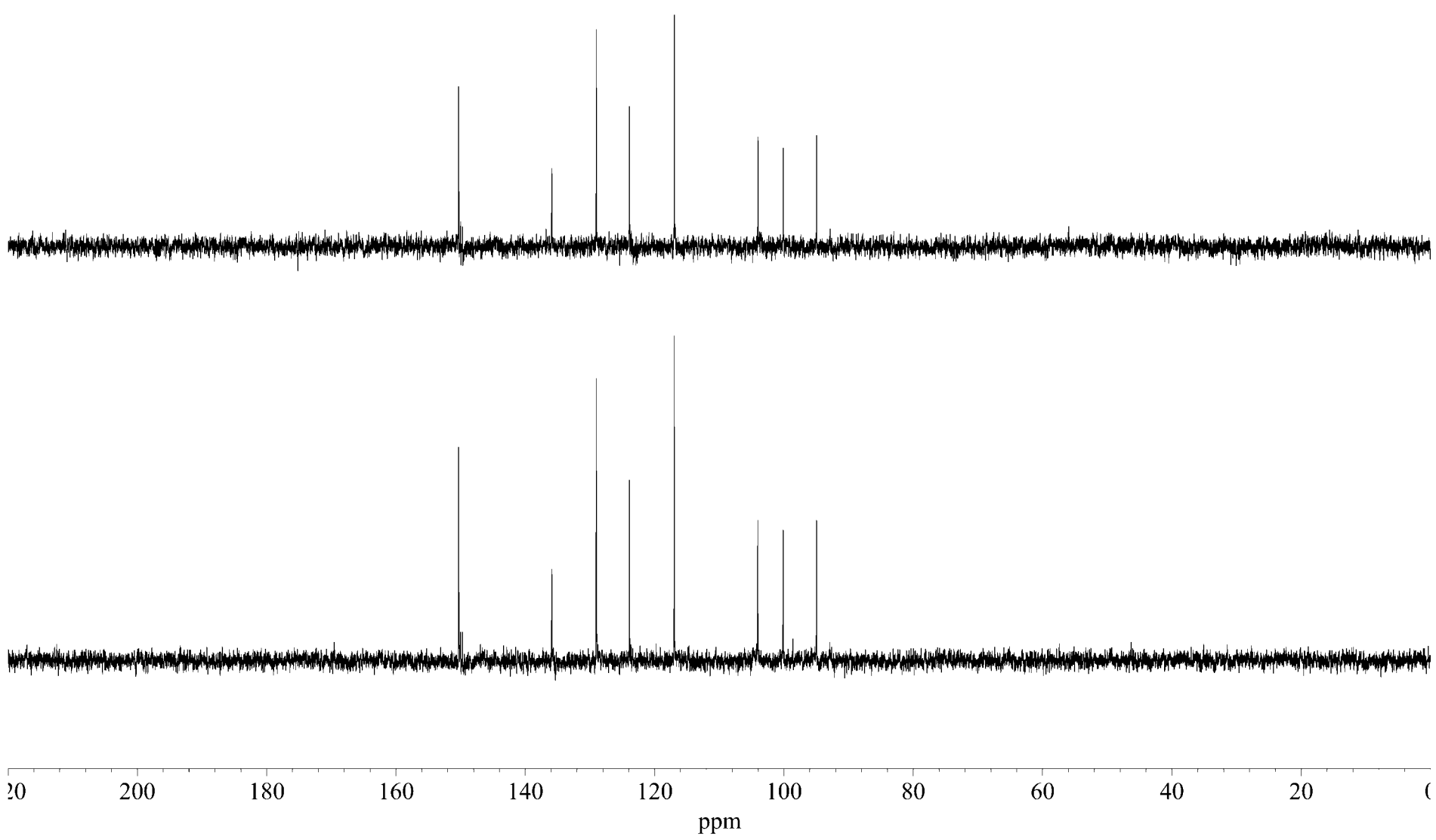


**Figure S50. ESI-MS spectrum of compound 5**

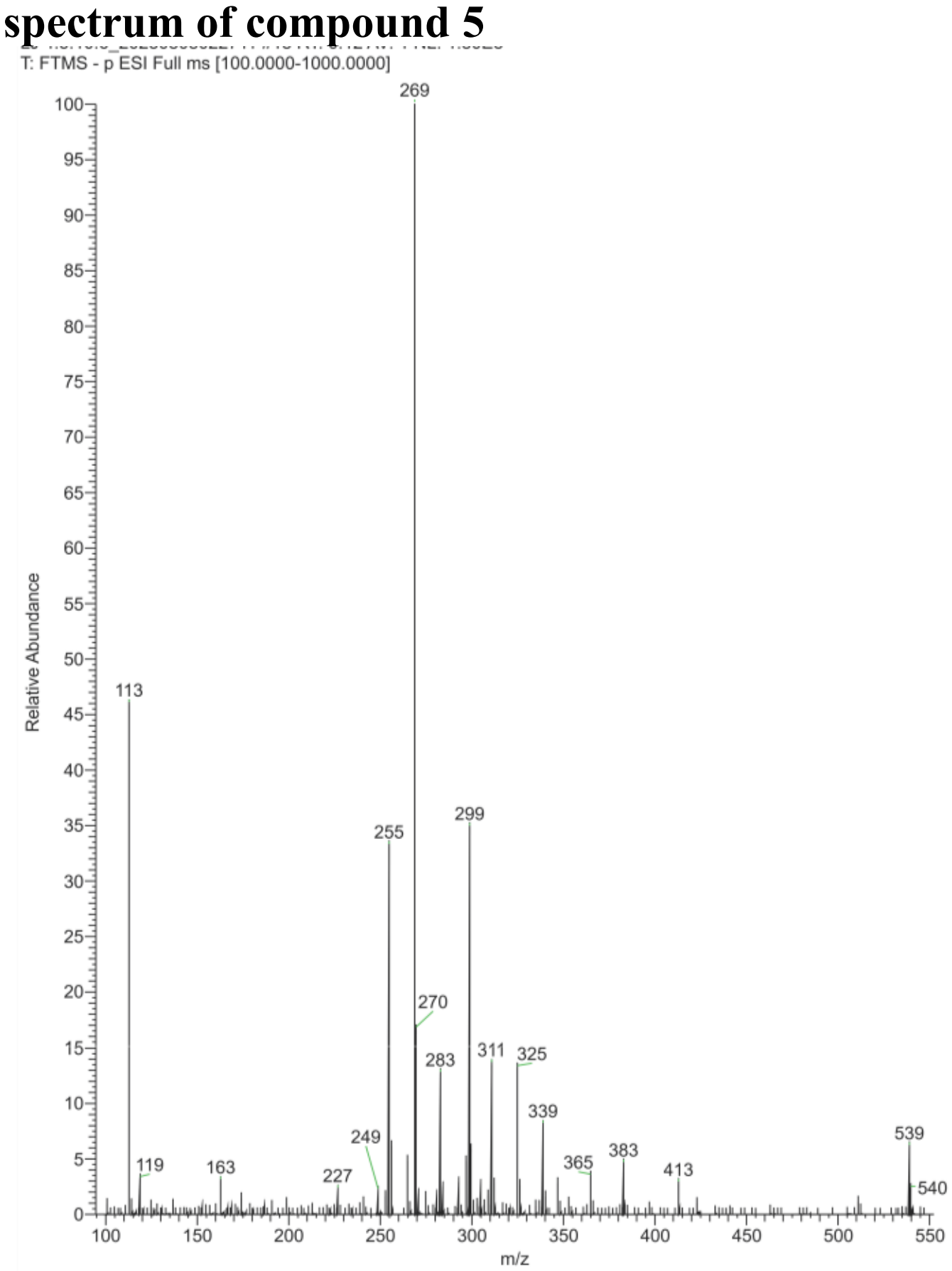

**Figure S51. UV and IR spectrum of compound 5**

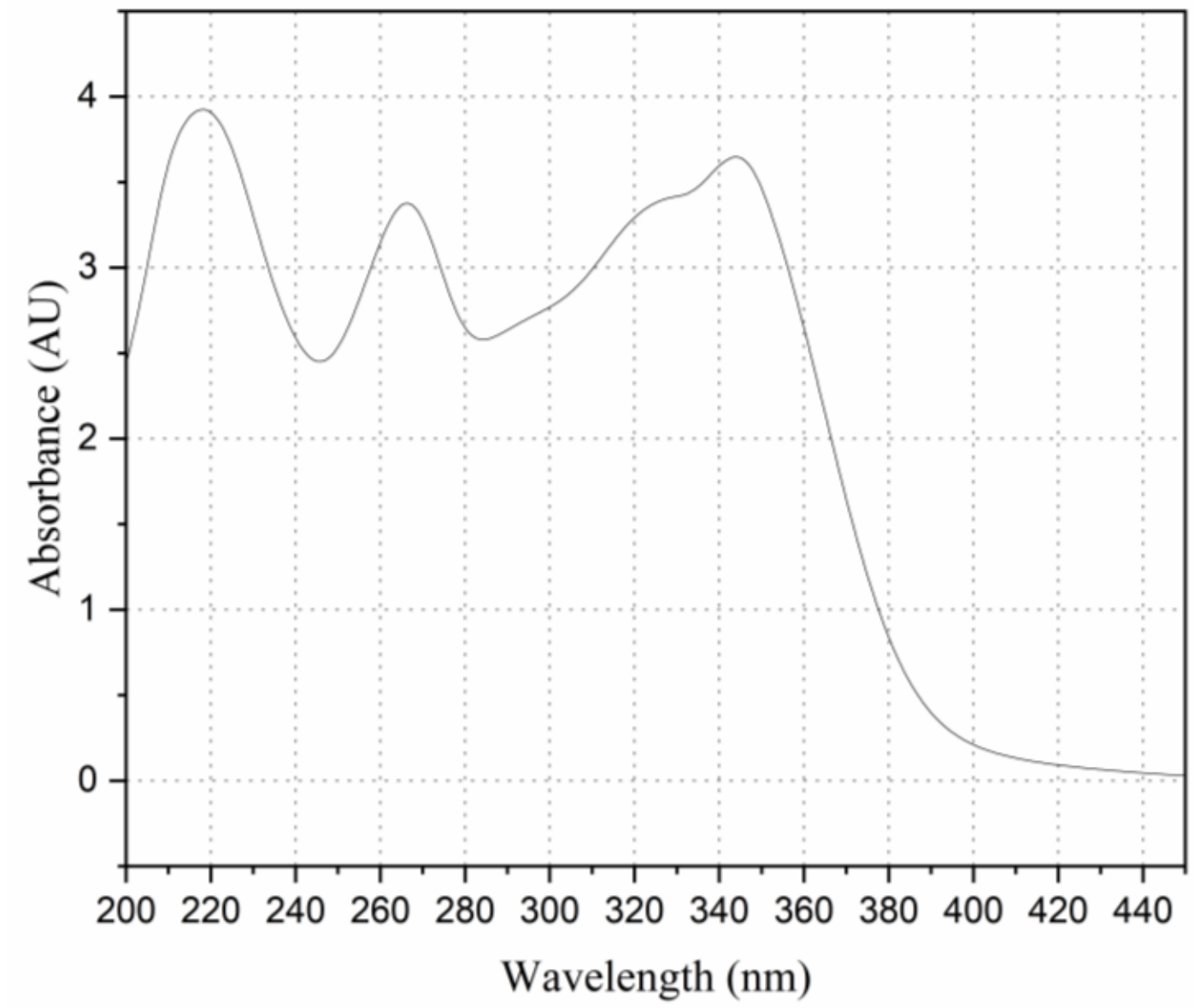


Compound **5**

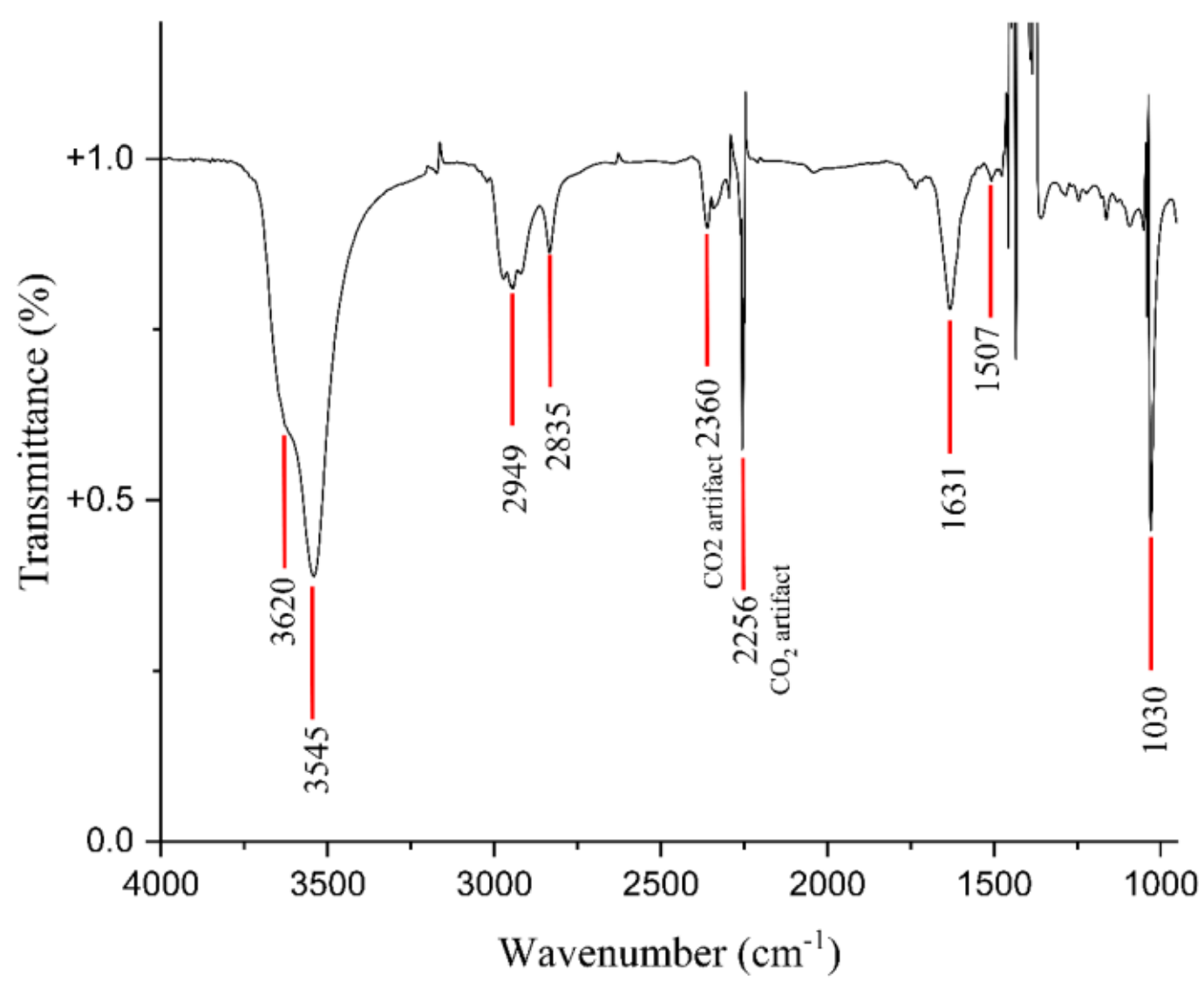


IR ($CH_3CN$) $\nu_{max}$ 3620, 3545, 2949, 2835, 1631, 1507, 1030, 1040, 917 cm$^{-1}$.

**Figure S52. $^1$H NMR spectrum of compound 6**

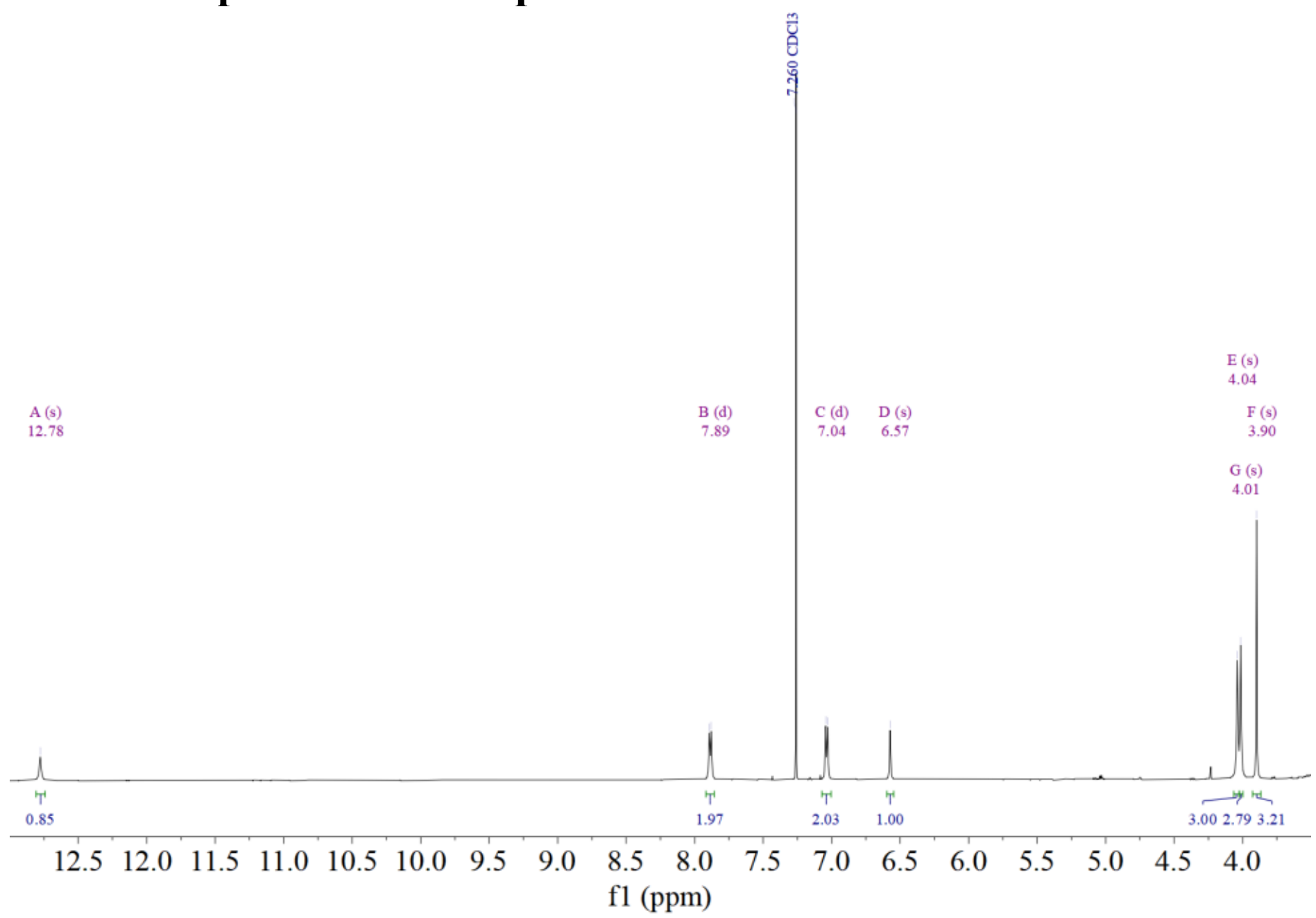


**Figure S53. $^{13}$C NMR spectrum of compound 6**

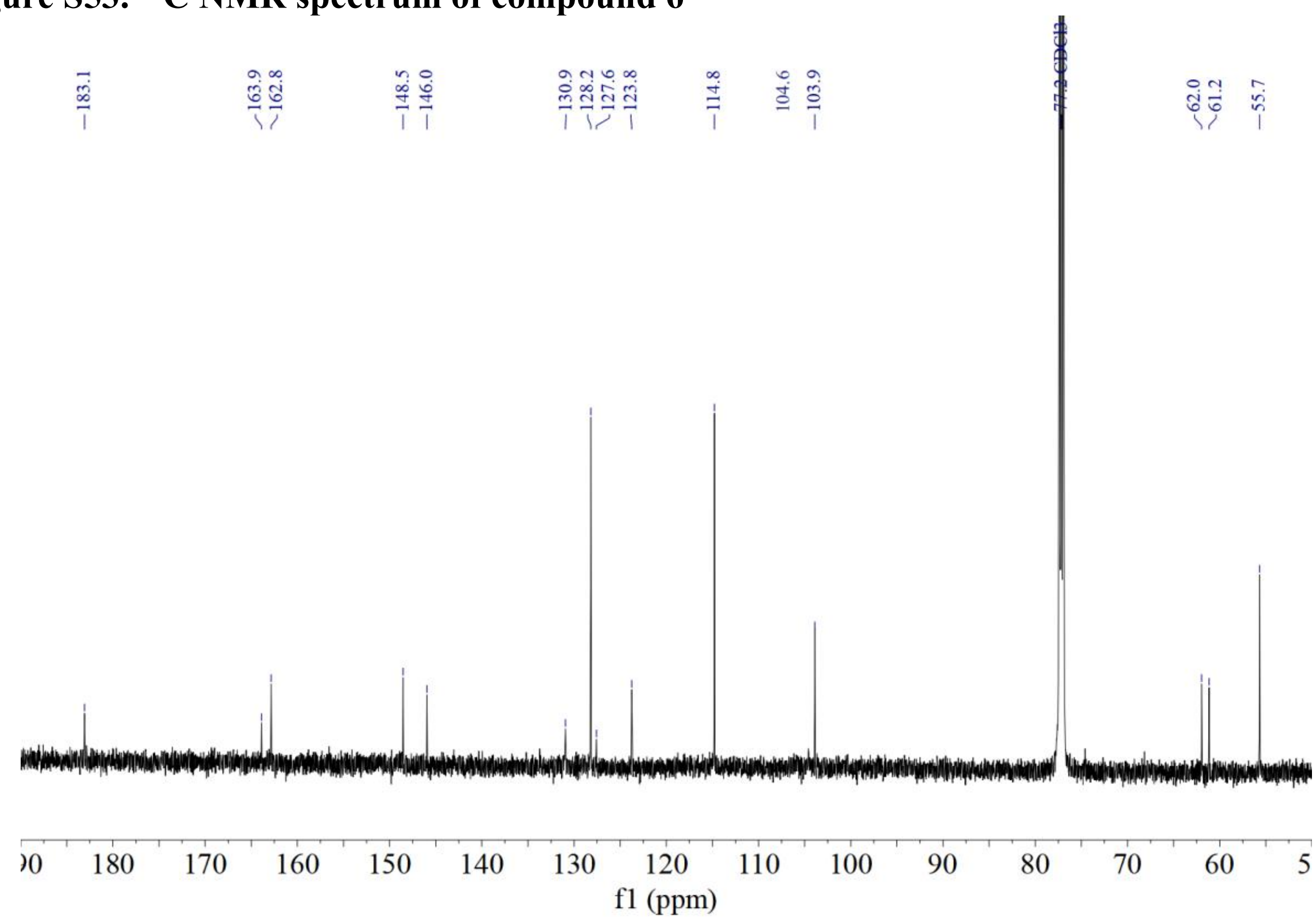

**Figure S54. DEPT NMR spectrum of compound 6**

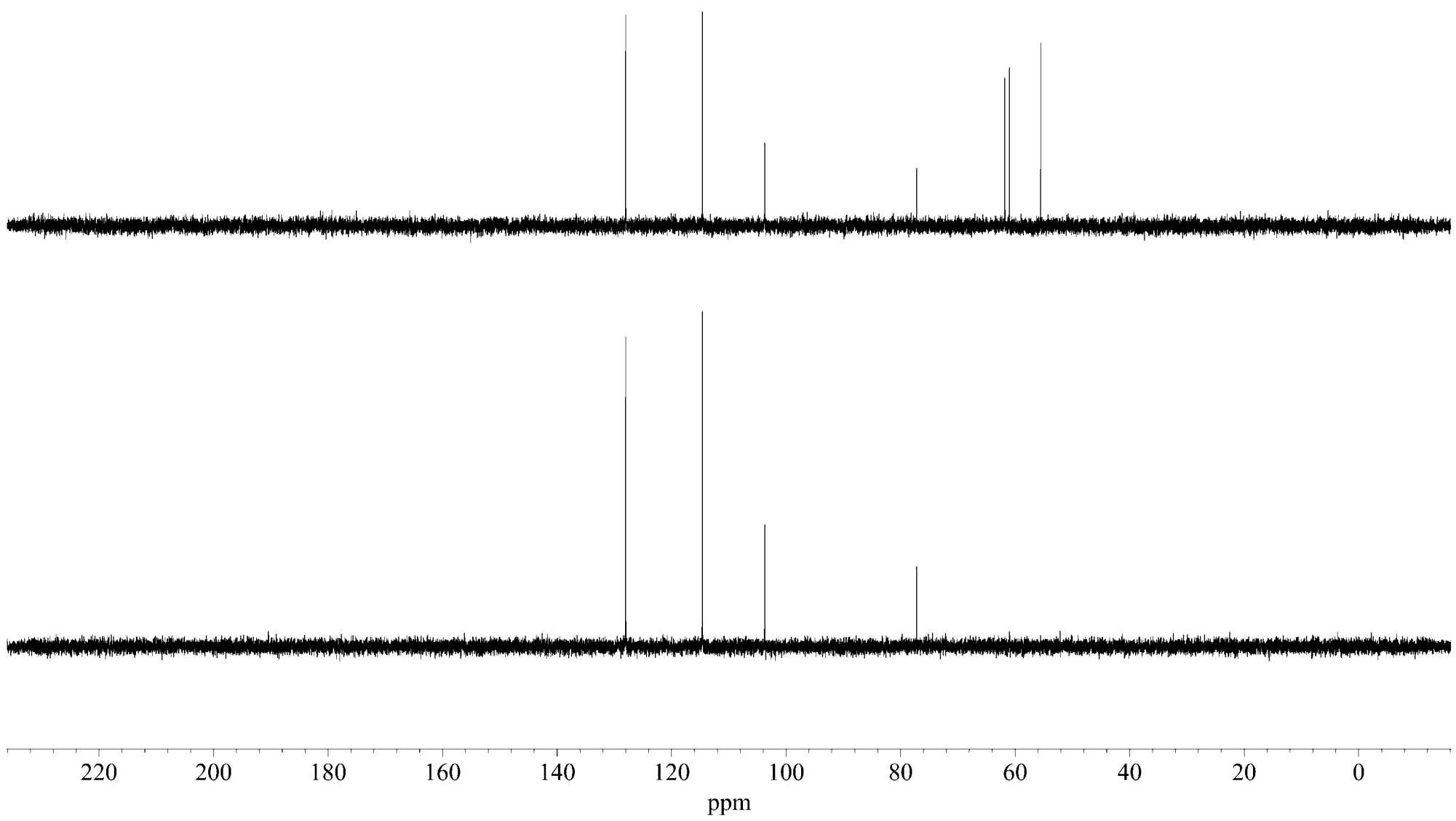


**Figure S55. COSY spectrum of compound 6**

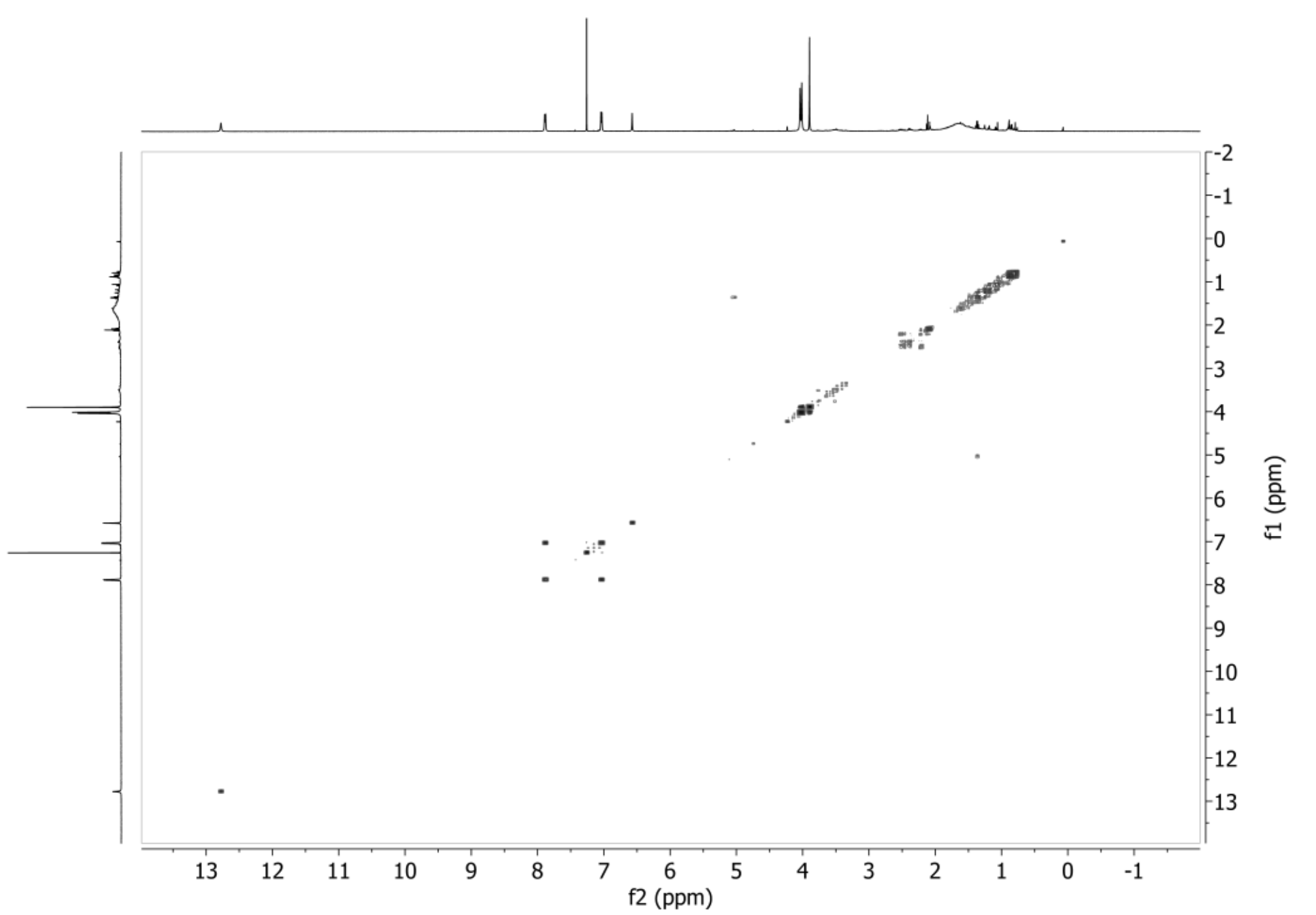

**Figure S56. HSQC spectrum of compound 6**

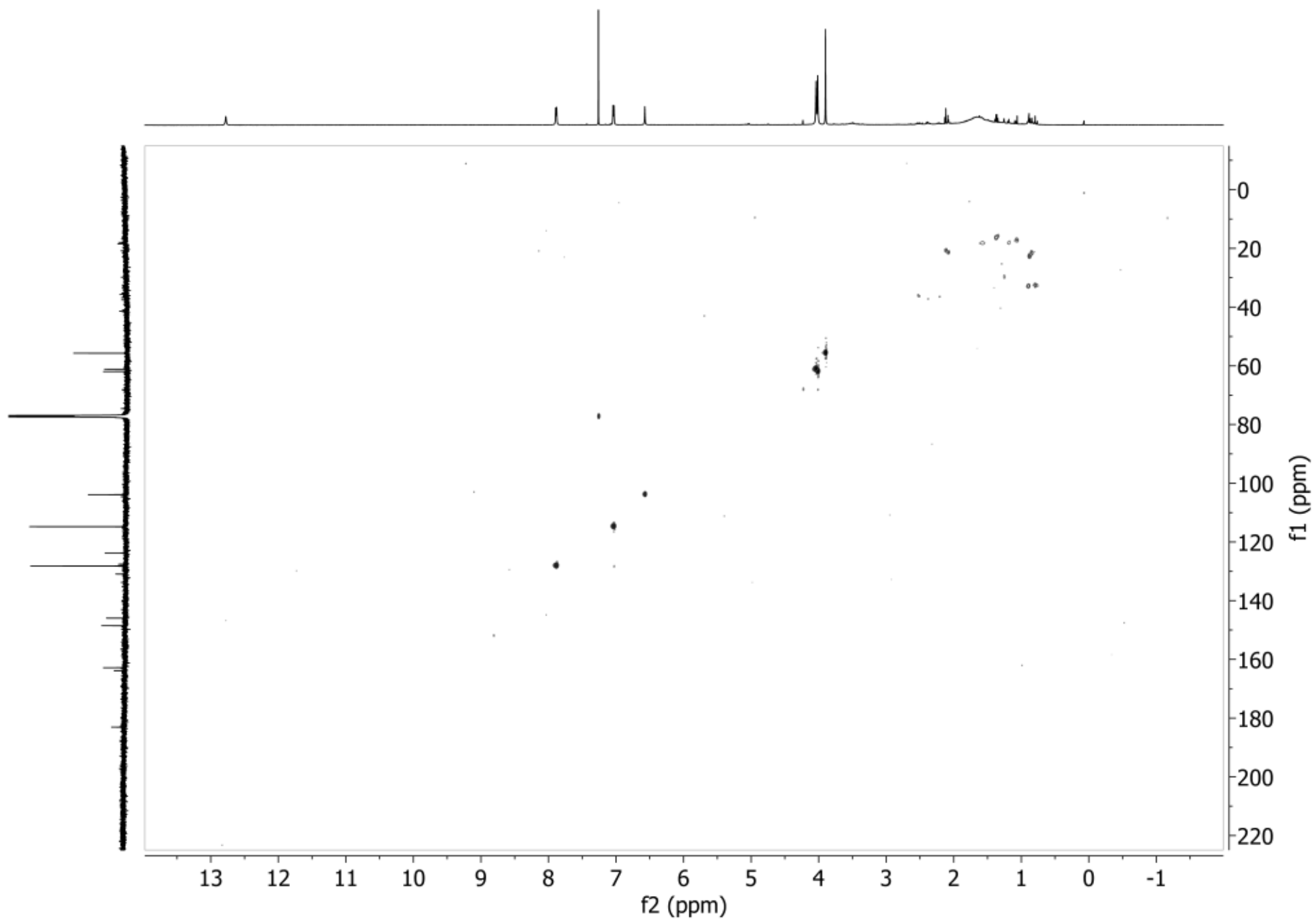


**Figure S57. HMBC spectrum of compound 6**

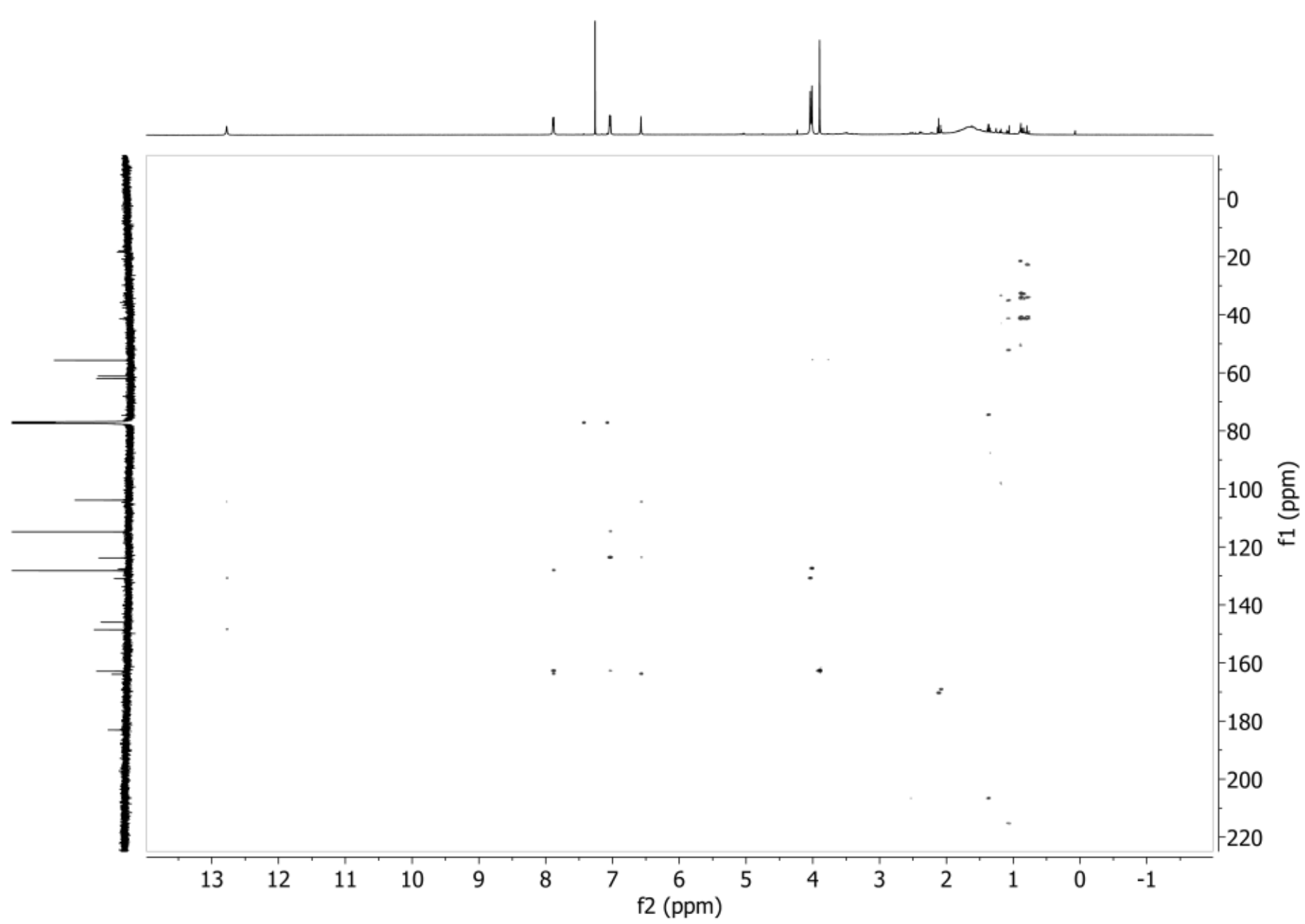

**Figure S58. NOESY spectrum of compound 6**

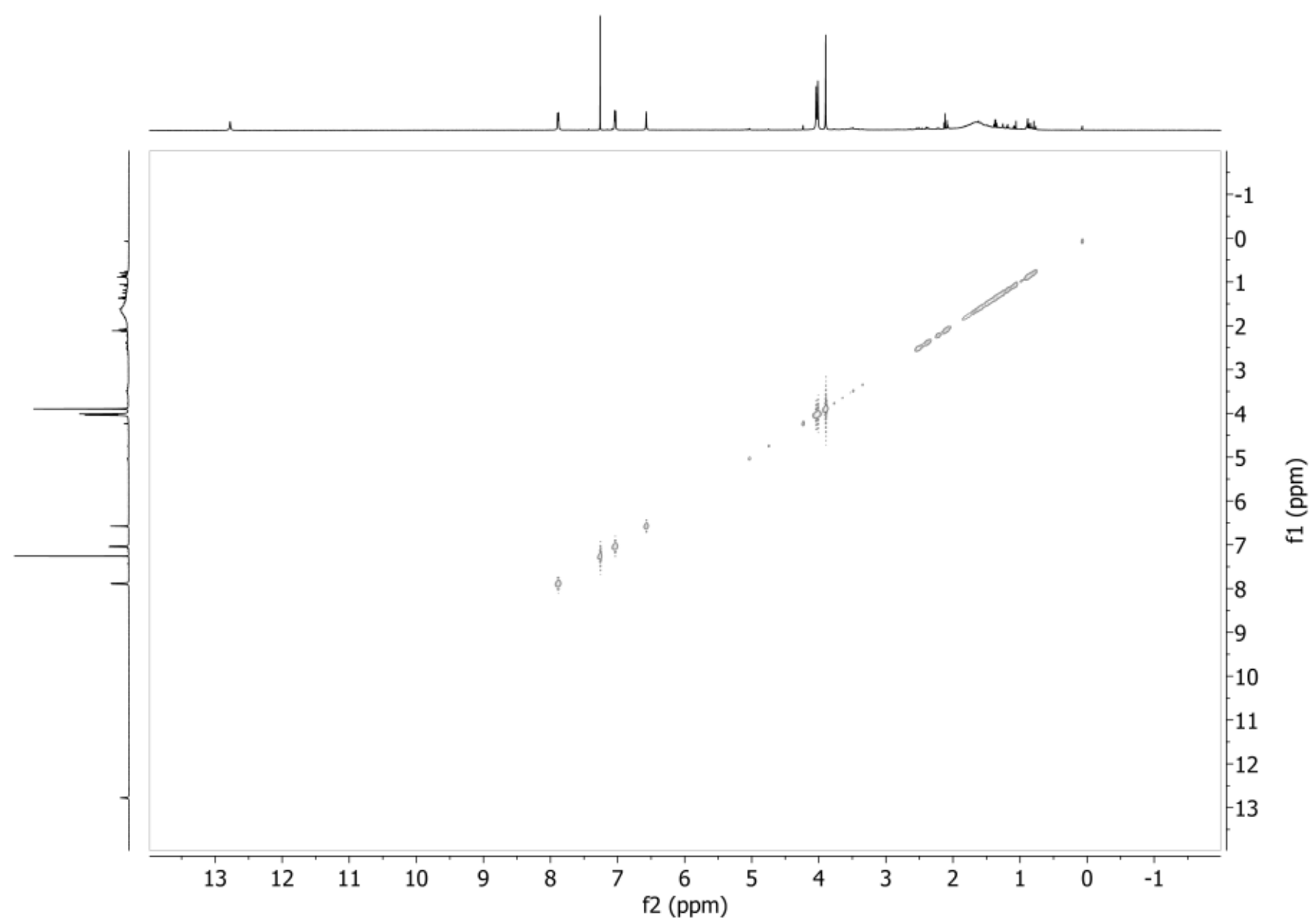


**Figure S59. ESI-MS spectrum of compound 6**

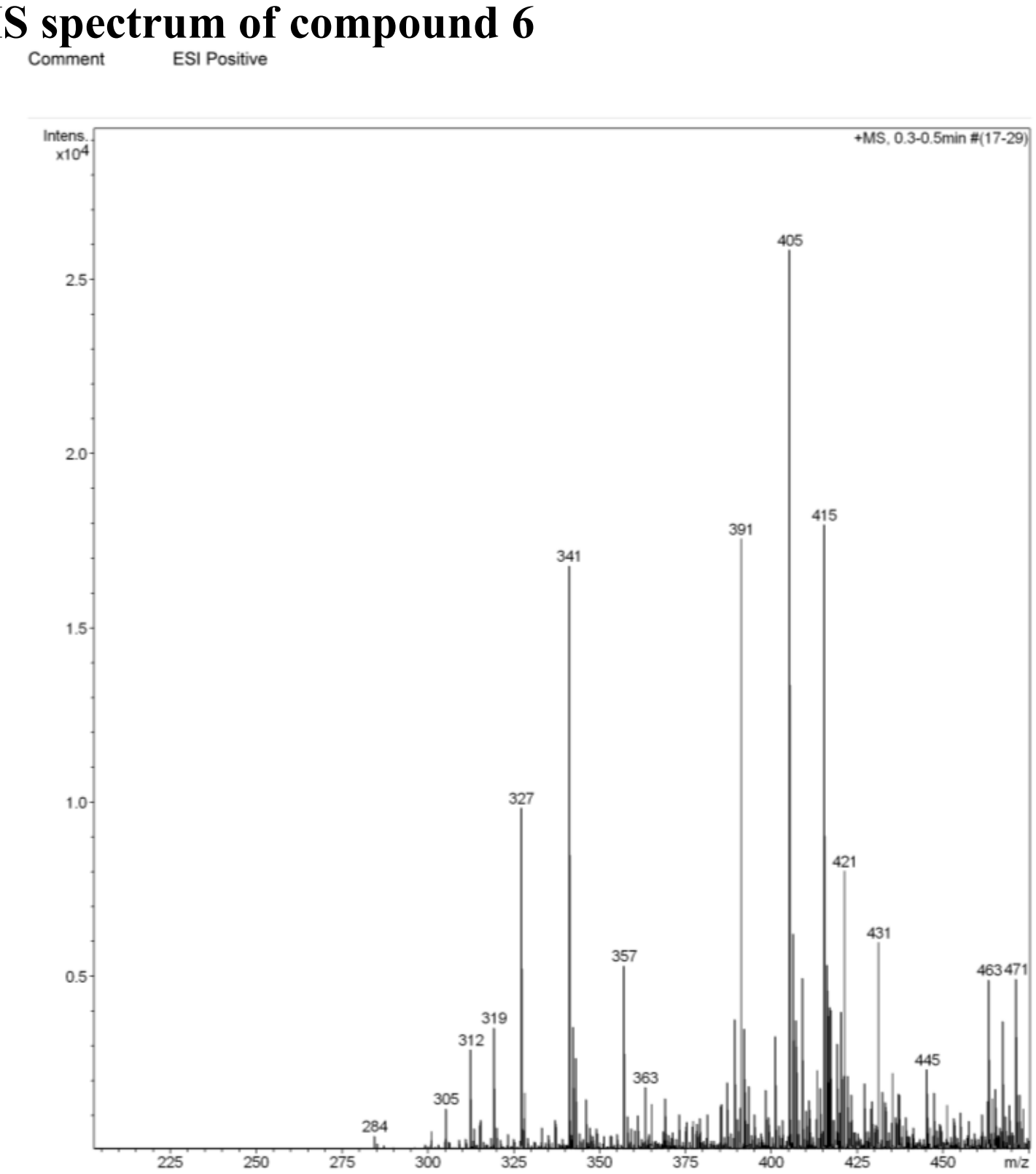

**Figure S60. UV and IR spectrum of compound 6**

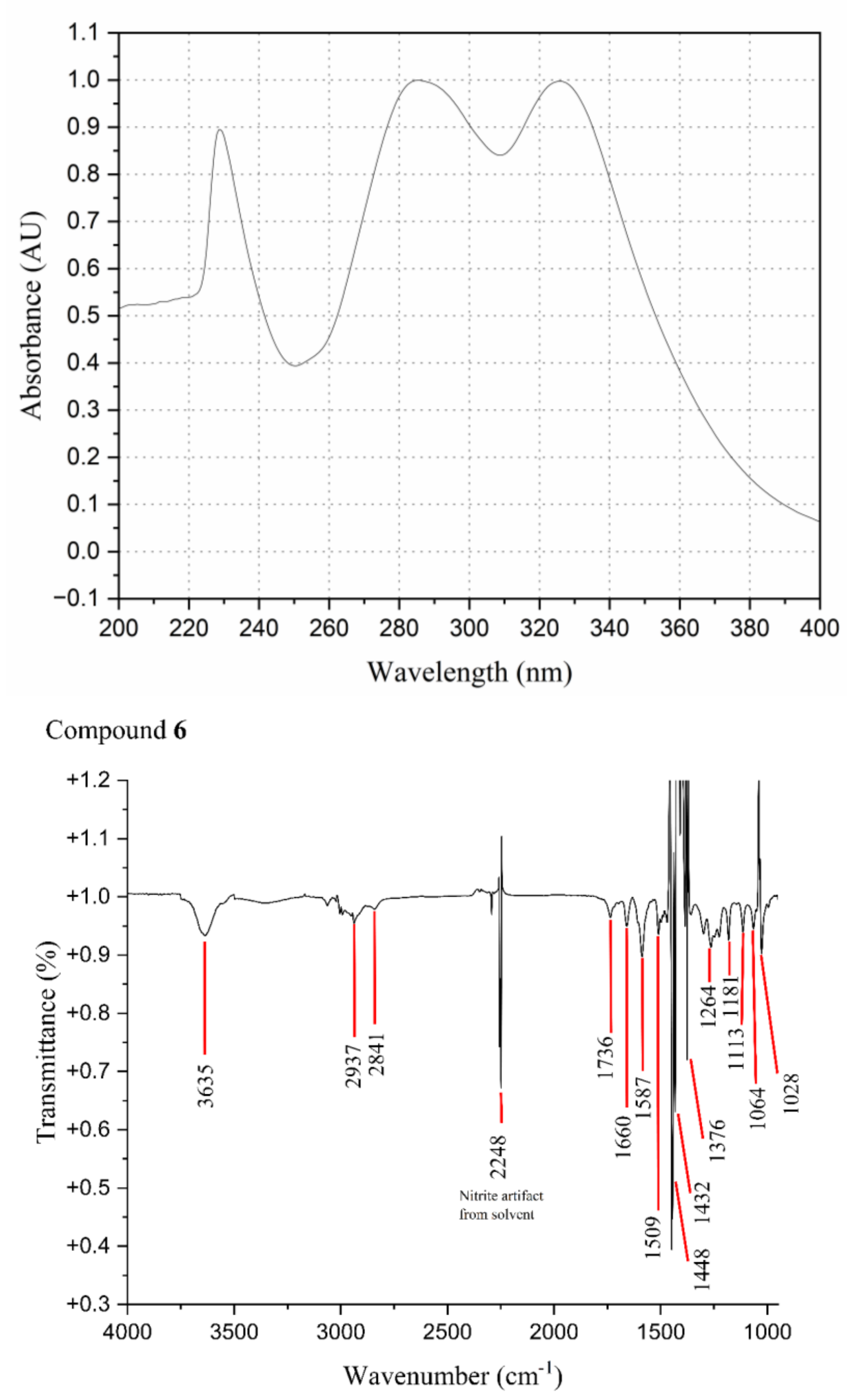


IR ($CH_3CN$) $\nu_{max}$ 3635, 2937, 2841, 1736, 1660, 1587, 1448, 1432, 1376 cm$^{-1}$.

## References

1. Hall, T. A. (1999). *BioEdit: a user-friendly biological sequence alignment editor and analysis program for Windows 95/98/NT.* Paper presented at the Nucleic acids symposium series..

2. Edgar, R. C. MUSCLE: multiple sequence alignment with high accuracy and high throughput. *Nucleic Acids Res.* 2004, 32, 1792-1797. doi:10.1093/nar/gkh340.

3. Tamura, K., Stecher, G., & Kumar, S. MEGA11: molecular evolutionary genetics analysis version 11. *Molecular Biology and Evolution*. 2021, 38, 3022-3027. doi:10.1093/molbev/msab120.

4. NCBI. Basic Local Alignment Search Tool (BLAST). Retrieved from https://blast.ncbi.nlm.nih.gov/Blast.cgi.

5. Vichai, V., & Kirtikara, K. Sulforhodamine B colorimetric assay for cytotoxicity screening. *Nature protocols*. 2006, 1, 1112-1116. doi:10.1038/nprot.2006.179.

6. Chen, W.-C., Chung, C.-H., Lu, Y.-C., Wu, M.-H., Chou, P.-H., Yen, J.-Y., . . . Cheng, J.-K. BMP-2 induces angiogenesis by provoking integrin α6 expression in human endothelial progenitor cells. *Biochem. Pharmacol.* 2018, 150, 256-266. doi:10.1016/j.bcp.2018.02.021.

7. Chung, C.-H., Chang, C.-H., Chen, S.-S., Wang, H.-H., Yen, J.-Y., Hsiao, C.-J., . . . Wang, P.-C. Butein inhibits angiogenesis of human endothelial progenitor cells via the translation dependent signaling pathway. *Evidence-Based Complementary and Alternative Medicine*. 2013, 2013, 943187. doi:10.1155/2013/943187.

8. Purnomo, K. A., Korinek, M., Tsai, Y.-H., Hu, H.-C., Wang, Y.-H., Backlund, A., . . . Chang, F.-R. Decoding Multiple Biofunctions of Maca on Its Anti-allergic, Anti-inflammatory, Anti-thrombotic, and Pro-angiogenic Activities. *Journal of Agricultural and Food Chemistry*. 2021, 69, 11856-11866. doi:10.1021/acs.jafc.1c03485.

9. Yang, C.-Y., Chen, C., Lin, C.-Y., Chen, Y.-H., Lin, C.-Y., Chi, C.-W., . . . Tang, C.-H. Garcimultiflorone K inhibits angiogenesis through Akt/eNOS-and mTOR-dependent pathways in human endothelial progenitor cells. *Phytomedicine*. 2019, 64, 152911. doi:10.1016/j.phymed.2019.152911.

10. Yang, C. Y., Chen, C., Lin, C. Y., Chen, Y. H., Lin, C. Y., Chi, C. W., . . . Wang, S. W. Garcimultiflorone K inhibits angiogenesis through Akt/eNOS- and mTOR-dependent pathways in

human endothelial progenitor cells. *Phytomedicine*. 2019, 64, 152911. doi:10.1016/j.phymed.2019.152911.

11. Dutta, B., Banerjee, A., Chakraborty, P., & Bandopadhyay, R. In silico studies on bacterial xylanase enzyme: Structural and functional insight. *Journal of Genetic Engineering and Biotechnology*. 2018, 16, 749-756. doi:10.1016/j.jgeb.2018.05.003.

12. Tran, N. T., Jakovlić, I., & Wang, W.-M. In silico characterisation, homology modelling and structure-based functional annotation of blunt snout bream (Megalobrama amblycephala) Hsp70 and Hsc70 proteins. *Journal of Animal Science and Technology*. 2015, 57, 1-9. doi:10.1186/s40781-015-0077-x.

13. Laskowski, R. A., Jabłońska, J., Pravda, L., Vařeková, R. S., & Thornton, J. M. PDBsum: Structural summaries of PDB entries. *Protein Sci.* 2018, 27, 129-134. doi:10.1002/pro.3289.

14. Raval, K., & Ganatra, T. Basics, types and applications of molecular docking: A review. *IP International Journal of Comprehensive and Advanced Pharmacology*. 2022, 7, 12-16. doi:10.18231/j.ijcaap.2022.003.

15. Bugata, B. K., Dowluru, K., & Avupati, V. R. Computational virtual screening of sulfonylurea chalcones as new class of 5-lipoxygenase inhibitors by molecular docking studies. *International Journal of Computer Applications*. 2013, 78. doi:10.5120/13587-1426.

16. El Rhabori, S., El Aissouq, A., Daoui, O., Elkhattabi, S., Chtita, S., & Khalil, F. Design of new molecules against cervical cancer using DFT, theoretical spectroscopy, 2D/3D-QSAR, molecular docking, pharmacophore and ADMET investigations. *Heliyon*. 2024, 10. doi:10.1016/j.heliyon.2024.e24551.

17. Lewis-Atwell, T., Townsend, P. A., & Grayson, M. N. Comparisons of different force fields in conformational analysis and searching of organic molecules: A review. *Tetrahedron*. 2021, 79, 131865. doi:10.1016/j.tet.2020.131865.

18. Renadi, S., Pratita, A. T. K., Mardianingrum, R., & Ruswanto, R. The Potency of Alkaloid Derivates as Anti-Breast Cancer Candidates: In Silico Study. *Jurnal Kimia Valensi*. 2023, 9, 89-108. doi:10.15408/jkv.v9i1.31481.

19. Shalini, T., Manivel, G., Ragothaman, P., Velu, R. K., & Senthilraja, P. Secondary metabolite profiling using HR-LCMS, antioxidant and anticancer activity of Bacillus cereus PSMS6 methanolic extract: In silico and in vitro study. *Biotechnology Reports*. 2024, 42, e00842. doi:10.1016/j.btre.2024.e00842.

20. Elseginy, S. A. Identifying and characterising promising small molecule inhibitors of kinesin spindle protein using ligand-based virtual screening, molecular docking, molecular dynamics and MM-GBSA calculations. *J. Comput. Aided Mol. Des.* 2024, 38, 16. doi:10.1007/s10822-024-00553-5.

21. Tam, B., Sinha, S., & Wang, S. M. Combining Ramachandran plot and molecular dynamics simulation for structural-based variant classification: Using TP53 variants as model. *Computational and Structural Biotechnology Journal*. 2020, 18, 4033-4039. doi:10.1016/j.csbj.2020.11.041.

22. Carugo, O., & Djinović-Carugo, K. Half a century of Ramachandran plots. *Acta Crystallogr. Sect. D. Biol. Crystallogr.* 2013, 69, 1333-1341. doi:10.1107/S090744491301158X.

23. Colovos, C., & Yeates, T. O. Verification of protein structures: patterns of nonbonded atomic interactions. *Protein Sci.* 1993, 2, 1511-1519. doi:10.1002/pro.5560020916.

24. Ruswanto, R., Miftah, A. M., & Tjahjono, D. H. In silico study of 1-benzoyl-3-methylthiourea derivatives activity as epidermal growth factor receptor (EGFR) tyrosine kinase inhibitor candidates. *Chemical Data Collections*. 2021, 34, 100741. doi:10.1016/j.cdc.2021.100741.

25. Pandey, A. K., & Verma, S. An *in-silico* evaluation of dietary components for structural inhibition of SARS-Cov-2 main protease. *J. Biomol. Struct. Dyn.* 2022, 40, 136-142. doi:10.1080/07391102.2020.1809522.

26. Herschlag, D., & Pinney, M. M. Hydrogen bonds: simple after all? *Biochemistry*. 2018, 57, 3338-3352. doi:10.1021/acs.biochem.8b00217.

27. Mohanty, M., & Mohanty, P. S. Molecular docking in organic, inorganic, and hybrid systems: a tutorial review. *Monatshefte für Chemie-Chemical Monthly*. 2023, 154, 683-707. doi:10.1007/s00706-023-03076-1.

28. Bitencourt-Ferreira, G., Veit-Acosta, M., & de Azevedo, W. F. Van der Waals potential in protein complexes. *Docking Screens for Drug Discovery*. 2019, 79-91. doi:10.1007/978-1-4939-9752-7_6.

29. Rajan, S., Baek, K., & Yoon, H. S. C-H… O hydrogen bonds in FK506-binding protein–ligand interactions. *Journal of Molecular Recognition*. 2013, 26, 550-555. doi:10.1002/jmr.2299.

30. Sarkhel, S., & Desiraju, G. R. N-H… O, O-H… O, and C-H… O hydrogen bonds in protein–ligand complexes: Strong and weak interactions in molecular recognition. *Proteins: Structure, Function, and Bioinformatics*. 2004, 54, 247-259. doi:10.1002/prot.10567.

31. Miao, L.-L., Zhou, Q.-M., Peng, C., Liu, Z.-H., & Xiong, L. Leonurus japonicus (Chinese motherwort), an excellent traditional medicine for obstetrical and gynecological diseases: A comprehensive overview. *Biomed Pharmacother*. 2019, 117, 109060. doi:10.1016/j.biopha.2019.109060.

32. Zhang, X.-J., Zhong, W.-M., Liu, R.-X., Wang, Y.-M., Luo, T., Zou, Y., . . . Xiao, W.-L. Structurally diverse labdane diterpenoids from Leonurus japonicus and their anti-inflammatory properties in LPS-induced RAW264.7 cells. *J. Nat. Prod.* 2020, 83, 2545-2558. doi:10.1021/acs.jnatprod.9b00597.
